# *Limosilactobacillus reuteri* for oral mucositis in patients undergoing radiotherapy for malignant head and neck tumors: a randomized controlled trial

**DOI:** 10.1038/s41467-026-78088-6

**Published:** 2026-09-29

**Authors:** Kun Gao, Yuan-tai Li, Yi-yan Pei, Zhi-hui Li, Jia-gang He, Zheng Jiang, Zhe-ran Liu, Yu Min, Zi-xia Li, Ping Ai, Shu-ming Ji, Wen-yan Guo, Xing-chen Peng

**Affiliations:** 1 Department of Biotherapy, Cancer Center, West China Hospital, Sichuan University, Chengdu, Sichuan China; 2 Sichuan Provincial Key Laboratory of Nuclear Physics and Medical Research, Sichuan University, Chengdu, Sichuan China; 3 Department of Oncology and Radiotherapy Center, Chengdu Seventh People’s Hospital, Chengdu, China; 4 Department of Oncology, The General Hospital of Western Theater Command, Chengdu, Sichuan China; 5 Department of Medical Education, Kweichow Moutai Hospital, Zunyi, Guizhou China; 6 Department of Cariology and Endodontics, West China Hospital of Stomatology, Sichuan University, Chengdu, Sichuan China; 7 Division of Head & Neck Tumor Multimodality Treatment, Cancer Center, West China Hospital, Sichuan University, Chengdu, Sichuan China; 8 Institute of Hospital Management, West China Hospital, Sichuan University, Chengdu, Sichuan China; 9 Clinical Research Management Office, West China Hospital, Sichuan University, Chengdu, Sichuan China

**Keywords:** Head and neck cancer, Cancer therapy, Mucositis

## Abstract

Oral mucositis (OM) is a major toxicity in patients with head and neck cancer undergoing radiotherapy. Here we conducted a double-blind, placebo-controlled, randomized phase II trial (NCT06285591) from March 2024 to January 2025 at West China Hospital of Sichuan University to evaluate whether *Limosilactobacillus reuteri* (*L. reuteri*) supplementation could reduce severe OM (SOM; WHO Oral Toxicity criteria grade ≥3). A total of 160 patients were randomly assigned to receive *L. reuteri* probiotic lozenges or placebo twice daily during radiotherapy. The primary endpoint was the incidence of SOM, and secondary endpoints included SOM onset and duration, as well as the incidence, onset, and duration of any-grade OM. Safety outcomes were monitored throughout the study. The primary endpoint was met, with a lower incidence of SOM in the *L. reuteri* group than in the placebo group (37.5% vs 61.3%; relative risk 0.61; 95% confidence interval 0.44-0.85; absolute risk reduction 23.8 percentage points, 95% CI 8.7% to 38.8%; *P* = 0.004). *L. reuteri* supplementation also delayed SOM onset and shortened SOM duration. No significant safety concerns were observed. These findings indicate that *L. reuteri* supplementation reduces SOM incidence and severity in patients with head and neck cancer receiving radiotherapy.

## Abstract (web summary)

Oral mucositis is an important complication for patients with head and neck cancer undergoing radiotherapy. Here the authors report the results of a randomized phase II comparing the activity of the probiotic *Limosilactobacillus reuteri* vs placebo in alleviating oral mucositis in patients with head and neck cancer receiving radiotherapy.

## Introduction

Oral mucositis (OM) is a ubiquitous complication among patients with head and neck cancer receiving radiotherapy, with severe OM (SOM) developing in approximately 50-70% of cases^1–3^. SOM is characterized by extensive mucosal damage, leading to persistent intense pain, impaired oral intake, and difficulties in talking and swallowing^4^. Collectively, these acute toxicities lead to malnutrition, profoundly diminish quality of life, and frequently necessitate unplanned radiotherapy interruptions, thereby jeopardizing therapeutic outcomes in patients with head and neck cancer. Currently, the only agent approved by both the U.S. Food and Drug Administration (FDA) and the European Medicines Agency (EMA) for OM is palifermin, a recombinant human keratinocyte growth factor, but it is approved only for OM prophylaxis in hematological malignancy patients receiving hematopoietic stem cell transplantation^5^. Among other interventions recommended by the Multinational Association of Supportive Care in Cancer/International Society of Oral Oncology (MASCC/ISOO), benzydamine mouthwash has Level I evidence for preventing OM, yet it is recommended only for head and neck cancer patients treated with radiotherapy at less than 50 Gy^6^. Other agents, including oral glutamine, antimicrobials, anesthetics, analgesics, and coating agents, are primarily used for symptomatic support and are limited to Level II recommendations^6^. Thus, the development of novel management strategies for radiation-induced oral mucositis (RIOM) remains paramount.

Accumulating findings suggest that the radiotherapy-induced restructuring of the oral microbiota, specifically characterized by the depletion of commensal taxa and the concomitant enrichment of pathogenic taxa, contributes to the development and progression of RIOM, and that oral probiotics may mitigate mucosal injury by restoring microbial homeostasis^3,7–9^. Among various probiotic strains, *Limosilactobacillus reuteri* (*L. reuteri*) exhibits notable potential for managing OM through multiple, complementary mechanisms^10,11^. A key attribute is its ability to form stable, protective biofilms on oral epithelia, which promotes mucosal colonization and competitively excludes pathogenic bacteria^12,13^. Furthermore, *L. reuteri* targets oxidative stress via its metabolite reuterin to reduce direct tissue damage and enhance epithelial barrier integrity, synergistically countering key drivers of mucosal dysfunction in OM^14–17^. Together, these actions help restore microbial balance and dampen inflammatory cascades, underpinning its documented efficacy in oral inflammatory diseases such as gingivitis and periodontitis^18–22^.

Despite promising preliminary findings, a recent randomized clinical trial reported controversial results, highlighting that its effectiveness in managing RIOM is not yet fully understood and requires further investigation^23^. In this study, we hypothesized that modulation of the oral microbiota through *L. reuteri* supplementation would alleviate RIOM in patients with head and neck cancer undergoing radiotherapy. Here, we assess the efficacy of *L. reuteri* probiotic lozenges in a double-blind, placebo-controlled, randomized Phase II trial and show that *L. reuteri* supplementation reduces the incidence and severity of SOM during radiotherapy.

## Results

### Baseline clinical characteristics

**Fig. 1:**
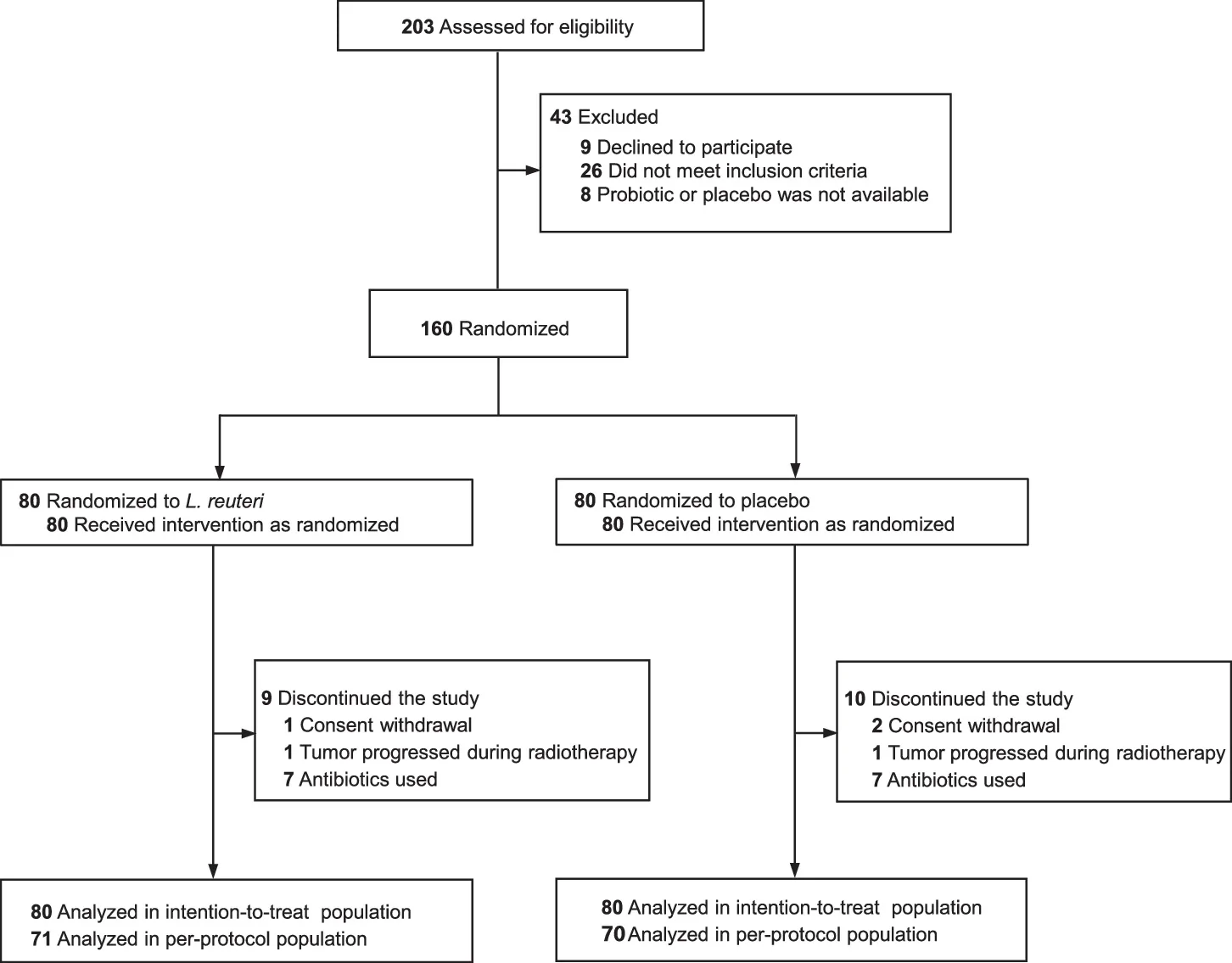
CONSORT diagram. patient random assignment; *L. reuteri*, *Limosilactobacillus reuteri*.

Building on promising preclinical data demonstrating *L. reuteri’s* efficacy in managing RIOM (Supplementary Fig. 1), a randomized controlled trial was initiated. In total, 160 eligible patients were enrolled, with all included in the Intention-To-Treat (ITT) analysis (Fig. 1). Nine patients in the *L. reuteri* group and 10 in the placebo group withdrew from the study (Supplementary Table 1), leaving 71 and 70 patients, respectively, available for Per-Protocol-Set (PPS) analysis. Two patients withdrew due to disease progression, and three patients withdrew consent due to their request to add other medications for OM prevention during the early stage of radiotherapy, whereas the remaining patients withdrew due to antibiotic use toward the end of radiotherapy. The mean adherence rate was 94.3% in the *L. reuteri* group and 92.6% in the placebo group in the ITT population, and 96.4% and 95.0% in the PP population, respectively. The two groups had balanced baseline characteristics (Table 1). The mean (SD) age of the study participants was 56.8 (11.8) years. Most of the participants were male (80.6%, 129 of 160). The majority of patients had nasopharyngeal carcinoma (30.0%), followed by oral cavity carcinoma (27.5%) and oropharyngeal carcinoma (16.2%). In the *L. reuteri* group, 37 patients (46.2%) received concurrent chemotherapy, compared with 34 (42.5%) in the placebo group. The *L. reuteri* and placebo groups had median oral cavity doses of 40.3 Gy and 40.1 Gy, respectively.

**Table 1:** Baseline patient characteristics

Characteristic	*L. reuteri* (*n* = 80)	Placebo (*n* = 80)	All Patients (*N* = 160)
Sex, No. (%)			
Male	68 (85.0)	61 (76.2)	129 (80.6)
Female	12 (15.0)	19 (23.8)	31 (19.4)
Age, years, mean ± SD	56.3 ± 11.3	57.4 ± 12.4	56.8 ± 11.8
BMI, kg/m^2^, mean ± SD	22.9 ± 3.6	22.6 ± 2.4	22.8 ± 3.0
ECOG PS, No. (%)			
0	54 (67.5)	53 (66.2)	107 (66.9)
1	24 (30.0)	25 (31.2)	49 (30.6)
2	2 (2.5)	2 (2.5)	4 (2.5)
Tumor pathologic types, No. (%)			
Squamous carcinoma	67 (83.8)	73 (91.2)	140 (87.5)
Others	13 (16.2)	7 (8.8)	20 (12.5)
Tumor site, No. (%)			
Nasopharynx	24 (30.0)	24 (30.0)	48 (30.0)
Oral cavity	22 (27.5)	22 (27.5)	44 (27.5)
Oropharynx	13 (16.2)	13 (16.2)	26 (16.2)
Hypopharynx	10 (12.5)	10 (12.5)	20 (12.5)
Larynx	8 (10.0)	7 (8.8)	15 (9.4)
Others ^a^	3 (3.8)	4 (5.0)	7 (4.4)
Treatment type, No. (%)			
Definitive	38 (47.5)	40 (50.0)	78 (48.8)
Postoperative treatment	42 (52.5)	40 (50.0)	82 (51.2)
Concurrent chemotherapy, No. (%)			
Yes	37 (46.2)	34 (42.5)	71 (44.4)
No	43 (53.8)	46 (57.5)	89 (55.6)
TNM stage ^b^, No. (%)			
I-II	18 (22.5)	16 (20.0)	34 (21.2)
III	23 (28.7)	22 (27.5)	45 (28.1)
IVa	25 (31.2)	29 (36.2)	54 (33.8)
IVb	14 (17.5)	13 (16.2)	27 (16.9)
Tobacco use, No. (%)			
Yes	38 (47.5)	43 (53.8)	81 (50.6)
No	42 (52.5)	37 (46.2)	79 (49.4)
Alcohol use, No. (%)			
Yes	43 (53.8)	35 (43.8)	78 (48.8)
No	37 (46.2)	45 (56.2)	82 (51.2)
Oropharyngeal cancer HPV status, No. (%)			
Positive	5 (38.5)	3 (23.1)	8 (30.8)
Negative	8 (61.5)	10 (76.9)	18 (69.2)
Nasopharynx cancer EBV status, No. (%)			
Positive	24 (100.0)	24 (100.0)	48 (100.0)
Negative	0 (0.0)	0 (0.0)	0 (0.0)
Total radiotherapy dose, Gy, median (IQR)	66.0 (7.0)	66.0 (6.4)	66.0 (7.0)
Dose to oral cavity, Gy, median (IQR)	40.3 (13.9)	40.1 (10.9)	40.3 (13.3)
Baseline salivary rates, ml/min, mean ± SD	0.8 ± 0.4	0.8 ± 0.3	0.8 ± 0.4

Body mass index (BMI) is calculated by taking the weight in kilograms and dividing it by the square of the height measured in meters. Abbreviations: *L. reuteri*, *Limosilactobacillus reuteri*; ECOG PS, Eastern Cooperative Oncology Group performance status; *SD* standard deviation; a: The ‘others’ category included malignancies of the salivary glands, sinonasal tract, external and middle ear, and occult primaries; b: According to the AJCC 8th edition staging system.

### Primary endpoint

The SOM incidence was significantly lower in the *L. reuteri* group (37.5%, 30 of 80 patients) than in the placebo group (61.3%, 49 of 80 patients), with an absolute difference of 23.8% in the ITT set (relative risk 0.61; 95% CI 0.44-0.85; *P* = 0.004; Table 2). To further evaluate the efficacy of *L. reuteri* while accounting for potential confounding factors, we performed multivariable analyses adjusting for clinical covariates. After adjustment for oral radiation dose, tumor site, and receipt of chemotherapy (Model 1), *L. reuteri* remained significantly associated with a reduced incidence of SOM (adjusted relative risk 0.62; 95% CI 0.45-0.84; *P* = 0.002; Table 2). Notably, these findings were robust to further adjustment for smoking and alcohol consumption history (Model 2), with the effect of *L. reuteri* remaining statistically significant (adjusted relative risk 0.62; 95% CI 0.46-0.85; *P* = 0.003; Table 2; Supplementary Table 2). In the PP population, consistent results were observed even after incorporating baseline Shannon index and the absolute abundance of *Actinomyces graevenitzii* into the Model 2 framework (adjusted relative risk 0.59; 95% CI 0.42-0.84; *P* = 0.003; Supplementary Table 3). Furthermore, post-hoc subgroup analyses suggested that the clinical benefit of *L. reuteri* was generally consistent across key clinical strata, including patients receiving concurrent chemoradiotherapy and those undergoing definitive radiotherapy (Supplementary Fig. 2).

**Table 2:** Efficacy results

Parameter	*L. reuteri* (*n* = 80)	Placebo (*n *= 80)	Effect size (95% CI)	*P*
Unadjusted primary analysis				
SOM incidence, %	37.5	61.3	0.61 (0.44–0.85)	.0044
SOM onset, days, median	NE	16.0	0.43 (0.27–0.67)	.0002
SOM duration, days, mean (SD)	6.4 (9.6)	14.9 (14.5)	0.43 (0.21–0.66)	.0009
OM incidence, %	56.3	78.8	0.71 (0.57–0.89)	.0041
OM onset, days, median	14.0	8.0	0.41 (0.28–0.60)	< .0001
OM duration, days, mean (SD)	19.9 (21.7)	35.8 (24.5)	0.56 (0.39–0.74)	.0033
Model 1				
SOM incidence, %	37.5	61.3	0.62 (0.45–0.84)	.0021
SOM onset, days, median	NE	16.0	0.44 (0.28–0.70)	.0005
SOM duration, days, mean (SD)	6.4 (9.6)	14.9 (14.5)	0.46 (0.25–0.70)	.0116
OM incidence, %	56.3	78.8	0.72 (0.59–0.88)	.0014
OM onset, days, median	14.0	8.0	0.39 (0.26–0.58)	< .0001
OM duration, days, mean (SD)	19.9 (21.7)	35.8 (24.5)	0.56 (0.40–0.74)	.0037
Model 2				
SOM incidence, %	37.5	61.3	0.62 (0.46–0.85)	.0026
SOM onset, days, median	NE	16.0	0.44 (0.28–0.69)	.0004
SOM duration, days, mean (SD)	6.4 (9.6)	14.9 (14.5)	0.48 (0.26–0.71)	.0163
OM incidence, %	56.3	78.8	0.72 (0.59–0.88)	.0013
OM onset, days, median	14.0	8.0	0.36 (0.25–0.54)	< .0001
OM duration, days, mean (SD)	19.9 (21.7)	35.8 (24.5)	0.57 (0.41–0.75)	.0074

Note: Unadjusted incidence comparisons used the χ² test (RR and its 95% CI were computed separately using the Katz logarithmic method); onset time used the log-rank test (HR, 95% CI); duration used a two-part model (MR, 95% CI). Model 1 adjusted for tumor site, oral radiotherapy dose, and chemotherapy. Model 2 extended Model 1 by additionally adjusting for smoking history and alcohol use history. Modified Poisson regression was used for incidence in adjusted models (aRR, 95% CI); Cox regression was used for onset in adjusted models (aHR, 95% CI); a two-part model was used for duration (aMR, 95% CI). As the proportional hazards assumption was violated for onset endpoints, the results of the piecewise Cox regression are presented in Supplementary Table 4. Abbreviations: *L. reuteri*, *Limosilactobacillus reuteri*, *OM* oral mucositis, *SOM* severe OM; *SD* standard deviation, *RR* relative risk, *HR* hazard ratio, *MR* mean ratio, *aRR* adjusted relative risk, *aHR* adjusted hazard ratio, *aMR* adjusted mean ratio, *CI* confidence interval, *NE* not estimable.

### Secondary endpoints

**Fig. 2:**
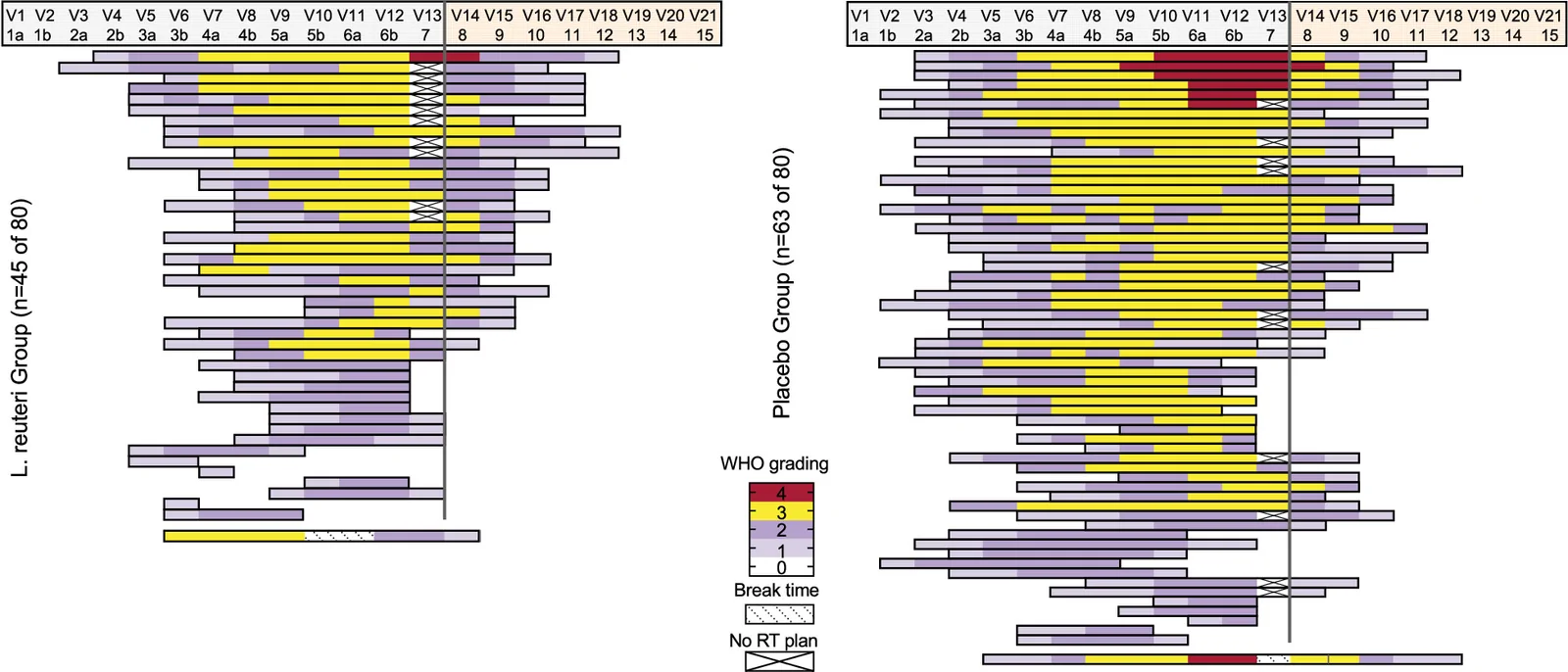
Swimmer plot displaying the OM scores for subsets of patients in the *Limosilactobacillus reuteri* group (*n* = 45, left) and placebo group (*n* = 63, right) who developed OM. This figure displays only participants from both the *L. reuteri* group and the placebo group who developed OM. Each horizontal strip represents an individual patient. The top section indicates the timeline during and post-radiotherapy, with a vertical gray line indicating the end of radiotherapy. The bottom strip highlights patients who experienced treatment breaks during radiotherapy (defined as ≥ 5 consecutive fractions). OM was assessed twice a week during radiotherapy and once a week for 8 weeks after radiotherapy. Red, WHO grade 4; yellow, WHO grade 3; purple, WHO grade 2; mauve, WHO grade 1; white, WHO grade 0; diagonal stripes, break time (Radiotherapy interruptions specifically attributable to SOM); X, no scheduled radiotherapy (The period at Week 7 with no scheduled radiotherapy, as patients had completed all radiotherapy by Week 6); V, the visit for oral mucositis assessment; OM, oral mucositis; SOM, severe oral mucositis; *L. reuteri*, *Limosilactobacillus reuteri*. Source data are provided as a Source Data file.

**Fig. 3:**
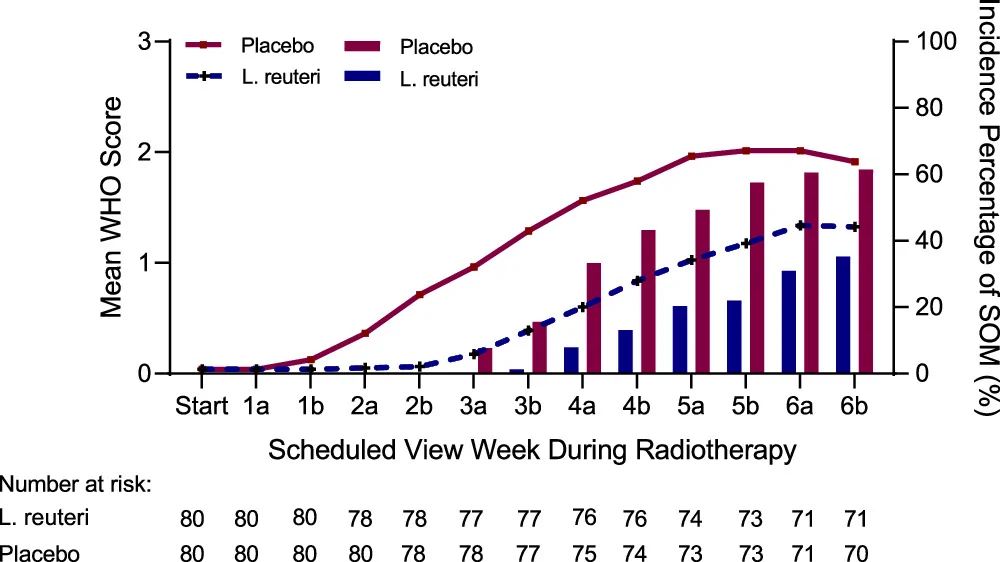
Mean WHO scores and incidence of SOM in the *Limosilactobacillus reuteri* group (*n* = 80) compared with the placebo group (*n* = 80) during radiotherapy. The bars represent the incidence of SOM, and the lines represent the mean WHO score. WHO assessments were performed twice weekly during radiotherapy; SOM, severe oral mucositis. The data presented are limited to the radiotherapy period to illustrate the dynamics of OM in direct relation to *L. reuteri* supplementation. Source data are provided as a Source Data file.

The time to SOM onset was markedly delayed in the *L. reuteri* group versus placebo (median, not reached vs 16.0 days; hazard ratio 0.43; 95% CI 0.27-0.67; *P* = 0.0002; Table 2 and Supplementary Fig. 3a). In addition, *L. reuteri’s* mean SOM duration was significantly shorter than placebo’s (mean, 6.4 vs 14.9 days; mean ratio 0.43; 95% CI 0.21-0.66; *P* = 0.0009; Table 2). Consistently, the administration of *L. reuteri* significantly decreased the incidence of OM (56.3% vs 78.8%; relative risk 0.71; 95% CI 0.57-0.89; *P* = 0.0041; Table 2), postponed the onset of OM (median, 14.0 days vs 8.0 days; hazard ratio 0.41; 95% CI 0.28-0.60; *P* < 0.0001; Table 2 and Supplementary Fig. 3b), and shortened the duration of OM (mean, 19.9 days vs 35.8 days; mean ratio 0.56; 95% CI 0.39-0.74; *P* = 0.0033; Table 2). Notably, although the proportional hazards assumption was violated for the onset endpoints, which may be attributed to the acute characteristics of OM, piecewise Cox regression revealed that the effect was still significant during the initial segment (Supplementary Table 4). Additional analyses of the onset and duration of OM and SOM in patients who exclusively developed these conditions also revealed differences between the *L. reuteri* and control groups (Supplementary Table 5). The distribution of OM throughout patients’ observation periods and its dynamic changes over the study duration were presented in a swimmer plot (Fig. 2). The time trajectories of mean WHO OM severity scores and SOM incidence in both groups showed that the *L. reuteri* group maintained consistently lower OM severity than the placebo group at all evaluated time points, with both groups exhibiting progressive symptom worsening that peaked during the final week of radiotherapy (Fig. 3).

### Exploratory endpoints

Nominal improvements in quality of life were observed in the *L. reuteri* group compared with the placebo group, with reductions in pain intensity (estimated mean difference, − 14.45; 95% CI − 19.39 to − 9.50; *P* < 0.001) and swallowing difficulties at the end of radiotherapy (estimated mean difference, −11.07; 95% CI − 15.89 to − 6.25; *P* < 0.001; Supplementary Fig. 4). At the end of radiotherapy, both groups exhibited declines in oral function, particularly in eating (estimated mean difference, − 0.42; 95% CI − 0.67 to − 0.16; *P* = 0.001) and swallowing capacity (estimated mean difference, − 0.28; 95% CI − 0.55 to − 0.01; *P* = 0.045; Supplementary Fig. 5). During radiotherapy, the *L. reuteri* group showed lower rates of parenteral nutrition use (23.9% vs 54.3%; relative risk 0.44; 95% CI 0.28-0.70; *P* < 0.001) and analgesic use (7.0% vs 21.4%; relative risk 0.33; 95% CI 0.13-0.87; *P* = 0.03) compared to the placebo group.

One patient in each group missed at least five consecutive radiation fractions due to SOM during radiotherapy (Fig. 2 and Supplementary Table 3). Gustatory perception was impaired during radiotherapy, with the most pronounced deficit observed for salty taste (estimated mean difference, 0.19; 95% CI − 0.07 to 0.45; *P* = 0.15; Supplementary Fig. 6). There was no significant difference in the incidence of grade ≥ 2 hyposalivation between the *L. reuteri* (81.69%, 58 of 71) and placebo (85.71%, 60 of 70) groups at the end of radiotherapy (Supplementary Table 6). The BMI reduction observed at the end of radiotherapy was comparable between groups, with decreases of 1.22 kg/m^2^ in the *L. reuteri* group and 1.49 kg/m^2^ in the placebo group.

### Oral microbiota

**Fig. 4:**
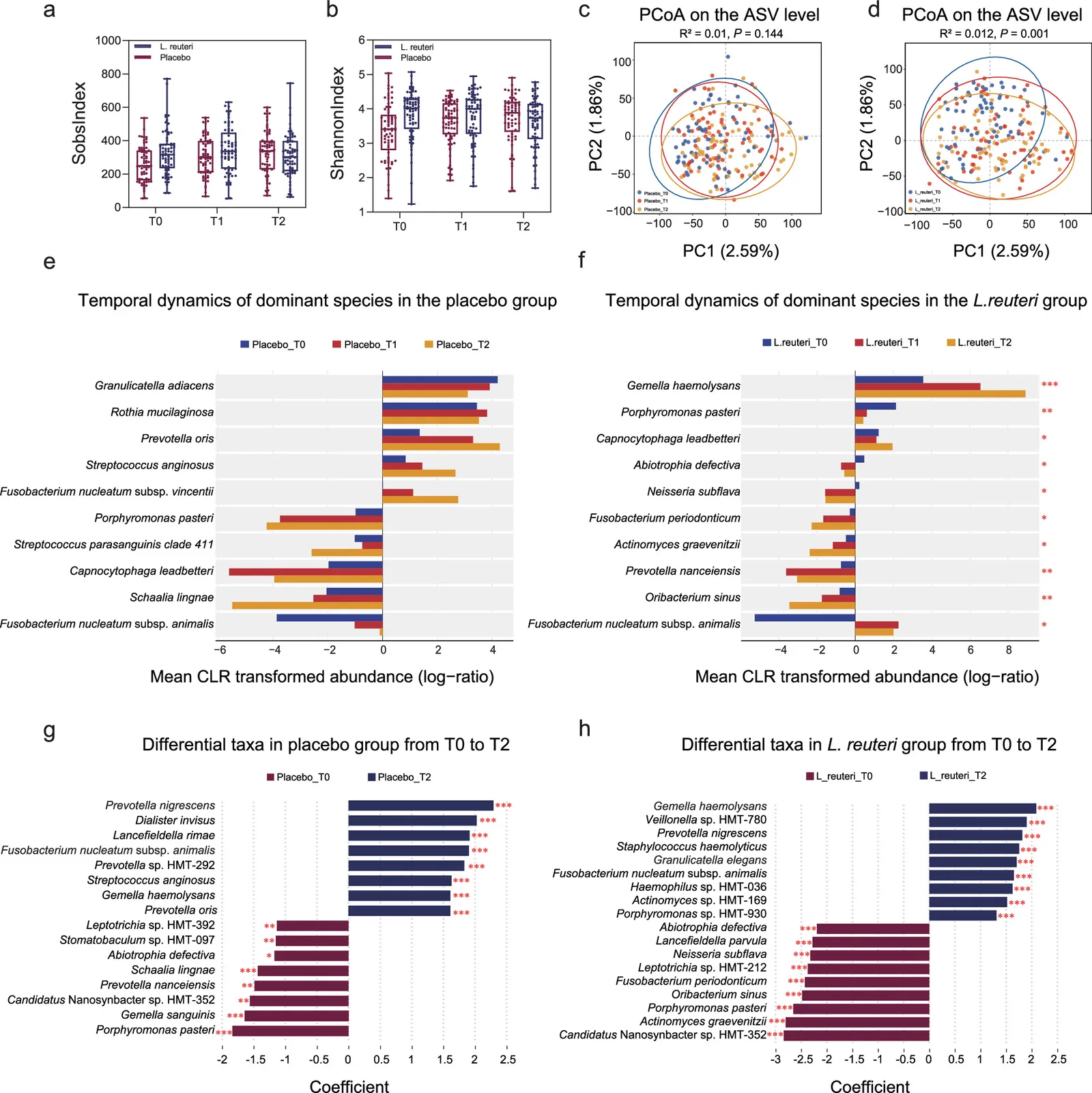
Salivary microbiota composition and temporal dynamics during radiotherapy in patients receiving *Limosilactobacillus reuteri* or placebo supplementation. Each group included samples from 70 participants at each time point. **a**, **b** The α diversity of the oral microbiota was evaluated using the Sobs and Shannon indices. Box-and-whisker plots represent the interquartile range (IQR), with the median indicated by the horizontal line and whiskers extending to the minimum and maximum observed values. **c**, **d** β diversity was assessed using Aitchison distances based on CLR-transformed taxon counts and visualized by PCoA. Statistical significance was evaluated using two-sided PERMANOVA with 999 permutations. **e**, **f** Temporal dynamics of dominant taxa at the species level in the placebo and *L. reuteri* groups during radiotherapy. Adjusted *P-*values were calculated using the two-sided Kruskal-Wallis H test with Benjamini-Hochberg FDR correction for multiple comparisons. **g**, **h** Differentially abundant taxa identified by MaAsLin2 analysis showing microbial changes from baseline (T0) to radiotherapy completion (T2) in the placebo and *L. reuteri* groups. MaAsLin2 models were fitted with appropriate covariate adjustment, and statistical significance was determined using two-sided tests. Asterisks in the figures denote nominal *P-*values, whereas the corresponding nominal *P-*values and FDR-adjusted *P-*values after multiple-testing correction are provided in the Source Data file. Positive and negative coefficients indicate enrichment in the comparison and reference groups, respectively. *P < 0.05, **P < 0.01, ***P < 0.001. ASV, amplicon sequence variant; PC, principal component; PCoA, principal coordinates analysis; IQR, interquartile range; CLR, centered log-ratio; FDR, false discovery rate; PERMANOVA, permutational multivariate analysis of variance. Placebo_T0, baseline samples before radiotherapy with placebo; Placebo_T1, mid-radiotherapy samples with placebo; Placebo_T2, samples at the end of radiotherapy with placebo; *L. reuteri*, *Limosilactobacillus reuteri*; *L. reuteri*_T0, baseline samples before radiotherapy with *L. reuteri* supplementation; *L. reuteri*_T1, mid-radiotherapy samples with *L. reuteri* supplementation; *L. reuteri*_T2, samples at the end of radiotherapy with *L. reuteri* supplementation. Raw sequencing data are available at NGDC under accession code HRA016457.

**Fig. 5:**
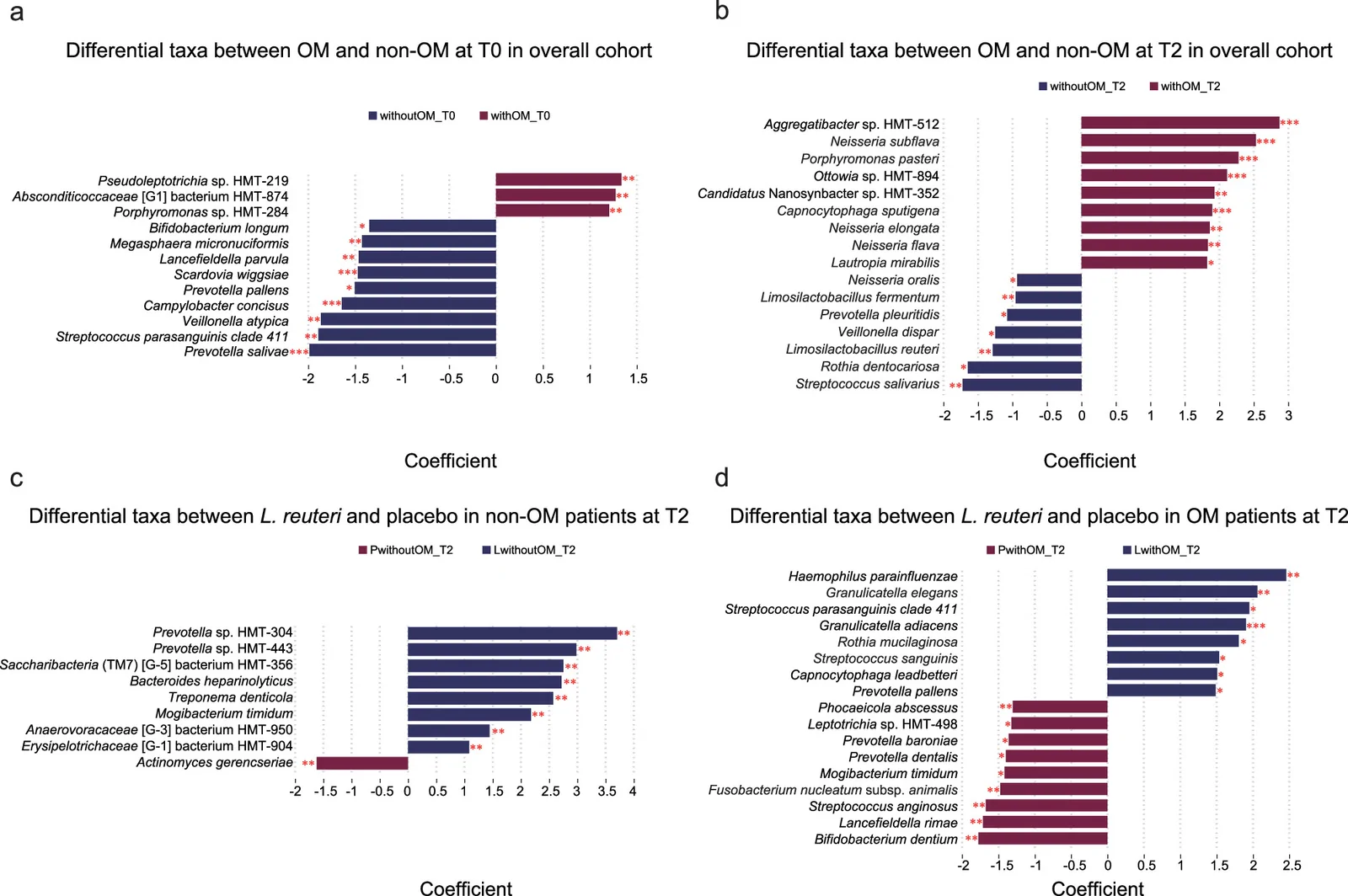
Differential salivary microbiota features associated with oral mucositis and treatment groups. **a**, **b** Differentially abundant taxa between patients with and without oral mucositis (OM) at baseline (T0) and the end of radiotherapy (T2) in the overall cohort. **c**, **d** Differential taxa between the *L. reuteri* and placebo groups among patients without OM and with OM at T2. MaAsLin2 models were fitted with appropriate covariate adjustment, and statistical significance was determined using two-sided tests. Asterisks in the figures denote nominal *P* values, whereas the corresponding nominal *P-*values and FDR-adjusted *P-* values after multiple-testing correction are provided in the Source Data file. Positive and negative coefficients indicate enrichment in the comparison and reference groups, respectively. *P < 0.05, **P < 0.01, ***P < 0.001. withoutOM_T0 (*n* = 46), baseline samples from patients who remained OM-free during radiotherapy; withOM_T0 (*n* = 94), baseline samples from patients who subsequently developed OM; withoutOM_T2 (*n* = 46), patients without OM at the end of radiotherapy; withOM_T2 (*n* = 94), patients with OM at the end of radiotherapy; PwithoutOM_T2 (*n* = 15), patients without OM in the placebo group at the end of radiotherapy; LwithoutOM_T2 (*n* = 31), patients without OM in the *L. reuteri* group at the end of radiotherapy; PwithOM_T2 (*n* = 55), patients with OM in the placebo group at the end of radiotherapy; LwithOM_T2 (*n* = 39), patients with OM in the *L. reuteri* group at the end of radiotherapy. Raw sequencing data are available at NGDC under accession code HRA016457.

As prespecified in the study protocol, a total of 423 saliva samples were collected for 16S rDNA sequencing, of which 420 passed quality control and were retained for analysis. Rarefaction curves confirmed adequate sequencing depth (Supplementary Fig. 7). Analysis of α diversity showed that both the Sobs and Shannon indices remained stable across the assessed time points in the *L. reuteri* group (Fig. 4a, b). For β diversity, principal coordinates analysis demonstrated specific shifts in community structure in the *L. reuteri* group during radiotherapy (*P* = 0.001), while no such change was shown in the placebo group (Fig. 4c, d and Supplementary Fig. 8). Longitudinal differential abundance analysis revealed that radiotherapy induced substantial shifts in the oral microbiome regardless of the intervention. Specifically, several opportunistic pathobionts, including *Fusobacterium nucleatum* subsp. *animalis* and *Prevotella nigrescens*, increased from T0 to T2 in both the placebo and *L. reuteri* groups (Fig. 4g, h). We subsequently evaluated the overall cohort to identify microbial signatures associated with OM clinical status. At T0, participants who remained OM-free throughout the study exhibited higher relative abundances of specific taxa, including *Veillonella atypica* and *Streptococcus parasanguinis* clade 411 (Fig. 5a). By T2, the microbial profiles of patients without OM were characterized by higher abundances of commensals (e.g., *Limosilactobacillus reuteri* and *Rothia dentocariosa*), while patients with OM showed higher abundances of *Porphyromonas pasteri* and *Capnocytophaga sputigena* (Fig. 5b). To further explore the associations of the probiotic intervention, we stratified the T2 cohort by OM status and compared the two treatment arms. In OM-free patients, specific taxonomic variations were observed in the *L.reuteri* group relative to the placebo group (Fig. 5c). Notably, in OM patients, the placebo group showed higher abundance of pathobionts (e.g., *Prevotella dentalis* and *Fusobacterium nucleatum* subsp. *animalis*), whereas the *L. reuteri* group showed higher abundance of oral commensals such as *Rothia mucilaginosa* and *Streptococcus sanguinis* despite inflammation (Fig. 5d). Consistently, microbiome comparisons in the overall cohort stratified by OM status and treatment group aligned with these observations (Supplementary Figs. 9–12). Functionally, PICRUSt2 prediction indicated a higher predicted functional potential for NADH dehydrogenase in the *L. reuteri* group at the end of radiotherapy compared with the placebo group (Supplementary Fig. 13). Collectively, these findings support a potential mechanistic model whereby *L. reuteri* may alleviate RIOM through coordinated modulation of the oral microbiota and related biological pathways (Supplementary Fig. 14).

### Safety

Three patients in the *L. reuteri* group experienced mild-to-moderate gastrointestinal symptoms (diarrhea, abdominal distension, and dyspepsia), while one patient in the placebo group reported dyspepsia, which were considered potentially associated with the study lozenges (Supplementary Table 7). All cases resolved spontaneously without intervention. No severe adverse events (SAEs) linked to *L. reuteri* or placebo were observed. The incidence of radiotherapy-related grade 3-4 adverse events (AEs) was comparable between groups, with 46.3% (37 of 80) in the *L. reuteri* group versus 52.5% (42 of 80) in the placebo group (Supplementary Table 8). AEs exhibited a balanced distribution across groups and aligned with the established radiotherapy toxicities (Supplementary Table 8).

### Tumor Response

Tumor response was additionally evaluated as a post-hoc analysis. Among the enrolled patients, 51.2% (82/160) underwent surgery before radiotherapy, whereas 48.8% (78/160) received definitive radiotherapy and were therefore assessed for tumor response at 3 months after radiotherapy. Among patients who underwent surgery, distant metastasis occurred in one patient in the *L. reuteri* group and two patients in the placebo group, while one patient in each group experienced local recurrence during follow-up. According to RECIST v1.1 criteria, the complete response rates and partial response rates were 89.5% (34/38) and 7.9% (3/38) in the *L. reuteri* group versus 90.0% (36/40) and 5.0% (2/40) in the placebo group among patients treated with definitive radiotherapy (Supplementary Table 9).

## Discussion

This double-blind, randomized clinical trial demonstrated that *L. reuteri* significantly reduced the incidence of SOM, delayed its onset and shortened its duration, while exhibiting favorable safety profiles. In addition, significant improvements in certain quality-of-life scores were observed, along with lower weekly average scores for specific mouth and throat soreness and better oral activity scores in patients receiving radiotherapy for head and neck cancer. Notably, the relatively low analgesic use in our cohort may be partly attributed to traditional stoic attitudes toward pain among Chinese patients^24–26^. Beyond symptom control, the supplementation of *L. reuteri* correlated with a decreased utilization of parenteral nutrition and analgesics relative to placebo. This reduction in high-intensity supportive care implies potential economic benefits through optimized resource utilization. In contrast to our findings, a recent randomized clinical trial found no overall benefit of *L. reuteri* against OM, which may be explained by factors including its sample size and patient population heterogeneity^23^. Notably, subgroup analysis of patients receiving radiotherapy alone demonstrated reduced OM severity, which is consistent with our primary findings. These findings suggest that *L. reuteri* may represent a promising prophylactic strategy to improve supportive care in patients undergoing radiotherapy.

Prior clinical trials have indicated that specific probiotic strains, including *Lactobacillus brevis CD2*, *Streptococcus salivarius K12* (*SsK12*), and probiotic combinations containing *Bifidobacterium longum*, *Lactobacillus lactis*, and *Enterococcus faecium* have demonstrated significant efficacy in reducing RIOM^3,27,28^. Collectively, these convergent beneficial effects across distinct strains raise important questions regarding the mechanistic basis of probiotic protection against RIOM. As proposed by Wardill et al., such conserved efficacy suggests that clinical benefit may not arise exclusively from strain-specific metabolic properties, but rather from the general ability of probiotic microorganisms to occupy ecological niches and competitively restrict pathogen colonization^29^. Notably, *L. reuteri* may extend beyond these shared mechanisms by additionally modulating the host inflammatory microenvironment and preserving mucosal homeostasis, thereby reinforcing its protective role against RIOM^30–32^.

We performed longitudinal profiling of the oral microbiota during radiotherapy to investigate the association between *L. reuteri* supplementation and RIOM. Our findings indicate that *L. reuteri* supplementation was associated with stabilization of the oral microbiota during radiotherapy, characterized by a higher relative abundance of commensal species, a lower relative abundance of potential pathogens, and different profiles of specific metabolic pathways. Specifically, the higher abundances of oral health-associated taxa, including *Rothia mucilaginosa* and *Streptococcus sanguinis,* among patients who developed OM within the *L. reuteri* group at the end of radiotherapy, indicate that *L. reuteri* supplementation may be associated with reduced radiation-induced perturbation of the oral microbial community^33,34^. In contrast, patients with OM receiving a placebo exhibited higher abundances of *Prevotella dentalis* and *Fusobacterium nucleatum*, taxa that are associated with dental caries, periodontal inflammation, and opportunistic pathogenicity in the oral cavity^35–38^. Their enrichment may be associated with further worsening of radiation-induced oral tissue injury. The key predicted functional alteration was the high expression of NADH dehydrogenase in the *L. reuteri* group at the end of radiotherapy. NADH dehydrogenase is a central component of the bacterial electron transport chain, playing a pivotal role in maintaining cellular redox balance and energy homeostasis^39,40^. The predicted upregulation of NADH dehydrogenase pathways in the *L. reuteri* group may suggest an enhanced predicted functional potential of the oral microbiota for scavenging free radicals or maintaining metabolic activity at the end of radiotherapy. Compared with our previous findings on *SsK12*, this result highlights that the effect of *L. reuteri* on energy metabolism pathways may be associated with alleviation of RIOM, thus providing a novel and promising perspective for the management of RIOM. Taken together, these findings support the hypothesis that *L. reuteri* administration may help maintain oral microbial homeostasis, thereby contributing to the management of RIOM.

The study’s strengths include the use of a randomized, double-blind, placebo-controlled design with strict eligibility criteria, which minimizes bias and ensures robust results. In contrast to systemic therapies, the lozenge formulation (sucking rather than swallowing) delivers high local concentrations of *L. reuteri* directly to the oral mucosa. Longitudinal microbiome analysis (T0/T1/T2) elucidated dynamic shifts in bacterial communities linked to RIOM severity, providing a foundation for future mechanistic studies.

This study has several limitations. First, the single-center design and lack of stratified randomization may limit generalizability and introduce potential prognostic imbalances across the heterogeneous tumor sites and variable radiation doses involved. Second, the exclusion of surgical patients receiving perioperative antibiotics precludes an assessment of *L. reuteri*’s antibiotic tolerance, a factor critical for broader clinical implementation. Third, the clinical efficacy was more modest than observed in murine models, likely reflecting translational discrepancies in relative probiotic dosing and biologically effective radiation doses. In addition, despite statistical adjustment, the baseline divergence in the oral microbiota between groups may still confound the interpretation of the intervention’s independent effects. Furthermore, the proportional hazards assumption was violated for onset endpoints; thus, the corresponding overall hazard ratios may not be statistically valid as standalone effect estimates. Moreover, as both the species-level annotations from 16S V3-V4 and the PICRUSt2 functional prediction are putative and no mechanistic, functional, or causal link between specific microbial changes and clinical outcomes has been demonstrated, the results of the oral microbiota should be interpreted cautiously. Finally, the distinct etiological profile of our cohort, characterized by a high prevalence of EBV and low rates of tobacco and alcohol use, may not fully represent the HPV-dominant or lifestyle-associated head and neck cancer populations typical of Western settings^41^. Future multicenter trials are warranted to validate these findings across more diverse clinical and demographic landscapes.

In conclusion, this phase II randomized clinical trial revealed that *L. reuteri* probiotic lozenges markedly reduced the incidence of SOM, delayed its onset, and shortened its duration among patients receiving radiotherapy for head and neck cancer. By supplementing with *L. reuteri*, this microbial intervention represents a promising strategy for RIOM management. The results of this trial highlight the potential of probiotic-based approaches in alleviating RIOM.

## Methods

### Design of basic research

We assessed the efficacy of *L. reuteri* in the murine model of RIOM. All animal experiments were approved by the Institutional Animal Care and Use Committee of West China Hospital of Sichuan University (approval no. 20230923070) and performed in accordance with the National Institutes of Health Guide for the Care and Use of Laboratory Animals.

Twenty-one healthy male C57BL/6 mice were used and randomly divided into three groups: Control, Saline + irradiation (IR), and *L. reuteri* + IR (*n* = 7/group). On Day 0 (D0), the Saline + IR and *L. reuteri* + IR groups received 18 Gy X-ray IR to the heads. From the initiation of IR to 7 days post-IR, mice were administered twice daily with either oral saline or *L. reuteri* (2 × 10^8^ CFU/dose), followed by 30 minutes fasting after each administration. At the experimental endpoint (D8), all animals were euthanized to prevent their body weight loss from exceeding the 20% limit stipulated by ethical guidelines^42,43^, and each group was further randomly divided into two subgroups for macroscopic (*n* = 4 per subgroup) and histopathological (*n* = 3 per subgroup) assessment. For macroscopic evaluation, whole tongue tissues were stained with 0.05% toluidine blue (10 min) and destained with 10% acetic acid (1 min). Ulcer area percentage was quantified using ImageJ software. For histopathological analysis, tongue tissues were fixed in 4% paraformaldehyde, paraffin-embedded, and sectioned for hematoxylin and eosin (H&E) staining. Three non-consecutive H&E-stained sections per sample (3 samples/group) were randomly selected, and epithelial thickness was measured at two representative sites per section. Basal cell density was assessed by counting the absolute number of basal layer cells.

Results are presented as means ± SD. Statistical significance was calculated using the one-way ANOVA test for comparisons among multiple groups, while intergroup comparisons between two groups were analyzed with Student’s *t* test.

### Study design and participants

This randomized, double-blind, placebo-controlled trial involving human participants was conducted at West China Hospital of Sichuan University between 1 March 2024 and 1 January 2025. The study protocol (available in Supplementary Information) was approved by the Institutional Review Board of West China Hospital, Sichuan University (No. 2023-1801), and the study was conducted in accordance with all relevant ethical regulations and the principles of the Declaration of Helsinki. This trial was prospectively registered at ClinicalTrials.gov (NCT06285591) on 29 February 2024. Written informed consent was obtained from all participants before initiation of the intervention. This clinical trial followed the Consolidated Standards of Reporting Trials (CONSORT) reporting guideline.

Eligible patients were pathologically confirmed head and neck cancer patients without distant metastasis, aged 18 to 80 years, with an Eastern Cooperative Oncology Group (ECOG) performance status ≤ 2, and with a planned radiotherapy regimen (60–72 Gy) alone or with chemotherapy. Exclusion criteria included patients with a known allergy to probiotics or a history of severe allergic reactions; use of antibiotics, antifungal drugs, or antimicrobial mouthwash within 1 month before study enrollment; poor oral hygiene (a simplified oral hygiene index score of ≥ 3), severe periodontal disease, or a history of prior head and neck radiotherapy; and any other condition deemed by the investigator to be incompatible with study participation (e.g., pregnancy, lactation, severe comorbidities, or significant psychiatric conditions).

### Randomization and masking

The patients were randomly assigned in a 1:1 ratio to the *L. reuteri* probiotic group or the placebo group, using a computer-generated randomization list with a permuted block design. An independent pharmacist packaged *L. reuteri* and placebo lozenges into identical containers labeled with randomization codes assigned by the trial’s statistical team to maintain blinding. The *L. reuteri* and placebo lozenges were identical in appearance, taste, and color (Supplementary Fig. 15). The randomization details, including the assignment order, block size, and random numbers, were kept in sealed, opaque envelopes and stored at the Clinical Trial Center of West China Hospital, Sichuan University. Participants and investigators were blinded to the treatment allocation throughout the study.

### Procedures

Prior to radiotherapy initiation, all patients received oral hygiene guidance, underwent baseline oral examinations, and received customized dental interventions as needed, including tooth fillings, endodontic therapy, removal of non-restorable teeth, and nonsurgical periodontal therapy. Definitive or postoperative adjuvant radiotherapy was administered to all patients at 1.8–2.2 Gy daily for 6–6.5 weeks, totaling a cumulative dose of 60–72 Gy, and all radiotherapy was delivered using volumetric modulated arc therapy (VMAT) technology. Delineation of clinical target volumes and organs at risk followed established international contouring standards^44,45^. Contouring of the oral cavity followed the criteria described by Mir et al.^46^. Cisplatin (100 mg/m²) was given as concurrent chemotherapy every 3 weeks.

The *L. reuteri* lozenges (strains DSM 17938 and ATCC PTA 5289) contained 2 × 10⁸ live bacteria per unit. The placebo lozenges shared the same formulation as the *L. reuteri* lozenges but without the probiotic, whose appearance, taste, and color were identical to those of the *L. reuteri* lozenges. During radiotherapy, patients were directed to suck either *L. reuteri* or placebo lozenges twice daily, with specific guidance to avoid eating, drinking, or engaging in oral hygiene practices for at least 1 hour after administration. Standard supportive measures, including oral rinsing (pure water), analgesic use, and nutritional supplementation, remained allowable throughout radiotherapy, while other oral agents, probiotics, antimicrobial products (such as antibiotics or antifungal agents), topical mouthwashes other than pure water (such as antibacterial mouthwashes or baking soda solutions), and any other experimental supportive interventions were prohibited.

Study lozenges were dispensed weekly during radiotherapy. At each scheduled weekly follow-up visit, participants received a new supply of lozenges and were required to return the container from the last week to allow direct quantitative counting of unused lozenges for adherence assessment. Furthermore, all participants were provided with a standardized medication diary (see Supplementary information) to self-record the exact timing and number of lozenges taken, any missed doses, and any AEs encountered during the study. During the weekly visits, trained study personnel jointly reviewed the completed medication diaries with the participants and cross-verified self-reported adherence data against the physical count of remaining lozenges from the returned containers. Treatment adherence was calculated as the ratio of the actual number of lozenges used to the prescribed number of lozenges during radiotherapy.

### Outcomes

The incidence of SOM was the primary endpoint. Secondary endpoints included the onset and duration of SOM, as well as the incidence, onset, and duration of any-grade OM. The exploratory endpoints included quality of life, mouth and throat soreness score, hyposalivation, gustatory function, change in weight, use of parenteral nutrition and analgesics, radiotherapy interruption rate, safety, and the composition and dynamics of salivary microbiota. Subgroup and tumor-response analyses were post-hoc.

OM was assessed by trained investigators using the WHO Oral Toxicity Scale (Supplementary Table 10), with evaluations conducted twice weekly during radiotherapy and once weekly after radiotherapy for up to 8 weeks. At each scheduled visit, patients were systematically asked about any worsening of symptoms since the last assessment, so as to allow for retrospective documentation of the actual onset time. In addition, all patients were encouraged to contact the blinded investigators immediately via WeChat if their oral symptoms worsened between visits to facilitate real-time evaluation. The exact date of SOM/OM onset was further verified by cross-referencing patient recall during follow-up visits with the corresponding WeChat communication records, where possible. Throughout radiotherapy, patients self-reported mouth and throat soreness severity and its impact on daily activities using the Weekly Oral Mucositis Questionnaire. To standardize data collection, all investigators completed Good Clinical Practice certification from China’s National Medical Products Administration prior to study initiation, with training including standardized assessment protocols, uniform oral examination techniques, and systematic data recording procedures. Accuracy and consistency of WHO scores were evaluated within 24 h. Two independent blinded evaluators assessed all efficacy endpoints, with any discordance ( ≥ 1-grade difference in OM scoring) reviewed by a third senior assessor. Inconsistent assessments were flagged for consensus discussion.

Quality of life was evaluated using the European Organization for Research and Treatment of Cancer Quality-of-life Questionnaire Core 30 and Head and Neck Cancer Module at pre-radiotherapy, mid-radiotherapy, end of radiotherapy, and 1, 2, 3 months post-radiotherapy. Hyposalivation was evaluated using the National Cancer Institute Common Terminology Criteria for Adverse Events (NCI-CTCAE v5.0), incorporating both unstimulated saliva flow measurements and patient-reported dry mouth symptoms (Supplementary Table 11). Gustatory function was assessed using taste strips infused with four primary taste modalities (sweet, salty, sour, and bitter) at four different concentrations^47^. Both assessments were performed at pre-radiotherapy, mid-radiotherapy, end of radiotherapy, and 1, 3 months post-radiotherapy. Other clinical parameters, including weight change, use of analgesics, use of parenteral nutrition, and radiotherapy interruptions, were systematically recorded and analyzed. All AEs were graded according to CTCAE v5.0.

### Saliva collection and bioinformatics

We collected saliva samples at three time points: T0 (baseline), T1 (mid-radiotherapy, usually week 3), and T2 (at the end of radiotherapy, usually week 6 or 6.5). For patients with xerostomia, saliva was collected using a cotton swab absorption method^48,49^. Sterile cotton swabs were pre-weighed and subsequently placed in the patient’s oral cavity for 5 min to allow for maximal saliva absorption, after which the swabs were removed and weighed again. All samples were immediately frozen at − 20 °C and transferred to − 80 °C for long-term storage until further analysis. After completion of the analysis, all remaining saliva samples were destroyed.

Bacterial genomic DNA was extracted using the FastPure DNA Isolation Kit (MJYH, Shanghai, China) according to the manufacturer’s protocols, with subsequent amplification of the 16S rDNA gene V3-V4 hypervariable regions using primer pair 338 F(5′-ACTCCTACGGGAGGCAGCAG-3′) /806 R(5′GGACTACHVGGGTWTCTAAT-3′) for high-throughput sequencing. Following sequencing, raw sequencing reads were processed to generate amplicon sequence variants (ASVs) using the DADA2 plugin in QIIME2^50,51^. The ASV table was rarefied to an even depth of 26,080 reads per sample for α diversity analysis. The original non-rarefied ASV table was used for β diversity analysis (Aitchison distance based on centered log-ratio transformation), Kruskal-Wallis comparative tests, and multivariate association analysis using MaAsLin2. Taxonomic assignment of ASVs was performed using BLASTn^51^ (version 2.12.0) against the Human Oral Microbiome Database (HOMD, version 15.22), with a minimum identity threshold of 98% and query coverage of 90% to classify at the species level. A blast-best-hit approach was adopted for annotation, with tie-breaking criteria applied in the following priority order: (1) smallest E-value (most significant), (2) highest percent identity if E-values were equal, (3) highest query coverage if both E-value and identity were equal, and (4) if still tied, the entry with the highest taxonomic rank and the most complete annotation information was selected. All bioinformatics analyses were conducted on the Majorbio Cloud Platform (Majorbio, Shanghai, China; available at: www.majorbio.com).

Rarefaction curves and α diversity metrics, including Sobs and Shannon indices, were assessed using Mothur v1.30.1. Linear mixed-effects models were used to assess the differences in α diversity indices (Shannon and Sobs) between the *L. reuteri* and placebo groups over time. β diversity was assessed using Aitchison distances, calculated from centered log-ratio transformed taxon counts to account for the compositional nature of relative abundance data. Principal coordinate analysis was applied to visualize shifts in microbial community structure and statistically compared using PERMANOVA. All analyses were conducted in R v4.4.0. Kruskal-Wallis rank sum tests were performed at the species level to identify differentially abundant taxa across groups, with unclassified taxa excluded prior to analysis. To further account for potential confounding factors, we applied multivariable association testing using MaAsLin2, including age, sex, BMI, smoking, and alcohol use as fixed effects, and subject as a random intercept for repeated measurements. *P-*values from all analyses were adjusted for multiple testing using the Benjamini-Hochberg false discovery rate (FDR) method, with statistical significance defined as adjusted *P* < 0.05. Functional profiling of microbial communities was performed using PICRUSt2 (v2.2.0-b).

### Sample size and statistical analysis

Based on previous studies and clinical practice, about 65% of head and neck cancer patients developed SOM during radiotherapy^52^. We anticipated that administration of *L. reuteri* would reduce SOM incidence by an absolute margin of 25% (from 65% to 40%). Accordingly, a sample size of 142 patients (71 per group) was required, calculated using PASS 2021 (version 21.0.3) with a two-sided α level of 0.05, 80% power, and 1:1 group allocation. After accounting for a 10% dropout rate and considerations for randomization balance, the final sample size was set at 160 patients (80 per group).

Efficacy analyses were performed both on the ITT population, which included all randomized patients, and on the PP population, defined as patients who completed the study. The primary analysis presented in the manuscript was based on the ITT population. The corresponding analysis for the PP population was provided in the Supplementary information. Primary and secondary endpoints were analyzed in the ITT population. Other clinical parameters, including quality of life, the mouth and throat soreness score, hyposalivation, the gustatory function, change in weight, use of parenteral nutrition and analgesics, radiotherapy interruption rate, were analyzed in the PP population. For all endpoints, analyses were restricted to cases with complete data without imputation. Specifically, for patients who withdrew from the intervention, we continued to collect data on OM-related endpoints to best reflect the clinical course, whereas no further data collection was performed for other endpoints. Bonferroni correction was applied for primary and secondary outcome analyses, with a corrected significance threshold of *P* < 0.0083 (0.05/6). For the safety analyses, patients who received at least one dose of the study medication were included.

To analyze longitudinal changes in questionnaire scores across follow-up time points, a mixed-effects model was employed. This model included fixed effects (treatment group, time, and group × time interaction) and random effects (subject), accounting for the correlation structure of repeated measurements. The model estimated predicted group means, mean differences, and corresponding *P-*values.

Continuous variables following a normal distribution were presented as mean ± SD, while those with a non-normal distribution were presented as median (IQR). Categorical variables were presented as frequencies and percentages. Continuous variables were analyzed using the *t* test (parametric) or Wilcoxon rank-sum test (non-parametric), while categorical variables were compared with χ² or Fisher’s exact tests, with statistical significance set at *P* < .05. For primary and secondary endpoints, the threshold for statistical significance was set to *P* < 0.0083 following Bonferroni correction. No multiplicity adjustment was applied to exploratory analyses; results with *P* < 0.05 were deemed nominally significant and interpreted with caution. Statistical analyses were performed using R software, version 4.4.0 (RStudio, PBC, Boston, MA, USA).

### Unadjusted primary analysis

The incidence of SOM was compared using the χ² test between the *L. reuteri* and placebo groups. The treatment effect was expressed as the relative risk (RR) with its 95% CI, calculated using the Katz logarithmic method. The time to the onset of SOM and OM was analyzed using the Kaplan-Meier method, with differences between groups compared via log-rank tests. Hazard ratios (HRs) and associated 95% CIs were estimated using a Cox proportional hazards model. Patients who did not develop SOM/OM during the study period were censored at the date of their last clinical assessment. The duration of SOM was calculated as the number of days from the first occurrence of SOM to the first instance of non-SOM, without subsequent SOM. Comparisons of duration were performed using a two-part model (incorporating both the probability of occurrence and the conditional duration).

RR is defined as:

$${{\rm{RR}}}=\frac{{p}_{1}}{{p}_{2}}=\frac{a/(a+b)}{c/(c+d)}$$

Where ${p}_{1}$ and ${p}_{2}$ are the incidence proportions in the *L. reuteri* and placebo groups, respectively, and ${a},b,c,{d}$ are the cell counts from the 2 × 2 contingency table (with a and c representing the number of events in each group).

### Adjusted multivariable models (Model 1)

To further evaluate the efficacy of *L. reuteri* while accounting for clinical heterogeneity, we employed multivariable regression models that adjusted for key baseline covariates (Model 1): oral cavity radiation dose (continuous), primary tumor site (categorical), and receipt of concurrent chemotherapy (categorical)^53^. The incidence of SOM/OM was analyzed using modified Poisson regression with robust standard errors, whereas time-to-onset endpoints were analyzed using Cox regression, with the placebo group as the reference category. The duration of SOM/OM was analyzed using a two-part approach. The first part utilized multivariable logistic regression to model the probability of SOM/OM occurrence. In the second part, among patients who developed SOM/OM, the positive duration was modeled using a Gamma generalized linear model with a log link. Adjusted marginal mean durations and between-group differences were derived by combining the predicted probabilities from the logistic component with the conditional mean estimates from the Gamma component. Results for the ITT population were presented in Table 2, while those for the PPS were detailed in Supplementary Table 3.

### Adjusted multivariable models (Model 2)

To ensure the robustness of our findings, we conducted further adjusted analyses (Model 2). For the ITT population, Model 2 was expanded to include smoking and alcohol consumption history (“Yes” versus “No”) as additional covariates in the modified Poisson regression, Cox models, and two-part models. For these binary variables, duration was expressed in the two-part models as the ratio of mean durations between exposure groups. For the PPS analysis, Model 2 further incorporated the baseline Shannon index and the absolute abundance of *Actinomyces graevenitzii*, alongside smoking and alcohol history, to evaluate the intervention effect while controlling baseline compositional differences in the microbiome. For the Shannon diversity index and the abundance of *Actinomyces graevenitzii* in duration outcome analyses, effect estimates were reported as average marginal effects owing to its continuous nature, representing the mean change in duration per one-unit increase in the index, with all other covariates held constant.

### Subgroup analysis

We have added a forest plot to summarize subgroup analyses of the primary outcome (incidence of SOM) across key clinical subgroups, including tumor site, oral cavity radiotherapy dose, radiotherapy intent (definitive vs. adjuvant), use of concurrent chemotherapy, smoking history, and alcohol history. RRs were estimated using modified Poisson regression with robust standard errors. For HPV status, Fisher’s exact test was used to compare the incidence of the SOM between HPV-positive and HPV-negative groups. The results for the association between HPV status and SOM incidence were provided in Supplementary Table 2.

### Reporting summary

Further information on research design is available in the Nature Portfolio Reporting Summary linked to this article.

## Supplementary information

Supplementary Information

Reporting Summary

Transparent Peer Review file

## Source data

Source Data

## Data Availability

All raw sequencing data generated in this study have been deposited in the National Genomics Data Center (NGDC) under the accession code HRA016457. Accession HRA016457 is under controlled access. Access can be requested through GSA. The de-identified individual patient data and sequencing data are available under restricted access within 3 years after the publication of this paper due to privacy protection and ethical restrictions related to participant confidentiality. Access to de-identified individual patient data (for example, demographic characteristics and radiotherapy-related information) can be obtained upon reasonable academic requests by contacting Professor Xing-chen Peng, the corresponding author (pxx2014@163.com). The researcher will also be required to sign a data access agreement with West China Hospital, Sichuan University. The study protocol is available in the Supplementary Information file. The remaining data are available within the Article, Supplementary information or Source Data file. Source data are provided in this paper.
