# Supplementary material for: Limosilactobacillus reuteri for oral mucositis in patients undergoing radiotherapy for malignant head and neck tumors: a randomized controlled trial
Source: Nat Commun. 2026 Sep 29;17:11226. doi: 10.1038/s41467-026-78088-6 (PMC13720049; doi:10.1038/s41467-026-78088-6)
Supplement: Supplementary file 1 [file 41467_2026_78088_MOESM1_ESM.pdf]

Supplementary Fig. 1. The preclinical validation of the efficacy of *Limosilactobacillus reuteri* in radiation-induced oral mucositis. 2
Supplementary Fig. 2. Subgroup analysis of SOM incidence. 3
Supplementary Fig. 3. Kaplan-Meier plots of time to onset of SOM/OM in *Limosilactobacillus reuteri* and placebo groups. 4
Supplementary Fig. 4. Quality-of-life indexes in the *Limosilactobacillus reuteri* and placebo groups at different assessment time points. 5
Supplementary Fig. 5. Mean scores of mouth and throat soreness and oral activities in the *Limosilactobacillus reuteri* and placebo groups at different assessment time points. 6
Supplementary Fig. 6. Gustatory function scores in the *Limosilactobacillus reuteri* and placebo groups at different assessment time points. 7
Supplementary Fig. 7. Rarefaction curves. 8
Supplementary Fig. 8. The comparison of principal coordinate analysis. 9
Supplementary Fig. 9. Salivary microbiota profiles stratified by subsequent OM development using baseline samples in the overall cohort. 10
Supplementary Fig. 10. Salivary microbiota profiles stratified by OM status in the overall cohort. 11
Supplementary Fig. 11. Comparisons of salivary microbiota between the *Limosilactobacillus reuteri* and placebo groups stratified by OM status at assessed timepoints. 12
Supplementary Fig. 12. Comparative salivary microbiota dynamics stratified by OM status within the placebo and *Limosilactobacillus reuteri* groups. 13
Supplementary Fig. 13. Taxonomic and predicted functional shifts between the *Limosilactobacillus reuteri* and placebo groups. 14
Supplementary Fig. 14. Schematic diagram illustrating the hypothesized mechanism of action of *Limosilactobacillus reuteri*. 15
Supplementary Fig. 15. The probiotic *Limosilactobacillus reuteri* lozenges and placebo lozenges were identical in physical appearance, taste and color. 16
Supplementary Table 1. The characteristics of the excluded patients. 17
Supplementary Table 2. Associations of key confounding factors with treatment efficacy 18
Supplementary Table 3. Efficacy results in per-protocol set. 19
Supplementary Table 4. Results of the piecewise Cox regression analysis for SOM/OM onset in the intention-to-treat population. 20
Supplementary Table 5. Efficacy results in patients with SOM/OM. 21
Supplementary Table 6. The incidence of grade 2 or higher hyposalivation in two groups. 22
Supplementary Table 7. Treatment-related adverse events in the *Limosilactobacillus reuteri* and placebo groups. 23
Supplementary Table 8. Summary of any-grade adverse events in all patients. 24
Supplementary Table 9. Tumor response in patients receiving definitive radiotherapy between *Limosilactobacillus reuteri* group and placebo group. 25
Supplementary Table 10. World Health Organization Oral Toxicity Scale. 26
Supplementary Table 11. Dry mouth scale according to CTCAE version 5.0. 27
Study Protocol 28

Supplementary Fig. 1. The preclinical validation of the efficacy of *Limosilactobacillus reuteri* in radiation-induced oral mucositis.

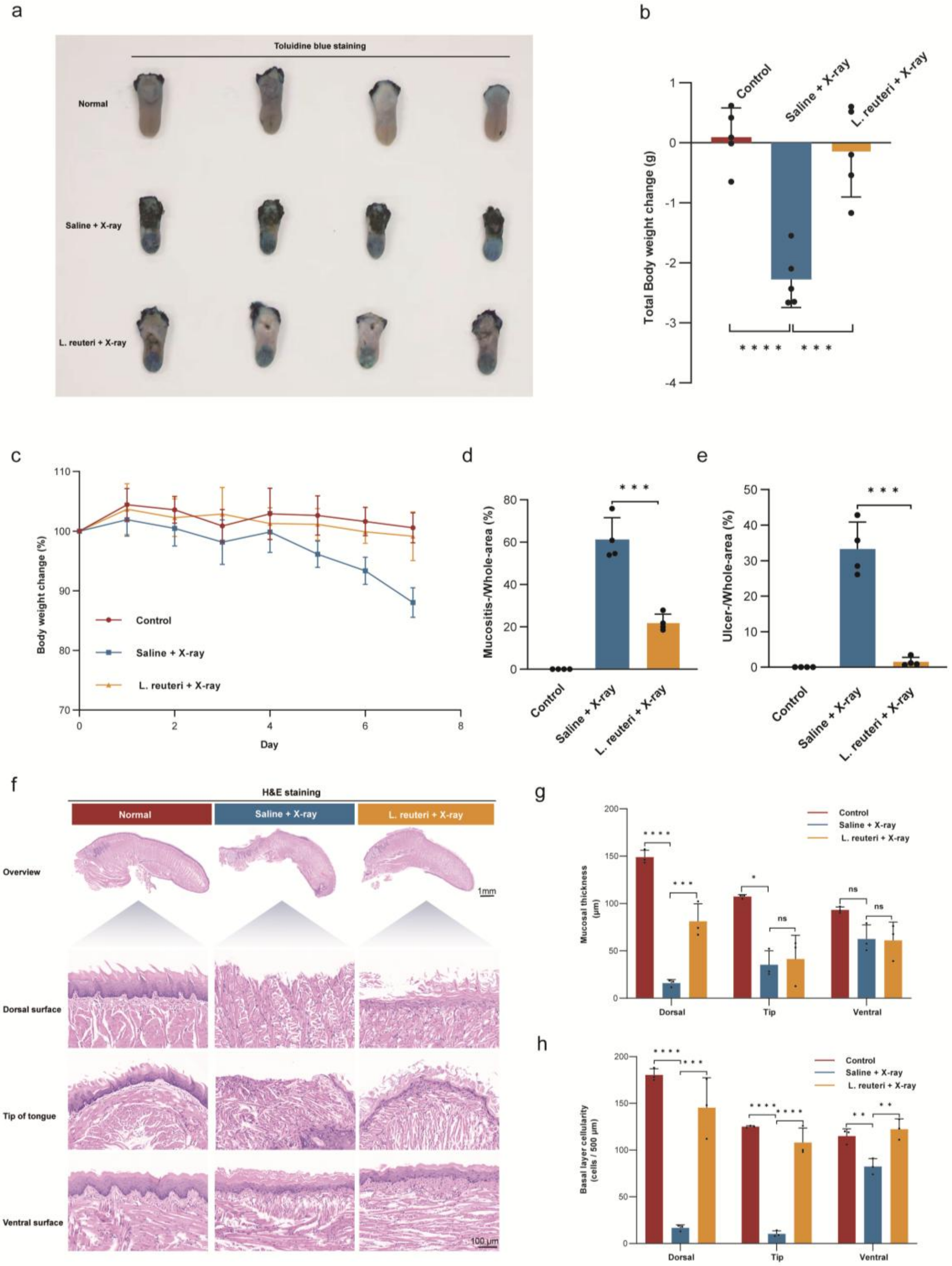

a, Representative photograph of tongues from each group on Day 8 (n= 4 mice per group). It should be acknowledged that the mice were euthanized at this time point per animal ethics criteria, such that the intervention may have delayed but not fully prevented OM onset. **b,** The *L. reuteri* + IR group exhibited significantly attenuated radiation-induced weight loss (n= 5 mice per group). **c,** Weight change trajectories demonstrated slower decline in the *L. reuteri* + IR group compared to the Saline + IR group (n= 5 mice per group). **d-e,** Quantitative analysis showed that *L. reuteri* treatment reduced the percentage of mucositis-affected areas (n= 4 mice per group). **f,** H&E staining revealed improved tongue histopathology. **g-h,** *L. reuteri* significantly attenuated the reduction in dorsal tongue mucosal thickness and increased the density of basal epithelial cells in the dorsal tongue region (n= 3 mice per group). ns, not significant; * $P < .05$; ** $P < .01$; *** $P < .001$; ****$P < .0001$; IR*:* irradiation*; L. reuteri: Limosilactobacillus reuteri.* Source data and exact *P* values are provided as a Source Data file.

Supplementary Fig. 2. Subgroup analysis of SOM incidence.

Subgroup	L. reuteri case/total	Placebo case/total	RR (95% CI)	*P*
Tobacco use (*p for interaction = 0.900*)				
No	17/42	24/37	1.60 (1.04−2.48)	0.034
Yes	13/38	25/43	1.70 (1.02−2.83)	0.041
Alcohol use (*p for interaction = 0.837*)				
No	16/37	32/45	1.64 (1.09−2.49)	0.018
Yes	14/43	17/35	1.49 (0.86−2.58)	0.153
Concurrent chemotherapy (*p for interaction = 0.038*)				
No	24/43	30/46	1.17 (0.83−1.64)	0.369
Yes	6/37	19/34	3.45 (1.56−7.60)	0.002
Treatment type (*p for interaction = 0.079*)				
Definitive	6/38	20/40	3.17 (1.43−7.03)	0.005
Postoperative treatment	24/42	29/40	1.27 (0.92−1.75)	0.150
Oral cavity dose (*p for interaction = 0.657*)				
<40 Gy	10/37	23/40	2.13 (1.18−3.85)	0.013
40−50 Gy	12/27	13/22	1.33 (0.77−2.30)	0.307
>50 Gy	8/16	13/18	1.44 (0.82−2.55)	0.204
Tumor site (*p for interaction = 0.182*)				
Hypopharynx	2/10	6/10	3.00 (0.79−11.44)	0.108
Larynx	5/8	4/7	0.91 (0.40−2.11)	0.834
Nasopharynx	3/24	14/24	4.67 (1.54−14.18)	0.007
Oral cavity	12/22	13/22	1.08 (0.65−1.82)	0.761
Oropharynx	8/13	10/13	1.25 (0.74−2.11)	0.403
Others	0/3	2/4	Not Evaluable	Not Evaluable

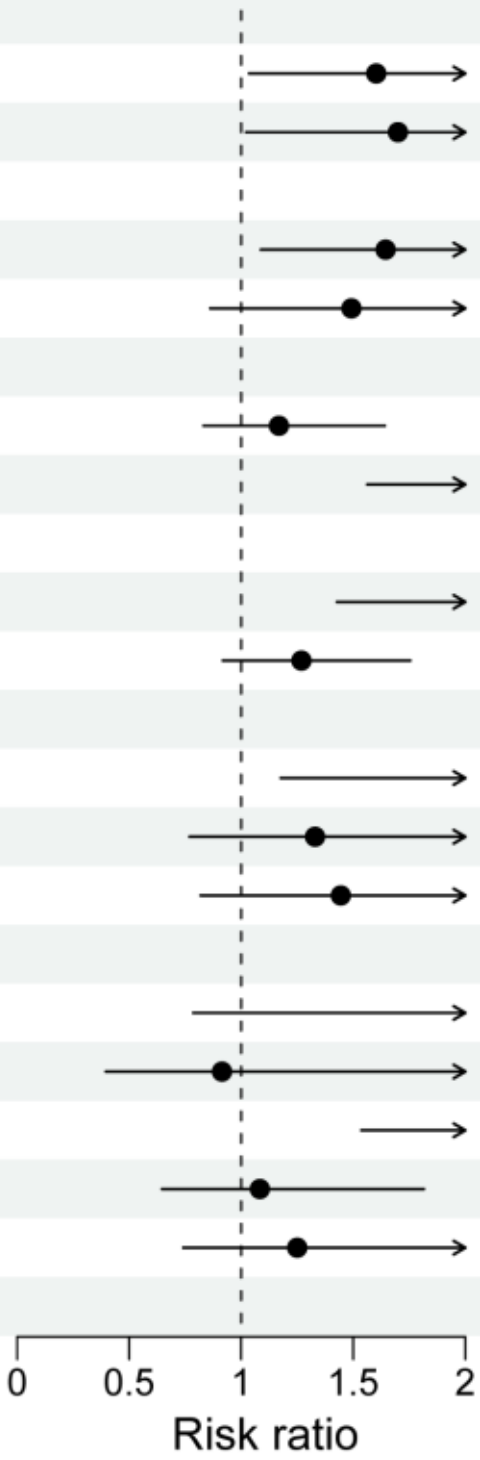

Risk ratios (RRs) and 95% CIs were estimated using modified Poisson regression with robust standard errors. The de-identified data supporting this figure are available from the corresponding author upon reasonable request, subject to patient privacy restrictions (see Data availability statement).

Supplementary Fig. 3. Kaplan-Meier plots of time to onset of SOM/OM in *Limosilactobacillus reuteri* and placebo groups.

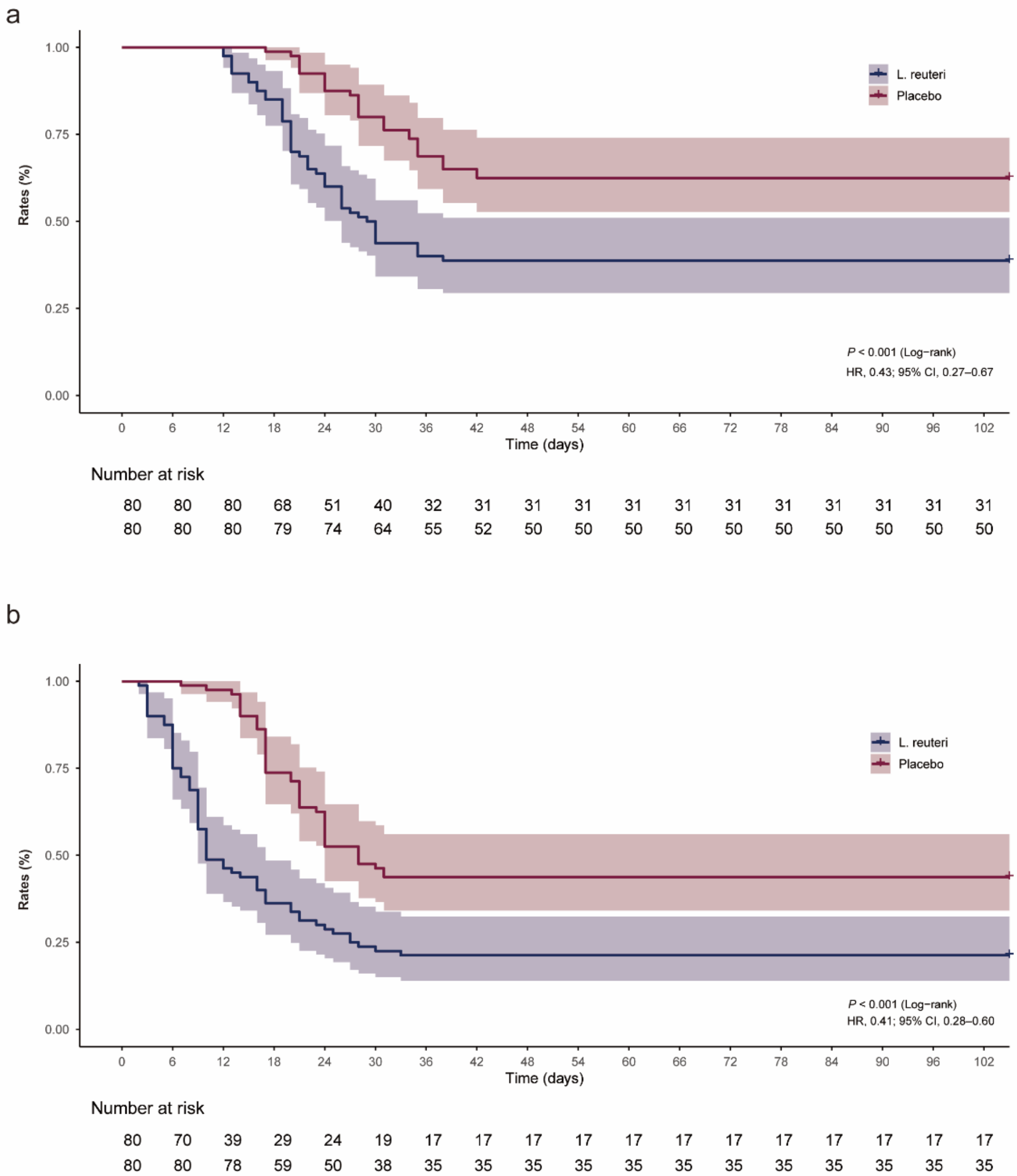

a, Kaplan-Meier plot of time to onset of SOM. **b,** Kaplan-Meier plot of time to onset of OM. Data were analyzed in 80 patients per group. Source data are provided as a Source Data file.

Supplementary Fig. 4. Quality-of-life indexes in the *Limosilactobacillus reuteri* and placebo groups at different assessment time points.

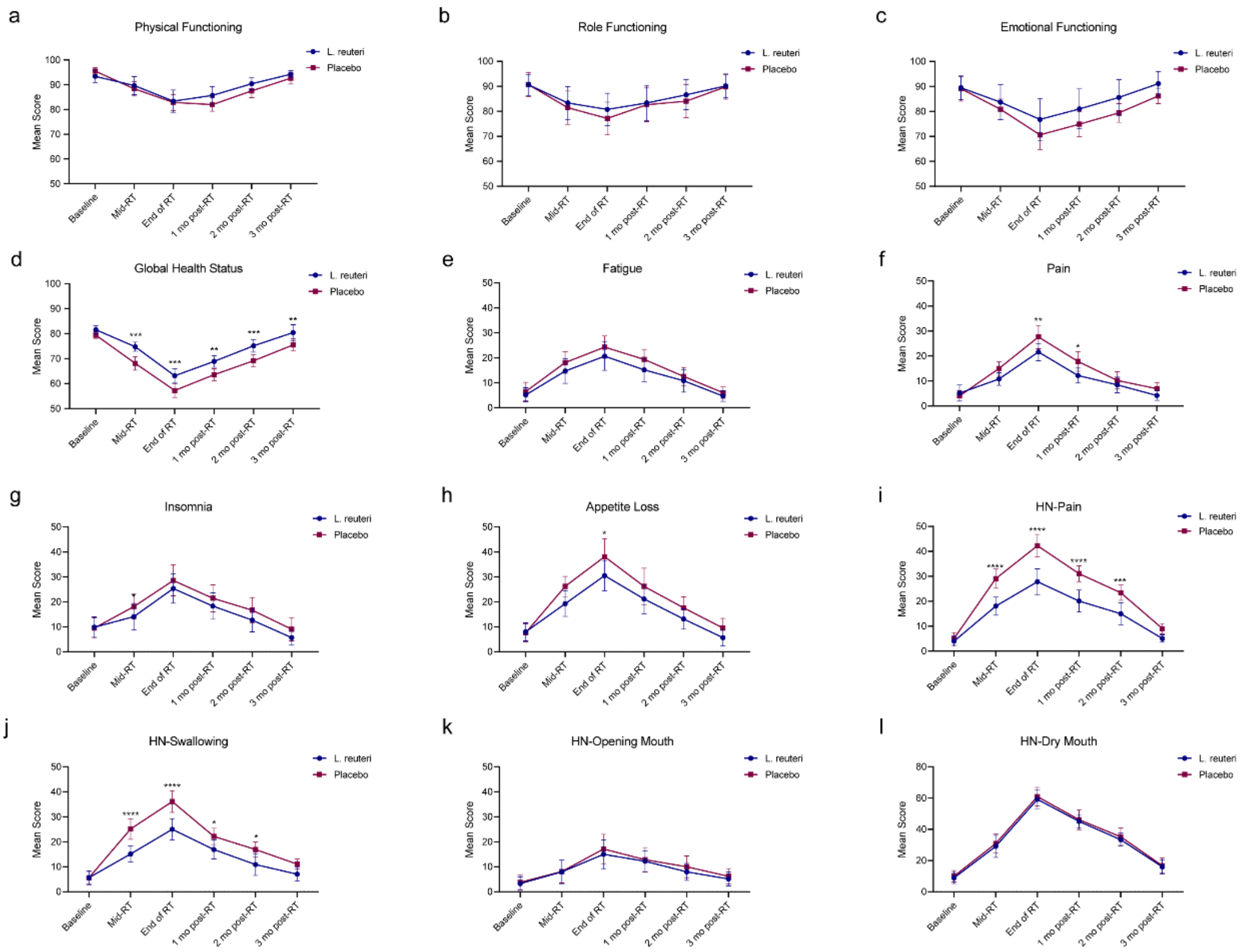

a, Physical Functioning; **b,** Role Functioning; **c,** Emotional Functioning; **d,** Global Health Status; **e,** Fatigue; **f,** Pain; **g,** Insomnia; **h,** Appetite Loss; **i,** HN-Pain; **j,** HN-Swallowing; **k,** HN-Opening Mouth; **l,** HN-Dry Mouth; Sample sizes were n = 71 in the *L. reuteri* group and n = 70 in the placebo group. Source data are provided as a Source Data file.

Supplementary Fig. 5. Mean scores of mouth and throat soreness and oral activities in the *Limosilactobacillus reuteri* and placebo groups at different assessment time points.

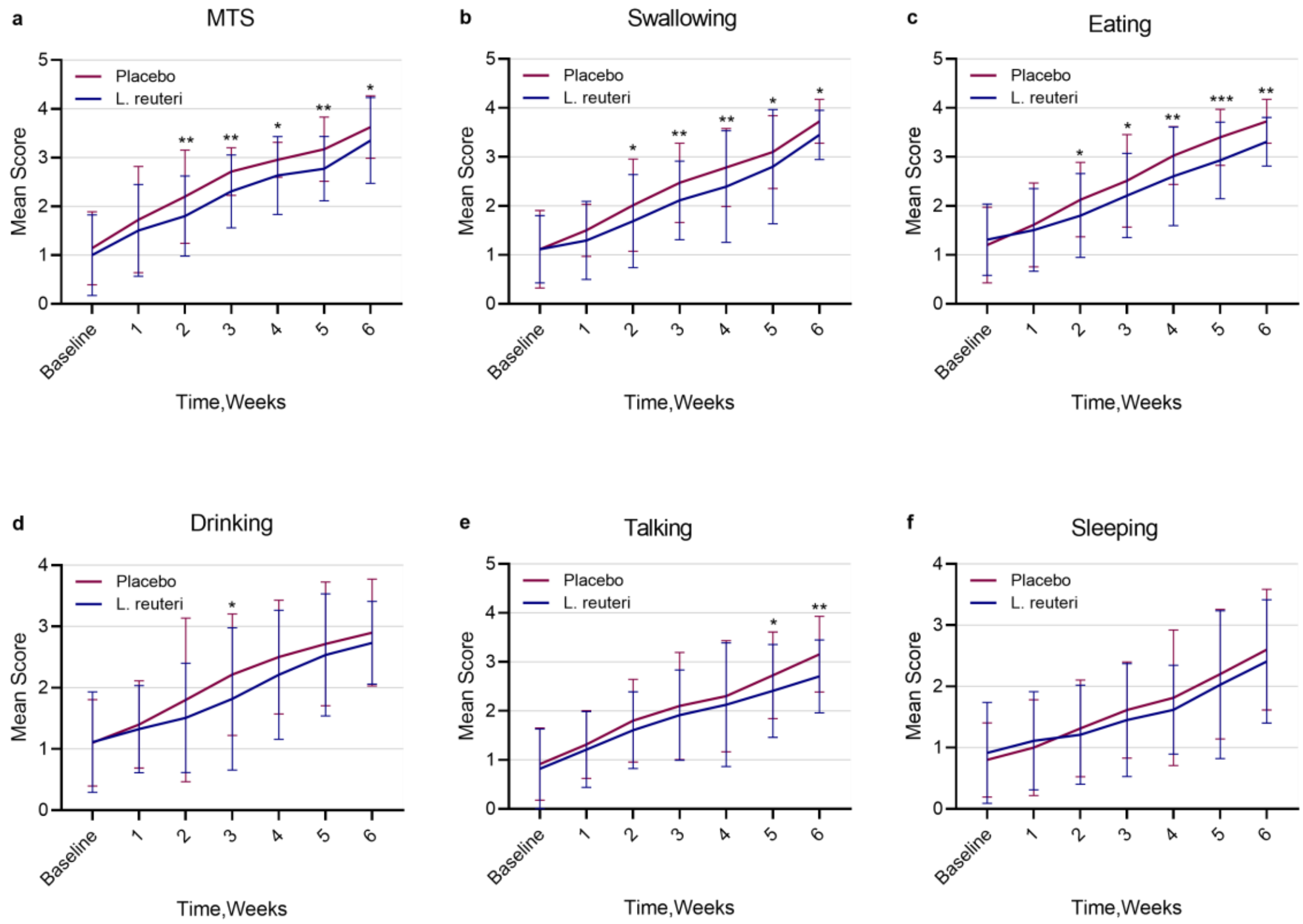

a, Mouth and throat soreness (MTS); **b,** Swallowing; **c,** Eating; **d,** Drinking; **e,** Talking; **f,** Sleeping. During radiotherapy, patients reported MTS scores (Likert scale 1 to 5) and the degree of the impact of MTS on oral activities (including swallowing, drinking, eating, talking, sleeping) (Likert scale 1 to 5) via the Weekly Oral Mucositis Questionnaire (OMWQ). Higher score indicated more severe symptoms. Sample sizes were n = 71 in the *L. reuteri* group and n = 70 in the placebo group. Source data and exact *P* values are provided as a Source Data file.

Supplementary Fig. 6. Gustatory function scores in the *Limosilactobacillus reuteri* and placebo groups at different assessment time points.

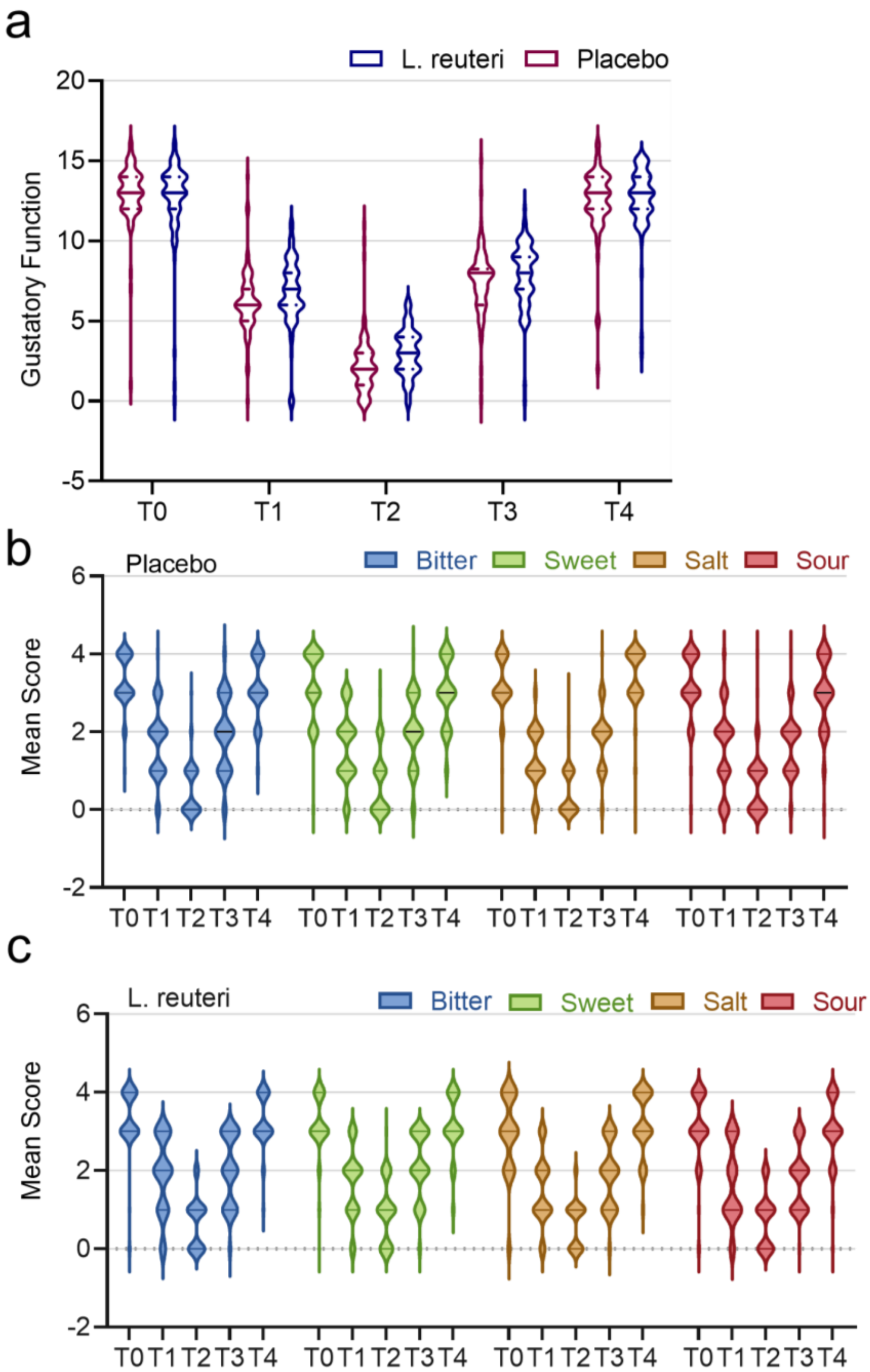

a, Mean scores of gustatory functions in the *Limosilactobacillus reuteri* and placebo group at five time points. **b,** Mean score of the four taste qualities in the placebo group. **c,** Mean score of the four taste qualities in the *Limosilactobacillus reuteri* group. Gustatory function was tested by “taste strip”. Four primary tastes, sweet, sour, salt and bitter have four different concentrations. The correct taste identification was scored as one point. T0, baseline; T1, mid-term radiotherapy (corresponding to the week of 50% fraction completion); T2, end of radiotherapy (the final week of radiotherapy); T3, 1-month post radiotherapy; T4, 3-month post radiotherapy. Sample sizes were n = 71 in the *L. reuteri* group and n = 70 in the placebo group. Source data are provided as a Source Data file.

Supplementary Fig. 7. Rarefaction curves.

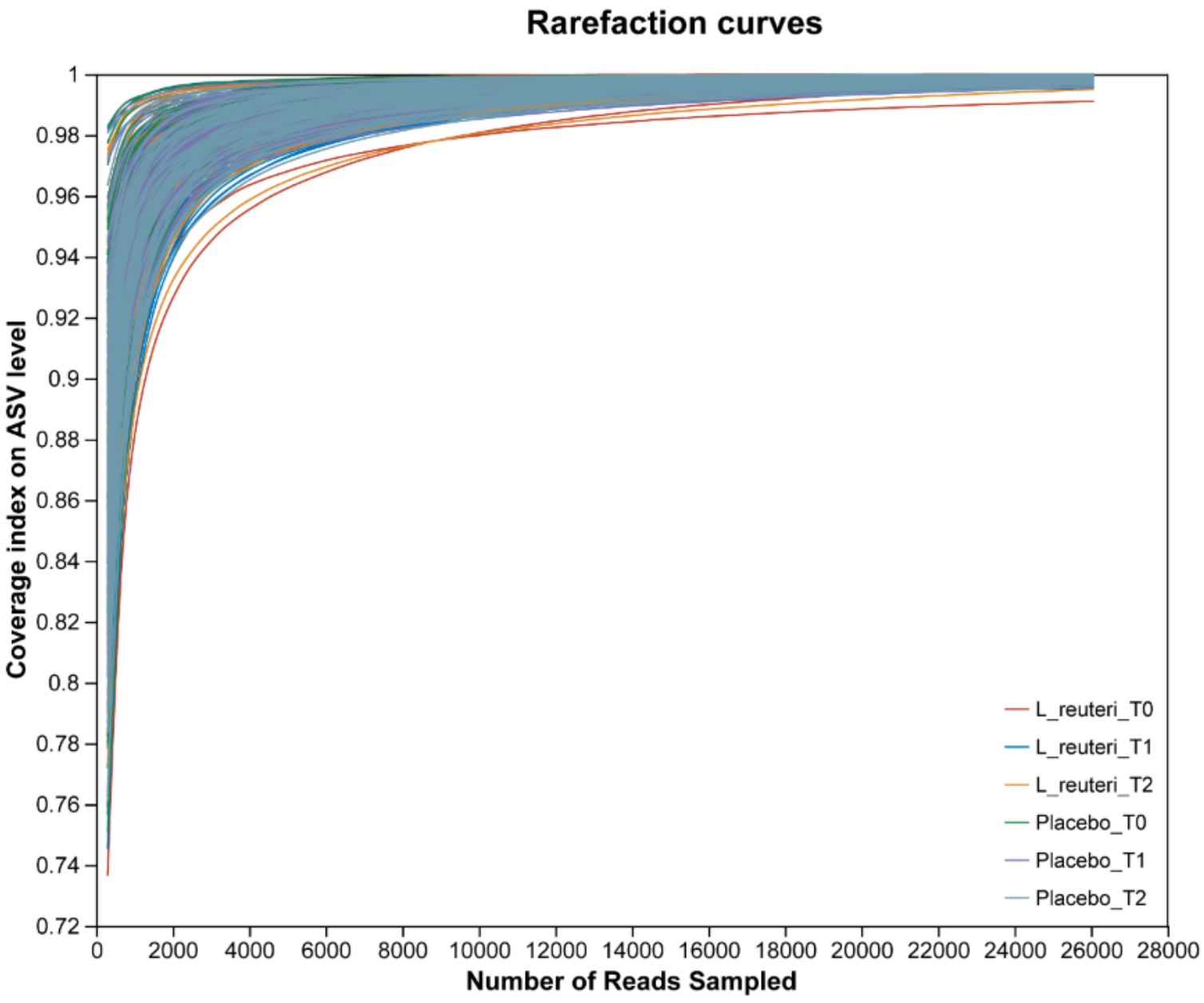

Each group included samples from 70 participants at each time point. Placebo_T0, prior to radiotherapy with a placebo; Placebo_T1, the mid-radiotherapy with a placebo; Placebo_T2, the end of radiotherapy with a placebo; *L. reuteri, Limosilactobacillus reuteri*; *L. reuteri*_T0, prior to radiotherapy with the probiotic *Limosilactobacillus reuteri*; *L. reuteri*_T1, the mid-radiotherapy with the probiotic *Limosilactobacillus reuteri*; *L. reuteri*_T2, the end of radiotherapy with the probiotic *Limosilactobacillus reuteri*. Raw sequencing data are available at NGDC under accession code HRA016457.

Supplementary Fig. 8. The comparison of principal coordinate analysis.

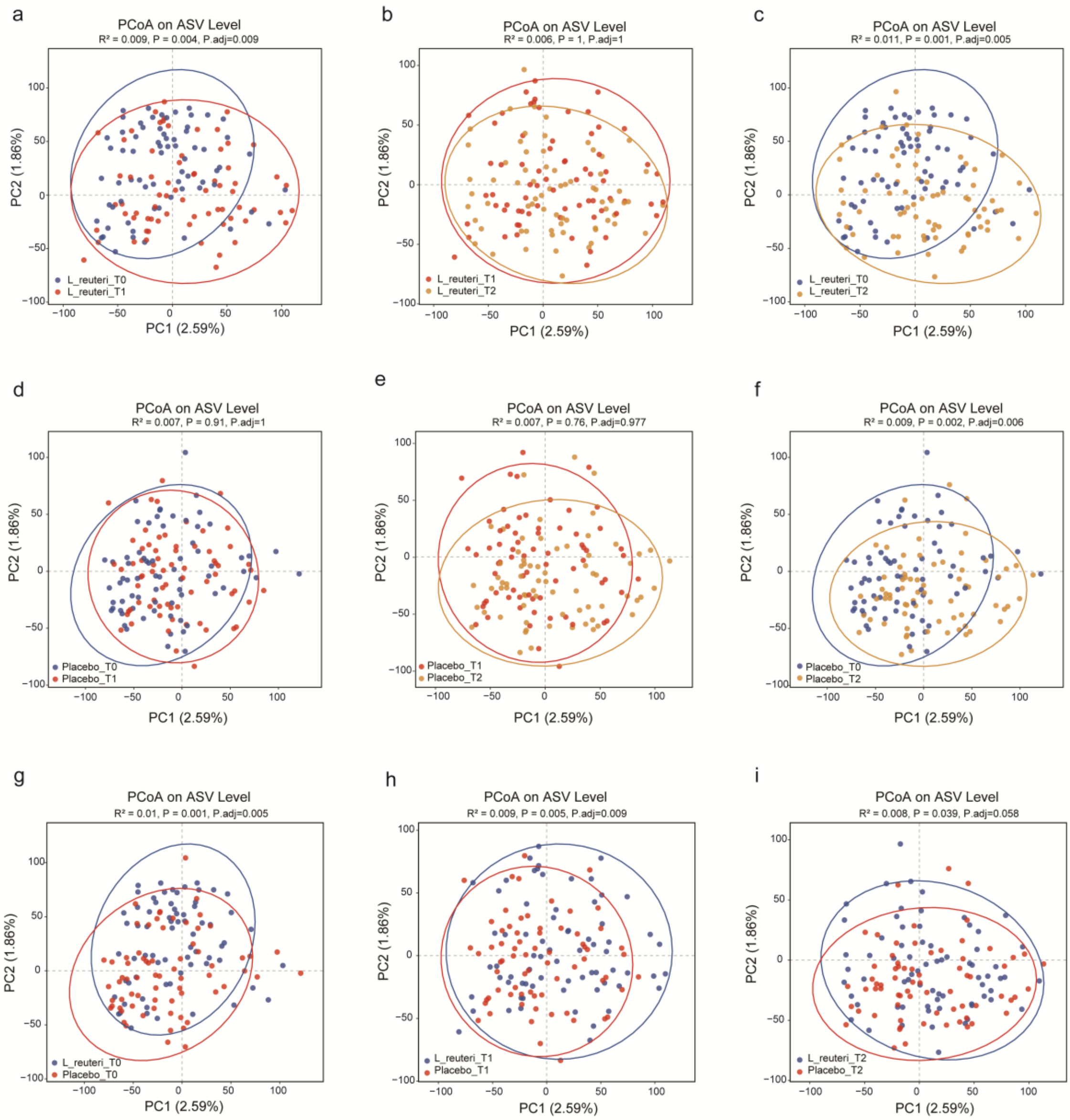

a-c, β diversity of oral microbiota in the *L. reuteri* group at baseline (T0) vs mid-radiotherapy (T1), T1 vs end of radiotherapy (T2), and T0 vs T2; **d-f,** β diversity of oral microbiota in the placebo group at T0 vs T1, T1 vs T2, and T0 vs T2; **g-i,** β diversity of oral microbiota comparing *L. reuteri* and placebo groups at T0, T1, and T2. Each point represents an individual saliva sample from a participant (n = 70 participants per group at each time point). All β diversity analyses were performed using principal coordinates analysis (PCoA) based on Aitchison distances, with statistical significance tested by permutational multivariate analysis of variance (PERMANOVA) and corrected for multiple comparisons using the false discovery rate (FDR) method. Raw sequencing data are available at NGDC under accession code HRA016457.

Supplementary Fig. 9. Salivary microbiota profiles stratified by subsequent OM development using baseline samples in the overall cohort.

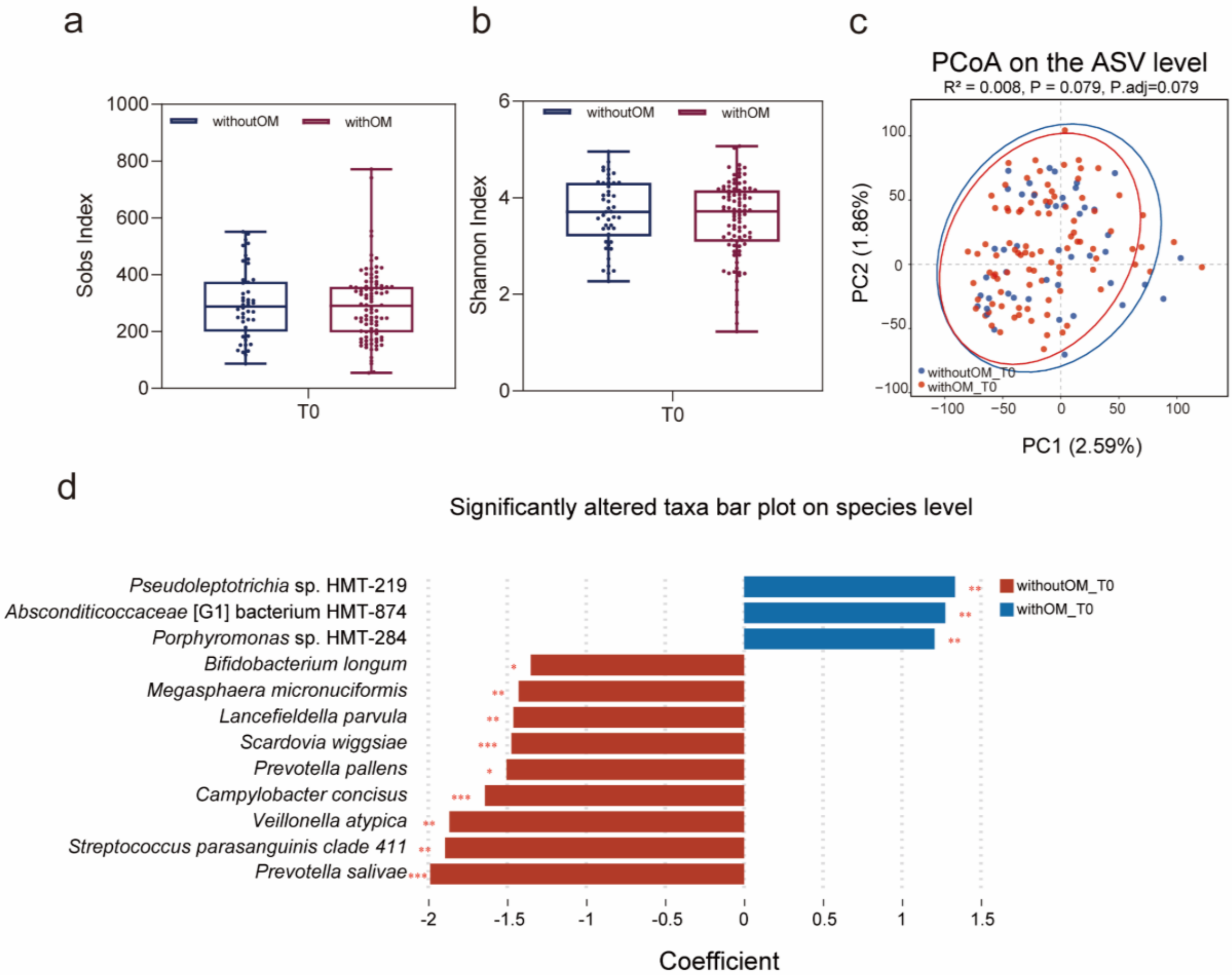

(**a-b**) α diversity: Sobs and Shannon indices comparing salivary microbiota profiles stratified by subsequently OM status at baseline. (**c**) β diversity: PCoA plots based on Aitchison distance at the ASV level illustrating salivary microbiota profiles at baseline, stratified by subsequently OM status. Statistical significance was assessed by PERMANOVA (R^2 and *P* values are indicated), with *P* values adjusted using the FDR correction. (**d**) Significantly differentially abundant bacterial taxa between baseline samples from patients with subsequent OM and those remaining OM-free, identified by MaAsLin2. Positive and negative coefficients indicate enrichment in the comparison and reference groups, respectively. MaAsLin2 models were fitted with appropriate covariate adjustment, and statistical significance was determined using two-sided tests. Asterisks in the figures denote nominal *P* values. **P* < 0.05, ***P* < 0.01, ****P* < 0.001. Abbreviations: OM, oral mucositis; T0, baseline; withOM, patients with oral mucositis; withoutOM, patients without oral mucositis; withOM_T0, baseline samples from patients who subsequently developed OM; withoutOM_T0, baseline samples from patients who remained OM-free; ASV, amplicon sequence variant; PCoA, principal coordinates analysis; FDR, false discovery rate. Raw sequencing data are available at NGDC under accession code HRA016457.

Supplementary Fig. 10. Salivary microbiota profiles stratified by OM status in the overall cohort.

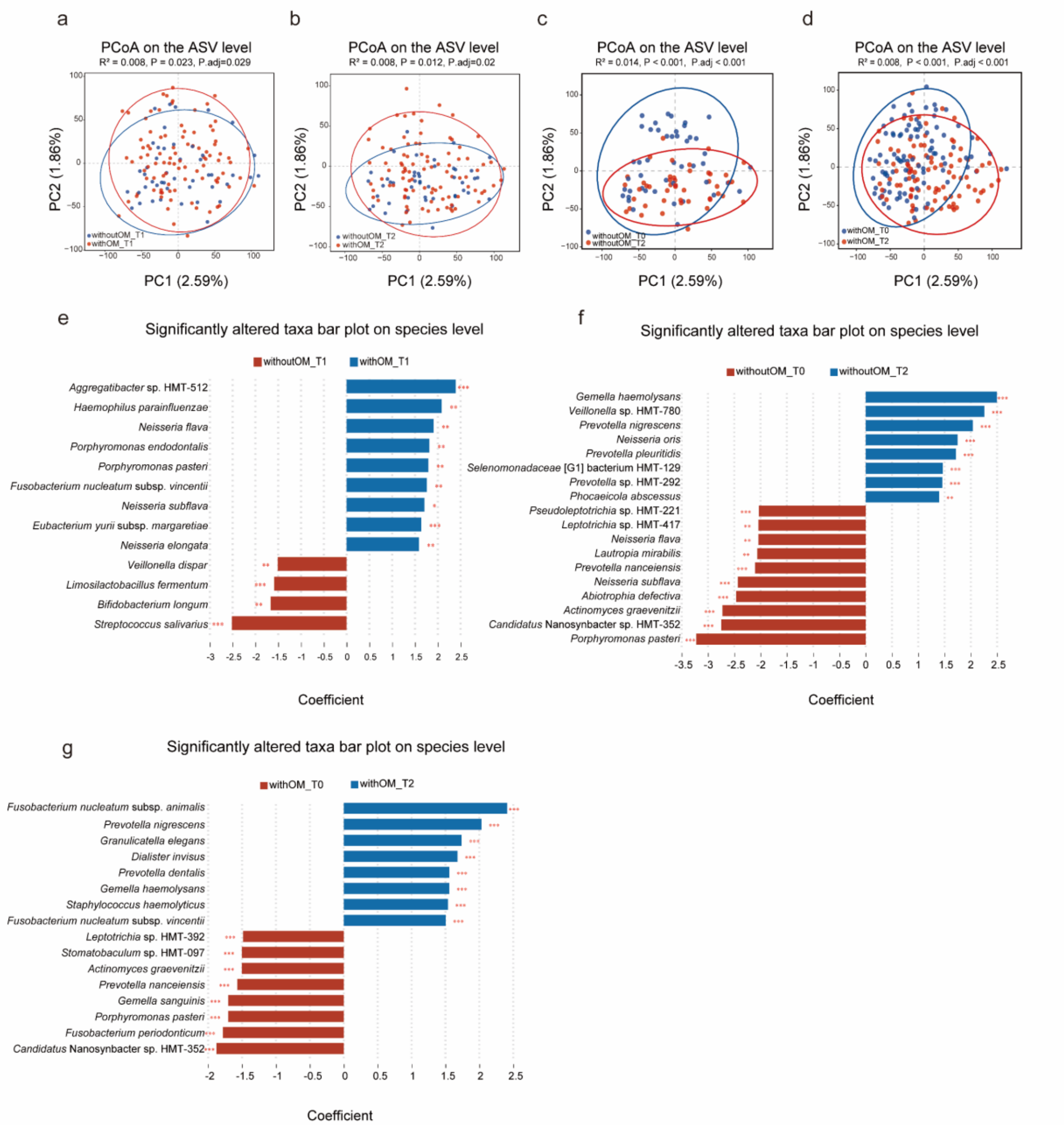

(**a-d**) PCoA plots based on Aitchison distances showing salivary microbial β diversity. Comparisons were presented between patients with and without OM at mid-radiotherapy (**a**) and radiotherapy completion (**b**), and longitudinal changes from baseline (T0) to radiotherapy completion (T2) within the non-OM (**c**) and OM (**d**) groups. R^2 and *P* values were calculated using PERMANOVA, with *P* values adjusted using the FDR correction. (**e-g**) Differentially abundant taxa at the species level identified by MaAsLin2 analysis. Bar plots illustrate significant differences between OM and non-OM groups at T1 (**e**), and shifts from T0 to T2 within the non-OM (**f**) and OM (**g**) groups. T2-associated taxa were presented in Figure 4. Positive and negative coefficients indicate enrichment in the comparison and reference groups, respectively. MaAsLin2 models were fitted with appropriate covariate adjustment, and statistical significance was determined using two-sided tests. Asterisks in the figures denote nominal *P* values. *$P < 0.05$, **$P < 0.01$, ***$P < 0.001$. Abbreviations: OM, oral mucositis; T0, baseline; T1, mid-radiotherapy; T2, end-radiotherapy; withOM_T0, baseline samples from patients who subsequently developed OM; withoutOM_T0, baseline samples from patients who remained OM-free; withOM_T1, patients with oral mucositis at mid-radiotherapy; withoutOM_T1, patients without oral mucositis at mid-radiotherapy; withOM_T2, patients with oral mucositis at end-radiotherapy; withoutOM_T2, patients without oral mucositis at end-radiotherapy; ASV, amplicon sequence variant; PCoA, principal coordinates analysis. Raw sequencing data are available at NGDC under accession code HRA016457.

Supplementary Fig. 11. Comparisons of salivary microbiota between the *Limosilactobacillus reuteri* and placebo groups stratified by OM status at assessed timepoints.

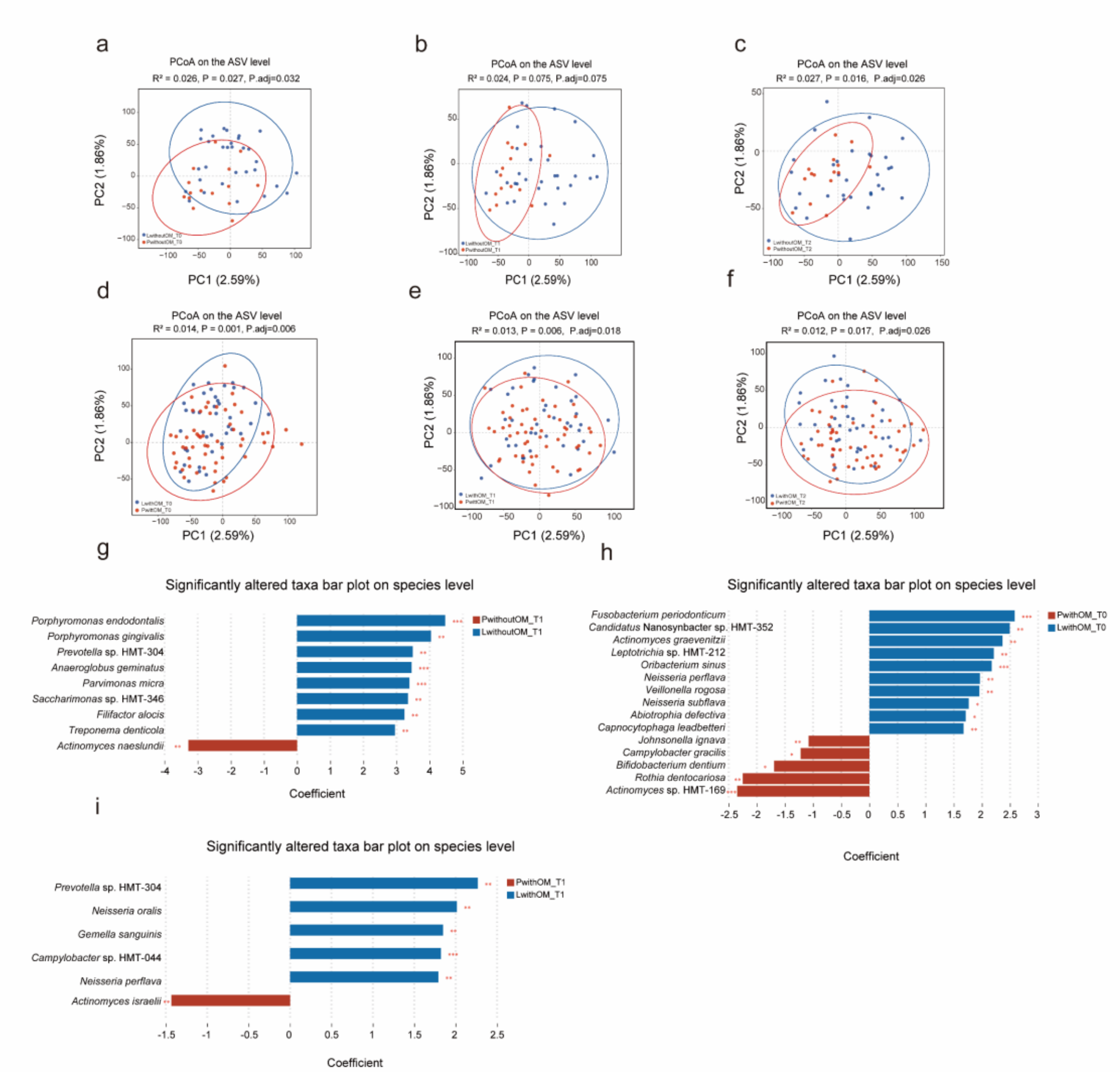

(**a-f**) PCoA plots based on Aitchison distances showing β diversity between the *L. reuteri* and placebo groups. Comparisons were presented for patients without OM at T0 (**a**), T1 (**b**), and T2 (**c**), and for patients with OM at T0 (**d**), T1 (**e**), and T2 (**f**). *P* values were determined by PERMANOVA, with *P* values adjusted using the FDR correction. (**g-i**) Differentially abundant taxa at the species level between the *L. reuteri* and placebo groups identified by MaAsLin2. Bar plots show significant differences in the non-OM group at T1 (**g**), and in the OM group at T0 (**h**) and T1 (**i**). No significant differential taxa were identified at T0 in non-OM patients; T2-associated taxa in OM and non-OM patients were presented in Figure 4. Positive and negative coefficients indicate enrichment in the comparison and reference groups, respectively. MaAsLin2 models were fitted with appropriate covariate adjustment, and statistical significance was determined using two-sided tests. Asterisks in the figures denote nominal *P* values. **P* < 0.05, ***P* < 0.01, ****P* < 0.001. OM, oral mucositis; T0, baseline; T1, mid-radiotherapy; T2, end-radiotherapy; LwithOM_T0, baseline samples from patients in the *L. reuteri* group who subsequently developed OM; LwithoutOM_T0, baseline samples from patients in the *L. reuteri* group who remained OM-free; LwithOM_T1, patients with OM in the *L. reuteri* group at mid-radiotherapy; LwithoutOM_T1, patients without OM in the *L. reuteri* group at mid-radiotherapy; LwithOM_T2, patients with OM in the *L. reuteri* group at radiotherapy completion; LwithoutOM_T2, patients without OM in the *L. reuteri* group at radiotherapy completion; PwithOM_T0, baseline samples from patients in the placebo group who subsequently developed OM; PwithoutOM_T0, baseline samples from patients in the placebo group who remained OM-free; PwithOM_T1, patients with OM in the placebo group at mid-radiotherapy; PwithoutOM_T1, patients without OM in the placebo group at mid-radiotherapy; PwithOM_T2, patients with OM in the placebo group at radiotherapy completion; PwithoutOM_T2, patients without OM in the placebo group at radiotherapy completion; ASV, amplicon sequence variant; PCoA, principal coordinates analysis; *L.reuteri, Limosilactobacillus reuteri*. Raw sequencing data are available at NGDC under accession code HRA016457.

Supplementary Fig. 12. Comparative salivary microbiota dynamics stratified by OM status within the placebo and *Limosilactobacillus reuteri* groups.

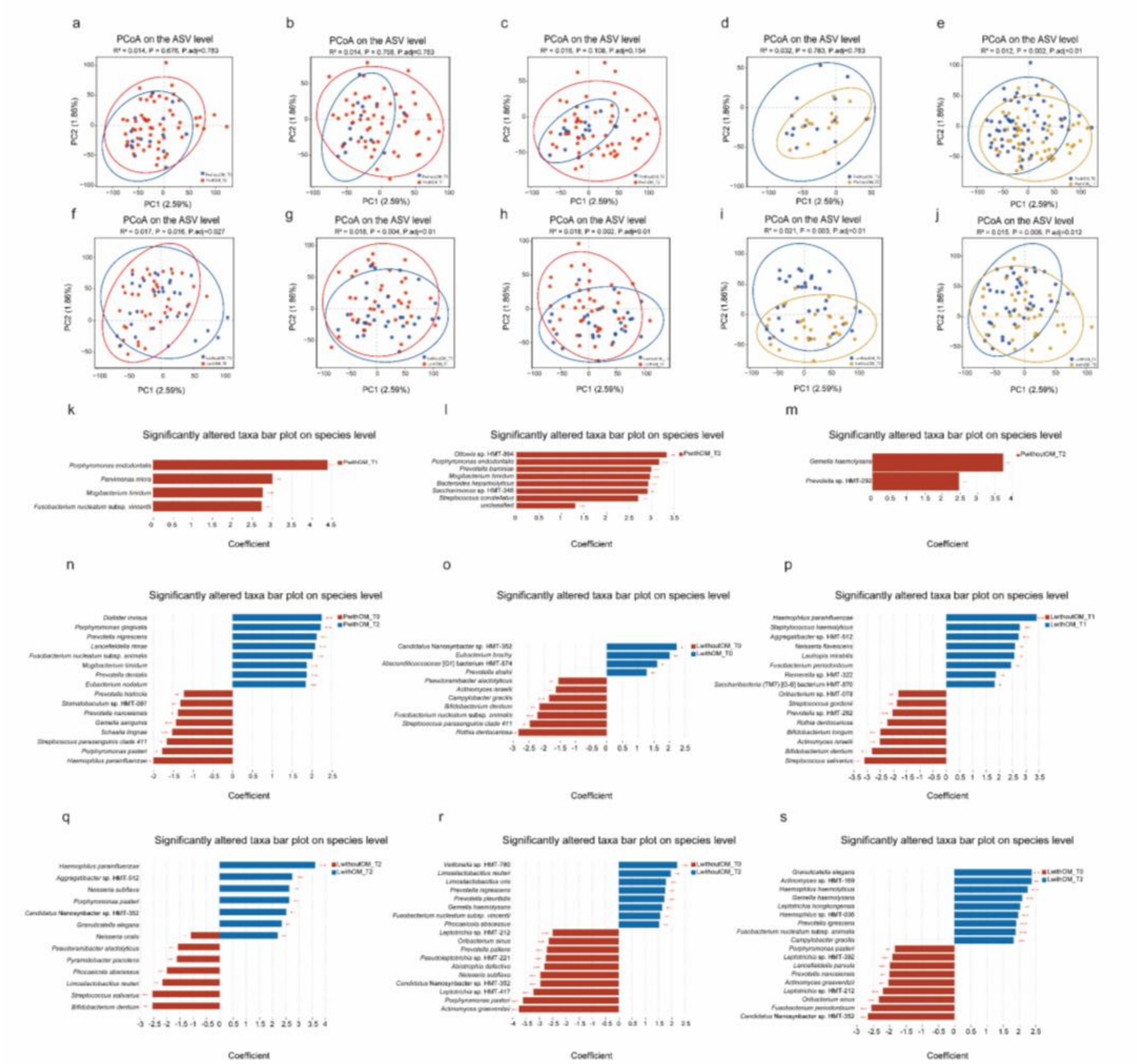

(**a-j**) PCoA plots based on Aitchison distances illustrating salivary microbial β diversity. Panels (**a–e**) represent the placebo group: comparisons between patients with and without OM at T0 (**a**), T1 (**b**), and T2 (**c**), alongside longitudinal shifts from T0 to T2 within the non-OM (**d**) and OM (**e**) subgroups. Panels (**f-j**) represent the *L. reuteri* group: comparisons by OM status at T0 (**f**), T1 (**g**), and T2 (**h**), and longitudinal shifts from T0 to T2 for the non-OM (**i**) and OM (**j**) subgroups. Statistical significance was assessed by PERMANOVA, with *P* values adjusted using the FDR correction. (**k-s**) Differentially abundant taxa at the species level identified by MaAsLin2. For the placebo group (**k-n**): significant differences between patients with and without OM at T1 (**k**) and T2 (**l**), and shifts from T0 to T2 for the non-OM (**m**) and OM (**n**) subgroups. No significant differences were observed at T0. For the *L. reuteri* group (**o–s**): significant differences by OM status at T0 (**o**), T1 (**p**), and T2 (**q**), and shifts from T0 to T2 for the non-OM (**r**) and OM (**s**) subgroups. Positive and negative coefficients indicate enrichment in the comparison and reference groups, respectively. MaAsLin2 models were fitted with appropriate covariate adjustment, and statistical significance was determined using two-sided tests. Asterisks in the figures denote nominal *P* values. **P* < 0.05, ***P* < 0.01, ****P* < 0.001. OM, oral mucositis; T0, baseline; T1, mid-radiotherapy; T2, end-radiotherapy; LwithOM_T0, baseline samples from patients in the *L. reuteri* group who subsequently developed OM; LwithoutOM_T0, baseline samples from patients in the *L. reuteri* group who remained OM-free; LwithOM_T1, patients with OM in the *L. reuteri* group at mid-radiotherapy; LwithoutOM_T1, patients without OM in the *L. reuteri* group at mid-radiotherapy; LwithOM_T2, patients with OM in the *L. reuteri* group at radiotherapy completion; LwithoutOM_T2, patients without OM in the *L. reuteri* group at radiotherapy completion; PwithOM_T0, baseline samples from patients in the placebo group who subsequently developed OM; PwithoutOM_T0, baseline samples from patients in the placebo group who remained OM-free ; PwithOM_T1, patients with OM in the placebo group at mid-radiotherapy; PwithoutOM_T1, patients without OM in the placebo group at mid-radiotherapy; PwithOM_T2, patients with OM in the placebo group at radiotherapy completion; PwithoutOM_T2, patients without OM in the placebo group at radiotherapy completion; ASV, amplicon sequence variant; PCoA, principal coordinates analysis; *L.reuteri, Limosilactobacillus reuteri*. Raw sequencing data are available at NGDC under accession code HRA016457.

Supplementary Fig. 13. Taxonomic and predicted functional shifts between the *Limosilactobacillus reuteri* and placebo groups.

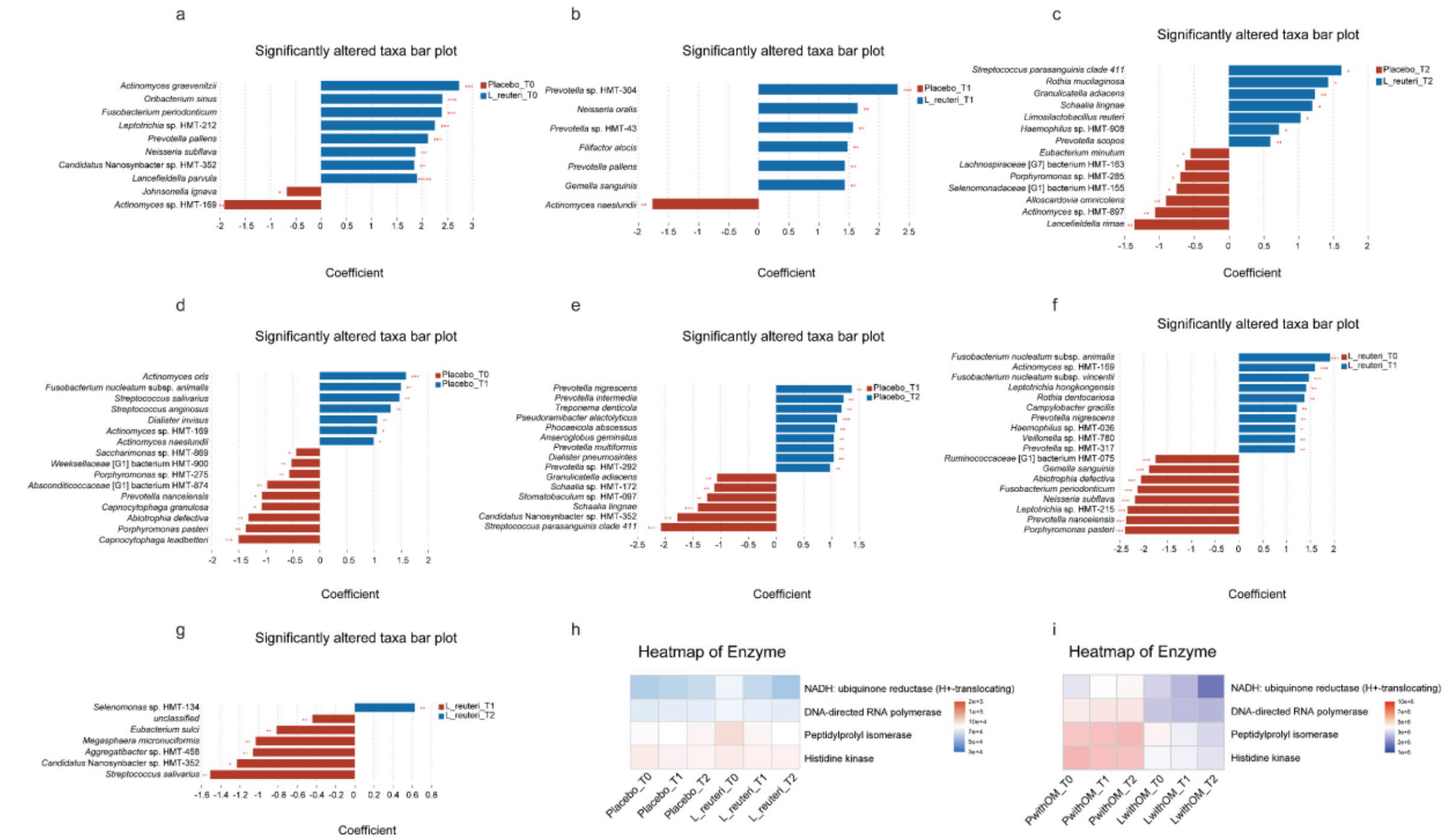

(**a-c**) Differentially abundant species between the *L. reuteri* and placebo groups at T0 (**a**), T1 (**b**), and T2 (**c**) identified by MaAsLin2 analysis. (**d-g**) Longitudinal shifts in microbial composition within the placebo group (T1 vs. T0, **d**; T1 vs. T2, **e**) and the *L. reuteri* group (T1 vs. T0, **f**; T1 vs. T2, **g**). Positive and negative coefficients indicate enrichment in the comparison and reference groups, respectively. MaAsLin2 models were fitted with appropriate covariate adjustment, and statistical significance was determined using two-sided tests. Asterisks in the figures denote nominal *P* values. **P* < 0.05, ***P* < 0.01, ****P* < 0.001. (**h-i**) Heatmaps showing predicted functional profiles (enzymes) based on PICRUSt2 between the *L. reuteri* and placebo groups across assessed timepoints in the overall cohort (**h**) and stratified by patients who developed OM (**i**). ASV: amplicon sequence variant; PC, principal component; PCoA, principal coordinates analysis; Placebo_T0, prior to radiotherapy with a placebo; Placebo_T1, the mid-radiotherapy with a placebo; Placebo_T2, the end of radiotherapy with a placebo; *L. reuteri*, *Limosilactobacillus reuteri*; *L. reuteri*_T0, prior to radiotherapy with the probiotic *Limosilactobacillus reuteri*; *L. reuteri*_T1, the mid-radiotherapy with the probiotic *Limosilactobacillus reuteri*; *L. reuteri*_T2, the end of radiotherapy with the probiotic *Limosilactobacillus reuteri*. Raw sequencing data are available at NGDC under accession code HRA016457.

Supplementary Fig. 14. Schematic diagram illustrating the hypothesized mechanism of action of *Limosilactobacillus reuteri*.

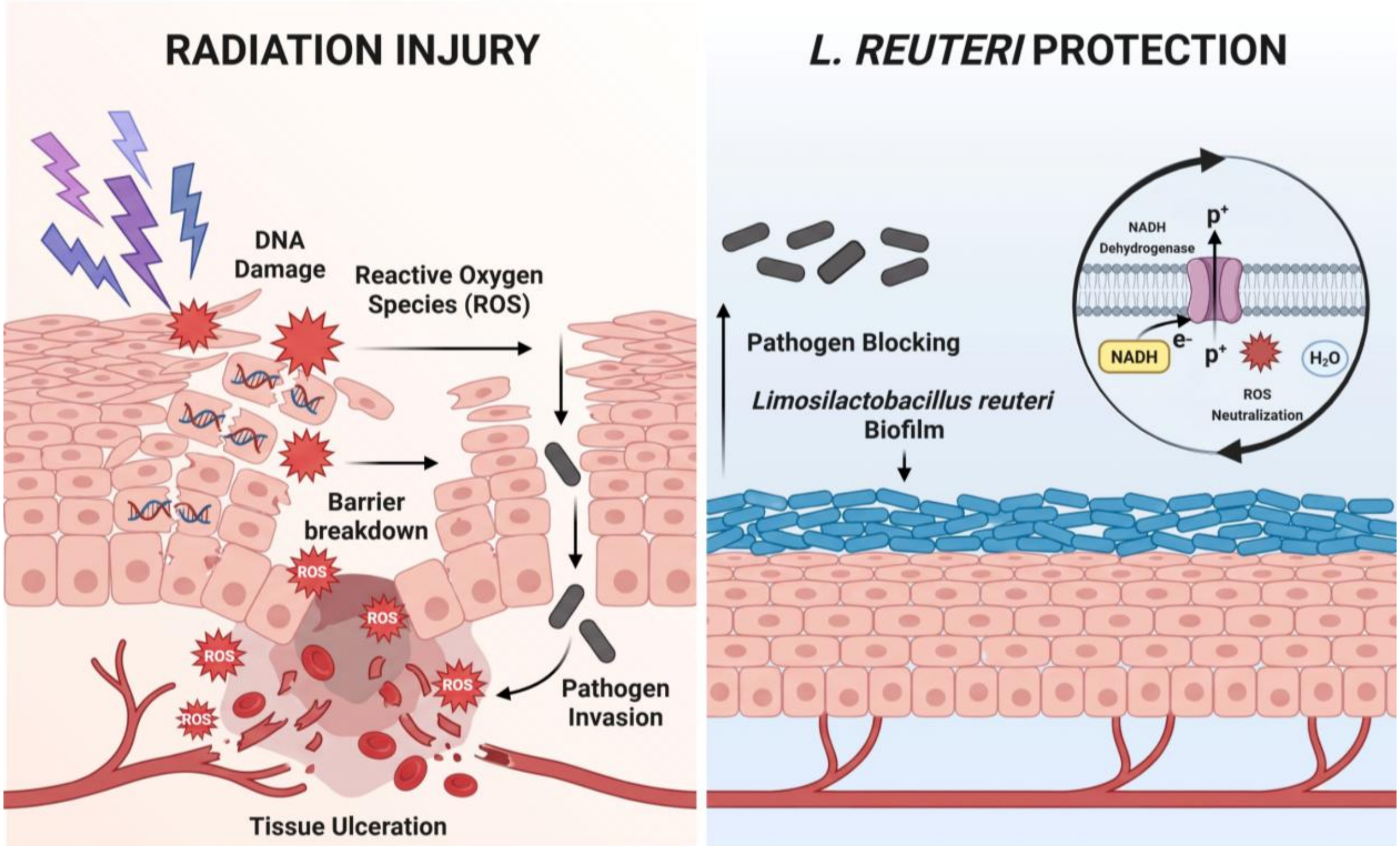

Created in BioRender. Gao, K. (2026) https://BioRender.com/5hzx5j7. All arrows represent hypothetical relationships derived from previously published evidence, which are not validated by the current study.

Supplementary Fig. 15. The probiotic *Limosilactobacillus reuteri* lozenges and placebo lozenges were identical in physical appearance, taste and color.

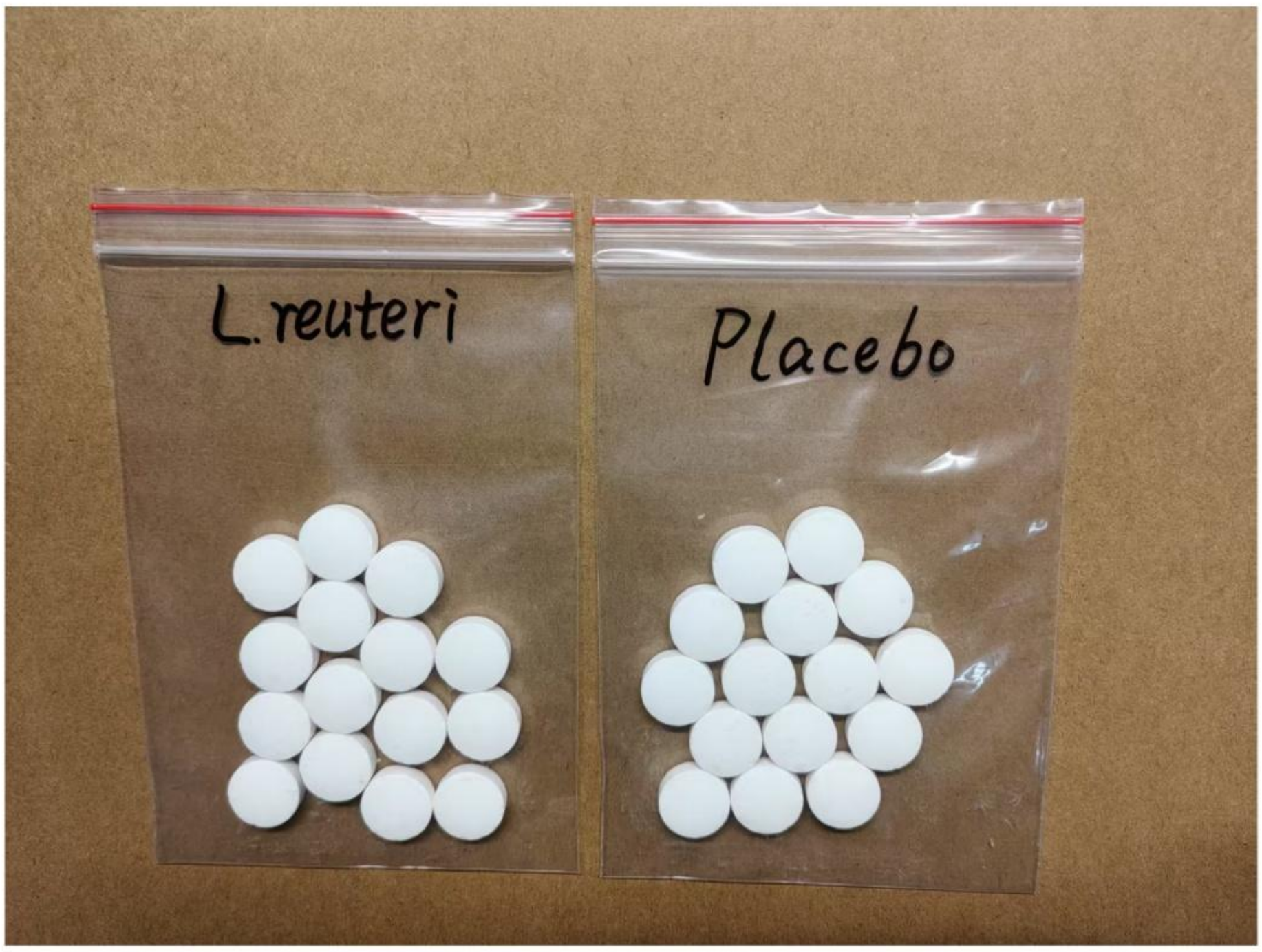

Supplementary Table 1. The characteristics of the excluded patients.

Characteristics	*L. reuteri* (n=9)	Placebo (n=10)	All Patients (n=19)
Sex, No. (%)			
Male	9 (100.0)	9 (90.0)	18 (94.7)
Female	0 (0.0)	1 (10.0)	1 (5.3)
Age, years, mean ± SD	58.8 ± 12.5	59.5 ± 21.1	59.2 ± 17.1
BMI, kg/m^2, mean ± SD	22.5 ± 1.8	21.2 ± 2.9	21.8 ± 2.5
ECOG PS, No. (%)			
0	7 (77.8)	7 (70.0)	14 (73.7)
1	2 (22.2)	3 (30.0)	5 (26.3)
2	0 (0.0)	0 (0.0)	0 (0.0)
Tumor site, No. (%)			
Larynx	4 (44.4)	3 (30.0)	7 (36.8)
Oral cavity	3 (33.3)	3 (30.0)	6 (31.6)
Nasopharynx	1 (11.1)	2 (20.0)	3 (15.8)
Oropharynx	1 (11.1)	1 (10.0)	2 (10.5)
Hypopharynx	0 (0.0)	1 (10.0)	1 (5.3)
Tumor pathological types, No. (%)			
Squamous carcinoma	9 (100.0)	10 (100.0)	19 (100.0)
Others	0 (0.0)	0 (0.0)	0 (0.0)
Treatment type, No. (%)			
Definitive	4 (44.4)	5 (50.0)	9 (47.4)
Postoperative treatment	5 (55.6)	5 (50.0)	10 (52.6)
TNM stage, No. (%)			
I-II	1 (11.1)	2 (20.0)	3 (15.8)
III	4 (44.4)	4 (40.0)	8 (42.1)
IVa	2 (22.2)	3 (30.0)	5 (26.3)
IVb	2 (22.2)	1 (10.0)	3 (15.8)
Tumor primary site, No. (%)			
T1	1 (11.1)	2 (20.0)	3 (15.8)
T2	4 (44.4)	3 (30.0)	7 (36.8)
T3	2 (22.2)	1 (10.0)	3 (15.8)
T4	2 (22.2)	4 (40.0)	6 (31.6)
Nodal involvement, No. (%)			
N0	2 (22.2)	2 (20.0)	4 (21.1)
N1	4 (44.4)	3 (30.0)	7 (36.8)
N2	1 (11.1)	4 (40.0)	5 (26.3)
N3	2 (22.2)	1 (10.0)	3 (15.8)
Concurrent chemotherapy, No. (%)			
Yes	4 (44.4)	4 (40.0)	8 (42.1)
No	5 (55.6)	6 (60.0)	11 (57.9)
Current tobacco use, No. (%)			
Yes	6 (66.7)	8 (80.0)	14 (73.7)
No	3 (33.3)	2 (20.0)	5 (26.3)
Current alcohol use, No. (%)			
Yes	6 (66.7)	5 (50.0)	11 (57.9)
No	3 (33.3)	5 (50.0)	8 (42.1)
Oropharyngeal cancer HPV status, No. (%)			
Positive	0 (0.0)	1 (100.0)	1 (50.0)
Negative	1 (100.0)	0 (0.0)	1 (50.0)
Nasopharynx cancer EBV status, No. (%)			
Positive	1 (100.0)	2 (100.0)	3 (100.0)
Negative	0 (0.0)	0 (0.0)	0 (0.0)
Total radiotherapy dose, Gy, median (IQR)	63.9 (10.0)	70.0 (5.3)	70.0 (7.0)
Dose to oral cavity, Gy, median (IQR)	39.1 (14.7)	38.9 (11.6)	39.1 (11.8)

Supplementary Table 2. Associations of key confounding factors with treatment efficacy

Covariate	Endpoint	ITT		PPS	
		Effect size (95%CI)	*P*	Effect size (95%CI)	*P*
Smoking history	SOM incidence	0.94 (0.67-1.33)	0.7337	0.88 (0.61-1.26)	0.4903
	SOM onset	0.95 (0.55-1.64)	0.8471	0.89 (0.50-1.59)	0.6855
	SOM duration	0.94 (0.63-1.40)	0.8360	0.93 (0.61-1.43)	0.4239
	OM incidence	0.81 (0.65-1.02)	0.0729	0.72 (0.56-0.92)	0.0099
	OM onset	0.76 (0.48-1.20)	0.2349	0.65 (0.39-1.07)	0.0780
	OM duration	0.88 (0.67-1.16)	0.3561	0.82 (0.62-1.10)	0.1554
Alcohol consumption history	SOM incidence	0.74 (0.53-1.02)	0.0688	0.71 (0.49-1.03)	0.0708
	SOM onset	0.61 (0.36-1.05)	0.0724	0.56 (0.31-1.00)	0.0531
	SOM duration	0.70 (0.44-1.03)	0.4380	0.66 (0.39-1.00)	0.3777
	OM incidence	0.77 (0.61-0.97)	0.0262	0.72 (0.56-0.92)	0.0096
	OM onset	0.52 (0.34-0.82)	0.0044	0.40 (0.24-0.67)	0.0004
	OM duration	0.71 (0.53-0.93)	0.2546	0.67 (0.48-0.89)	0.4036
Shannon index	SOM incidence			1.08 (0.87-1.34)	0.5034
	SOM onset			1.23 (0.87-1.74)	0.2331
	SOM duration			1.16 (-1.64, 3.96)	0.8831
	OM incidence			1.09 (0.96-1.23)	0.1905
	OM onset			1.62 (1.07-2.47)	0.0108
	OM duration			1.48 (-3.60, 6.55)	0.4888
Actinomyces graevenitzii	SOM incidence			1.00 (0.99-1.00)	0.3159
	SOM onset			1.00 (0.99-1.00)	0.5987
	SOM duration			4.56e-4 (-3.66e-3, 4.57e-3)	0.6367
	OM incidence			1.00 (0.99-1.00)	0.3828
	OM onset			1.00 (0.99-1.00)	0.6141
	OM duration			3.26e-3 (-4.40e-3, 0.01)	0.0723
HPV status	SOM incidence	1.35 (0.22-8.41)	1.0000	0.97 (0.14-6.49)	1.0000
	OM incidence	3.84 (0.15-99.1)	0.5292	2.42 (0.08-69.3)	1.0000

Abbreviations: Model 2 adjusted for smoking and alcohol history in the ITT set, with additional adjustment for Shannon index and *Actinomyces graevenitzii* abundance in the PP population. The incidence of SOM/OM was analyzed using modified Poisson regression with robust standard errors, time-to-onset endpoints were analyzed using Cox proportional hazards regression, and duration of SOM/OM was analyzed using a two-part model. All statistical tests were two-sided. The placebo group was used as the reference category. Multiple comparisons were adjusted using the Bonferroni correction, with a corrected significance threshold of $P < 0.0083$. For categorical variables, the effect size is RR for incidence, HR for time to onset, and MR for duration. For continuous variables, the effect size is RR for incidence, HR for time to onset, and AME for duration. Due to the limited sample size of HPV-status-stratified patients (restricted to oropharyngeal cancer cases), HPV status was not included in the adjustment model but was analyzed as a separate subgroup. *L. reuteri, Limosilactobacillus reuteri*; OM, oral mucositis; SOM, severe OM; SD, standard deviation; RR: relative risk; HR: hazard ratio; MR: mean ratio; AME, average marginal effect; CI: confidence interval; HPV, human papillomavirus; ITT, intention-to-treat; PPS, per-protocol set.

Supplementary Table 3. Efficacy results in per-protocol set.

	L. reuteri (n=71)	Placebo (n=70)	Effect size (95% CI)	*P*
**Unadjusted primary analysis**				
SOM incidence, %	38.0	61.4	0.62 (0.44-0.88)	.0090
SOM onset, days, median	28.0	20.0	0.42 (0.26-0.68)	.0003
SOM duration, days, mean (SD)	6.7 (10.0)	15.4 (15.0)	0.44 (0.21-0.69)	.0022
OM incidence, %	56.3	78.6	0.72 (0.56-0.91)	.0084
OM onset, days, median	18.5	9.0	0.39 (0.26-0.59)	<.0001
OM duration, days, mean (SD)	20.4 (22.4)	35.9 (25.0)	0.57 (0.39-0.77)	.0132
Radiotherapy treatment breaks ≥ five consecutive fractions, n (%)	1 (1.4)	1 (1.4)		
**Model 1**				
SOM incidence, %	38.0	61.4	0.62 (0.45-0.86)	.0039
SOM onset, days, median	28.0	20.0	0.44 (0.27-0.72)	.0009
SOM duration, days, mean (SD)	6.7 (10.0)	15.4 (15.0)	0.49 (0.26-0.74)	.0305
OM incidence, %	56.3	78.6	0.72 (0.59-0.89)	.0026
OM onset, days, median	18.5	9.0	0.37 (0.24-0.56)	<.0001
OM duration, days, mean (SD)	20.4 (22.4)	35.9 (25.0)	0.58 (0.40-0.77)	.0151
**Model 2**				
SOM incidence, %	38.0	61.4	0.59 (0.42-0.84)	.0035
SOM onset, days, median	28.0	20.0	0.36 (0.21-0.63)	.0003
SOM duration, days, mean (SD)	6.7 (10.0)	15.4 (15.0)	0.47 (0.24-0.74)	.0804
OM incidence, %	56.3	78.6	0.71 (0.57-0.88)	.0022
OM onset, days, median	18.5	9.0	0.25 (0.15-0.40)	<.0001
OM duration, days, mean (SD)	20.4 (22.4)	35.9 (25.0)	0.56 (0.38-0.76)	.0261

Abbreviations: Unadjusted incidence comparisons were performed using the χ^2 test, with RR and 95% CI calculated separately using the Katz logarithmic method. Time-to-onset endpoints were analyzed using the log-rank test, with HR and 95% CI reported. Duration endpoints were analyzed using a two-part model, with MR and 95% CI reported. Model 1 was adjusted for tumor site, oral radiotherapy dose, and chemotherapy. Model 2 further adjusted for smoking history, alcohol consumption history, Shannon index, and the absolute abundance of *Actinomyces graevenitzii*. In adjusted analyses, modified Poisson regression was used for incidence (aRR, 95% CI), Cox proportional hazards regression was used for time-to-onset endpoints (aHR, 95% CI), and a two-part model was used for duration endpoints (aMR, 95% CI). All statistical tests were two-sided. Multiple comparisons were accounted for using a Bonferroni-adjusted significance threshold of $P < 0.0083$. *L. reuteri, Limosilactobacillus reuteri*; OM, oral mucositis; SOM, severe OM; SD, standard deviation; RR: relative risk; HR: hazard ratio; MR: mean ratio; aRR: adjusted relative risk; aHR: adjusted hazard ratio; aMR: adjusted mean ratio; CI: confidence interval.

Supplementary Table 4. Results of the piecewise Cox regression analysis for SOM/OM onset in the intention-to-treat population.

		HR (95% CI)	*P*
SOM onset	<36 days	0.37 (0.23-0.59)	<0.0001
	⩾36 days	2.96 (0.34-25.44)	0.3230
OM onset	<30 days	0.40 (0.27-0.58)	<0.0001
	⩾30 days	1.00 (0.10-10.33)	1.000

The proportional hazards assumption was assessed using Schoenfeld residuals tests before Cox regression analysis. Piecewise Cox proportional hazards regression was performed to analyze time-to-onset outcomes. All statistical tests were two-sided. Hazard ratios (HRs) and 95% confidence intervals (CIs) are presented. Multiple comparisons were accounted for using a Bonferroni-adjusted significance threshold of $P < 0.0083$. OM, oral mucositis; SOM, severe OM.

Supplementary Table 5. Efficacy results in patients with SOM/OM.

Analysis Set	Endpoint	*L.reuteri*	Placebo	Effect size (95%CI)	*P*
Participants in the ITT population who developed SOM (N=79)/OM (N=108)	**Unadjusted primary analysis**				
	SOM duration, days, mean (SD)	16.9 (8.2)	24.4 (10.6)	NA	.0025
	SOM onset, days, median (IQR)	28.0 (11.0)	21.0 (7.0)	0.40 (0.25-0.64)	.0001
	OM duration, days, mean (SD)	35.4 (16.8)	45.5 (17.8)	NA	.0061
	OM onset, days, median (IQR)	20.0 (7.0)	9.0 (10.0)	0.46 (0.31-0.68)	<.0001
	**Adjusted model**				
	SOM duration, days, mean (SD)	16.9 (8.2)	24.4 (10.6)	0.76 (0.61-0.94)	0.0116
	SOM onset, days, median (IQR)	28.0 (11.0)	21.0 (7.0)	0.36 (0.21-0.60)	<.0001
	OM duration, days, mean (SD)	35.4 (16.8)	45.5 (17.8)	0.77 (0.64-0.92)	0.0037
	OM onset, days, median (IQR)	20.0 (7.0)	9.0 (10.0)	0.37 (0.24-0.57)	<.0001
Participants in the PPS who developed SOM (N=70)/OM (N=95)	**Unadjusted primary analysis**				
	SOM duration, days, mean (SD)	17.7 (8.2)	25.1 (10.9)	NA	.0064
	SOM onset, days, median (IQR)	28.0 (11.0)	20.0 (8.0)	0.29 (0.17-0.52)	<.0001
	OM duration, days, mean (SD)	36.3 (17.6)	45.7 (18.5)	NA	.0176
	OM onset, days, median (IQR)	18.5 (8.0)	9.0 (6.5)	0.40 (0.26-0.61)	<.0001
	**Adjusted model**				
	SOM duration, days, mean (SD)	17.7 (8.2)	25.1 (10.9)	0.78 (0.63-0.98)	0.0305
	SOM onset, days, median (IQR)	28.0 (11.0)	20.0 (8.0)	0.25 (0.14-0.46)	<.0001
	OM duration, days, mean (SD)	36.3 (17.6)	45.7 (18.5)	0.78 (0.65-0.95)	0.0151
	OM onset, days, median (IQR)	18.5 (8.0)	9.0 (6.5)	0.33 (0.21-0.53)	<.0001

Unadjusted onset time comparisons were performed using the two-sided log-rank test (HR, 95% CI), and duration comparisons were performed using the two-sided Mann–Whitney U test. The adjusted model included tumor site, oral radiotherapy dose, and chemotherapy as covariates. Cox proportional hazards regression was used for onset analysis (HR, 95% CI), and Gamma regression was used for duration analysis (MR, 95% CI) in adjusted models. All statistical tests were two-sided. *L. reuteri*, *Limosilactobacillus reuteri*; OM, oral mucositis; SOM, severe OM; MR: mean ratio; HR: hazard ratio; CI: confidence interval; NA, not applicable.

Supplementary Table 6. The incidence of grade 2 or higher hyposalivation in two groups.

	No. (%) of patients	
	L. reuteri	**Placebo**
Baseline	0 (0)	0 (0)
Mid-term radiotherapy	45 (63)	49 (70)
End of radiotherapy	58 (82)	60 (86)
1 month post radiotherapy	18 (25)	16 (23)
3 months post radiotherapy	14 (20)	11 (16)

Supplementary Table 7. Treatment-related adverse events in the *Limosilactobacillus reuteri* and placebo groups.

Adverse events grade, **No. (%)**	***L. reuteri* (n=80)**			**Placebo (n=80)**		
	Grade 1	Grade 2	Grade ≥3	Grade 1	Grade 2	Grade ≥3
Abdominal distension	1 (1.3)	0 (0)	0 (0)	0 (0)	0 (0)	0 (0)
Diarrhea	1 (1.3)	0 (0)	0 (0)	0 (0)	0 (0)	0 (0)
Dyspepsia	1 (1.3)	0 (0)	0 (0)	1 (1.3)	0 (0)	0 (0)

Supplementary Table 8. Summary of any-grade adverse events in all patients.

Adverse events grade, **No. (%)**	***L. reuteri* (n=80)**			**Placebo (n=80)**		
	Grade 1	Grade 2	Grade ≥3	Grade 1	Grade 2	Grade ≥3
Abdominal distension	1 (1.3)	0 (0)	0 (0)	0 (0)	0 (0)	0 (0)
Alopecia	20 (25.0)	0 (0)	0 (0)	18 (22.5)	0 (0)	0 (0)
Anemia	13 (16.3)	0 (0)	0 (0)	17 (21.3)	2 (2.5)	1 (1.3)
Anxiety	7 (8.8)	1 (1.3)	0 (0)	7 (8.8)	0 (0)	0 (0)
Cough	22 (27.5)	6 (7.5)	0 (0)	23 (28.8)	7 (8.8)	0 (0)
Constipation	4 (5.0)	0 (0)	0 (0)	7 (8.8)	1 (1.3)	0 (0)
Decreased appetite	34 (42.5)	8 (10.0)	0 (0)	22 (27.5)	27 (33.8)	0 (0)
Depression	4 (5.0)	0 (0)	0 (0)	3 (3.8)	0 (0)	0 (0)
Diarrhea	1 (1.3)	0 (0)	0 (0)	0 (0)	0 (0)	0 (0)
Dizziness	8 (10.0)	0 (0)	0 (0)	11 (13.8)	0 (0)	0 (0)
Dyspepsia	1 (1.3)	0 (0)	0 (0)	1 (1.3)	0 (0)	0 (0)
Dysphagia	33 (41.3)	15 (18.8)	10 (12.5)	20 (25.0)	29 (36.3)	13 (16.3)
Dyspnea	1 (1.3)	0 (0)	0 (0)	2 (2.5)	0 (0)	0 (0)
Face and neck edema	20 (25.0)	2 (2.5)	0 (0)	21 (26.3)	2 (2.5)	0 (0)
Fatigue	36 (45.0)	4 (5.0)	1 (1.3)	34 (42.5)	7 (8.8)	2 (2.5)
Headache	5 (6.3)	0 (0)	0 (0)	8 (10.0)	1 (1.3)	0 (0)
Hoarseness	7 (8.8)	0 (0)	0 (0)	6 (7.5)	0 (0)	0 (0)
Insomnia	24 (30.0)	3 (3.8)	0 (0)	22 (27.5)	1 (1.3)	0 (0)
Leukopenia	8 (10.0)	3 (3.8)	3 (3.8)	8 (10.0)	4 (5.0)	6 (7.5)
Lymphopenia	25 (31.3)	16 (20.0)	17 (21.3)	28 (35.0)	14 (17.5)	19 (23.8)
Nausea	26 (32.5)	8 (10.0)	3 (3.8)	16 (20.0)	25 (31.3)	2 (2.5)
Neck pain	4 (5.0)	0 (0)	0 (0)	6 (7.5)	0 (0)	0 (0)
Neutropenia	4 (5.0)	0 (0)	0 (0)	5 (6.3)	1 (1.3)	0 (0)
Oropharyngeal pain	35 (43.8)	19 (23.8)	3 (3.8)	31 (38.8)	32 (40.0)	6 (7.5)
Pyrexia	1 (1.3)	0 (0)	0 (0)	1 (1.3)	0 (0)	0 (0)
Radiation skin injury	19 (23.8)	39 (48.8)	4 (5.0)	24 (30.0)	36 (45.0)	5 (6.3)
Stoma site pain	13 (16.3)	2 (2.5)	0 (0)	14 (17.5)	2 (2.5)	0 (0)
Thrombocytopenia	3 (3.8)	0 (0)	0 (0)	4 (5.0)	0 (0)	0 (0)
Tinnitus	2 (2.5)	0 (0)	0 (0)	3 (3.8)	0 (0)	0 (0)
Vomiting	10 (12.5)	1 (1.3)	0 (0)	7 (8.8)	2 (2.5)	0 (0)

Supplementary Table 9. Tumor response in patients receiving definitive radiotherapy between *Limosilactobacillus reuteri* group and placebo group.

Response	*L. reuteri* (n=38)	Placebo (n=40)
Complete response, No. (%)	34 (89.5)	36 (90.0)
Partial response, No. (%)	3 (7.9)	2 (5.0)
Progressive disease, No. (%)	1 (2.6)	2 (5.0)

NOTE. Tumor response assessments were conducted at baseline and 3 months post-radiotherapy in patients with head and neck cancer receiving definitive radiotherapy. Radiographic evaluation included contrast-enhanced CT or MRI of the head and neck, contrast enhanced CT of the thorax, and abdominal ultrasound. Changes in tumor size were evaluated according to RECIST v1.1 criteria.

Supplementary Table 10. World Health Organization Oral Toxicity Scale.

Grade	Definition
0	None
1	Soreness, erythema
2	Erythema, ulcers, ability to eat solids
3	Ulcers, requiring liquid diet
4	Alimentation not possible

Supplementary Table 11. Dry mouth scale according to CTCAE version 5.0.

Grade	Definition
0	None
1	Symptomatic (e.g., dry or thick saliva) without significant dietary alteration; unstimulated saliva flow 0.2 to 0.3 ml/min.
2	Moderate symptoms; oral intake alterations (e.g., copious water, other lubricants, diet limited to purees and/or soft, moist foods); unstimulated saliva flow 0.1 to 0.2 ml/min.
3	Inability to adequately aliment orally; tube feeding or TPN indicated; unstimulated saliva flow <0.1 ml/min.

Study Protocol

***Limosilactobacillus reuteri* for Radiation-Induced Oral Mucositis in Patients with Head and Neck Cancer: A Randomized Clinical Trial**

Principal Investigator: Dr. Xingchen Peng

Sponsor: West China Hospital, Sichuan University

1. Introduction
2. Objectives
2.1 Primary Objective 30
2.2 Secondary Objectives 30
2.3 Exploratory Objectives 31
3. Selection of Subjects
3.1 Inclusion Criteria 31
3.2 Exclusion Criteria 31
4. Screening and Baseline Evaluation
4.1 Screening 32
4.2 Baseline (1 week before RT) 33
5. Randomization Procedure
6. Intervention0020Strategies
6.1 Anti-cancer Treatment Regimen 34
6.2 Investigate Product 34
6.3 Study Lozenges Administration 34
6.4 Concomitant Medications/Treatments 35
6.5 Withdrawal Criteria 35
7. Efficacy Assessment
7.1 Quality Assurance in Radiotherapy Target Delineation 35
7.2 Oral Mucositis Assessment 35
7.3 Quality Assurance of Sample Collection 36
8. Safety Assessment
8.1 Definition of AE 36
8.2 Assessment of AE 37
8.3 AE Causality Assessment 37
9. Other Endpoints Assessment
10. Laboratory Assessment
10.1 Sample Collection 38
10.2 DNA extraction, PCR amplification and Illumina MiSeq sequencing 38
10.3 Data Processing 39
11. Statistical Analysis
11.1 Simple Size 39
11.2 Analysis Method 40
Appendix 1: Eastern Cooperative Oncology Group (ECOG) - Performance Status Conversion
Appendix 2: World Health Organization (WHO) Oral Toxicity Scale 43
Appendix 3: Patient's Diary
Appendix 4: Weekly Oral Mucositis Questionnaire (OMWQ) (English version)
Appendix 5: Taste Strips Test
Appendix 6: Assessment of Xerostomia
Appendix 7: European Organization for Research and Treatment of Cancer (EORTC) Quality-of-life Questionnaire Core 30 (EORTC QLQ-C30) and Head and Neck Cancer Module (EORTC QLQ-H&N35) (English version)
Appendix 8: National Cancer Institute Common Terminology Criteria for Adverse Events (NCI CTCAE) version 5.0

1. Introduction

Oral mucositis (OM) is a ubiquitous complication in head and neck cancer (HNC) patients undergoing radiotherapy (RT), with severe OM (SOM) developing in approximately 50–70% of cases.[1,2] SOM is characterized by extensive mucosal damage, leading to persistent intense pain, impaired oral intake, and difficulties in talking and swallowing.[3] Collectively, these acute phase responses lead to malnutrition, profoundly diminish quality of life (QoL), and frequently necessitate unplanned RT interruptions, thereby jeopardizing therapeutic outcomes in HNC patients. While the Multinational Association of Supportive Care in Cancer/International Society of Oral Oncology (MASCC/ISOO) have proposed several interventions for radiation-induced OM (RIOM), the development of novel therapeutic strategies remains paramount.[4]

Accumulating findings suggest that dysbiosis of the oral microbiota contributes to the development and progression of RIOM, and that oral probiotics can alleviate OM by reestablishing mucosal homeostasis.[5,] As a naturally dominant probiotic strain colonizing the human oral cavity, *Limosilactobacillus reuteri (L. reuteri)* has well-established roles in maintaining oral microecological balance—specifically by inhibiting pathogenic bacterial proliferation and promoting colonization of beneficial commensals.[6-8] It has been approved for the management of stomatological inflammatory conditions, including gingivitis and periodontitis.[9-13] In addition, our basic research showed that *L. reuteri* has efficacy in managing RIOM.

Despite promising preliminary findings, the effectiveness of the *L. reuteri* supplementation in managing RIOM among head and neck cancer patients is not yet fully understood and is not sufficiently investigated. To assess its clinical efficacy and clarify its therapeutic potential, we conducted a double-blind, placebo-controlled, randomized Phase II trial.

2. Objectives

2.1 Primary Objective

The primary objective of this study is to evaluate and compare the incidence of SOM (WHO grade >2).

2.2 Secondary Objectives

- To evaluate and compare the time to onset of SOM.

- To evaluate and compare the duration of SOM, as assessed from the first determination of SOM to the first instance of non-severe OM (WHO grade < 3), without a subsequent instance of SOM.
- To evaluate and compare the incidence of OM, time to onset of OM and duration of OM.

2.3 Exploratory Objectives

- To evaluate and compare the patients' quality of life (QoL).
- To evaluate and compare mouth and throat soreness (MTS) and oral activities scores.
- To evaluate and compare the incidence of xerostomia.
- To evaluate and compare gustatory function.
- To evaluate and compare the change in body mass index (BMI) during RT.
- To evaluate and compare the use of parenteral nutrition.
- To evaluate and compare the use of analgesics.
- To evaluate and compare the number of patients who missed five or more consecutive radiation fractions.
- To evaluate the safety of *L. reuteri*.
- To evaluate the composition and analyze the longitudinal dynamics of salivary microbiota.

3. Selection of Subjects

3.1 Inclusion Criteria

- Patients with histopathologically confirmed head and neck cancer without evidence of distant metastasis;
- Aged 18 to 80 years;
- Systemic functional status Eastern Cooperative Oncology Group (ECOG) (Appendix 1) rating ≤2;
- Receiving definitive RT or postoperative adjuvant RT at a dose of 60-72Gy with/without concurrent chemotherapy;
- Sign the informed consent.

3.2 Exclusion Criteria

- Patients with known allergy to probiotics (e.g. itchy skin, diarrhea, rhinitis) or severe allergic constitution;

- Use of antibiotics, antifungal drugs, or antimicrobial mouthwash within 1 month of the study;
- As assessed by a qualified dentist/oral physician, the presence of poor oral hygiene (a simplified oral hygiene index (OHI-S) score of ⩾ 3), and/or severe periodontal disease, defined as at least two teeth with a probing pocket depth (PPD) of ⩾ 6 mm, clinical attachment loss (CAL) of ⩾ 5 mm, and bleeding on probing (BOP) in at least two non-adjacent teeth;

*The Simplified Oral Hygiene Index (OHI-S) comprises the Debris Index (DI-S) and the Calculus Index (CI-S). Each index is scored from 0 to 3 based on the area of tooth surface coverage: a score of 0 indicates no debris or calculus; 1 indicates debris covering no more than one-third of the tooth surface or supragingival calculus covering only a fleck of the surface; 2 indicates debris or calculus covering more than one-third but less than two-thirds; and a score of 3 indicates debris or calculus covering two-thirds or more of the tooth surface. The examination is performed on the buccal and lingual surfaces of six designated index teeth, and the final OHI-S score is the average of all DI-S and CI-S scores.

- Any previous RT to the head and neck region;
- Any other condition deemed by the investigator to be incompatible with study participation (e.g., pregnancy, lactation, severe comorbidities, or significant psychiatric conditions).

4. Screening and Baseline Evaluation

4.1 Screening

Patients who meet the inclusion criteria and do not meet the exclusion criteria will be enrolled in the study. During screening, the following assessment steps will be completed and recorded:

- Assessment of inclusion and exclusion criteria;
- Basic information of patients (age, gender, native place, allergy history, smoking, alcohol use, etc.);
- Past medical history, concomitant medications, recent antibiotics/antifungal drugs use and concomitant therapies;
- Physical examination including height, weight, etc.;

- Dental examination including oral hygiene, caries, periodontal disease and impacted tooth etc. The exam must be performed by a licensed dentist. If necessary, dental filling, endodontic treatment, extraction of non-restorable teeth and periodontal non-surgical treatment are performed (Within 1 month before RT);

  * Standardized oral hygiene refers to that all patients underwent a professional dental cleaning (scaling and polishing) during the screening period. And all patients were instructed to follow a standardized daily oral care regimen throughout radiotherapy, which comprised brushing with a soft-bristled toothbrush at least twice daily, performing gentle flossing once daily, and rinsing the mouth with water after each meal. Oral hygiene education was provided by trained study personnel during the study screening period, and adherence to the oral hygiene regimen was reviewed and reinforced at each scheduled follow-up visit during the entire study course. Specifically, after using the lozenge, the patients should avoid eating, drinking and conducting any oral hygiene activities for at least 1 hour.
- Blood routine and biochemical routine examination;
- Malignant tumor pathological classification and TNM stage;
- Planned RT and chemotherapy parameters;
- OM assessment according to WHO oral toxicity Scale (Appendix 2);
- ECOG Performance Status.

4.2 Baseline (1 week before RT)

Prior to receiving the first lozenge of *L. reuteri* or placebo the following observations and procedures will be conducted for all patients:

- Confirm continued patient eligibility by reviewing inclusion/exclusion criteria;
- Provide the daily diary for recording the number of lozenges or other drugs used (Appendix 3);
- Complete the Oral Mucositis Weekly Questionnaire (OMWQ) (Appendix 4);
- OM assessment according to WHO oral toxicity Scale;
- Measure body weight and ECOG Performance Status;
- Assessment of gustatory function (Appendix 5);
- Assessment of xerostomia (Appendix 6);

- Complete the EORTC QLQ-C30 and H&N35 questionnaires (Appendix 7);
- Collect unstimulated saliva.
- Provide instructions for maintaining oral hygiene.

5. Randomization Procedure

All patients will be randomly assigned in a 1:1 ratio to receive probiotic *L. reuteri* or placebo via a computer-generated randomization list using a randomized permutation block design. The pharmacists who are not involved in the implementation of the trial will pre-package the *L. reuteri* and placebo in identical containers. The lozenges will be distributed in accordance with random numbers. The assignment order list, block size and random numbers will be sealed in opaque envelopes and stored at West China Hospital, Sichuan University.

6. Intervention Strategies

6.1 Anti-cancer Treatment Regimen

The intervention strategies are generally referred to the NCCN and CSCO guidelines. The total radiation dose and oral cavity dose will be recorded in the case report form (CRF) in detail. All patients undergo RT in daily 1.8-2.2 Gy, five times per week (total, 6-6.5 weeks), cumulative tumor dose of 60-72 Gy, and all RT is delivered using volumetric modulated arc therapy (VMAT) technology. Concurrent chemotherapy consists of cisplatin 100 mg/m^2 every 3 weeks.

6.2 Investigate Product

L. reuteri group: *L. reuteri* lozenges contain 2×10^8 CFU viable cells of *L. reuteri* as the active ingredient, with isomalt and natural flavor as inactive ingredients.

Placebo group: Placebo lozenges contain isomalt and natural flavor used as excipients in the inactive formulation.

**L. reuteri* lozenges and placebo lozenges are identical in appearance, taste and color.

6.3 Study Lozenges Administration

L. reuteri/placebo lozenge: The patients will be instructed to suck a *L. reuteri* or placebo lozenge twice daily from the beginning to the end of RT. After using the lozenge, the patients should avoid eating, drinking and conducting any oral hygiene activities for at least 1 hour.

6.4 Concomitant Medications/Treatments

Provide routine health education, routine nursing, and relevant symptomatic support and treatment measures. Normal oral rinses (pure water), topical analgesics, and nutritional support are allowed. However, the use of systemic medications (such as antibiotics or antifungal agents), topical mouthwashes or oral rinses other than pure water (such as antibacterial mouthwashes or baking soda solutions), and any other experimental interventions is prohibited.

6.5 Withdrawal Criteria

- The subjects request to end study medication or withdraw informed consent.
- The subjects experience adverse events (AEs) and the investigators consider that the study should be stopped.
- The investigators determine that the best option for the subjects' benefit is to discontinue the trial intervention.
- In the opinion of the investigators, the subjects intentionally or unintentionally do not comply with the study protocol and it is necessary to stop the prevention study.
- Other circumstances that require withdrawal from the study.

7. Efficacy Assessment

7.1 Quality Assurance in Radiotherapy Target Delineation

The RT treatment strategies are implemented in accordance with the NCCN and CSCO clinical guidelines. The target volumes, including the clinical target areas (CTV) and organs at risk (OARs), are delineated based on consensus contouring guidelines, with the definition of the oral cavity specifically referenced to the anatomical criteria described by Mir et al.[14] All patients receive RT with a standardized dose regimen. The prescribed dose is delivered in daily fractions of 1.8 – 2.2 Gy, with a cumulative tumor dose of 60 – 72 Gy over a treatment duration of 6 – 6.5 weeks, and the RT is delivered using VMAT technology. To ensure treatment accuracy and consistency, rigorous quality control (QC) measures are applied throughout the RT workflow. For target volume delineation, all contours are independently reviewed by at least two senior radiation oncologists to minimize inter-observer variability.

7.2 Oral Mucositis Assessment

OM severity is evaluated twice weekly (with intervals of at least 48 hours) during RT and weekly for 8 weeks post-RT using the WHO grading scale by GCP-certified dental professionals trained under China's National Medical Products Administration guidelines, with all assessors receiving standardized WHO grading training prior to trial initiation to ensure consistent application. To maximize endpoint reliability, two independent, blinded evaluators assess all efficacy endpoints. Any discordance (defined as a ⩾ 1-grade difference in OM scoring) is reviewed by a third senior assessor, while inconsistent or incomplete assessments are flagged for consensus discussion before data locking. The assessment of OM are shown in the figure below.

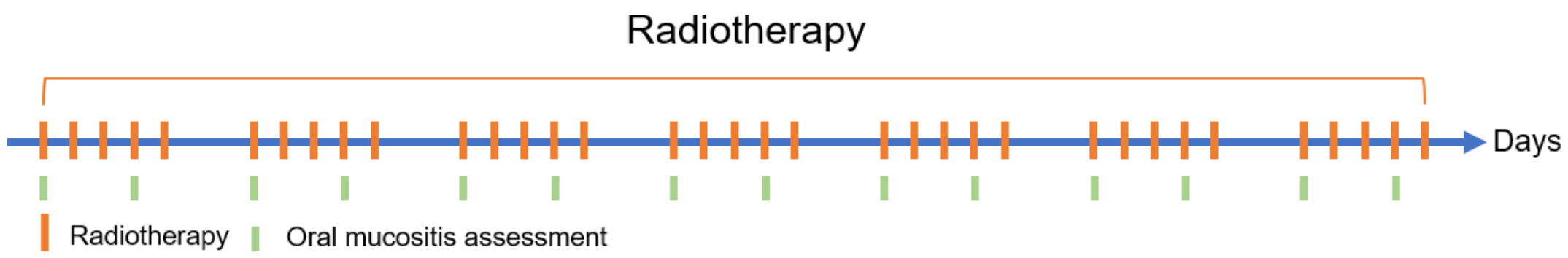

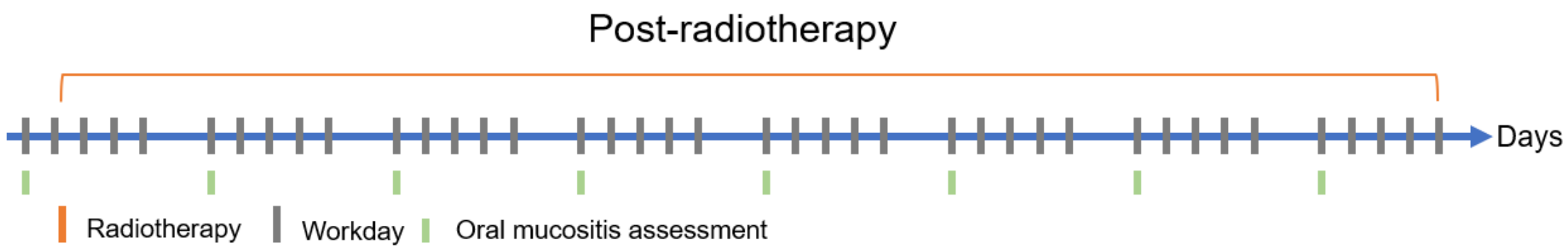

*The timing of assessments for this measure is tailored to each individual's actual number of radiotherapy sessions. A longitudinal follow-up protocol is developed at enrollment and strictly followed during the study period.

*Assessing the impact of OM on a patient's ability to eat is essential for accurate scoring of the WHO scale. First, assessors should clarify the patient's food intake and standardize the definition of food forms. Second, determine whether dietary changes are caused by OM-related pain.

7.3 Quality Assurance of Sample Collection

Unstimulated whole saliva will be collected by spitting method at baseline (before RT initiation), mid-term RT (usually week 3), and at the end of RT (usually week 6 or 6.5). All samples will be frozen immediately at −20 °C and then stored at −80 °C until analysis.

8. Safety Assessment

8.1 Definition of AE

An AE is any adverse medical event in a clinical study subject receiving a drug, regardless of cause and effect. A serious AE (SAE) is an AE that meets any of the following criteria: 1) Death; 2) Life-threatening; 3) Results in permanent or significant disability or impairment of organ function; 4) Congenital malformations/birth defects in newborns/infants born from mothers using the study lozenges; 5) Requires hospitalization or prolonged hospitalization; 6) The investigators determine that it is a major medical event.

The investigators should ask the patient's contact for AE information.

8.2 Assessment of AE

All AEs, whether being reported by the patient or recorded by the researcher, should be recorded in the patient's medical record and the adverse event CRF during RT. AE will be assessed using the National Cancer Institute Common Terminology Criteria for Adverse Events (CAECT; version 5.0) (Appendix 8) during RT. If an AE is not within the range given by this standard, the severity will be assessed using the following scale: Grade 1 (mild): asymptomatic or mild symptoms; clinical or diagnostic observations; or no treatment required; Grade 2 (moderate): need minimal, local, or noninvasive intervention; or limited age-appropriate instrumental activities in daily living; Grade 3: Severe or medically significant but not immediately life-threatening: hospitalization or prolongation of hospitalization; disabling; or limited self-care activities of daily living; Grade 4: Life-threatening consequences, or urgent treatment required; Grade 5: AEs leading to death.

8.3 AE Causality Assessment

The investigators should determine whether the AE is related to the study drug based on his knowledge of the patient, the circumstances in which the event occurred, an assessment of any possible alternative causes, and state "relevant" or "irrelevant" accordingly. The following guidelines should be considered: 1) There was a time relationship between the occurrence of the AE and the initiation of the study drug administration. 2) The AE is related to known AEs of the drug. 3) The AE resolved with drug discontinuation. 4) The AE recurred after the administration of the drug. 5) The AE could not be otherwise explained.

9. Other Endpoints Assessment

- Patients report mouth and throat soreness (MTS) scores (Likert scale 1 to 5) and the degree to the impact of MTS on oral activities (including swallowing, drinking, eating, talking, sleeping) (Likert scale 1 to 5) via the OMWQ. Higher score indicates more severe symptoms.
- Xerostomia is assessed according to the National Cancer Institute Common Terminology Criteria for Adverse Events (CTCAE, version 5.0) at baseline, mid-term RT (usually week 3), the end of RT, and 1- and 3-month post-RT.
- Gustatory function is assessed using "taste strips" impregnated with four basic tastes (sweet, salty, sour, and bitter) in four different concentrations at baseline, mid-term RT (usually week 3), the end of RT, and 1- and 3-month post-RT.
- QoL is assessed using the EORTC QLQ-C30 and H&N35 questionnaires at the following time points: baseline, mid-term RT (usually week 3), the end of RT, and 1-, 2-, and 3-month post-RT.
- The use of parenteral nutrition, the use of analgesics and the number of RT interruptions should be recorded during RT.
- Body weight measure at baseline and the end of RT.
- The radiographic assessment is performed every 3 months for 2 years after the end of RT.

10. Laboratory Assessment

10.1 Sample Collection

Unstimulated whole saliva will be collected by spitting method at baseline, mid-term RT (usually week 3), and at the end of RT (usually week 6 or 6.5). All samples will be frozen immediately at −20 °C and then stored at −80 °C until analysis.

10.2 DNA extraction, PCR amplification and Illumina MiSeq sequencing

Total DNA will be extracted using the FastPure DNA Isolation Kit (MJYH, Shanghai, China) according to the manufacturer's instructions. The quality and concentration of DNA are determined by 1.0% agarose gel electrophoresis and a NanoDrop 2000 spectrophotometer (Thermo Scientific, United States), and the DNA is stored at −80 °C prior to further use. The hypervariable region V3-V4 of the bacterial 16S rDNA gene is

amplified with the primer pairs 338F (5′-ACTCCTACGGGAGGCAGCAG-3′) and 806R (5′-GGACTACHVGGGTWTCTAAT-3′) using a T100 Thermal Cycler (BIO-RAD, USA). The PCR reaction mixture includes 4 μL of 5× Fast Pfu buffer, 2 μL of 2.5 mM dNTPs, 0.8 μL of each primer (5 μM), 0.4 μL of Fast Pfu polymerase, 10 ng of template DNA, and ddH_2O to a final volume of 20 μL. The PCR amplification cycling conditions are as follows: initial denaturation at 95 °C for 3 min, followed by 27 cycles of denaturation at 95 °C for 30 s, annealing at 55 °C for 30 s, and extension at 72 °C for 45 s, with a final single extension at 72 °C for 10 min and holding at 4 °C. The PCR product is extracted from a 2% agarose gel and purified using the PCR Clean-Up Kit (YuHua, Shanghai, China) according to the manufacturer's instructions, then quantified using a Qubit 4.0 fluorometer (Thermo Fisher Scientific, USA). Purified amplicons are pooled in equimolar amounts and subjected to paired-end sequencing on an Illumina NextSeq 2000 platform (Illumina, San Diego, USA) following the standard protocols of Majorbio Bio-Pharm Technology Co. Ltd. (Shanghai, China).

10.3 Data Processing

The raw 16S rDNA gene sequencing reads are demultiplexed and quality-filtered by fastp (version 0.19.6) [15] and merged by FLASH (version 1.2.11) [16] Then the high-quality sequences are denoised using DADA2 plugin [17] in the Qiime2 [18] pipeline (version 2020.2) with recommended parameters, which obtains single nucleotide resolution based on error profiles within samples. Sequences denoised by DADA2 are referred to as amplicon sequence variants (ASVs). To minimize the effects of sequencing depth on alpha and beta diversity measurements, the number of sequences from each sample is rarefied to 20,000, which still yields an average Good's coverage of 97.90%. Taxonomic assignment of ASVs is performed using the Naive Bayes consensus taxonomy classifier implemented in Qiime2 and the SILVA 16S rDNA database (v138), with a confidence threshold of 0.7.

11. Statistical Analysis

11.1 Simple Size

- The study is designed to test for superiority, with the primary endpoint being the incidence of SOM. Based on previous studies and clinical practice, about 65% of HNC patients

developed SOM during RT. We anticipated that administration of *L. reuteri* would reduce SOM incidence by 25%. Thus, 142 patients (71 per group) were required using a two-sided α level of 0.05, 80% power, and 1:1 group allocation. Considering both a 10% dropout rate and routine requirements for randomization, the sample size was determined to be 160 patients (80 per group).

11.2 Analysis Method

- All patients who complete the study (per-protocol set, PPS) and all randomly assigned patients (intention-to-treat, ITT) will be included in the efficacy analyses. Safety analyses include the patients who received at least one study medications.
- For patients who withdraw from the study, OM data collection will be continued until the point of loss to follow-up.
- For patients with persistent OM at the final follow-up visit, follow-up was extended until resolution of OM.
- Handling of missing data on OM: Data on patients who discontinue from the trial without complete follow-up of WHO OM scores are processed as follows. For patients with unresolved SOM/OM at the last assessment, the duration of their condition is estimated as the median duration among patients in the same treatment group with SOM of at least the duration observed at the last assessment. For patients without observed SOM/OM but with incomplete follow-up, duration is estimated as the median duration among patients in the same treatment group who do not experience SOM/OM for at least that follow-up time. Data of patients who occasionally miss WHO OM scores but with complete follow-up are filled in using mean imputation method.
- Sequencing analysis: Sequencing reads from all samples are standardized by rarefying the ASV tables to a minimum number of reads prior to bioinformatics analysis. Analyses are performed by using the online platform of Majorbio Cloud Platform (Majorbio, Shanghai, China) (www.majorbio.com). Sobs index and Shannon index are applied to evaluate Alpha diversity. Based on the ASV information, rarefaction curves and alpha diversity indices (Sobs index and Shannon index) are calculated using Mothur v1.30.1.21.[19] Additionally, the T-test and Wilcoxon rank-sum test are used to examine differences in alpha diversity indices and microbial relative abundances. The similarity

among the microbial communities in different samples is determined by principal coordinate analysis (PCoA) based on Bray-Curtis dissimilarity using the Vegan v2.5-3 package, and differences between microbial communities are assessed by analysis of similarities (ANOSIM). The Benjamini-Hochberg method is used to calculate the false discovery rate (FDR) for multiple comparisons, and an adjusted P-value < 0.05 is considered significant.

Appendix 1: Eastern Cooperative Oncology Group (ECOG) - Performance Status Conversion

Score	Description
0	Fully active, able to carry on all pre-disease performance without restriction.
1	Restricted in physically strenuous activity but ambulatory and able to carry out work of a light or sedentary nature (e.g., light house work, office work).
2	Ambulatory and capable of all self-care but unable to carry out any work activities. Up and about more than 50% of waking hours.
3	Capable of only limited self-care, confined to bed or chair more than 50% of waking hours.
4	Completely disabled; Cannot carry on any self-care; Totally confined to bed or chair.
5	Death.

Appendix 2: World Health Organization (WHO) Oral Toxicity Scale

Grade	Definition
0	None
1	Soreness, erythema
2	Erythema, ulcers, ability to eat solids
3	Ulcers, requiring liquid diet
4	Alimentation not possible

Appendix 3: Patient’s Diary

受试者试验产品使用日志卡

Patient’s Diary

方案名称：罗伊氏乳杆菌用于减轻头颈部恶性肿瘤患者放射性口腔黏膜炎的临床试验

Clinical Trial Name: *Limosilactobacillus reuteri* Alleviates Oral Mucositis in Patients Undergoing Radiotherapy for Malignant Head and Neck Tumors

受试者姓名: 受试开始日期：|___|___|___|___|/|___|___|/|___|___|

Subject's name： **Start Date：|___|___|___|___|/|___|___|/|___|___|**

202|_| 年 - 202|_| 年 / Year	周一 Monday	周二 Tuesday	周三 Wednesday	周四 Thursday	周五 Friday	周六 Saturday	周日 Sunday
	|_|月|_|日 Month/Day	|_|月|_|日 Month/Day	|_|月|_|日 Month/Day	|_|月|_|日 Month/Day	|_|月|_|日 Month/Day	|_|月|_|日 Month/Day	|_|月|_|日 Month/Day
早/ Morning							
晚/ Night							
202|_| 年 - 202|_| 年 / Year	|_|月|_|日 Month/Day	|_|月|_|日 Month/Day	|_|月|_|日 Month/Day	|_|月|_|日 Month/Day	|_|月|_|日 Month/Day	|_|月|_|日 Month/Day	|_|月|_|日 Month/Day
早/ Morning							
晚/ Night							
202|_| 年 - 202|_| 年 / Year	|_|月|_|日 Month/Day	|_|月|_|日 Month/Day	|_|月|_|日 Month/Day	|_|月|_|日 Month/Day	|_|月|_|日 Month/Day	|_|月|_|日 Month/Day	|_|月|_|日 Month/Day

**早/ Morning**							
**晚/ Night**							
**202|_| 年 - 202|_| 年 / Year**	|_|月|_|日 **Month/Day**	|_|月|_|日 **Month/Day**	|_|月|_|日 **Month/Day**	|_|月|_|日 **Month/Day**	|_|月|_|日 **Month/Day**	|_|月|_|日 **Month/Day**	|_|月|_|日 **Month/Day**
**早/ Morning**							
**晚/ Night**							
**202|_| 年 - 202|_| 年 / Year**	|_|月|_|日 **Month/Day**	|_|月|_|日 **Month/Day**	|_|月|_|日 **Month/Day**	|_|月|_|日 **Month/Day**	|_|月|_|日 **Month/Day**	|_|月|_|日 **Month/Day**	|_|月|_|日 **Month/Day**
**早/ Morning**							
**晚/ Night**							
**202|_| 年 - 202|_| 年 / Year**	|_|月|_|日 **Month/Day**	|_|月|_|日 **Month/Day**	|_|月|_|日 **Month/Day**	|_|月|_|日 **Month/Day**	|_|月|_|日 **Month/Day**	|_|月|_|日 **Month/Day**	|_|月|_|日 **Month/Day**
**早/ Morning**							
**晚/ Night**							
**202|_| 年 - 202|_| 年 / Year**	|_|月|_|日 **Month/Day**	|_|月|_|日 **Month/Day**	|_|月|_|日 **Month/Day**	|_|月|_|日 **Month/Day**	|_|月|_|日 **Month/Day**	|_|月|_|日 **Month/Day**	|_|月|_|日 **Month/Day**
**早/ Morning**							
**晚/ Night**							

（1） 试验含片使用时间：从放射治疗的第一天到放射治疗结束时。Duration of use: From the first day of radiotherapy to the end of radiotherapy.

（2） 使用剂量：每日两次，每次一片。Dosage: One lozenge each time, twice a day

（3） 使用方法：每日两次，早晚刷牙后试验含片在口腔里含化后吞咽。使用含片后，患者应至少 1 小时内避免进食、饮水和进行其它口腔卫生活动。Method of use: The lozenge is sucked in the morning and night after brushing the teeth. Patients should avoid eating, drinking, and conducting any oral hygiene activities for

	at least 1 hour after using the lozenge.
（4）	请在“早”、“晚”对应的空格处填写试验含片使用的具体精确到分的时间。Please fill in the spaces corresponding to "morning" and "evening" with the exact time to the minute when the lozenge is to be sucked.
（5）	填写务必准确、清晰，不得随意涂改，如发现填写内容有误，应在原记录上划单横线，在旁边写明正确内容及修改原因，并签署修改者姓名首字母缩写。不得用任何方式（橡皮、涂改液等）涂抹原记录。Please make sure to fill in the form accurately and clearly, and do not alter it at will. If an error is found in the content, a horizontal line should be drawn on the original record, and the correct content and the reason for the modification should be written next to it, and the modified person's initials should be signed. Do not smear the original record in any way (eraser, correction solution, etc.).

Appendix 4: Weekly Oral Mucositis Questionnaire (OMWQ) (English version)

1. How much MOUTH AND THROAT SORENESS did you experience in the past week?

- None
- Mild
- Moderate
- Severe
- Very Severe

2. How much did MOUTH AND THROAT SORENESS limit you in each of the following activities during the past week:

	Not at All	A Little Bit	Moderately	Quite a Bit	Cannot Do
Swallowing	O	O	O	O	O
Drinking	O	O	O	O	O
Eating	O	O	O	O	O
Talking	O	O	O	O	O
Sleeping	O	O	O	O	O

3. On a scale from 0 to 10, how would you rate your OVERALL MOUTH AND THROAT SORENESS during the past week?

Very healthy Very terrible

0 ———————————————————— 10

4. Have you used pain-killers?

- Yes (Please answer Question 5）
- No

5. If you are taking painkillers, please fill in：

Analgesic name：

Dosage：

Duration of use：

Appendix 5: Taste Strips Test

Materials and Methods: Filter paper strips with length of 8 cm and tip area of 2 cm^2 were immersed in taste-stimulating liquid and repeatedly applied to the anterior third of the tongue to identify their taste, asking to choose one of five descriptions, namely, "sweet, sour, salty, bitter, and tasteless". The test started from the low concentration, and if the subject answered the taste of the test solution correctly, the test was repeated again with the same concentration solution to ensure the accuracy of the results. Four concentrations of each stimulus were assigned a score of 4 for the lowest concentration, 1 for the highest concentration, and 0 if none of the highest concentrations could be identified. The sum of the four taste scores represented the overall gustatory function of the subjects.

The following concentrations were used for the taste strips: sweet: 0.4, 0.2, 0.1, 0.05 g/ml sucrose; sour: 0.3, 0.165, 0.09, 0.05 g/ml citric acid; salty: 0.25, 0.1, 0.04, 0.016 g/ml sodium chloride; bitter: 0.006, 0.0024, 0.0009, 0.0004 g/ml quinine hydrochloride. Distilled water was used as a solvent; taste solutions were prepared freshly at regular intervals.

Appendix 6: Assessment of Xerostomia

Grade	Definition
0	None
1	Symptomatic (e.g., dry or thick saliva) without significant dietary alteration; unstimulated saliva flow 0.2 to 0.3 ml/min.
2	Moderate symptoms; oral intake alterations (e.g., copious water, other lubricants, diet limited to purees and/or soft, moist foods); unstimulated saliva flow 0.1 to 0.2 ml/min.
3	Inability to adequately aliment orally; tube feeding or TPN indicated; unstimulated saliva flow <0.1 ml/min.

Appendix 7: European Organization for Research and Treatment of Cancer (EORTC) Quality-of-life Questionnaire Core 30 (EORTC QLQ-C30) and Head and Neck Cancer Module (EORTC QLQ-H&N35) (English version)

EORTC QLQ-C30 (version 3)

We are interested in some things about you and your health. Please answer all of the questions yourself by circling the number that best applies to you. There are no "right" or "wrong" answers. The information that you provide will remain strictly confidential.

Today's date (Day, Month, Year): |_|_|_|_|_|_|_|_|

	Not at All	A Little	Quite a Bit	Very Much
1. Do you have any trouble doing strenuous activities, like carrying a heavy shopping bag or a suitcase?	1	2	3	4
2. Do you have any trouble taking a long walk?	1	2	3	4
3. Do you have any trouble taking a short walk outside of the house?	1	2	3	4
4. Do you need to stay in bed or a chair during the day?	1	2	3	4
5. Do you need help with eating, dressing, washing yourself or using the toilet?	1	2	3	4

**During the past week:**	Not at All	A Little	Quite a Bit	Very Much
6. Were you limited in doing either your work or other daily activities?	1	2	3	4
7. Were you limited in pursuing your hobbies or other leisure time activities?	1	2	3	4
8. Were you short of breath?	1	2	3	4
9. Have you had pain?	1	2	3	4
10. Did you need to rest?	1	2	3	4
11. Have you had trouble sleeping?	1	2	3	4
12. Have you felt weak?	1	2	3	4
13. Have you lacked appetite?	1	2	3	4
14. Have you felt nauseated?	1	2	3	4
15. Have you vomited?	1	2	3	4
16. Have you been constipated?	1	2	3	4

Please go on to the next page

ENGLISH

**During the past week:**	**Not at All**	**A Little**	**Quite a Bit**	**Very Much**
17. Have you had diarrhea?	1	2	3	4
18. Were you tired?	1	2	3	4
19. Did pain interfere with your daily activities?	1	2	3	4
20. Have you had difficulty in concentrating on things, like reading a newspaper or watching television?	1	2	3	4
21. Did you feel tense?	1	2	3	4
22. Did you worry?	1	2	3	4
23. Did you feel irritable?	1	2	3	4
24. Did you feel depressed?	1	2	3	4
25. Have you had difficulty remembering things?	1	2	3	4
26. Has your physical condition or medical treatment interfered with your family life?	1	2	3	4
27. Has your physical condition or medical treatment interfered with your social activities?	1	2	3	4
28. Has your physical condition or medical treatment caused you financial difficulties?	1	2	3	4

For the following questions please circle the number between 1 and 7 that best applies to you

29. How would you rate your overall health during the past week?

1	2	3	4	5	6	7
Very poor						Excellent

30. How would you rate your overall quality of life during the past week?

1	2	3	4	5	6	7
Very poor						Excellent

© Copyright 1995 EORTC Quality of Life Group. All rights reserved. Version 3.0

ENGLISH

EORTC QLQ - H&N35

Patients sometimes report that they have the following symptoms or problems. Please indicate the extent to which you have experienced these symptoms or problems during the past week. Please answer by circling the number that best applies to you.

**During the past week:**	**Not at all**	**A little**	**Quite a bit**	**Very much**
31. Have you had pain in your mouth?	1	2	3	4
32. Have you had pain in your jaw?	1	2	3	4
33. Have you had soreness in your mouth?	1	2	3	4
34. Have you had a painful throat?	1	2	3	4
35. Have you had problems swallowing liquids?	1	2	3	4
36. Have you had problems swallowing pureed food?	1	2	3	4
37. Have you had problems swallowing solid food?	1	2	3	4
38. Have you choked when swallowing?	1	2	3	4
39. Have you had problems with your teeth?	1	2	3	4
40. Have you had problems opening your mouth wide?	1	2	3	4
41. Have you had a dry mouth?	1	2	3	4
42. Have you had sticky saliva?	1	2	3	4
43. Have you had problems with your sense of smell?	1	2	3	4
44. Have you had problems with your sense of taste?	1	2	3	4
45. Have you coughed?	1	2	3	4
46. Have you been hoarse?	1	2	3	4
47. Have you felt ill?	1	2	3	4
48. Has your appearance bothered you?	1	2	3	4

Please go on to the next page

ENGLISH

**During the past week:**	**Not at all**	**A little**	**Quite a bit**	**Very much**
49. Have you had trouble eating?	1	2	3	4
50. Have you had trouble eating in front of your family?	1	2	3	4
51. Have you had trouble eating in front of other people?	1	2	3	4
52. Have you had trouble enjoying your meals?	1	2	3	4
53. Have you had trouble talking to other people?	1	2	3	4
54. Have you had trouble talking on the telephone?	1	2	3	4
55. Have you had trouble having social contact with your family?	1	2	3	4
56. Have you had trouble having social contact with friends?	1	2	3	4
57. Have you had trouble going out in public?	1	2	3	4
58. Have you had trouble having physical contact with family or friends?	1	2	3	4
59. Have you felt less interest in sex?	1	2	3	4
60. Have you felt less sexual enjoyment?	1	2	3	4

**During the past week:**	**No**	**Yes**
61. Have you used pain-killers?	1	2
62. Have you taken any nutritional supplements (excluding vitamins)?	1	2
63. Have you used a feeding tube?	1	2
64. Have you lost weight?	1	2
65. Have you gained weight?	1	2

© Copyright QLQ-H&N35 1994 EORTC Quality of Life Group, version 1. All rights reserved

Appendix 8: National Cancer Institute Common Terminology Criteria for Adverse Events (NCI CTCAE) version 5.0

For comprehensive details, please refer to the complete standard available at NCI CTCAE v5.0 or access the online information at the following NCI website: https://ctep.canCEr.gov/protocolDevelopment/electronic_applications/ctc.htm.

Amendments

Table of Changes

Version	Date	Section No.	Section Title	Description of Change (s)	Rationale
1.0	2023-08-05	All sections	General	Original protocol.	NA
2.0	2025-11-10	3.2; 7.2	Exclusion Criteria; Oral Mucositis Assessment	1. Added detailed definitions of poor oral hygiene in the exclusion criteria.<br>Original: Poor oral hygiene and/or severe periodontal diseases;<br>Revised: As assessed by a qualified dentist/oral physician, the presence of poor oral hygiene (a simplified oral hygiene index (OHI-S) score of ⩾ 3), and/or severe periodontal disease, defined as at least two teeth with a probing pocket depth (PPD) of ⩾ 6 mm, clinical attachment loss (CAL) of ⩾ 5 mm, and bleeding on probing (BOP) in at least two non-adjacent teeth;<br>*The Simplified Oral Hygiene Index (OHI-S) comprises the Debris Index (DI-S) and the Calculus Index (CI-S). Each index is scored from 0 to 3 based on the area of tooth surface coverage: a score of 0 indicates no debris or calculus; 1 indicates debris covering no more than one-third of the tooth surface or supragingival calculus covering only a fleck of the surface; 2 indicates debris or calculus covering more than one-third but less than two-thirds; and a score of 3 indicates debris or calculus covering two-thirds or more of the tooth surface. The examination is performed on the buccal and lingual surfaces of six designated index teeth, and the final OHI-S score is the average of all DI-S and CI-S scores.<br>2. Included a figure legend illustrating the follow-up schedule.<br>Revised:<br>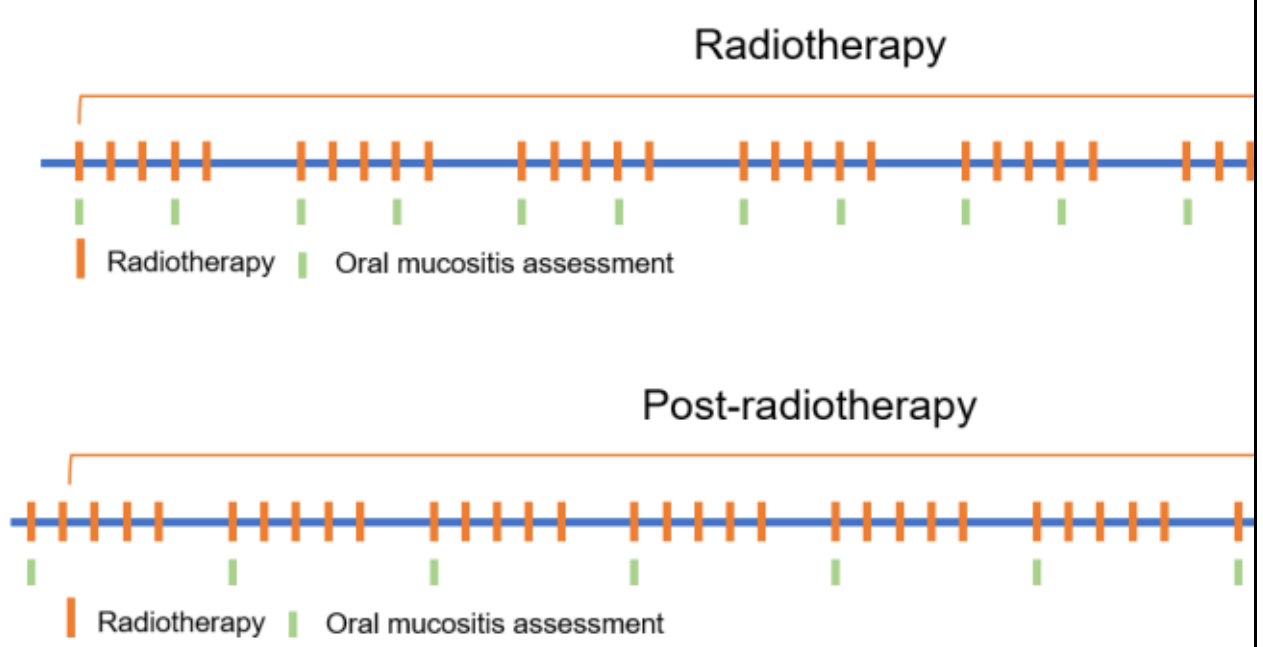	To enhance clarity and reproducibility of the study protocol.
3.0	2026-04-10	4.1; 7.2	Screening; Oral Mucositis Assessment	1. Added detailed definitions of poor oral hygiene in the exclusion criteria.<br>Original: Dental examination including oral hygiene, caries, periodontal disease and impacted tooth etc.<br>Revised: Standardized oral hygiene refers to that all patients underwent a professional dental cleaning (scaling and polishing) during the screening period. And all patients were instructed to follow a standardized daily oral care regimen throughout radiotherapy, which comprised brushing with a soft-bristled toothbrush at least twice daily, performing gentle flossing once daily, and rinsing	To enhance clarity and reproducibility of the study protocol.

				the mouth with water after each meal. Oral hygiene education was provided by trained study personnel during the study screening period, and adherence to the oral hygiene regimen was reviewed and reinforced at each scheduled follow-up visit during the entire study course. Specifically, after using the lozenge, the patients should avoid eating, drinking and conducting any oral hygiene activities for at least 1 hour.<br>2. Revised the figure legend illustrating the follow-up schedule.<br>Original:<br>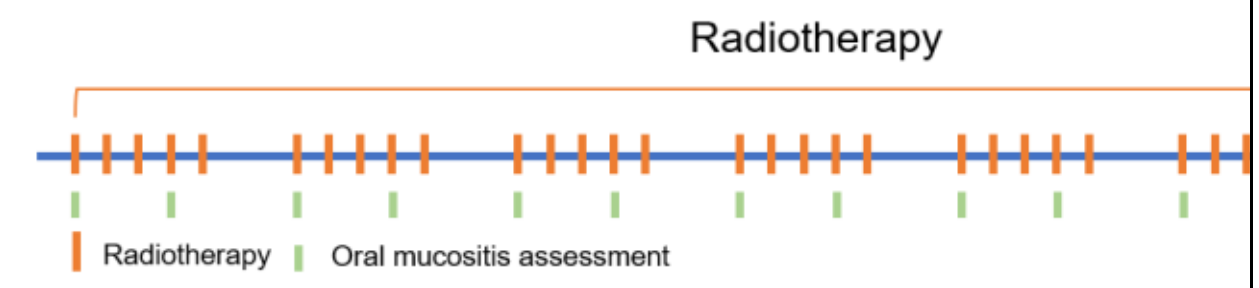<br>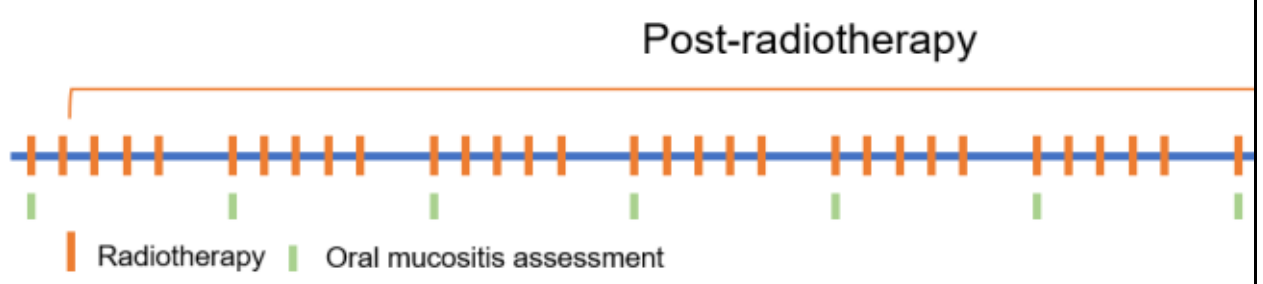<br>Revised:<br>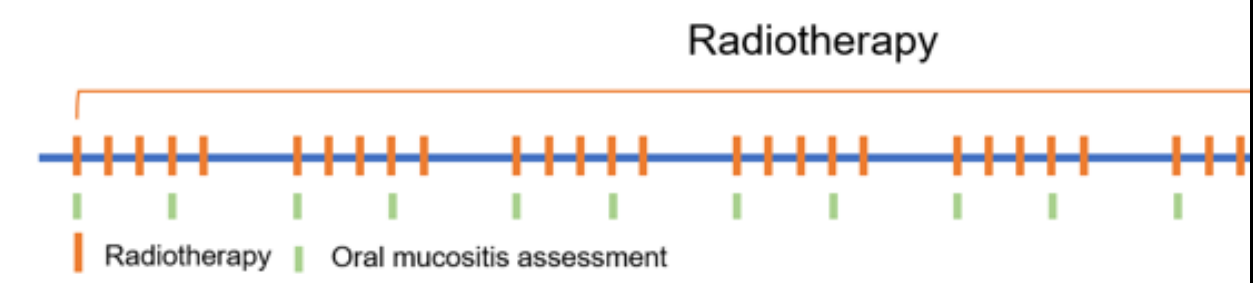<br>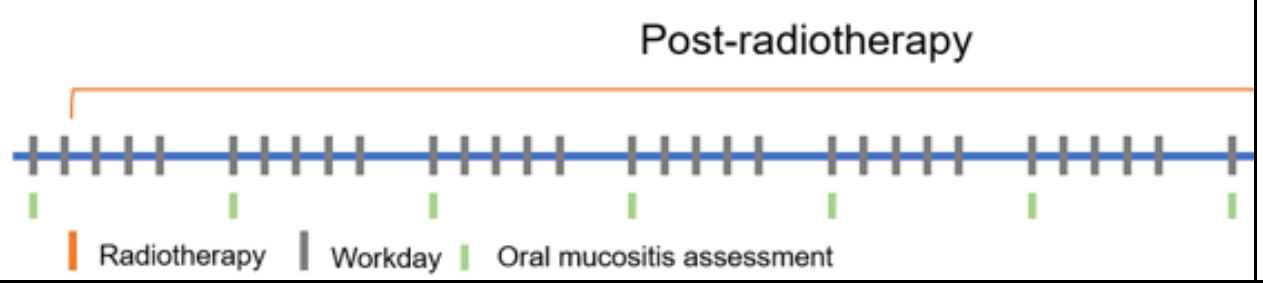	

References

1. Anderson, C.M., *et al.* Phase IIb, Randomized, Double-Blind Trial of GC4419 Versus Placebo to Reduce Severe Oral Mucositis Due to Concurrent Radiotherapy and Cisplatin For Head and Neck Cancer. *J Clin Oncol* **37**, 3256-3265 (2019).
2. Blijlevens, N. & Sonis, S. Palifermin (recombinant keratinocyte growth factor-1): a pleiotropic growth factor with multiple biological activities in preventing chemotherapy- and radiotherapy-induced mucositis. *Ann Oncol* **18**, 817-826 (2007).
3. Elad, S., Yarom, N., Zadik, Y., Kuten-Shorrer, M. & Sonis, S.T. The broadening scope of oral mucositis and oral ulcerative mucosal toxicities of anticancer therapies. *CA Cancer J Clin* **72**, 57-77 (2022).
4. Elad, S., *et al.* MASCC/ISOO clinical practice guidelines for the management of mucositis secondary to cancer therapy. *Cancer* **126**, 4423-4431 (2020).
5. Belkaid, Y. & Hand, T.W. Role of the microbiota in immunity and inflammation. *Cell* **157**, 121-141 (2014).
6. Mu, Q., Tavella, V.J. & Luo, X.M. Role of Lactobacillus reuteri in Human Health and Diseases. *Front Microbiol* **9**, 757 (2018).
7. Naureen, Z., *et al.* Polyphenols and Lactobacillus reuteri in oral health. *J Prev Med Hyg* **63**, E246-e254 (2022).
8. Talarico, T.L., Casas, I.A., Chung, T.C. & Dobrogosz, W.J. Production and isolation of reuterin, a growth inhibitor produced by Lactobacillus reuteri. *Antimicrob Agents Chemother* **32**, 1854-1858 (1988).
9. Szkaradkiewicz, A.K., Stopa, J. & Karpiński, T.M. Effect of oral administration involving a probiotic strain of Lactobacillus reuteri on pro-inflammatory cytokine response in patients with chronic periodontitis. *Arch Immunol Ther Exp (Warsz)* **62**, 495-500 (2014).
10. İnce, G., *et al.* Clinical and Biochemical Evaluation of Lozenges Containing Lactobacillus reuteri as an Adjunct to Non-Surgical Periodontal Therapy in Chronic Periodontitis. *J Periodontol* **86**, 746-754 (2015).
11. Teughels, W., *et al.* Clinical and microbiological effects of Lactobacillus reuteri probiotics in the treatment of chronic periodontitis: a randomized placebo-controlled study. *J Clin Periodontol* **40**, 1025-1035 (2013).
12. Iniesta, M., *et al.* Probiotic effects of orally administered Lactobacillus reuteri-containing tablets on the subgingival and salivary microbiota in patients with gingivitis. A randomized clinical trial. *J Clin Periodontol* **39**, 736-744 (2012).
13. Krasse, P., *et al.* Decreased gum bleeding and reduced gingivitis by the probiotic Lactobacillus reuteri. *Swed Dent J* **30**, 55-60 (2006).
14. Mir, R., *et al.* Organ at risk delineation for radiation therapy clinical trials: Global Harmonization Group consensus guidelines. *Radiother Oncol* **150**, 30-39 (2020).
15. Chen, S., Zhou, Y., Chen, Y. & Gu, J. fastp: an ultra-fast all-in-one FASTQ preprocessor. *Bioinformatics* **34**, i884-i890 (2018).
16. Magoč, T. & Salzberg, S.L. FLASH: fast length adjustment of short reads to improve genome assemblies. *Bioinformatics* **27**, 2957-2963 (2011).
17. Callahan, B.J., *et al.* DADA2: High-resolution sample inference from Illumina amplicon data. *Nat Methods* **13**, 581-583 (2016).
18. Bolyen, E., *et al.* Reproducible, interactive, scalable and extensible microbiome data science using QIIME 2. *Nat Biotechnol* **37**, 852-857 (2019).
19. Schloss, P.D., *et al.* Introducing mothur: open-source, platform-independent, community-supported software for describing and comparing microbial communities. *Appl Environ Microbiol* **75**, 7537-7541 (2009).
