# Supplementary material for: Limosilactobacillus reuteri for oral mucositis in patients undergoing radiotherapy for malignant head and neck tumors: a randomized controlled trial
Source: Nat Commun. 2026 Sep 29;17:11226. doi: 10.1038/s41467-026-78088-6 (PMC13720049; doi:10.1038/s41467-026-78088-6)
Supplement: Supplementary file 2 [file 41467_2026_78088_MOESM2_ESM.pdf]

nature portfolio

Corresponding author(s): Dr Xingchen Peng

Last updated by author(s): Apr 2, 2026

Reporting Summary

Nature Portfolio wishes to improve the reproducibility of the work that we publish. This form provides structure for consistency and transparency in reporting. For further information on Nature Portfolio policies, see our Editorial Policies and the Editorial Policy Checklist.

Statistics

For all statistical analyses, confirm that the following items are present in the figure legend, table legend, main text, or Methods section.

n/a	Confirmed	
☐	☒	The exact sample size (*n*) for each experimental group/condition, given as a discrete number and unit of measurement
☐	☒	A statement on whether measurements were taken from distinct samples or whether the same sample was measured repeatedly
☐	☒	The statistical test(s) used AND whether they are one- or two-sided<br>*Only common tests should be described solely by name; describe more complex techniques in the Methods section.*
☐	☒	A description of all covariates tested
☐	☒	A description of any assumptions or corrections, such as tests of normality and adjustment for multiple comparisons
☐	☒	A full description of the statistical parameters including central tendency (e.g. means) or other basic estimates (e.g. regression coefficient) AND variation (e.g. standard deviation) or associated estimates of uncertainty (e.g. confidence intervals)
☐	☒	For null hypothesis testing, the test statistic (e.g. *F*, *t*, *r*) with confidence intervals, effect sizes, degrees of freedom and *P* value noted<br>*Give P values as exact values whenever suitable.*
☒	☐	For Bayesian analysis, information on the choice of priors and Markov chain Monte Carlo settings
☐	☒	For hierarchical and complex designs, identification of the appropriate level for tests and full reporting of outcomes
☒	☐	Estimates of effect sizes (e.g. Cohen's *d*, Pearson's *r*), indicating how they were calculated

Our web collection on statistics for biologists contains articles on many of the points above.

Software and code

Policy information about availability of computer code

Data collection	Clinical data were collected using case report forms.
Data analysis	Statistical analyses were performed using R version 4.4.0 (R Foundation for Statistical Computing) and GraphPad Prism version 9.5 (GraphPad Software). Microbiome profiles were analyzed using the Majorbio Cloud platform.

For manuscripts utilizing custom algorithms or software that are central to the research but not yet described in published literature, software must be made available to editors and reviewers. We strongly encourage code deposition in a community repository (e.g. GitHub). See the Nature Portfolio guidelines for submitting code & software for further information.

Data

Policy information about availability of data

All manuscripts must include a data availability statement. This statement should provide the following information, where applicable:

- Accession codes, unique identifiers, or web links for publicly available datasets
- A description of any restrictions on data availability
- For clinical datasets or third party data, please ensure that the statement adheres to our policy

All raw sequencing data generated in this study have been deposited in the National Genomics Data Center (NGDC) under the accession code HRA016457 (https://ngdc.cncb.ac.cn/gsa-human/browse/HRA016457). The de-identified individual patient data and sequencing data are available under restricted access within 3 years after the publication of this paper due to privacy protection and ethical restrictions related to participant confidentiality. Access can be obtained upon reasonable academic request by contacting Professor Xing-chen Peng, the corresponding author (pxx2014@163.com). The researcher will also be required to sign a data access

agreement with West China Hospital, Sichuan University. The remaining data are available within the Article, Supplementary Information or Source Data file.

Research involving human participants, their data, or biological material

Policy information about studies with human participants or human data. See also policy information about sex, gender (identity/presentation), and sexual orientation and race, ethnicity and racism.

Reporting on sex and gender	Gender was not limited in this study. Gender of participants was determined based on self-report.
Reporting on race, ethnicity, or other socially relevant groupings	This study was not involved with the constructs of race and/or ethnicity.
Population characteristics	Eligible patients were pathologically confirmed head and neck cancer patients without distant metastasis, aged 18 to 80 years, with an Eastern Cooperative Oncology Group (ECOG) performance status ≤ 2, and with a planned radiotherapy regimen (60-72 Gy) alone or with chemotherapy.
Recruitment	Patients were recruited and screened at West China Hospital of Sichuan University. No known bias for recruitment was identified.
Ethics oversight	Institutional Review Board at West China Hospital of Sichuan University.

Note that full information on the approval of the study protocol must also be provided in the manuscript.

Field-specific reporting

Please select the one below that is the best fit for your research. If you are not sure, read the appropriate sections before making your selection.

[x] Life sciences [ ] Behavioural & social sciences [ ] Ecological, evolutionary & environmental sciences

For a reference copy of the document with all sections, see nature.com/documents/nr-reporting-summary-flat.pdf

Life sciences study design

All studies must disclose on these points even when the disclosure is negative.

Sample size	Based on previous studies and clinical practice, about 65% of head and neck cancer patients developed SOM during radiotherapy. We anticipated that administration of L. reuteri would reduce SOM incidence by an absolute margin of 25% (from 65% to 40%). Accordingly, a sample size of 142 patients (71 per group) was required, calculated using PASS 2021 (version 21.0.3) with a two-sided α level of 0.05, 80% power, and 1:1 group allocation. After accounting for a 10% dropout rate and considerations for randomization balance, the final sample size was set at 160 patients (80 per group).
Data exclusions	A total of 203 patients were assessed for eligibility. Forty-three patients were excluded from the analysis.
Replication	Since this is a single-center clinical trial, direct biological replication is not applicable. However, to ensure reproducibility of the findings, we followed a pre-registered protocol (NCT06285591) and used standardized operating procedures for data collection. Independent replication in a separate cohort would be required to confirm the results.
Randomization	Eligible patients were allocated equally (1:1) to receive L. reuteri or placebo via a computerized block randomization system with permutation method.
Blinding	Pharmacists who were blinded to the trial assignment prepackaged L. reuteri and placebo in identical containers. The lozenges were allocated based on random numbers. The allocation order list, block size, and random numbers were sealed in opaque envelopes.

Reporting for specific materials, systems and methods

We require information from authors about some types of materials, experimental systems and methods used in many studies. Here, indicate whether each material, system or method listed is relevant to your study. If you are not sure if a list item applies to your research, read the appropriate section before selecting a response.

Materials & experimental systems

n/a	Involved in the study
☒	☐ Antibodies
☒	☐ Eukaryotic cell lines
☒	☐ Palaeontology and archaeology
☐	☒ Animals and other organisms
☐	☒ Clinical data
☒	☐ Dual use research of concern
☒	☐ Plants

Methods

n/a	Involved in the study
☒	☐ ChIP-seq
☒	☐ Flow cytometry
☒	☐ MRI-based neuroimaging

Animals and other research organisms

Policy information about studies involving animals; ARRIVE guidelines recommended for reporting animal research, and Sex and Gender in Research

Laboratory animals	Twenty-one healthy male C57BL/6 mice were used in this study and randomly assigned to three groups (n = 7 per group): Control, Saline + irradiation (IR), and L. reuteri + IR.
Wild animals	The study did not involve wild animals.
Reporting on sex	Only male mice were used in this study.
Field-collected samples	The study did not involve field-collected samples.
Ethics oversight	All animal experiments were approved by the Institutional Animal Care and Use Committee (IACUC) of West China Hospital of Sichuan University (approval no. 20230923070) and performed in accordance with the National Institutes of Health (NIH) Guide for the Care and Use of Laboratory Animals.

Note that full information on the approval of the study protocol must also be provided in the manuscript.

Clinical data

Policy information about clinical studies

All manuscripts should comply with the ICMJE guidelines for publication of clinical research and a completed CONSORT checklist must be included with all submissions.

Clinical trial registration	NCT06285591.
Study protocol	All versions of the trial protocol have been submitted as supplementary material (versions 1.0–3.0).
Data collection	Data were collected at the Department of Radiation Oncology, West China Hospital of Sichuan University (Chengdu, China). Recruitment took place from March 2024 to January 2025. All data were collected during radiotherapy and the follow-up period after radiotherapy completion. Data sources included electronic medical records, patient-reported outcome questionnaires, and assessments performed by blinded outcome assessors.
Outcomes	The incidence of SOM (≥ grade 3 per WHO Oral Toxicity criteria) was the primary end point. Secondary end points included the onset and duration of SOM, as well as the incidence, onset, and duration of any-grade OM. The exploratory end point included quality of life, mouth and throat soreness score, hyposalivation, gustatory function, change in weight, use of parenteral nutrition and analgesics, radiotherapy interruption rate, safety, and the composition and dynamics of salivary microbiota.

Plants

Seed stocks	*Report on the source of all seed stocks or other plant material used. If applicable, state the seed stock centre and catalogue number. If plant specimens were collected from the field, describe the collection location, date and sampling procedures.*
Novel plant genotypes	*Describe the methods by which all novel plant genotypes were produced. This includes those generated by transgenic approaches, gene editing, chemical/radiation-based mutagenesis and hybridization. For transgenic lines, describe the transformation method, the number of independent lines analyzed and the generation upon which experiments were performed. For gene-edited lines, describe the editor used, the endogenous sequence targeted for editing, the targeting guide RNA sequence (if applicable) and how the editor was applied.*
Authentication	*Describe any authentication procedures for each seed stock used or novel genotype generated. Describe any experiments used to assess the effect of a mutation and, where applicable, how potential secondary effects (e.g. second site T-DNA insertions, mosiacism, off-target gene editing) were examined.*
