# Supplementary material for: Limosilactobacillus reuteri for oral mucositis in patients undergoing radiotherapy for malignant head and neck tumors: a randomized controlled trial
Source: Nat Commun. 2026 Sep 29;17:11226. doi: 10.1038/s41467-026-78088-6 (PMC13720049; doi:10.1038/s41467-026-78088-6)
Supplement: Supplementary file 3 [file 41467_2026_78088_MOESM3_ESM.pdf]

nature portfolio

Peer Review File

Limosilactobacillus reuteri for Oral Mucositis in Patients Undergoing Radiotherapy for Malignant Head and Neck Tumors: A Randomized Controlled Trial

Corresponding Author: Professor Xingchen Peng

Parts of this Peer Review File have been redacted as indicated to remove third-party material.

This file contains all reviewer reports in order by version, followed by all author rebuttals in order by version.

Version 0:

Reviewer comments:

Reviewer #1

(Remarks to the Author)
This manuscript reports a double-blind, placebo-controlled randomized phase II trial evaluating Lactobacillus reuteri lozenges for the prevention and mitigation of radiation-induced oral mucositis (RIOM) in patients with head and neck cancer. The authors demonstrate a statistically significant reduction in the incidence, delayed onset, and shortened duration of severe oral mucositis (SOM), accompanied by favorable microbiome changes and an acceptable safety profile.

Major comments

1. Conceptual and narrative redundancy with prior work

The authors have previously published a randomized controlled trial of Streptococcus salivarius K12 for RIOM (J Clin Oncol, 2024), which is cited. While the present study evaluates a different probiotic strain, the conceptual framing, endpoint hierarchy, manuscript structure, and discussion logic are strikingly similar, with the probiotic species effectively substituted within an otherwise unchanged narrative. This degree of structural and rhetorical overlap diminishes perceived novelty and is problematic for a high-impact, general-interest journal.

2. Limited translational positioning

Despite repeated references (in the Abstract, Introduction, Results, and Discussion) to the authors' previous basic and translational research, no specific foundational or mechanistic references are provided. Furthermore, the translational relevance remains limited for a journal such as Nature Communications, with no functional validation beyond 16S rDNA profiling.
While Figure 4 provides valuable associative evidence that Lactobacillus reuteri administration modulates the oral microbiome during radiotherapy, the data remain descriptive and taxonomic. The manuscript would benefit from a more cautious interpretation, as no functional or causal link between specific microbial changes and clinical outcomes is demonstrated.

3. Trial reporting and transparency

• The clinical trial registration number should be included in the Abstract, in accordance with CONSORT and journal standards.
• Nineteen patients did not complete or receive the experimental intervention. The reasons for non-receipt or discontinuation require clearer explanation, including timing and potential impact on outcome interpretation.

4. Radiotherapy-related considerations

• The authors should briefly discuss the influence of tumor localization (nasopharynx, oral cavity, oropharynx, hypopharynx,

larynx) on dose delivered to the oral cavity, a major determinant of mucositis risk.
• Radiotherapy doses are reported in cGy, whereas standard clinical convention uses Gy. Units should be standardized throughout.

5. Supportive care and plausibility of reported data

• The reported 54% rate of parenteral nutrition appears unexpectedly high and requires clarification.
• The manuscript should explicitly confirm that no other oral agents, probiotics, mouthwashes, antimicrobial products, or experimental supportive treatments were administered during radiotherapy.

6. Oncological outcomes and safety reporting

• Although this is a supportive care trial, oncological outcomes (e.g., locoregional control, early progression, treatment interruptions) should be briefly described in both arms.
• Toxicities should be reported with explicit CTCAE grading rather than aggregated events.

7. Compliance with the experimental intervention

• Treatment compliance (e.g., proportion of prescribed lozenges consumed, adherence over time) should be reported, as this is essential for interpreting prophylactic efficacy.

8. Figure 3

Figure 3 presents data only during radiotherapy. The absence of post-radiotherapy mucositis follow-up is a limitation and should be justified or acknowledged, as mucositis often persists beyond treatment completion.

Minor comments

Table 1 indicates that approximately 50% of patients were non-smokers and non-drinkers, which differs markedly from typical European head and neck cancer populations. Given the predominance of nasopharyngeal carcinoma, the authors should discuss etiological differences (e.g., EBV, HPV status) and limitations in generalizability to Western populations. If available, HPV or viral status data should be reported or explicitly acknowledged as unavailable.

The Discussion claims better acceptability of L. reuteri compared with palifermin, yet no supporting data or references are provided.

Clarify the exact L. reuteri strain(s) used (e.g., DSM 17938, ATCC PTA 5289).

Report absolute risk reductions alongside relative risks in the Abstract.

Consider adding a schematic summarizing hypothesized mechanisms.

Reduce repetitive background statements in the Discussion.

Reviewer #2

(Remarks to the Author)
This manuscript describes a phase II double blinded randomized clinical trial evaluating the capability of L. reuteri to reduces severe oral mucositis (SOM) in patients with HNSCC treated with radiotherapy.
The researchers included 160 eligible patients in a single center from march 2024 till January 2025.
The primary endpoint was incidence of SOM, defined as grade 3 or higher on the WHO oral toxicity score. The mucositis was scored twice a week during radiotherapy and once a week till 8 weeks after radiotherapy.
They found a significant reduction in SOM from 61.3% in the control group to 37.5% in the intervention group. The duration of SOM/OM was significantly shorter and the onset was delayed.
This reduction of SOM also led to clinical significant reduction of toxicity burden reflected by lower use of parental feeding and lower use of analgesics.

Novelty: this is the first RCT on the use of L reuteri to prevent SOM in HNSCC radiotherapy patients.

Comments:
This is a very clear and nicely described RCT. The findings are highly relevant for the field.
Major remarks:
- Was the primary outcome scored by a single observer, or by many? And where the scores done by a single observer at each time point, or was there a double check? Please describe this more in detail.
- Was there a control mechanism on the administration, I could imagine that patients stop using the medication when the suffer from SOM. Please clarify
- There is a clear correlation between SOM and dental fillings, was there a correction for that, or a standard protocol for patients with dental fillings? Please clarify
- Please describe the standard protocol for oral hygiene, was that similar between both groups? Please clarify

- Smoking was noted in table 1, with half of the patients smoked, were the results corrected for smoking and alcohol use? Please clarify
- How many patients developed oropharyngeal candidiasis, and how was this managed? Were patients excluded from the trial? And the same for concurrent use of antibiotics for other infections (pneumonia, etc) how were these patients managed in the trial?

Reviewer #3

(Remarks to the Author)
Thank you for the opportunity to review this piece of work. You provide compelling evidence demonstrating the mucoprotection achieved by an oral probiotic (L Reuteri), delivered as a lozenge, in people undergoing radiation (+/- chemotherapy) for head and neck cancer. This work tackles a longstanding, common yet often overlooked problem in head and neck cancer, and hence, the significance of this work cannot be underestimated. I sincerely want to thank you for your level of commitment to this major clinical problem. Overall, the manuscript is well presented, the study is rigorously designed and executed and the findings are of interest to the supportive oncology community. However, the manuscript itself is somewhat superficial is its analyses and interpretation, and would benefit from a deeper level of thought and interpretation, particularly with respect to existing work (published by you/your group) on another probiotic, Ssk12. Below are some points that I feel need to be acknowledged in your work before it can be considered for publication.

Major:
1) The justification for the use of this specific strain requires more explanation, specifically related to its role in OM pathogenesis. I not you mentioned "In addition, our basic research showed that L reuteri has efficacy in managing RIOM" (Line 79), however there is no reference. Are you referring to the data presented in this manuscript, or past data? If the data in this manuscript, I would suggest removing this from the introduction as you cannot justify a strain selected based on not yet generated preclinical data. If it has been published, please reference.
2) Following from point 1, the biggest question I have with this work is why the pivot from Ssk12. You have previously shown that Ssk12 was clinically effective in reducing RIOM (published din JCO, 2023). So what is the justification for testing a new strain? Please include a detailed justification of the rationale of testing a new strain, rather than pushing forward with implementing Ssk12 after your highly successful Phase II trial.
3) Were randomisation schedules stratified in any way? e.g. by inclusion of chemotherapy, diagnostic criteria, HPV status?
4) The quality of life tool was performed repeatedly over the course of the study (weekly). This is quite high burden on participants. Did you see much attrition in the rate of completion? How were missing data handled?
5) Saliva collection was achieved by spitting. Based on my experience with this patient population, it is extremely difficult to collect saliva after week 3 of treatment due to xerostomia. Can you please comment, or include more information on how saliva collection was achieved in cases where xerostomia was present. Also please include the number saliva samples collected per participant per timepoints, separated by treatment group. I am curious as to whether there are biases creeping into your microbiome data based on selected saliva sampling.
6) How were confounders handled, especially HPV status and smoking status. Please review this paper on how to handle mucositis confounders: https://pubmed.ncbi.nlm.nih.gov/35639187/ and incorporate appropriate methodology. At the very least, reporting HPV+/- rates in your treatment groups would be needed. Similarly, stratification or your data based on the addition of chemotherapy would be interesting. Do you see different efficacy signals in radiation vs chemoradiation?
7) Your microbiome data is interesting - were you surprised at the subtleness of the changes observed in your placebo group. It certainly doesnt appear that there is profound "dysbiosis" occurring. If anything, the shifts look more apparent in your probiotic group. Please comment. In addition, there is considerable divergence in the baseline alpha diversity between probiotic and placebo. This is concerning and certainly needs to be discussed in your paper.
8) There were minimal differences in the QoL outcomes between your experimental groups, with the exception of pain. Can you please comment on this. I would hypothesise that such a profound improvement in mucositis would translate into a positive QoL signal. In the absence of such signal, it is difficult to justify why this probiotic should be taken forward. Alternatively, you may like to consider flow on effects of controlling mucositis on clinical service provision. Is this something that can be extracted from clinical records? e.g. emergency presentations, admissions, length of stay, radiation dose reductions or modifications, insertion of a feeding tube, infection, supportive care medication use (e.g. not just opioids, but also antibiotics). Could you use this to model the economic benefit of your intervention?
9) You mention in your methods that both IIT and PP analyses were performed. In the results, it is unclear which has been used. Can you please report on both in your results, or make it explicitly clear what analysis was used.
10) Why was the onset of(dats) for RIOM not estimable in your intervention group (Table 2)?
11) I am extremely surprised at the very low rate of drop out. In my experience, this is a complex patient cohort with high needs. Can you please comment on why you had such low drop out. This is especially surprising in the placebo group who had no benefit. In my experience, when people are required to adhere to such a long intervention program (like this) and experience no benefit, the rate of drop out is high, or at least, the rate of adherence is low. Can you please comment on this.
12) Following on from point 11 - there is no mention or discussion of adherence to the intervention. Was this recorded. Please include data on this. This is especially important as, in the context of mucositis and xerostomia, the feasibility of consuming sticky lozenges each day for 6-7 weeks seems challenging. Adherence should be considered in y our ITT vs PP analyses. In line with this, I acknowledge that you reviewed Lactobacillus abundance as an indication of LReuteri colonisation, however feel this could have been achieved with greater precision. Could you perform targeted PCR for the probiotic strain in saliva samples to truly determine colonisation. This again may assist in more nuanced analyses, where efficacy could be strategies by the rate/completeness of colonisation.
13) The discussion would benefit from less repetition of results and more mechanistic thought / forward gazing. One key observation I have made from these data is that the efficacy signal is near identical to that you report in your JCO paper on

Ssk12. There needs to be discussion on what this means mechanistically. To me, and as written in my JCO UTP paper (Wardill, Sonis, Bossi, 2024), there is a distinct possibility that the efficacy of both Ssk12 and LR are related not to the strain, but simply the sheer presence of a probiotic micro-organism that is capable of displacing or restricting pathogen blooms. Please comment on this, and relate your findings to your Ssk12 data more explicitly, particularly in terms of translating these promising clinical findings into a standard of care clinical intervention. Which one would you choose? Should you even choose one? Would there be greater efficacy if they were combined? Similarly - you could consider the concept of responder/non-responder, i.e. were there microbial attributes at baseline that dictate response?
14) The preclinical model showed almost complete prevention of OM. This is extremely challenging to achieved and may indeed be cause for concern if it is impacting radiation toxicity so profoundly ... is it therefore influencing efficacy? I would recommend reviewing/including clinical data on treatment response, i.e. RECiST at 3 months post treatment to confirm your probiotic is not influencing radiation efficacy. Similarly, the level of protection you see in your preclinical model is much higher than what you see clinically. This should be discussed in light of the dose justification (both mice and men receiving the same dose of probiotic - thus relative to body size, the mice are getting a lot more microbes. Does this explain the higher efficacy seen in mice?).
15) What is the justification of stopping the animal model at day 8? What happens beyond day 8? Perhaps you havent totally prevented OM, but instead delayed its onset?

Minor:
1) The term dysbiosis is used throughout this manuscript and while I appreciate its convenience, it is increasingly scrutinised for its lack of specificity. Hence I strongly suggest amending your language throughout to conform with modern recommendations. https://pmc.ncbi.nlm.nih.gov/articles/PMC7017827/
2) You may like to consider including this reference to support the role of the microbiome in OM - this was published by MASCC as an addendum to their pathogenesis framework. I transparently disclose that I am a co-author on this manuscript. https://link.springer.com/article/10.1007/s00520-024-08752-4
3) Some figures and tables would benefit from re-ordering of data, e.g, it feels awkward to have the intervention before the placebo, and much more natural to present placebo first. Similarly, in Figure 4e, starting with T2>>T0 (left to right) feels counterintuitive.
4) The rate of opioid use seems to be very low. In our experience, this patient group has rates of opioids use >90%. Please comment on this.
5) Please provide more clarity on why N=7 mice/group were used, but then downstream analyses are only performed on N=3-4. How were the subgroups defined/selected?

Reviewer #4

(Remarks to the Author)

This is a high-significant, well-designed clinical trial that assessed the efficacy of Lactobacillus reuteri for prevention of radiation-induced oral mucositis (OM) in head and neck cancer patients. The trial was randomized and double-blinded in concordance with the CONSORT guidelines. The study found to an intervention with Lactobacillus reuteri containing lozenges twice a day to substantially and significantly reduce incidence, time-to-onset and duration as well as severity of OM, which is if replicated in other cohorts, can transform clinical management of head and neck cancer patients undergoing radiotherapy.

The manuscript is well written and organized. The methods are sufficiently described in the main text and supplementary material. Patients' clinical characteristics and results of analysis of primary, secondary and exploratory outcomes are adequately and well presented, although it is recommended to present results from adjusted analysis in the main text and move unadjusted results to the supplementary.

It is important to note that Lactobacillus reuteri has been reclassified as Limosilactobacillus reuteri, so the nomenclature should be updated in the manuscript accordingly. Moreover, a recent randomized clinical trial (PMID: 40887814) reported limited efficacy of L. reuteri in a similar clinical context. The study should be acknowledged in the introduction and discussed in comparison with the findings of the present study.

While the trial is reported to have been registered under the identifier NCT06285591, no corresponding record can be found on ClinicalTrials.gov. This discrepancy should be clarified by the authors.

This reviewer has major concerns with respect to the microbiome component of manuscript as follows:
1- Substantial biological data is lost by restricting the analysis to the genus level. Using appropriate classification algorithm, e.g. blastn, and reference database (such as the Human Oral Microbiome Database, HOMD), ASVs generated from V3-4 sequencing can be reliably classified to the species level, which is more biologically meaningful for interpreting association with mucosal health and disease, as health and disease associated species frequently belong to the same genus.

2- The bioinformatic analysis pipeline used is suboptimal. In addition to the low taxonomic resolution, the methods used for beta diversity (UniFrac distances) and differential abundance analysis (LEfSe) are not compositional data aware (CDA). In addition, LEfSe does not appropriately account for multiple testing, as it does not report false discovery rate (FDR)–adjusted results. It is highly recommended to reanalyze the data using appropriate CDA methods such as Aitchison distances calculated from CLR transformed data for beta diversity and CLR transformed data with regression models adjusting for both confounders and FDR (such as MaAslin) for differential abundance analysis.

3- Data analysis lacks sufficient depth and integration with the clinical findings. For example, no comparisons of the microbiome were made between patients who developed and didn't develop OM/SOM, both overall and within each study arm. This is particularly important since beta-diversity results presented in the supplementary materials show a significant difference in baseline microbiomes between the two study arms, despite randomization and comparable baseline clinical characteristics. This raises concern that observed differences in clinical outcomes may be driven, at least in part, by underlying microbiome differences rather than the intervention itself. Accordingly, the microbiome may need to be included as a variable in the adjusted analysis. Furthermore, while the PCoA plots indicate significant microbial community composition between timepoints in each study arm, the specific microbial shifts at the genus and species levels are not described or correlated with outcomes.

Other points:
1- Oral health status (caries, gingivitis, periodontitis, number of teeth, etc.) and baseline salivary rates not reported in Table 1.
2-

Reviewer #5

(Remarks to the Author)
We appreciate your important work investigating a probiotic intervention for a debilitating side effect of radiotherapy. Please find below our major and specific concerns aimed at strengthening your manuscript.

Major concerns:
1.The CONSORT diagram indicates that the Protocol and SAP were submitted as supplementary files. However, only the Protocol was found, and its statistical analysis section is quite vague. Please provide the SAP. The authors should provide a detailed description of all analytical methods used in the manuscript or supplementary materials, including but not limited to: how continuous and categorical data were described, what regression model was used for the primary outcome, and how relative risks (RR) were calculated.

2.The supplementary materials mention the use of the Cox proportional hazards model to calculate HR and 95% CI without verifying the proportional hazards assumption. Please supplement the methods section with a description of how this assumption was evaluated.

3.Duration endpoints are not handled appropriately. In the main analysis, patients who never developed SOM were assigned duration = 0 days, which can be seen in Supplementary statistical methods. This approach creates a heavily zero-inflated distribution, particularly in the intervention group where the incidence fell below 50%, rendering the reported median of 0.0 days a mathematical artifact rather than a true reflection of clinical recovery speed. Furthermore, treating "non-events" as "instantaneous events" within a Kaplan-Meier or Cox proportional hazards framework is statistically inappropriate as it violates the underlying assumptions of survival analysis. The Hurdle two-part model described in the Supplementary statistical methods (used for Supplementary Table 4) is suggested to be the main analysis.

4.Line 355: The manuscript states that analyses were performed only on complete cases. It is recommended that the authors clearly state the hypothesized the missing data mechanism, evaluate it and consider imputation for sensitivity analysis where appropriate. Moreover, this description appears inconsistent with the Protocol, which mentions an imputation strategy for missing OM data. Please explain this discrepancy and report the missing data in the baseline table.

5.Line 112 and Supplementary Table 4: In the adjusted analysis for the primary outcome, why does the title of S Table 4 state the ITT population, while the actual numbers for the treatment and control are 71 and 70 (which appear to be the PP set)? Similarly, Supplementary Table 5 shows the results for PP set but the first two column has identical results to Supplementary Table 4 that analyzed the ITT set. Please clarify.

6.Protocol Section 7.2: The authors state that OM was assessed twice per week during radiotherapy (at least 48 hours apart) and once per week for 8 weeks after radiotherapy. This raises concerns about true effect measurement:
1.Potential underestimation of cumulative incidence if SOM occurs after the 8-week post-radiotherapy follow-up window and is treated as non-occurrence rather than right-censoring
2.Bias in estimated onset and duration if OM episodes that both start and resolve between two scheduled visits are not captured, which may systematically shorten observed durations and exaggerate apparent treatment efficacy
Please clarify how interval censoring and the finite observation window were handled analytically.

7.Figure 4a and 4b present biodiversity and richness measured repeatedly across multiple time points. However, the statistical analysis described in Line 337 appears to rely on t-tests, which does not account for within-subject correlations. Please clarify whether methods appropriate for repeated measures (e.g., repeated measures ANOVA or mixed-effects models) were considered, and justify the choice of the current analytical approach.

8.Line 348: The description of the sample size calculation is unclear. It is not evident whether the assumed event rate in the intervention group was based on an absolute reduction of 40% (65%–25%) or a relative reduction of 25% from 65% (i.e., 48.75%). The authors are requested to clarify the exact assumptions used for the intervention group and to provide the

detailed sample size calculation, including the formula and/or statistical code, in their response.

9.The protocol provided with the manuscript (version 1.0, dated 10 November 2025) appears to have been finalized after the completion of the study (March 2024–January 2025). To ensure transparency and compliance with good clinical practice, the authors should submit the original protocol in place prior to study initiation, the final protocol version, and a detailed amendment history, including dates and justifications.

Other comments:
1.Supplementary materials: Why were other clinical parameters analyzed in the per-protocol (PP) population instead of the intention-to-treat (ITT) population (the primary analysis set)? Further clarification is needed.

2.Since the authors mention in the Introduction that OM is a ubiquitous complication (with SOM occurring in 50%–70% of cases) and that MASCC/ISOO has proposed several interventions, why was the control group a placebo rather than an existing active treatment? Are there any recommended treatment measures in the current guidelines? It is suggested that the authors clarify in the Introduction the limitations of previous treatment options (if any).

3.Line 306: The authors mention that patients self-reported daily medication intake. Has authors considered how to verify the issue of adherence, such as regularly counting the pills in the medication boxes? Because overestimating adherence could lead to bias in the PP set.

4.In Figure 1, the N for PP set of the placebo group should be 70 instead of 71 based on the flow chart.

5.Line 243: The authors state, "L. reuteri lozenges demonstrated significantly better patient acceptability, greater administration convenience, and more favorable cost-effectiveness profiles." As a placebo-controlled Phase II trial, direct comparison with other active treatments is not supported. The authors are advised to avoid extrapolating these findings beyond the study context.

6.Figure 2 shows "No RT plan" and "Break time," but these labels are not interpretable without a clear legend embedded in the figure/caption. Also clarify explicitly why the swimmer plot uses n=45/80 vs 63/80 (appears to be restricted to participants who developed OM, but should be stated).

7.Supplementary Table 6 presents the same endpoints under two different N values (N=108 and N=95) without a clear definition of what differs between these sets. Please define inclusion criteria for each set.

8."Supplementary Table S1" should be "Supplementary Table 1" (Lines 314–315), and "Supplementary 1" appears to be an incomplete/incorrect reference (Line 329).

9.The process graph in the Protocol (page 10) incorrectly includes radiotherapy bars in the post-radiotherapy period and should be corrected.

Reviewer #6

(Remarks to the Author)
I co-reviewed this manuscript with one of the reviewers who provided the listed reports. This is part of the Nature Communications initiative to facilitate training in peer review and to provide appropriate recognition for Early Career Researchers who co-review manuscripts.

Version 1:

Reviewer comments:

Reviewer #1

(Remarks to the Author)

Reviewer #2

(Remarks to the Author)
All my comments are addressed properly, I have no further comments

Reviewer #3

(Remarks to the Author)
Dear Peng and colleagues - thank you for our thorough and considered rebuttal. It was great to see almost all of the requested edits implemented, and where necessary, clear justification for any concerns raised in R1. Thank you for your efforts.
I have only a few minor points for suggestion:

1. When describing your study relative to the Ssk12 study (2023), I would make it explicitly clear that these were independent studies. When setting up the rationale as aiming to compare the efficacy of different strains, it may be misinterpreted as a head to head comparison. Please make it clear that all cohorts, including the placebo, were independent in the two studies to avoid confusion.
2. The baseline microbiome differences between the placebo and intervention are nicely described now, thank you. Can you please clarify that you tested how you tested the association between baseline microbiome composition and future OM. What time point did you define mucositis (or was it just highest grade?). Ini this statement, is sounds like you looked at baseline microbiome and stratified by baseline OM severity. "Expanded analysis of oral microbiota at baseline: We performed additional analyses to explore whether baseline oral microbiota composition differed according to OM status. The results showed that no significant differences in α diversity or β diversity between patients with OM and patients without OM at baseline (Supplementary Fig. 11a-c).
3. It was fascinating to learn of the nuances of the Chinese culture with respect to stoicism, pain, adherence etc. I note that none of this went into the discussion of the manuscript. While I appreciate it doesnt require lengthy discussion, it may be of use to some readers who are familiar with these values.

Thank you again for your work, I look forward to seeing what you and your team does next.

Reviewer #4

(Remarks to the Author)
The reviewers have made a commendable effort to address the concerns raised during the first round of review; however, the revisions are still not satisfactory:

Bioinformatic analysis
1- While the authors performed species-level taxonomy assignment of the ASVs using blastn as recommended, the percent identity threshold applied (80%) is very low. For species-level classification, at least 98% is commonly used. Often higher coverage than 90% is also required for more reliable assignments. With the parameters employed, the species level calls are not reliable. It is also not clear how blastn was run and how ties were handled.
2- Since CLR-transformation is now applied to the data, there is no need to rarefy the ASV tables since this results in loss of data.
3- The authors have switched to compositional data aware methods as recommended (CLR transformation, Aitchison distances, and MaAslin2, etc.) but they need to specify the threshold an method used for FDR and ensure that PERMANOVA results are also adjusted for pairwise comparisons where applicable.

Downstream microbiome data analysis
This remains a major concern. A more thoughtful and focused analysis is needed to demonstrate how baseline microbiome composition may have influenced the study outcomes and how it was modulated by the intervention. Here are specific comments:
1- Supplementary Figure 9g shows a significant difference in the microbiome between the intervention and placebo groups at baseline. Differential abundance analysis should be performed, and the identified DA species (not just the Shannon index) should be included in the efficacy analysis to assess whether they confounded the effect of the intervention.
2- A more important question is whether baseline (T0) microbiome predicts development of OM/SOM overall (i.e., independent of the intervention) and within each study arm. The first part is addressed in Supplementary Figure 11a–c, but although the beta diversity analysis is only marginally significant (0.07), DA analysis should be carried out to identify taxa (species or genera) that could potentially predict OM/SOM. The authors attempted to perform stratified analysis by intervention in Supplementary Figure 11d–g, but used data from all time points (likely misunderstanding what was meant by “overall”), which is not appropriate. As in a–c, the stratified analysis should be limited to T0 samples and accompanied by DA analysis. As these analyses are based on baseline data, they are predictive in nature and should be framed as such
3- Accordingly, the data presented in Supplementary Figure 12 are not meaningful, since all time points were included. Instead, the authors should assess the microbiome associated with development of OM/SOM at T1 and, separately, T2, both overall (i.e., independent of the intervention) and stratified by intervention. This may provide important insight into why the intervention was effective only in a subset of patients (e.g., probiotic colonization occurring only in a subset). These analyses are associative rather than predictive and should be clearly interpreted in that context

Figure 4: I think this should be used to carefully present microbiome data most relevant to the study objective and outcomes, i.e. 1) Effect of the intervention on the microbiome (e.g. Longitudinal changes T0 T1 T02 including DA analysis results); and 2) Microbiome differences/changes in relation to OM/SOM (both baseline and follow-up). Perhaps, supplementary figures 11 and 12 after the revisions mentioned above. Multivariate analysis (e.g. MaAslin2) of T0, and separately T2, data including both clinical outcome and intervention as covariates would be very informative.

Reviewer #5

(Remarks to the Author)
We thank the authors for their detailed point-by-point responses and revisions. Several concerns from the first round have been adequately addressed. However, a number of methodological issues remain unresolved or insufficiently responded to, as outlined below.

The following comments concern issues that were raised in the first round but have not been fully resolved in the revised

manuscript:

1.In response to our previous comment, the authors confirmed that the proportional hazards assumption was violated for both SOM and OM onset (Schoenfeld residuals test, $P < 0.05$), and conducted a piecewise Cox regression as a sensitivity analysis. However, the results of this piecewise Cox analysis were presented only in a "Table for reviewers" and have not been incorporated into the manuscript or supplementary materials. Given that the proportional hazards assumption has been formally shown to be violated, the overall hazard ratios reported in Table 2 for onset endpoints may not be statistically valid as standalone effect estimates. The authors are requested to include the piecewise Cox regression results in the supplementary materials and to add appropriate discussion of this limitation in the manuscript.

2.In response to our previous comment, the authors adopted the two-part (Hurdle) model as the primary analytical approach for duration endpoints. However, the revised Table 2 continues to report the median SOM duration as 0.0 days for the L. reuteri group alongside the mean ratio from the two-part model. Given that this median value was previously acknowledged to be a mathematical artifact of the zero-inflated distribution rather than a clinically meaningful estimate, its continued presence in the table risks misleading readers. The authors are requested to either remove the median duration from Table 2 and throughout the paper, or add a clear explanatory footnote.

The following comments concern new issues identified in the revised manuscript and newly submitted materials:

3.The title of Supplementary Table 4 refers to results "with efficacy in Model 3." However, Model 3 is not defined anywhere in the manuscript, the supplementary methods, or the SAP. Only Model 1 and Model 2 are formally described. The authors are requested to either formally define Model 3 or correct the table title, and to confirm that the term "Model 3" has been removed from all parts of the manuscript.

4.In the main text (Lines 131–135), the authors describe the adjusted effect estimates from Model 1 and Model 2 as "adjusted relative risk," whereas the footnote of Table 2 explicitly states that "Cox regression was used for incidence/onset in adjusted models." Cox proportional hazards regression yields a hazard ratio (HR), not a relative risk (RR). These two effect measures are conceptually and numerically distinct and are not interchangeable, and mislabeling HR as RR represents a fundamental statistical terminology error that directly contradicts the table footnote.

Furthermore, in Table 2, the effect size for SOM/OM incidence switches from RR in the unadjusted analysis to HR in the adjusted models (Model 1 and Model 2), yet both are presented in the same "Effect size" column without per-row labeling. This is potentially misleading, as readers may interpret the change in magnitude (e.g., RR 0.61 to HR 0.44) as solely reflecting the effect of covariate adjustment, when it also reflects a change in the effect measure itself.

The authors should use the modified Poisson regression as used in subgroup analysis if the modified RR is the desired output, or clearly state in the manuscript that HR and RR are not comparable.

5.The manuscript states (Line 440) that "Bonferroni correction for multiple comparisons was applied for secondary outcome analyses." However, the corrected significance threshold is not reported anywhere in the paper. The authors are requested to explicitly report this threshold in the manuscript so that readers can independently evaluate the statistical significance of each secondary endpoint.

Reviewer #6

(Remarks to the Author)
I co-reviewed this manuscript with one of the reviewers who provided the listed reports. This is part of the Nature Communications initiative to facilitate training in peer review and to provide appropriate recognition for Early Career Researchers who co-review manuscripts.

Version 2:

Reviewer comments:

Reviewer #4

(Remarks to the Author)
Thank you to the authors for the substantial revision. A few more comments:
1- For unnamed taxa ending with sp. HMT XXX, the genus name should be included throughout (text and figures), e.g. Pseudoleptotrichia sp. HMT-219.

2- Figure 4i: the description above the panel should be corrected (T0 instead of T2). Logically, panel j should be used to present differences between OM and without OM at T2. Additional two panels should be used to show differences between Placebo and L. reuteri in OM and separately without OM groups. This will account of all major findings that need to presented in the main manuscript.

3- Be very careful with interpertation of the results. For example several pathobionts increased overtime in both groups not just the placebo groups, e.g. F. nucleatum and Prevotella nigrescens (compare panels g and h, Figure 4. Also, note that the taxa with -ve coefficient means association with the reference (control) group. In this case, taxa such as Scardovia wiggsiae

and C. concisus at T0 are actually associated with decreased risk of OM not the other way around. Please check the directionality of associations and revise accordingly--this is the most important part of the microbial findings.

Reviewer #5

(Remarks to the Author)
We thank the authors for their detailed point-by-point responses and revisions. Several concerns from the previous round have been adequately addressed. However, a number of methodological issues remain unresolved or insufficiently responded to, as outlined below.

1.Following Reviewer 5's Comment 6, the authors now report a Bonferroni-corrected significance threshold of $P < 0.01$ (0.05/5) for secondary outcome analyses. However, restricting Bonferroni correction to the five secondary endpoints while testing the primary endpoint at an uncorrected alpha = 0.05 is only valid under a pre-specified hierarchical (gatekeeping) testing procedure, in which formal testing of secondary endpoints is conditional on the primary endpoint reaching significance. Neither the Methods section nor the SAP describes such a hierarchical structure. In the absence of a pre-specified gatekeeping procedure, all formally tested endpoints belong to a single multiplicity family, and Bonferroni correction should be applied across all six endpoints (corrected threshold ≈ 0.0083). The authors should either explicitly describe and justify a pre-specified hierarchical testing procedure in the Methods, or apply Bonferroni correction across all six endpoints.

The Table 2 footnote states that unadjusted incidence comparisons used "the χ^2 test (RR, 95% CI)." This is imprecise: the χ^2 test yields only a P value and does not itself produce a risk ratio or confidence interval. The RR and its 95% CI must be computed separately. The footnote should clarify the method used to obtain the unadjusted RR and CI as described in the

Version 3:

Reviewer comments:

Reviewer #4

(Remarks to the Author)
No further comments

Reviewer #5

(Remarks to the Author)
I have no more comments

Open Access This Peer Review File is licensed under a Creative Commons Attribution 4.0 International License, which permits use, sharing, adaptation, distribution and reproduction in any medium or format, as long as you give appropriate credit to the original author(s) and the source, provide a link to the Creative Commons license, and indicate if changes were made.

In cases where reviewers are anonymous, credit should be given to 'Anonymous Referee' and the source.
The images or other third party material in this Peer Review File are included in the article's Creative Commons license, unless indicated otherwise in a credit line to the material. If material is not included in the article's Creative Commons license and your intended use is not permitted by statutory regulation or exceeds the permitted use, you will need to obtain permission directly from the copyright holder.
To view a copy of this license, visit https://creativecommons.org/licenses/by/4.0/

We sincerely appreciate the reviewers for their constructive and insightful comments. In response to their feedback, we have undertaken substantial revisions to the manuscript. In brief, we have clarified methodological details, updated statistical analyses where appropriate, supplemented analyses with oral microbiota data, incorporated data on adherence, strengthened the interpretation of clinical relevance, and highlighted the study's novelty and translational value. We have also refined the language and structure to enhance readability.

We believe that all reviewer comments have been addressed in the revised manuscript and in this response. We hope that the revisions now meet the standards for publication in ***Nature Communications***. All changes have been tracked using the "Track Changes" function in the manuscript. Below, we provide point-by-point responses to each comment. Please also find attached a detailed, point-by-point response to all reviewer comments and a marked version of the revised manuscript.

Point-by-point response to the reviewer comments on the manuscript "***Lactobacillus reuteri* for Radiation-Induced Oral Mucositis in Patients with Head and Neck Cancer: A Randomized Clinical Trial**".

Reviewers' Comments:

Reviewer #1

Comment 1: *"This manuscript reports a double-blind, placebo-controlled randomized phase II trial evaluating Lactobacillus reuteri lozenges for the prevention and mitigation of radiation-induced oral mucositis (RIOM) in patients with head and neck cancer. The authors demonstrate a statistically significant reduction in the incidence, delayed onset, and shortened duration of severe oral mucositis (SOM), accompanied by favorable microbiome changes and an acceptable safety profile."*

Response to the Comment 1:

We sincerely thank you for the thoughtful summary and constructive assessment of our manuscript. Your insightful comments and valuable suggestions have greatly improved the quality of our work. In the following sections, we have tried our best to carefully address each specific comment to further improve the clarity and completeness of our work, with particular attention to the novelty, translational potential, and completeness of the reported results.

Comment 2: *Conceptual and narrative redundancy with prior work*

The authors have previously published a randomized controlled trial of Streptococcus salivarius K12 for RIOM (J Clin Oncol, 2024), which is cited. While the present study evaluates a different probiotic strain, the conceptual framing, endpoint hierarchy, manuscript structure, and discussion logic are strikingly similar, with the probiotic species effectively substituted within an otherwise unchanged narrative. This degree of structural and rhetorical overlap diminishes perceived novelty and is problematic for a high-impact, general-interest journal.

Response to Comment 2:

Thank you for your insightful comments. The main reason we conducted this study was the difference in mechanisms between the two probiotic strains. Specifically, in addition to the competitive exclusion and direct inhibition of pathogenic microorganisms exhibited by *SsK12*, *L. reuteri* also actively modulates the host inflammatory microenvironment and maintains mucosal homeostasis.[1-4] On the other hand, we sought not only to explore the effectiveness of *L. reuteri* in relieving RIOM, but also to explore whether the protective mechanism of probiotics against RIOM is strain specific or a common property. These considerations were the main rationale for undertaking this study. We have carefully discussed the differences between our study and the *SsK12* study, and emphasized the rationale for conducting this study in the Discussion section.

Accordingly, the specific revisions made in the manuscript are as follows:

Page 12, Line 257 in the revised manuscript:

"Prior clinical trials have indicated that specific probiotic strains, including *Lactobacillus brevis CD2*, *Streptococcus salivarius K12* (*SsK12*), and probiotic combinations containing *Bifidobacterium longum*, *Lactobacillus lactis*, and *Enterococcus faecium* have demonstrated significant efficacy in reducing RIOM.[3,26,27] Collectively, these convergent beneficial effects across distinct strains raise important questions regarding the mechanistic basis of probiotic protection against RIOM. As proposed by Wardill et al., such conserved efficacy suggests that clinical benefit may not arise exclusively from strain-specific metabolic properties, but rather from the general ability of probiotic microorganisms to occupy ecological niches and competitively restrict pathogen colonization.[28] Notably, *L. reuteri* may extend beyond these shared mechanisms by additionally modulating the host inflammatory microenvironment and preserving mucosal homeostasis, thereby reinforcing its protective role against RIOM.[29-31]"

Comment 3: *Limited translational positioning.*

Despite repeated references (in the Abstract, Introduction, Results, and Discussion) to the authors' previous basic and translational research, no specific foundational or mechanistic references are provided. Furthermore, the translational relevance remains limited for a journal such as Nature Communications, with no functional validation beyond 16S rDNA profiling.

Response to Comment 3:

We sincerely thank you for your constructive comments. In response, we have added foundational references regarding *L. reuteri* in oral health and mucosal protection to the Introduction. The proposed mechanisms mainly include forming stable, protective biofilms on oral epithelia, the production of the antimicrobial compound reuterin, and its immunomodulatory effects. In addition, regarding the concern about the lack of functional validation beyond 16S rDNA profiling, we have supplemented our analysis with PICRUSt2-based functional prediction. However, we acknowledge that our study may still lack direct functional validation. Accordingly, we have noted in the Discussion that, as no mechanistic, functional, or causal link between specific microbial changes and clinical outcomes has been demonstrated, the results of the oral microbiota should be interpreted cautiously.

Accordingly, the specific revisions made in the manuscript are as follows:

Page 5, Line 86 in the revised manuscript:

"Among various probiotic strains, *Limosilactobacillus reuteri* (*L. reuteri*) exhibits notable potential for managing OM through multiple, complementary mechanisms.[10,11] A key attribute is its ability to form stable, protective biofilms on oral epithelia, which promotes mucosal colonization and competitively excludes pathogenic bacteria.[12,13] Furthermore, *L. reuteri* targets oxidative stress via its metabolite reuterin to reduce direct tissue damage and enhances epithelial barrier integrity, synergistically countering key drivers of mucosal dysfunction in OM.[14-17] Together, these actions help restore microbial balance and dampen inflammatory cascades, underpinning its documented

efficacy in oral inflammatory diseases such as gingivitis and periodontitis.[18-22]"

Page 10, Line 208 in the revised manuscript:

"Functionally, PICRUSt2 prediction showed that the expression of NADH dehydrogenase (EC 1.6.5.3) was markedly upregulated in the *L. reuteri* group at the end of radiotherapy compared with the placebo group (Fig. 4h). Given that NADH dehydrogenase is a key component of the bacterial respiratory chain and is often linked to oxidative stress responses and energy metabolism, its high expression in the *L.reuteri* group at the end of radiotherapy suggests that *L. reuteri* may alleviate RIOM by enhancing the metabolic fitness and antioxidant capacity of the oral microbiota.[24,25]"

Page 15, Line 314 in the revised manuscript:

"Moreover, as no mechanistic, functional, or causal link between specific microbial changes and clinical outcomes has been demonstrated, the results of the oral microbiota should be interpreted cautiously."

Comment 4: *Limited translational positioning*

While Figure 4 provides valuable associative evidence that Lactobacillus reuteri administration modulates the oral microbiome during radiotherapy, the data remain descriptive and taxonomic. The manuscript would benefit from a more cautious interpretation, as no functional or causal link between specific microbial changes and clinical outcomes is demonstrated.

Response to Comment 4:

Thank you very much for your insightful suggestion. We have thoroughly revised the microbiome section of the manuscript to ensure a more cautious interpretation. Also, we have explicitly stated in the revised Discussion section that the observed microbial changes represent associations rather than causal links and that functional validation is lacking, underscoring the need for caution when interpreting these results.

Accordingly, the specific revisions made in the manuscript are as follows:

Page 15, Line 314 in the revised manuscript:

"Moreover, as no mechanistic, functional, or causal link between specific microbial changes and clinical outcomes has been demonstrated, the results of the oral microbiota should be interpreted cautiously."

Comment 5: *Trial reporting and transparency*

The clinical trial registration number should be included in the Abstract, in accordance with CONSORT and journal standards.

Response to Comment 5:

We sincerely thank you for this suggestion. We have now included the clinical trial registration number (NCT06285591) in the Abstract section.

Accordingly, the specific revisions made in the manuscript are as follows:

Page 2, Line 39 in the revised manuscript:

"To assess whether modulation of the oral microbiota through probiotic supplementation could alleviate severe OM (WHO Oral Toxicity criteria grade ≥ 3,

SOM) and enhance treatment tolerance, we conducted a double-blind, placebo-controlled, randomized phase II trial from March 2024 to January 2025 at West China Hospital of Sichuan University (NCT06285591)."

Comment 6: *Trial reporting and transparency*

Nineteen patients did not complete or receive the experimental intervention. The reasons for non-receipt or discontinuation require clearer explanation, including timing and potential impact on outcome interpretation.

Response to Comment 6:

We sincerely thank you for raising this important point. We have now provided a detailed summary in the revised manuscript, specifying the exact time points, primary reason, and potential impact on outcome interpretation for the 19 patients who did not complete the experimental intervention.

Accordingly, the specific revisions made in the manuscript are as follows:

Page 6, Line 110 in the revised manuscript:

"Two patients withdrew due to disease progression, and three patients withdrew consent due to their request to add other medications for OM prevention during the early stage of radiotherapy, whereas the remaining patients withdrew due to antibiotic use toward the end of radiotherapy."

Comment 7: *Radiotherapy-related considerations*

The authors should briefly discuss the influence of tumor localization (nasopharynx, oral cavity, oropharynx, hypopharynx, larynx) on dose delivered to the oral cavity, a major determinant of mucositis risk.

Response to Comment 7:

Thank you very much for your valuable suggestion. In response, we have conducted subgroup analyses stratified by tumor site and oral cavity radiation dose. The results showed that *L. reuteri* remained a significant protective factor in patients with nasopharyngeal carcinoma and in the subgroup with an oral cavity radiation dose < 40 Gy. Additionally, we have added a concise discussion on the influence of tumor location on oral radiation dose and mucositis risk in the Discussion section of the revised manuscript.

Accordingly, the specific revisions made in the manuscript are as follows:

Page 15, Line 305 in the revised manuscript:

"First, the single-center design and lack of stratified randomization may limit generalizability and introduce potential prognostic imbalances across the heterogeneous tumor sites and variable radiation doses involved."

Page 8 in the revised Supplementary information:

Supplementary Fig. 3. Subgroup analysis of severe oral mucositis incidence.

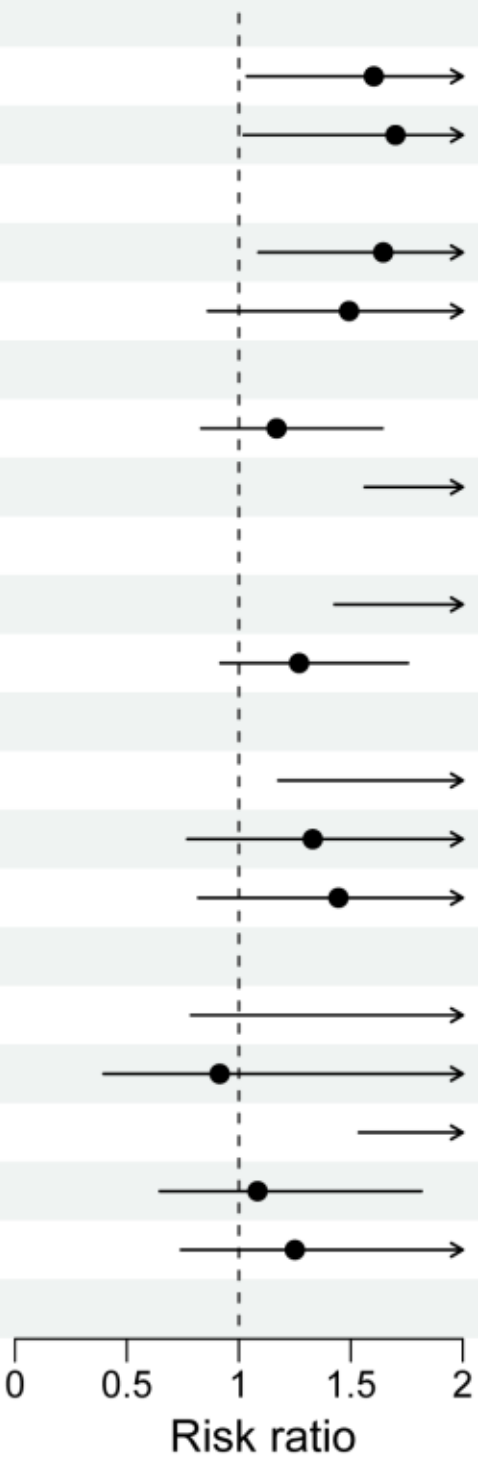

Subgroup	L. reuteri case/total	Placebo case/total	RR (95% CI)	*P*
Tobacco use (*p for interaction = 0.900*)				
No	17/42	24/37	1.60 (1.04−2.48)	0.034
Yes	13/38	25/43	1.70 (1.02−2.83)	0.041
Alcohol use (*p for interaction = 0.837*)				
No	16/37	32/45	1.64 (1.09−2.49)	0.018
Yes	14/43	17/35	1.49 (0.86−2.58)	0.153
Concurrent chemotherapy (*p for interaction = 0.038*)				
No	24/43	30/46	1.17 (0.83−1.64)	0.369
Yes	6/37	19/34	3.45 (1.56−7.60)	0.002
Treatment type (*p for interaction = 0.079*)				
Definitive	6/38	20/40	3.17 (1.43−7.03)	0.005
Postoperative treatment	24/42	29/40	1.27 (0.92−1.75)	0.150
Oral cavity dose (*p for interaction = 0.657*)				
<40 Gy	10/37	23/40	2.13 (1.18−3.85)	0.013
40−50 Gy	12/27	13/22	1.33 (0.77−2.30)	0.307
>50 Gy	8/16	13/18	1.44 (0.82−2.55)	0.204
Tumor site (*p for interaction = 0.182*)				
Hypopharynx	2/10	6/10	3.00 (0.79−11.44)	0.108
Larynx	5/8	4/7	0.91 (0.40−2.11)	0.834
Nasopharynx	3/24	14/24	4.67 (1.54−14.18)	0.007
Oral cavity	12/22	13/22	1.08 (0.65−1.82)	0.761
Oropharynx	8/13	10/13	1.25 (0.74−2.11)	0.403
Others	0/3	2/4	Not Evaluable	Not Evaluable

Abbreviations: Risk ratios (RRs) and 95% CIs were estimated using modified Poisson regression with robust standard errors.

Comment 8: *Radiotherapy-related considerations*

Radiotherapy doses are reported in cGy, whereas standard clinical convention uses Gy. Units should be standardized throughout.

Response to Comment 8:

We sincerely thank you for this correction. We have now standardized the reporting of radiotherapy doses by converting all values from cGy to Gy throughout the manuscript and tables to align with the standard clinical convention.

Comment 9: *Supportive care and plausibility of reported data*

The reported 54% rate of parenteral nutrition appears unexpectedly high and requires

clarification.

Response to Comment 9:

We sincerely thank you for raising this important point. The relatively high rate of parenteral nutrition in our study is the institutional practice at our center to ensure uninterrupted delivery of radiotherapy. In patients with head and neck cancer undergoing radiotherapy, treatment-related toxicities, including RIOM, xerostomia, and dysgeusia, frequently lead to eating difficulties such as anorexia and dysphagia. In addition, common gastrointestinal reactions raised by radiotherapy such as nausea, vomiting, and anorexia may compromise patients' tolerance to enteral nutrition. In our center, when patients are unable to tolerate enteral nutrition, parenteral nutrition is routinely recommended to maintain nutritional status and ensure uninterrupted radiotherapy, in line with established guidelines.[5,6] This practice may contribute to the 54% parenteral nutrition utilization rate in our study.

Comment 10: *Supportive care and plausibility of reported data*

The manuscript should explicitly confirm that no other oral agents, probiotics, mouthwashes, antimicrobial products, or experimental supportive treatments were administered during radiotherapy.

Response to Comment 10:

We sincerely thank you for highlighting this important point. We have now explicitly stated in the Methods section that no concurrent oral probiotics, therapeutic

mouthwashes, antimicrobial agents, or other experimental supportive oral treatments were permitted during the radiotherapy period.

Accordingly, the specific revisions made in the manuscript are as follows:

Page 18, Line 379 in the revised manuscript:

"Standard supportive measures including oral rinsing (pure water), analgesic use, and nutritional supplementation remained allowable throughout radiotherapy, while other oral agents, probiotics, antimicrobial products (such as antibiotics or antifungal agents), topical mouthwashes other than pure water (such as antibacterial mouthwashes or baking soda solutions), and any other experimental supportive interventions were prohibited."

Comment 11: *Oncological outcomes and safety reporting*

Although this is a supportive care trial, oncological outcomes (e.g., locoregional control, early progression, treatment interruptions) should be briefly described in both arms.

Response to Comment 11:

We sincerely thank you for this valuable suggestion. We have added a summary in the Results section reporting oncological outcomes for both the *L. reuteri* and placebo groups, as detailed in Supplementary Table 10.

Accordingly, the specific revisions made in the manuscript are as follows:

Page 11, Line 227 in the revised manuscript:

"Among the enrolled patients, 51.2% (82/160) underwent surgery before radiotherapy, whereas 48.8% (78/160) received definitive radiotherapy and were therefore assessed for tumor response at 3 months after radiotherapy. Among patients who underwent surgery, distant metastasis occurred in one patient in the *L. reuteri* group and two patients in the placebo group, while one patient in each group experienced local recurrence during follow-up. According to RECIST v1.1 criteria, the complete response rates and partial response rates were 89.5 % (34/38) and 7.9% (3/38) in the *L. reuteri* group versus 90.0% (36/40) and 5.0% (2/40) in the placebo group among patients treated with definitive radiotherapy (Supplementary Table 10)."

Page 27 in the revised Supplementary information:

Supplementary Table 10. Tumor response in patients receiving definitive radiotherapy between *Limosilactobacillus reuteri* group and placebo group.

Response	*L. reuteri* (n=38)	Placebo (n=40)
Complete response, No. (%)	34 (89.5)	36 (90.0)
Partial response, No. (%)	3 (7.9)	2 (5.0)
Progressive disease, No. (%)	1 (2.6)	2 (5.0)

NOTE. Tumor response assessments were conducted at baseline and 3 months post-radiotherapy in patients with head and neck cancer receiving definitive radiotherapy. Radiographic evaluation included contrast-enhanced CT or MRI of the head and neck, contrast enhanced CT of the thorax, and abdominal ultrasound. Changes in tumor size were evaluated according to RECIST v1.1 criteria.

Comment 12: *Oncological outcomes and safety reporting*

Toxicities should be reported with explicit CTCAE grading rather than aggregated events.

Response to Comment 12:

We sincerely thank you for this insightful suggestion. We have now revised the relevant sections and Supplementary information to report toxicities with explicit CTCAE grading instead of aggregated event counts.

Accordingly, the specific revisions made in the Supplementary information are as follows:

Page 25 in the revised Supplementary information:

Supplementary Table 8. Treatment-related adverse events in the *Limosilactobacillus reuteri* and placebo groups.

Adverse events	*L. reuteri* (n=80)			Placebo (n=80)		
grade, **No. (%)**	Grade 1	Grade 2	Grade ≥3	Grade 1	Grade 2	Grade ≥3
Abdominal distension	1 (1.3)	0 (0)	0 (0)	0 (0)	0 (0)	0 (0)
Diarrhea	1 (1.3)	0 (0)	0 (0)	0 (0)	0 (0)	0 (0)
Dyspepsia	1 (1.3)	0 (0)	0 (0)	1 (1.3)	0 (0)	0 (0)

Supplementary Table 9. Summary of any-grade adverse events in all patients.

Adverse events	*L. reuteri* (n=80)			Placebo (n=80)		
grade, No. (%)	Grade 1	Grade 2	Grade ≥3	Grade 1	Grade 2	Grade ≥3
Abdominal distension	1 (1.3)	0 (0)	0 (0)	0 (0)	0 (0)	0 (0)
Alopecia	20 (25.0)	0 (0)	0 (0)	18 (22.5)	0 (0)	0 (0)
Anemia	13 (16.3)	0 (0)	0 (0)	17 (21.3)	2 (2.5)	1 (1.3)
Anxiety	7 (8.8)	1 (1.3)	0 (0)	7 (8.8)	0 (0)	0 (0)
Cough	22 (27.5)	6 (7.5)	0 (0)	23 (28.8)	7 (8.8)	0 (0)
Constipation	4 (5.0)	0 (0)	0 (0)	7 (8.8)	1 (1.3)	0 (0)
Decreased appetite	34 (42.5)	8 (10.0)	0 (0)	22 (27.5)	27 (33.8)	0 (0)
Depression	4 (5.0)	0 (0)	0 (0)	3 (3.8)	0 (0)	0 (0)
Diarrhea	1 (1.3)	0 (0)	0 (0)	0 (0)	0 (0)	0 (0)
Dizziness	8 (10.0)	0 (0)	0 (0)	11 (13.8)	0 (0)	0 (0)
Dyspepsia	1 (1.3)	0 (0)	0 (0)	1 (1.3)	0 (0)	0 (0)
Dysphagia	33 (41.3)	15 (18.8)	10 (12.5)	20 (25.0)	29 (36.3)	13 (16.3)
Dyspnea	1 (1.3)	0 (0)	0 (0)	2 (2.5)	0 (0)	0 (0)
Face and neck edema	20 (25.0)	2 (2.5)	0 (0)	21 (26.3)	2 (2.5)	0 (0)
Fatigue	36 (45.0)	4 (5.0)	1 (1.3)	34 (42.5)	7 (8.8)	2 (2.5)
Headache	5 (6.3)	0 (0)	0 (0)	8 (10.0)	1 (1.3)	0 (0)

Hoarseness	7 (8.8)	0 (0)	0 (0)	6 (7.5)	0 (0)	0 (0)
Insomnia	24 (30.0)	3 (3.8)	0 (0)	22 (27.5)	1 (1.3)	0 (0)
Leukopenia	8 (10.0)	3 (3.8)	3 (3.8)	8 (10.0)	4 (5.0)	6 (7.5)
Lymphopenia	25 (31.3)	16 (20.0)	17 (21.3)	28 (35.0)	14 (17.5)	19 (23.8)
Nausea	26 (32.5)	8 (10.0)	3 (3.8)	16 (20.0)	25 (31.3)	2 (2.5)
Neck pain	4 (5.0)	0 (0)	0 (0)	6 (7.5)	0 (0)	0 (0)
Neutropenia	4 (5.0)	0 (0)	0 (0)	5 (6.3)	1 (1.3)	0 (0)
Oropharyngeal pain	35 (43.8)	19 (23.8)	3 (3.8)	31 (38.8)	32 (40.0)	6 (7.5)
Pyrexia	1 (1.3)	0 (0)	0 (0)	1 (1.3)	0 (0)	0 (0)
Radiation skin injury	19 (23.8)	39 (48.8)	4 (5.0)	24 (30.0)	36 (45.0)	5 (6.3)
Stoma site pain	13 (16.3)	2 (2.5)	0 (0)	14 (17.5)	2 (2.5)	0 (0)
Thrombocytopenia	3 (3.8)	0 (0)	0 (0)	4 (5.0)	0 (0)	0 (0)
Tinnitus	2 (2.5)	0 (0)	0 (0)	3 (3.8)	0 (0)	0 (0)
Vomiting	10 (12.5)	1 (1.3)	0 (0)	7 (8.8)	2 (2.5)	0 (0)

Comment 13: *Compliance with the experimental intervention*

Treatment compliance (e.g., proportion of prescribed lozenges consumed, adherence over time) should be reported, as this is essential for interpreting prophylactic efficacy.

Response to Comment 13:

Thank you very much for this important and insightful comment. We have now reported the detailed adherence data and also added a description of the adherence assessment method in the Methods section. The mean adherence rate was 94.3% in the *L. reuteri* group and 92.6% in the placebo group in the ITT population, and 96.4% and 95.0% in the PP population, respectively.

Accordingly, the specific revisions made in the manuscript are as follows:

Page 18, Line 378 in the revised manuscript:

"Treatment adherence was calculated as the ratio of the actual number of lozenges used to the prescribed number of lozenges during radiotherapy."

Page 6, Line 113 in the revised manuscript:

"The mean adherence rate was 94.3% in the *L. reuteri* group and 92.6% in the placebo group in the ITT population, and 96.4% and 95.0% in the PP population, respectively."

Table for reviewers:

Adherence rate in the *L. reuteri* and placebo groups. Adherence rate was calculated as the ratio of the actual number of lozenges used to the prescribed number of lozenges during radiotherapy. Adherence rates were compared using the Wilcoxon rank-sum test.

Adherence rate (%), mean ± SD	*L. reuteri*	Placebo	*P*
Intention-to-Treat Set	94.3 ±10.3)	92.6 ± 11.5	0.063
Per Protocol Set	96.4 ± 3.57)	95.0 ± 4.56	0.051

Comment 14: *Figure 3 presents data only during radiotherapy. The absence of post-radiotherapy mucositis follow-up is a limitation and should be justified or acknowledged, as mucositis often persists beyond treatment completion.*

Response to Comment 14:

We sincerely thank you for highlighting this important point. OM was assessed once a week for 8 weeks after radiotherapy in our study. Figure 3 was designed to illustrate the dynamic trajectory of OM severity in direct response to the accumulating radiation dose during radiotherapy. The complete post-radiotherapy follow-up data were presented in Figure 2, which together with Figure 3 provides a comprehensive overview of the overall course of OM. We have clarified this point in the figure legend.

Accordingly, the specific revisions made in the manuscript are as follows:

Page 28, Line 662 in the revised manuscript:

"Fig. 3. The data presented are limited to the radiotherapy period to illustrate the dynamics of OM in direct relation to *L.reuteri* supplementation."

Comment 15: *Table 1 indicates that approximately 50% of patients were non-smokers and non-drinkers, which differs markedly from typical European head and neck cancer populations. Given the predominance of nasopharyngeal carcinoma, the authors should discuss etiological differences (e.g., EBV, HPV status) and limitations in generalizability to Western populations. If available, HPV or viral status data should be reported or explicitly acknowledged as unavailable.*

Response to Comment 15:

We sincerely thank you for this thoughtful and insightful comment. We have now supplemented Table 1 and Supplementary Table 3 with data on HPV status (for oropharyngeal carcinoma patients) and EBV status (for nasopharyngeal carcinoma patients), as determined by pathological testing. We have also added a brief discussion in the Discussion section of the manuscript, acknowledging that the high proportion of non-smokers and non-drinkers, as well as the etiological role of viral infections (particularly EBV in nasopharyngeal carcinoma) in our cohort, may limit the direct generalizability of our findings to Western populations.

Accordingly, the specific revisions made in the manuscript are as follows:

Page 15, Line 317 in the revised manuscript:

“Finally, the distinct etiological profile of our cohort characterized by a high prevalence of EBV and low rates of tobacco and alcohol use may not fully represent the HPV-dominant or lifestyle-associated head and neck cancer populations typical of Western settings.[40] Future multicenter trials are warranted to validate these findings across more diverse clinical and demographic landscapes.”

Page 31 in the revised manuscript:

TABLE 1 Baseline patient characteristics

Characteristic	No. (%) of Patients		
	L. reuteri (n = 80)	Placebo (n = 80)	All Patients (N = 160)
Sex, No. (%)			
Male	68 (85.0)	61 (76.2)	129 (80.6)
Female	12 (15.0)	19 (23.8)	31 (19.4)
Age, years, mean ± SD	56.3 ± 11.3	57.4 ± 12.4	56.8 ± 11.8
BMI, kg/m^2, mean ± SD	22.9 ± 3.6	22.6 ± 2.4	22.8 ± 3.0
ECOG PS, No. (%)			
0	54 (67.5)	53 (66.2)	107 (66.9)
1	24 (30.0)	25 (31.2)	49 (30.6)
2	2 (2.5)	2 (2.5)	4 (2.5)
Tumor pathologic types, No. (%)			
Squamous carcinoma	67 (83.8)	73 (91.2)	140 (87.5)
Others	13 (16.2)	7 (8.8)	20 (12.5)
Tumor site, No. (%)			
Nasopharynx	24 (30.0)	24 (30.0)	48 (30.0)
Oral cavity	22 (27.5)	22 (27.5)	44 (27.5)
Oropharynx	13 (16.2)	13 (16.2)	26 (16.2)
Hypopharynx	10 (12.5)	10 (12.5)	20 (12.5)
Larynx	8 (10.0)	7 (8.8)	15 (9.4)
Others [a]	3 (3.8)	4 (5.0)	7 (4.4)
Treatment type, No. (%)			
Definitive	38 (47.5)	40 (50.0)	78 (48.8)
Postoperative treatment	42 (52.5)	40 (50.0)	82 (51.2)
Concurrent chemotherapy, No. (%)			
Yes	37 (46.2)	34 (42.5)	71 (44.4)
No	43 (53.8)	46 (57.5)	89 (55.6)
TNM stage [b], No. (%)			
I-II	18 (22.5)	16 (20.0)	34 (21.2)
III	23 (28.7)	22 (27.5)	45 (28.1)
IVa	25 (31.2)	29 (36.2)	54 (33.8)

IVb	14 (17.5)	13 (16.2)	27 (16.9)
Tumor primary site, No. (%)			
T1	6 (7.5)	9 (11.2)	15 (9.4)
T2	26 (32.5)	26 (32.5)	52 (32.5)
T3	23 (28.7)	24 (30.0)	47 (29.4)
T4	25 (31.2)	21 (26.2)	46 (28.7)
Nodal involvement, No. (%)			
N0	28 (35.0)	25 (31.2)	53 (33.1)
N1	14 (17.5)	15 (18.8)	29 (18.1)
N2	26 (32.5)	24 (30.0)	50 (31.2)
N3	12 (15.0)	16 (20.0)	28 (17.5)
Tobacco use, No. (%)			
Yes	38 (47.5)	43 (53.8)	81 (50.6)
No	42 (52.5)	37 (46.2)	79 (49.4)
Alcohol use, No. (%)			
Yes	43 (53.8)	35 (43.8)	78 (48.8)
No	37 (46.2)	45 (56.2)	82 (51.2)
Oropharyngeal cancer HPV status, No. (%)			
Positive	5 (38.5)	3 (23.1)	8 (30.8)
Negative	8 (61.5)	10 (76.9)	18 (69.2)
Nasopharynx cancer EBV status, No. (%)			
Positive	24 (100.0)	24 (100.0)	48 (100.0)
Negative	0 (0.0)	0 (0.0)	0 (0.0)
Total radiotherapy dose, Gy, median (IQR)	66.0 (7.0)	66.0 (6.4)	66.0 (7.0)
Dose to oral cavity, Gy, median (IQR)	40.3 (13.9)	40.1 (10.9)	40.3 (13.3)
Baseline salivary rates, ml/min, mean ± SD	0.8 ± 0.4	0.8 ± 0.3	0.8 ± 0.4

NOTE: Body mass index (BMI) is calculated by taking the weight in kilograms and dividing it by the square of the height measured in meters. Abbreviations: *L. reuteri*, *Limosilactobacillus reuteri*; ECOG PS, Eastern Cooperative Oncology Group performance status; SD, standard deviation; a: The 'others' category included malignancies of the salivary glands, sinonasal tract, external and middle ear, and occult primaries; b: According to the AJCC 8th edition staging system.

Comment 16: *The Discussion claims better acceptability of L. reuteri compared with palifermin, yet no supporting data or references are provided.*

Response to Comment 16:

We sincerely thank you for this insightful comment. We agree that such a statement is not appropriate for our study. Accordingly, we have removed the relevant statement from the Discussion section.

Comment 17: *Clarify the exact L. reuteri strain(s) used (e.g., DSM 17938, ATCC PTA 5289).*

Response to Comment 17:

We sincerely thank you for raising this important point. We have now clarified the exact strain utilized (*Limosilactobacillus reuteri* strains DSM 17938 and ATCC PTA 5289) in the Methods section of the revised manuscript.

Accordingly, the specific revisions made in the manuscript are as follows:

Page 18, Line 370 in the revised manuscript:

"The *L. reuteri* lozenges (ProDentis, BioGaia AB, Sweden; strains DSM 17938 and ATCC PTA 5289) contained 2×10^8 live bacteria per unit."

Comment 18: *Report absolute risk reductions alongside relative risks in the Abstract.*

Response to Comment 18:

We sincerely thank you for this important suggestion. We have revised the Abstract to include both the absolute risk reduction and relative risk as recommended.

Accordingly, the specific revisions made in the manuscript are as follows:

Page 3, Line 48 in the revised manuscript:

"For the primary end point, *L. reuteri* was associated with a significant decrease in SOM incidence compared with the placebo group (37.5% vs 61.3%; relative risk 0.61; 95% confidence interval 0.44-0.85; absolute risk reduction 23.8%, 95% CI 8.7% to 38.8%; $P = 0.004$)."

Comment 19: *Consider adding a schematic summarizing hypothesized mechanisms.*

Response to Comment 19:

Thank you very much for this suggestion. We have added a schematic diagram (Supplementary Fig. 10) summarizing the hypothesized mechanisms of *L. reuteri* in the management of OM.

Accordingly, the specific revisions made in the Supplementary information are as follows:

Page 16 in the revised Supplementary information:

Supplementary Fig. 10. Schematic diagram illustrating the hypothesized mechanism of action of *Limosilactobacillus reuteri*.

[editorial note: third party material redacted]

Comment 20: *Reduce repetitive background statements in the Discussion.*

Response to Comment 20:

Thank you very much for your thoughtful and constructive suggestion. We have substantially revised the Discussion section by removing repetitive background statements, providing further interpretation of our key findings, and incorporating promising perspectives on the clinical and translational value of our study.

Reviewer #2

Comment 1: *"This manuscript describes a phase II double blinded randomized clinical trial evaluating the capability of L. reuteri to reduces severe oral mucositis (SOM) in patients with HNSCC treated with radiotherapy. The researchers included 160 eligible patients in a single center from march 2024 till January 2025. The primary endpoint was incidence of SOM, defined as grade 3 or higher on the WHO oral toxicity score. The mucositis was scored twice a week during radiotherapy and once a week till 8 weeks after radiotherapy. They found a significant reduction in SOM from 61.3% in the control group to 37.5% in the intervention group. The duration of SOM/OM was significantly shorter and the onset was delayed. This reduction of SOM also led to clinically significant reduction of toxicity burden reflected by lower use of parental feeding and lower use of analgesics. Novelty: this is the first RCT on the use of L reuteri to prevent SOM in HNSCC radiotherapy patients."*

"Comments: This is a very clear and nicely described RCT. The findings are highly relevant for the field."

Response to Comment 1:

We sincerely thank you for the careful evaluation of our manuscript and for the constructive comments and valuable suggestions, which have helped us further improve the quality and clarity of the paper. We are greatly encouraged by your overall assessment and sincerely appreciate your time and effort. We have thoroughly revised the manuscript in response to your comments, with a focus on enhancing transparency in reporting, improving the precision and standardization of terminology, and strengthening overall readability to ensure a more accessible and rigorous presentation of our work.

Comment 2: *Was the primary outcome scored by a single observer, or by many? And where the scores done by a single observer at each time point, or was there a double check? Please describe this more in detail.*

Response to Comment 2:

Thank you very much for raising this important point. All efficacy endpoints were assessed by two independent, blinded evaluators. Any discordance (defined as a ≥1-grade difference in OM scoring) was reviewed by a third senior assessor, and inconsistent or incomplete assessments were flagged for consensus discussion. We have now provided a clearer and more detailed description of this process in the Supplementary information.

Accordingly, the specific revisions made in the Supplementary information are as follows:

Page 2 in the revised Supplementary information:

"Two independent blinded evaluators assessed all efficacy endpoints, with any discordance (≥ 1-grade difference in OM scoring) reviewed by a third senior assessor. Inconsistent assessments were flagged for consensus discussion."

Comment 3: *Was there a control mechanism on the administration, I could imagine that patients stop using the medication when the suffer from SOM. Please clarify.*

Response to Comment 3:

We sincerely thank you for raising this important point. To ensure study compliance, we implemented measures both before and during the study.

Before the study, all participants received standardized education emphasizing the necessity of continuous administration of lozenges throughout the full course of radiotherapy. This measure was designed to reinforce adherence even when patients experienced SOM symptoms.

During the study, a multi-layered strategy was implemented to rigorously monitor adherence:

(1) Weekly dispensing and adherence monitoring: Study lozenges were dispensed on a weekly basis rather than as a bulk supply. Participants received a new container at each weekly visit and returned the container from the previous week, allowing direct quantification of unused lozenges.

(2) Daily medication diary: All participants were provided with a daily medication

diary (Protocol Appendix 3) to self-record the time and number of lozenges taken, as well as any missed doses.

(3) Weekly verification: During scheduled weekly follow-up visits, study personnel reviewed the diary entries together with the participant, cross-checked the count against the returned containers, and documented any discrepancies.

Together, these measures not only allowed accurate adherence monitoring but also served as ongoing reinforcement to encourage patients to take the lozenges regularly. We have now elaborated on these adherence-monitoring procedures in the Supplementary information to ensure clarity.

Accordingly, the specific revisions made in the Supplementary information are as follows:

Page 3 in the revised Supplementary information:

"Study lozenges were dispensed weekly during radiotherapy. At each scheduled weekly follow-up visit, participants received a new supply of lozenges and were required to return the container from the last week to allow direct quantitative counting of unused lozenges for adherence assessment. Furthermore, all participants were provided with a standardized daily medication diary (Protocol Appendix 3) to self-record the exact timing and number of lozenges taken, any missed doses, and any adverse events encountered during the study. During the weekly visits, trained study personnel jointly reviewed the completed medication diaries with the participants and cross-verified self-reported adherence data against the physical count of remaining

lozenges from the returned containers. Treatment adherence was calculated as the ratio of the actual number of lozenges used to the prescribed number of lozenges during radiotherapy."

Comment 4: *There is a clear correlation between SOM and dental fillings, was there a correction for that, or a standard protocol for patients with dental fillings? Please clarify.*

Response to Comment 4:

Thank you very much for this thoughtful comment. We fully agree that dental fillings may influence the SOM, and this issue was carefully considered and actively discussed by our research team during the study design phase. In response, we would like to clarify the measures taken.

First, to ensure a uniform oral baseline and minimize confounding due to heterogeneity in oral health status, all participants underwent a standardized dental evaluation conducted by a licensed dentist at baseline. For participants requiring dental fillings, the use of non-metallic fillings was recommended to reduce potential interference with radiotherapy.

In addition, given the clinical context in China, where the majority of patients receive non-metal dental fillings and metal fillings constitute a relatively small proportion[7,8], randomization was considered sufficient to achieve a balanced distribution between the two groups. Accordingly, no further statistical correction for

dental fillings was incorporated into the primary analysis.

Comment 5: *Please describe the standard protocol for oral hygiene, was that similar between both groups? Please clarify.*

Response to Comment 5:

We sincerely thank you for this valuable comment. We confirm that the oral hygiene protocol was strictly standardized and identical for all participants in both study groups throughout the course of radiotherapy. Specifically, oral hygiene education consisted of the following steps: all patients received professional dental cleaning (scaling and polishing) during the screening period, and were instructed to adhere to a standardized daily oral care routine: brushing at least twice daily with a soft-bristled toothbrush, gentle daily flossing, and rinsing the mouth with water after each meal. Specifically, after using the lozenge, the patients should avoid eating, drinking and conducting any oral hygiene activities for at least 1 hour. We have supplemented the Screening section of the Protocol with these detailed descriptions.

Accordingly, the specific revisions made in the Protocol are as follows:

Page 7 in the revised Protocol:

"Standardized oral hygiene refers to that all patients underwent a professional dental cleaning (scaling and polishing) during the screening period. And all patients were instructed to follow a standardized daily oral care regimen throughout radiotherapy, which comprised brushing with a soft-bristled toothbrush at least twice

daily, performing gentle flossing once daily, and rinsing the mouth with water after each meal. Oral hygiene education was provided by trained study personnel during the study screening period, and adherence to the oral hygiene regimen was reviewed and reinforced at each scheduled follow-up visit during the entire study course. Specifically, after using the lozenge, the patients should avoid eating, drinking and conducting any oral hygiene activities for at least 1 hour."

Comment 6: *Smoking was noted in table 1, with half of the patients smoked, were the results corrected for smoking and alcohol use? Please clarify.*

Response to Comment 6:

We sincerely thank you for this important comment. We have performed stepwise multivariate analyses to account for potential confounders of SOM/OM in the revised manuscript. Specifically, we performed additional multivariate analyses adjusted for smoking history, alcohol consumption, tumor site, oral cavity radiation dose, and concurrent chemotherapy for SOM and OM (Model 3 in Table 2). The results showed that *L. reuteri* supplementation remained a significant independent predictor of reduced SOM incidence in the ITT population (adjusted HR [95% CI]: 0.44 [0.28-0.69], $P <$ 0.001), whereas neither smoking (adjusted HR [95% CI]: 0.95 [0.55-1.64], $P = 0.85$) nor alcohol use (adjusted HR [95% CI]: 0.61 [0.36-1.05], $P = 0.07$) showed a statistically significant association with SOM incidence.

Accordingly, the specific revisions made in the manuscript are as follows:

Page 33, Line 700 in the revised manuscript:

TABLE 2 Efficacy results

Parameter	*L. reuteri* (n = 80)	Placebo (n = 80)	Effect size (95% CI)	*P*
Unadjusted primary analysis				
SOM incidence, %	37.5	61.3	0.61 (0.44-0.85)	.004
SOM onset, days, median	NE	16.0	0.43 (0.27-0.67)	<.001
SOM duration, days, median/mean (SD)	0.0/6.4 (9.6)	14.0/14.9 (14.5)	0.43 (0.21-0.66)	<.001
OM incidence, %	56.3	78.8	0.71 (0.57-0.89)	.004
OM onset, days, median	14.0	8.0	0.41 (0.28-0.60)	<.001
OM duration, days, median	14.0	35.0	0.56 (0.39-0.74)	.003
Model 1				
SOM incidence, %	37.5	61.3	0.44 (0.28-0.70)	<.001
SOM onset, days, median	NE	16.0	0.44 (0.28-0.70)	<.001
SOM duration, days, median/mean (SD)	0.0/6.4 (9.6)	14.0/14.9 (14.5)	0.46 (0.25-0.70)	.01
OM incidence, %	56.3	78.8	0.39 (0.26-0.58)	<.001
OM onset, days, median	14.0	8.0	0.39 (0.26-0.58)	<.001
OM duration, days, median	14.0	35.0	0.56 (0.40-0.74)	.004
Model 2				
SOM incidence, %	37.5	61.3	0.44 (0.28-0.69)	<.001
SOM onset, days, median	NE	16.0	0.44 (0.28-0.69)	<.001
SOM duration, days, median/mean (SD)	0.0/6.4 (9.6)	14.0/14.9 (14.5)	0.48 (0.26-0.71)	.02
OM incidence, %	56.3	78.8	0.36 (0.25-0.54)	<.001
OM onset, days, median	14.0	8.0	0.36 (0.25-0.54)	<.001
OM duration, days, median	14.0	35.0	0.57 (0.41-0.75)	.007

Abbreviations: Unadjusted incidence comparisons used χ^2 test (RR, 95% CI); onset time: log-rank test (HR, 95% CI); duration: two-part model (MR, 95%CI). Model 1 adjusted for tumor site, oral cavity radiotherapy dose and chemotherapy; Model 2 extended Model 1 by additionally adjusting for smoking history and alcohol consumption history. Cox regression was used for incidence/onset in adjusted models; two-part model for duration. *L. reuteri*, *Limosilactobacillus reuteri*; OM, oral mucositis; SOM, severe OM; SD, standard deviation; RR: relative risk; HR: hazard ratio; MR: mean ratio; CI: confidence interval; NE, not estimable.

Comment 7: *How many patients developed oropharyngeal candidiasis, and how was*

this managed? Were patients excluded from the trial? And the same for concurrent use of antibiotics for other infections (pneumonia, etc) how were these patients managed in the trial?

Response to Comment 7:

We sincerely thank you for this important comment. The management of oropharyngeal candidiasis in our study was consistent with previously published recommendations and was stratified according to severity. Mild cases were managed via risk factor modification such as smoking cessation and standardized oral care together with close monitoring; moderate cases received additional topical antifungal therapy such as nystatin oral suspension; and severe cases were treated with combined topical and systemic antifungal therapy such as oral fluconazole plus specialist consultation.[9,10] In total, 60 patients (37.5% of all enrolled patients) developed oropharyngeal candidiasis in our study, with detailed data presented in the table below. Notably, patients who received antifungal therapy for oropharyngeal candidiasis, whether local or systemic, were excluded from the trial to avoid interference with results.

Regarding antibiotic use for other infections (e.g., pneumonia), patients who used antibiotics for any reason were excluded from the study to ensure the reliability of the study results.

Table for reviewers:

Incidence, severity, and management strategies of oropharyngeal candidiasis in the

L. reuteri group and the placebo group.

Oropharyngeal candidiasis, No, (%)	*L. reuteri* (n=80)	Placebo (n=80)	All patients (n=160)	Management
No occurrence	51 (63.7)	49 (61.3)	100 (62.5)	Not applicable
Mild	25 (31.3)	27 (33.7)	52 (32.5)	Manage risk factors (e.g., smoking cessation, oral hygiene)
Moderate	4 (5)	4 (5)	8 (5)	Manage risk factors + topical antifungal therapy

Reviewer #3

Comment 1: *Thank you for the opportunity to review this piece of work. You provide compelling evidence demonstrating the mucoprotection achieved by an oral probiotic (L Reuteri), delivered as a lozenge, in people undergoing radiation (+/- chemotherapy) for head and neck cancer. This work tackles a longstanding, common yet often overlooked problem in head and neck cancer, and hence, the significance of this work cannot be underestimated. I sincerely want to thank you for your level of commitment to this major clinical problem. Overall, the manuscript is well presented, the study is rigorously designed and executed and the findings are of interest to the supportive oncology community. However, the manuscript itself is somewhat superficial is its analyses and interpretation, and would benefit from a deeper level of thought and interpretation, particularly with respect to existing work (published by you/your group) on another probiotic, Ssk12. Below are some points that I feel need to be acknowledged in your work before it can be considered for publication.*

Response to Comment 1:

We are deeply grateful for your careful review and expert guidance on our

manuscript. Your constructive comments and suggestions are crucial in significantly enhancing the academic rigor, clarity, and overall quality of our work. We are greatly encouraged by your favorable overall assessment and sincerely appreciate the considerable time and effort you have devoted to improving our paper.

Comment 2: *The justification for the use of this specific strain requires more explanation, specifically related to its role in OM pathogenesis. I note you mentioned "In addition, our basic research showed that L reuteri has efficacy in managing RIOM" (Line 79), however there is no reference. Are you referring to the data presented in this manuscript, or past data? If the data in this manuscript, I would suggest removing this from the introduction as you cannot justify a strain selected based on not yet generated preclinical data. If it has been published, please reference.*

Response to Comment 2:

We sincerely thank you for these insightful comments. First, we have added foundational references regarding the specific role of *L. reuteri* in OM pathogenesis to the Introduction. Specifically, the main mechanisms include the formation of protective biofilms, inhibition of pathogenic bacteria, alleviation of oxidative stress, and maintenance of mucosal barrier integrity. Second, the statement noted above was derived from the present study. We fully agree that citing data generated within the present study may not be appropriate. Therefore, we have removed the statement from the Introduction.

Accordingly, the specific revisions made in the manuscript are as follows:

Page 5, Line 86 in the revised manuscript:

"Among various probiotic strains, *Limosilactobacillus reuteri* (*L. reuteri*) exhibits notable potential for managing OM through multiple, complementary mechanisms.[10,11] A key attribute is its ability to form stable, protective biofilms on oral epithelia, which promotes mucosal colonization and competitively excludes pathogenic bacteria.[12,13] Furthermore, *L. reuteri* targets oxidative stress via its metabolite reuterin to reduce direct tissue damage and enhances epithelial barrier integrity, synergistically countering key drivers of mucosal dysfunction in OM.[14-17] Together, these actions help restore microbial balance and dampen inflammatory cascades, underpinning its documented efficacy in oral inflammatory diseases such as gingivitis and periodontitis.[18-22]"

Comment 3: *Following from point 1, the biggest question I have with this work is why the pivot from Ssk12. You have previously shown that Ssk12 was clinically effective in reducing RIOM (published din JCO, 2023). So what is the justification for testing a new strain? Please include a detailed justification of the rationale of testing a new strain, rather than pushing forward with implementing Ssk12 after your highly successful Phase II trial.*

Response to Comment 3:

We sincerely thank you for these insightful and constructive comments. The main reason we conducted this study was the difference in mechanisms between the two

probiotic strains. Specifically, in addition to the competitive exclusion and direct inhibition of pathogenic microorganisms exhibited by *SsK12*, *L. reuteri* also actively modulates the host inflammatory microenvironment and maintains mucosal homeostasis.[1-4] On the other hand, we sought not only to explore the effectiveness of *L. reuteri* in relieving RIOM, but also to explore whether the protective mechanism of probiotics against RIOM is strain specific or a common property. These considerations were the main rationale for undertaking this study. We have carefully discussed the differences between our study and the *SsK12* study, and emphasized the rationale for conducting this study in the Discussion section.

Accordingly, the specific revisions made in the manuscript are as follows:

Page 12, Line 257 in the revised manuscript:

"Prior clinical trials have indicated that specific probiotic strains, including *Lactobacillus brevis CD2*, *Streptococcus salivarius K12* (*SsK12*), and probiotic combinations containing *Bifidobacterium longum*, *Lactobacillus lactis*, and *Enterococcus faecium* have demonstrated significant efficacy in reducing RIOM.[3,26,27] Collectively, these convergent beneficial effects across distinct strains raise important questions regarding the mechanistic basis of probiotic protection against RIOM. As proposed by Wardill et al., such conserved efficacy suggests that clinical benefit may not arise exclusively from strain-specific metabolic properties, but rather from the general ability of probiotic microorganisms to occupy ecological niches and competitively restrict pathogen colonization.[28] Notably, *L. reuteri* may extend beyond

these shared mechanisms by additionally modulating the host inflammatory microenvironment and preserving mucosal homeostasis, thereby reinforcing its protective role against RIOM.[29-31]"

Comment 4: *Were randomisation schedules stratified in any way? e.g. by inclusion of chemotherapy, diagnostic criteria, HPV status?*

Response to Comment 4:

We sincerely thank you for raising this important point. No stratification factors were applied during randomization. We agree that the absence of stratification may increase the risk of prognostic imbalance, and we have acknowledged this limitation in the revised manuscript. To address your concern, we examined the baseline distribution of key prognostic variables between treatment groups. All tests showed that the two arms were comparable at baseline, with no statistically significant imbalances in these parameters.

Accordingly, the specific revisions made in the manuscript are as follows:

Page 15, Line 305 in the revised manuscript:

"First, the single-center design and lack of stratified randomization may limit generalizability and introduce potential prognostic imbalances across the heterogeneous tumor sites and variable radiation doses involved."

Table for reviewers:

Baseline characteristics of the study population. Values are presented as number

(percentage). Categorical variables were compared using the chi-square test or Fisher's exact test, as appropriate.

Characteristic	*L. reuteri* (n = 80)	Placebo (n = 80)	*P*
Sex, No. (%)			0.23
Male	68 (85.0)	61 (76.2)	
Female	12 (15.0)	19 (23.8)	
Age, years, mean ± SD	56.3 ± 11.3	57.4 ± 12.4	0.56
BMI, kg/m^2, mean ± SD	22.9 ± 3.6	22.6 ± 2.4	0.52
ECOG PS, No. (%)			0.99
0	54 (67.5)	53 (66.2)	
1	24 (30.0)	25 (31.2)	
2	2 (2.5)	2 (2.5)	
Tumor pathologic types, No. (%)			0.23
Squamous carcinoma	67 (83.8)	73 (91.2)	
Others	13 (16.2)	7 (8.8)	
Tumor site, No. (%)			1.00
Nasopharynx	24 (30.0)	24 (30.0)	
Oral cavity	22 (27.5)	22 (27.5)	
Oropharynx	13 (16.2)	13 (16.2)	
Hypopharynx	10 (12.5)	10 (12.5)	
Larynx	8 (10.0)	7 (8.8)	
Others [a]	3 (3.8)	4 (5.0)	
Treatment type, No. (%)			0.87
Definitive	38 (47.5)	40 (50.0)	
Postoperative treatment	42 (52.5)	40 (50.0)	
Concurrent chemotherapy, No. (%)			0.75
Yes	37 (46.2)	34 (42.5)	
No	43 (53.8)	46 (57.5)	
TNM stage [b], No. (%)			0.93
I-II	18 (22.5)	16 (20.0)	
III	23 (28.7)	22 (27.5)	
IVa	25 (31.2)	29 (36.2)	
IVb	14 (17.5)	13 (16.2)	
Tumor primary site, No. (%)			0.81
T1	6 (7.5)	9 (11.2)	
T2	26 (32.5)	26 (32.5)	
T3	23 (28.7)	24 (30.0)	
T4	25 (31.2)	21 (26.2)	
Nodal involvement, No. (%)			0.84

N0	28 (35.0)	25 (31.2)	
N1	14 (17.5)	15 (18.8)	
N2	26 (32.5)	24 (30.0)	
N3	12 (15.0)	16 (20.0)	
Tobacco use, No. (%)			0.53
Yes	38 (47.5)	43 (53.8)	
No	42 (52.5)	37 (46.2)	
Alcohol use, No. (%)			0.27
Yes	43 (53.8)	35 (43.8)	
No	37 (46.2)	45 (56.2)	
Oropharyngeal cancer HPV status, No. (%)			0.70
Positive	5 (38.5)	3 (23.1)	
Negative	8 (61.5)	10 (76.9)	
Nasopharynx cancer EBV status, No. (%)			1.00
Positive	24 (100.0)	24 (100.0)	
Negative	0 (0.0)	0 (0.0)	
Total radiotherapy dose, Gy, median (IQR)	66.0 (7.0)	66.0 (6.4)	0.39
Dose to oral cavity, Gy, median (IQR)	40.3 (13.9)	40.1 (10.9)	0.79
Baseline salivary rates, ml/min, mean ± SD	0.8 ± 0.4	0.8 ± 0.3	0.59

Comment 5: *The quality of life tool was performed repeatedly over the course of the study (weekly). This is quite high burden on participants. Did you see much attrition in the rate of completion? How were missing data handled?*

Response to Comment 5:

Thank you very much for raising these important points. The QoL assessments did not impose a substantial burden on participants in our study. Point-by-point responses are displayed below.

First, regarding the potential burden of repeated QoL assessments, the radiotherapy protocol required patients to visit the hospital daily for treatment and attend weekly follow-up visits regardless of study participation. The completion of QoL questionnaires was integrated into these scheduled weekly visits and therefore did not

impose additional time or travel burden beyond the standard care routine. With the exception of 19 patients (11.9%) who withdrew from the study, all participants in the PPS completed the QoL questionnaires.

The high adherence and completion rates observed in our study may be attributed to a combination of cultural and institutional factors. In China, Confucian values emphasizing collective well-being and respect for medical authority contribute to strong physician-patient trust and high treatment compliance, which is reflected in participation rates that often exceed those seen in Western settings. For example, Professor Shen Lin's phase III randomized controlled trial at Peking University Cancer Hospital (published in *Journal of Clinical Oncology*, 2021) achieved an enrollment rate of 89.6% (328/366), substantially higher than typical Western trial participation rates of 30-50%.[11] Additionally, our study was conducted at West China Hospital of Sichuan University, a leading national medical center consistently ranked as the second-best hospital in the China Hospital Rankings, where strong institutional reputation and patient trust further supported retention and adherence throughout the study period.

Regarding the handling of missing data, all analyses for QoL outcomes were pre-specified to be conducted in the PPS. Participants who discontinued treatment or withdrew consent were excluded from the PPS according to the pre-defined statistical plan. For all participants included in the PPS, the QoL questionnaires were fully completed at every scheduled assessment time point. Consequently, there were no missing QoL data within this analysis set, and no imputation methods were applied.

Comment 6: *Saliva collection was achieved by spitting. Based on my experience with this patient population, it is extremely difficult to collect saliva after week 3 of treatment due to xerostomia. Can you please comment, or include more information on how saliva collection was achieved in cases where xerostomia was present. Also please include the number saliva samples collected per participant per timepoints, separated by treatment group. I am curious as to whether there are biases creeping into your microbiome data based on selected saliva sampling.*

Response to Comment 6:

We sincerely thank you for the comments. For patients with significant xerostomia, we employed a standardized cotton swab absorption method-an approach that has been validated in prior studies as a reliable means of assessing salivary secretion for patients with xerostomia.[12,13] Specifically, a pre-weighed sterile cotton swab was placed in the patient’s mouth for 5 minutes to absorb saliva, then weighed again and immediately frozen for subsequent analysis.

We fully agree with your concern that the spitting method and the cotton swab absorption method may introduce potential bias into the microbiome data. To address this, we analyzed the microbiota composition between saliva and swab samples collected at the end of radiotherapy. The results showed no significant differences in either α diversity or β diversity between the groups, suggesting that the sample collection method did not introduce systematic bias into the microbiome analysis.

We have supplemented and described the detailed procedures of saliva collection in the Supplementary information. Furthermore, we have added detailed information on the number of saliva samples collected from two groups at each time point in the table below. Meanwhile, the salivary microbial profiles from the two collection methods are shown in the figure below.

Accordingly, the specific revisions made in the Supplementary information are as follows:

Page 3 in the revised Supplementary information:

"For patients with xerostomia, saliva was collected using a cotton swab absorption method.[4,5] Sterile cotton swabs were pre-weighed and subsequently placed in the patient's oral cavity for 5 minutes to allow for maximal saliva absorption, after which the swabs were removed and weighed again. All samples were immediately frozen at -20°C and transferred to -80°C for long-term storage until further analysis."

Table for reviewers:

Number and type of samples collected between two groups at each timepoint. Percentages are based on the number of participants from whom samples were collected at each time point.

Timepoint	Sample type, No (%)	*L. reuteri* (n=80)	Placebo (n=80)
Baseline	Saliva	80 (100.0)	80 (100.0)
	Swab	0 (0.0)	0 (0.0)
Mid-radiotherapy	Saliva	72 (92.3)	70 (90.9)
	Swab	6 (7.7)	7 (9.1)
End-radiotherapy	Saliva	39 (54.9)	40 (57.1)
	Swab	32 (45.1)	30 (42.9)

Figure for reviewers:

Comparison of oral microbial diversity between swab and saliva samples at the end of radiotherapy. α diversity (Sobs and Shannon indices, Fig. a-b) and β diversity (PCoA at ASV level, Fig. c) were analyzed by Wilcoxon rank-sum test and PERMANOVA, respectively.

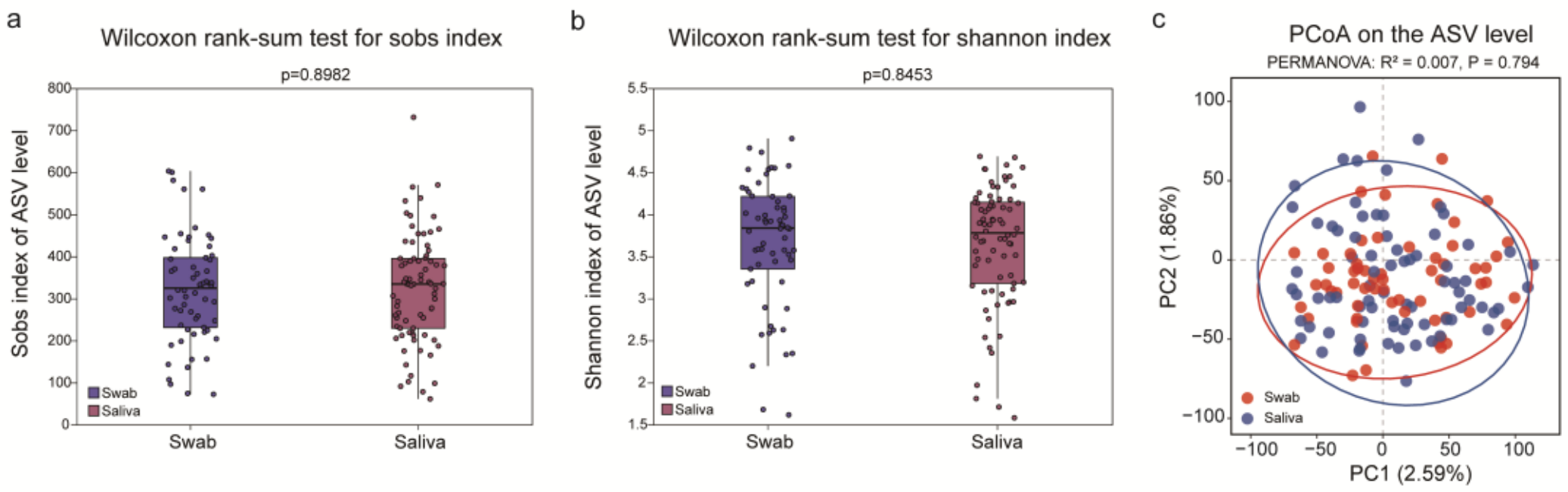

Comment 7: *How were confounders handled, especially HPV status and smoking status. Please review this paper on how to handle mucositis confounders: https://pubmed.ncbi.nlm.nih.gov/35639187/ and incorporate appropriate methodology. At the very least, reporting HPV+/- rates in your treatment groups would be needed. Similarly, stratification or your data based on the addition of chemotherapy would be interesting. Do you see different efficacy signals in radiation vs chemoradiation?*

Response to Comment 7:

We sincerely thank you for your valuable comments and suggestions. We have carefully reviewed the recommended article (PubMed ID: 35639187) and have supplemented additional analyses in the revised manuscript, as detailed below:

(1) Reporting of HPV status: We have clearly reported the distribution of HPV-positive and HPV-negative among oropharynx cancer patients within each group in the

baseline characteristics table (Table 1) of the manuscript. Specifically, among the enrolled patients with oropharyngeal cancer, there were 8 positive cases (30.8%) and 18 negative cases (69.2%).

(2) Adjustment for confounders: We conducted stepwise adjustment for potential confounders in the efficacy analyses. For the ITT set, Model 1 included tumor site, oral cavity radiation dose, and concurrent chemotherapy; Model 2 further added smoking history and alcohol consumption based on Model 1. For the PPS population, Model 2 was further adjusted for the Shannon index in addition to smoking history and alcohol consumption. Due to the small number of oropharyngeal cancer patients (the only subgroup for which HPV testing is recommended), the limited sample size precluded convergence of the statistical model in HPV-stratified analyses. We therefore did not include HPV status as a covariate in the statistical models, but instead performed a subgroup analysis stratified by HPV status, which revealed no statistically significant difference between the two groups. The results of major confounding factors on SOM and OM were presented in Supplementary Table 6.

(3) Subgroup and stratified analyses: We have added subgroup analyses of the primary outcome (SOM incidence) across key clinical subgroups, including radiotherapy intent, use of concurrent chemotherapy, smoking history, alcohol history, oral cavity radiation dose, and tumor site. In the subgroups receiving concurrent chemoradiotherapy and definitive radiotherapy, the results showed that *L. reuteri* supplementation still significantly reduced the incidence of SOM. The results were now

presented in the revised Supplementary Fig. 3.

In summary, we have comprehensively addressed the issue of confounding factors by incorporating additional statistical analyses. The results showed that after adjusting for the aforementioned common clinical confounding factors, *L. reuteri* remained a protective factor against OM.

Accordingly, the specific revisions made in the manuscript and Supplementary information were as follows:

Page 31, Line 693 in the revised manuscript:

TABLE 1 Baseline patient characteristics

Characteristic	No. (%) of Patients		
	L. reuteri (n = 80)	Placebo (n = 80)	All Patients (N = 160)
Sex, No. (%)			
Male	68 (85.0)	61 (76.2)	129 (80.6)
Female	12 (15.0)	19 (23.8)	31 (19.4)
Age, years, mean ± SD	56.3 ± 11.3	57.4 ± 12.4	56.8 ± 11.8
BMI, kg/m^2, mean ± SD	22.9 ± 3.6	22.6 ± 2.4	22.8 ± 3.0
ECOG PS, No. (%)			
0	54 (67.5)	53 (66.2)	107 (66.9)
1	24 (30.0)	25 (31.2)	49 (30.6)
2	2 (2.5)	2 (2.5)	4 (2.5)
Tumor pathologic types, No. (%)			
Squamous carcinoma	67 (83.8)	73 (91.2)	140 (87.5)
Others	13 (16.2)	7 (8.8)	20 (12.5)
Tumor site, No. (%)			
Nasopharynx	24 (30.0)	24 (30.0)	48 (30.0)
Oral cavity	22 (27.5)	22 (27.5)	44 (27.5)
Oropharynx	13 (16.2)	13 (16.2)	26 (16.2)
Hypopharynx	10 (12.5)	10 (12.5)	20 (12.5)
Larynx	8 (10.0)	7 (8.8)	15 (9.4)
Others [a]	3 (3.8)	4 (5.0)	7 (4.4)
Treatment type, No. (%)			
Definitive	38 (47.5)	40 (50.0)	78 (48.8)

Postoperative treatment	42 (52.5)	40 (50.0)	82 (51.2)
Concurrent chemotherapy, No. (%)			
Yes	37 (46.2)	34 (42.5)	71 (44.4)
No	43 (53.8)	46 (57.5)	89 (55.6)
TNM stage [b], No. (%)			
I-II	18 (22.5)	16 (20.0)	34 (21.2)
III	23 (28.7)	22 (27.5)	45 (28.1)
IVa	25 (31.2)	29 (36.2)	54 (33.8)
IVb	14 (17.5)	13 (16.2)	27 (16.9)
Tumor primary site, No. (%)			
T1	6 (7.5)	9 (11.2)	15 (9.4)
T2	26 (32.5)	26 (32.5)	52 (32.5)
T3	23 (28.7)	24 (30.0)	47 (29.4)
T4	25 (31.2)	21 (26.2)	46 (28.7)
Nodal involvement, No. (%)			
N0	28 (35.0)	25 (31.2)	53 (33.1)
N1	14 (17.5)	15 (18.8)	29 (18.1)
N2	26 (32.5)	24 (30.0)	50 (31.2)
N3	12 (15.0)	16 (20.0)	28 (17.5)
Tobacco use, No. (%)			
Yes	38 (47.5)	43 (53.8)	81 (50.6)
No	42 (52.5)	37 (46.2)	79 (49.4)
Alcohol use, No. (%)			
Yes	43 (53.8)	35 (43.8)	78 (48.8)
No	37 (46.2)	45 (56.2)	82 (51.2)
Oropharyngeal cancer HPV status, No. (%)			
Positive	5 (38.5)	3 (23.1)	8 (30.8)
Negative	8 (61.5)	10 (76.9)	18 (69.2)
Nasopharynx cancer EBV status, No. (%)			
Positive	24 (100.0)	24 (100.0)	48 (100.0)
Negative	0 (0.0)	0 (0.0)	0 (0.0)
Total radiotherapy dose, Gy, median (IQR)	66.0 (7.0)	66.0 (6.4)	66.0 (7.0)
Dose to oral cavity, Gy, median (IQR)	40.3 (13.9)	40.1 (10.9)	40.3 (13.3)
Baseline salivary rates, ml/min, mean ± SD	0.8 ± 0.4	0.8 ± 0.3	0.8 ± 0.4

NOTE: Body mass index (BMI) is calculated by taking the weight in kilograms and dividing it by the square of the height measured in meters. Abbreviations: *L. reuteri*, *Limosilactobacillus reuteri*; ECOG PS, Eastern Cooperative Oncology Group performance status; SD, standard deviation; a: The 'others' category included malignancies of the salivary glands, sinonasal tract, external and middle ear, and occult primaries; b: According to the AJCC 8th edition staging system.

Page 33, Line 700 in the revised manuscript:

TABLE 2 Efficacy results

Parameter	*L. reuteri* (n = 80)	Placebo (n = 80)	Effect size (95% CI)	*P*
Unadjusted primary analysis				
SOM incidence, %	37.5	61.3	0.61 (0.44-0.85)	.004
SOM onset, days, median	NE	16.0	0.43 (0.27-0.67)	<.001
SOM duration, days, median/mean (SD)	0.0/6.4 (9.6)	14.0/14.9 (14.5)	0.43 (0.21-0.66)	<.001
OM incidence, %	56.3	78.8	0.71 (0.57-0.89)	.004
OM onset, days, median	14.0	8.0	0.41 (0.28-0.60)	<.001
OM duration, days, median	14.0	35.0	0.56 (0.39-0.74)	.003
Model 1				
SOM incidence, %	37.5	61.3	0.44 (0.28-0.70)	<.001
SOM onset, days, median	NE	16.0	0.44 (0.28-0.70)	<.001
SOM duration, days, median/mean (SD)	0.0/6.4 (9.6)	14.0/14.9 (14.5)	0.46 (0.25-0.70)	.01
OM incidence, %	56.3	78.8	0.39 (0.26-0.58)	<.001
OM onset, days, median	14.0	8.0	0.39 (0.26-0.58)	<.001
OM duration, days, median	14.0	35.0	0.56 (0.40-0.74)	.004
Model 2				
SOM incidence, %	37.5	61.3	0.44 (0.28-0.69)	<.001
SOM onset, days, median	NE	16.0	0.44 (0.28-0.69)	<.001
SOM duration, days, median/mean (SD)	0.0/6.4 (9.6)	14.0/14.9 (14.5)	0.48 (0.26-0.71)	.02
OM incidence, %	56.3	78.8	0.36 (0.25-0.54)	<.001
OM onset, days, median	14.0	8.0	0.36 (0.25-0.54)	<.001
OM duration, days, median	14.0	35.0	0.57 (0.41-0.75)	.007

Abbreviations: Unadjusted incidence comparisons used χ^2 test (RR, 95% CI); onset time: log-rank test (HR, 95% CI); duration: two-part model (MR, 95%CI). Model 1 adjusted for tumor site, oral cavity radiotherapy dose and chemotherapy; Model 2 extended Model 1 by additionally adjusting for smoking history and alcohol consumption history. Cox regression was used for incidence/onset in adjusted models; two-part model for duration. *L. reuteri*, *Limosilactobacillus reuteri*; OM, oral mucositis; SOM, severe OM; SD, standard deviation; RR: relative risk; HR: hazard ratio; MR: mean ratio; CI: confidence interval; NE, not estimable.

Page 8 in the revised Supplementary information:

Supplementary Fig. 3. Subgroup analysis of severe oral mucositis incidence.

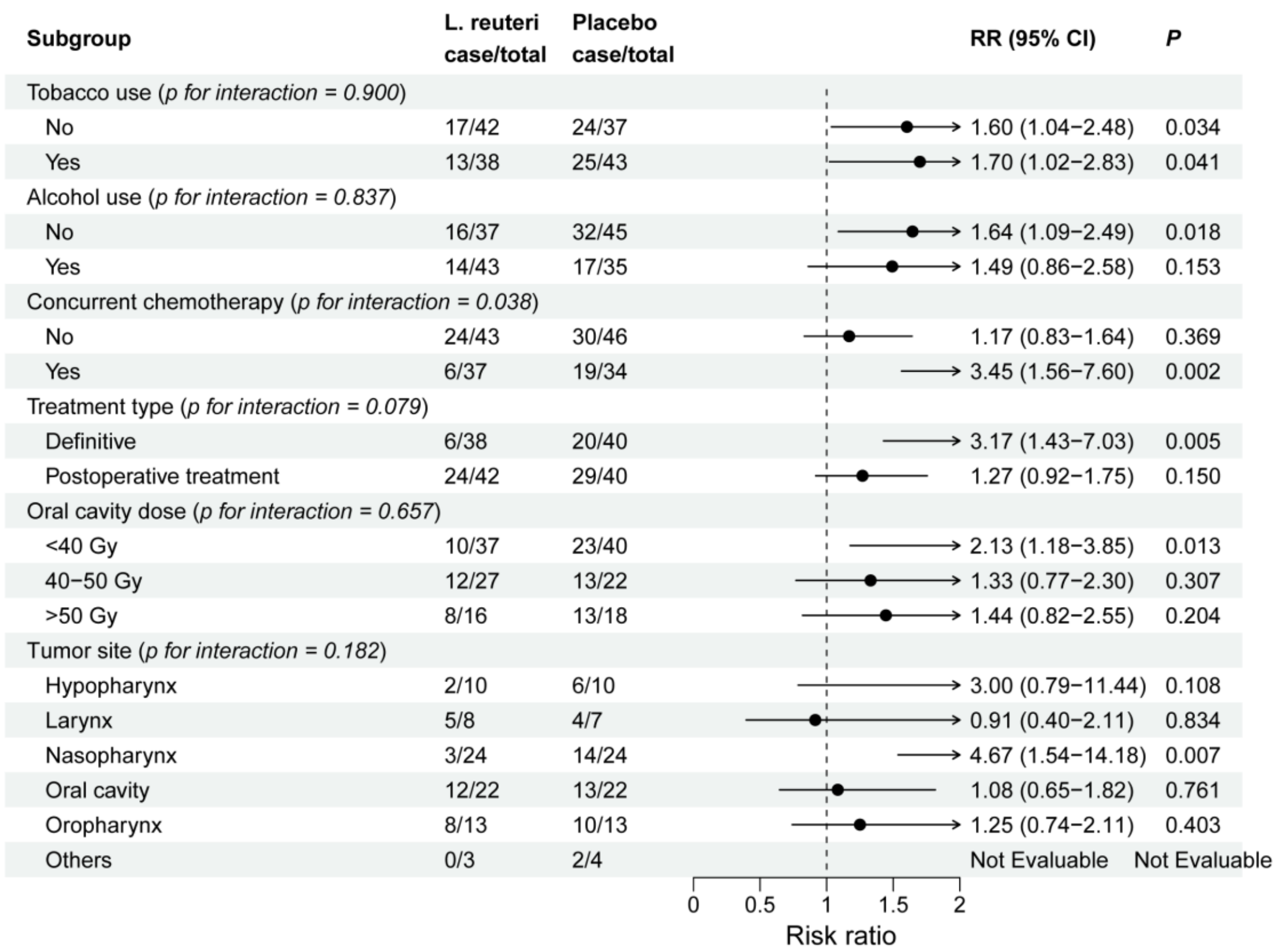

Risk ratios (RRs) and 95% CIs were estimated using modified Poisson regression with robust standard errors.

Comment 8: *Your microbiome data is interesting - were you surprised at the subtleness of the changes observed in your placebo group. It certainly doesn't appear that there is profound "dysbiosis" occurring. If anything, the shifts look more apparent in your probiotic group. Please comment. In addition, there is considerable divergence in the baseline alpha diversity between probiotic and placebo. This is concerning and certainly needs to be discussed in your paper.*

Response to Comment 8:

Thank you for these insightful comments on our microbiome data.

Firstly, regarding the "subtle" changes in the placebo group versus more apparent shifts in the probiotic group: we agree that the placebo group did not exhibit profound

dysbiosis during radiotherapy, whereas the *L.reuteri* group showed more pronounced changes. In our study, while the placebo group did not display marked alterations in α diversity and β diversity over time, we observed increased enrichment of specific pathogenic taxa (*Alloscardovia omnicolens* and *Lancefieldella rimae*) at the end of radiotherapy. In contrast, the *L. reuteri* group demonstrated notable enrichment of *L. reuteri* and other potentially beneficial taxa, including *Rothia mucilaginosa* and *Granulicatella adiacens*, which indicates distinct differences in microbial community composition between the two groups.

Secondly, we acknowledge your concern regarding the divergence in baseline α diversity between the two groups. The human microbiome is characterized by substantial inter-individual heterogeneity even in the context of randomized controlled trial. Furthermore, the relatively small sample size of our study may have contributed to baseline imbalances in microbial metrics. To address your concern and ensure the robustness of our efficacy findings, we have taken the following steps:

(1) Expanded discussion: We have added a dedicated paragraph in the Discussion section to acknowledge the baseline microbial divergence and recognize it as a study limitation.

(2) Multivariable statistical adjustment: We included baseline Shannon index and other key clinical factors as covariates in the statistical models for the efficacy analysis of the PPS. Even after accounting for baseline Shannon index, *L. reuteri* supplementation remained a significant independent factor associated with a reduced

incidence and duration of SOM (Supplementary Table 4).

(3) Expanded analysis of oral microbiota at baseline: We performed additional analyses to explore whether baseline oral microbiota composition differed according to OM status. The results showed that no significant differences in α diversity or β diversity between patients with OM and patients without OM at baseline (Supplementary Fig. 11a-c).

Accordingly, the specific revisions made in the manuscript are as follows:

Page 7, Line 136 in the revised manuscript:

"In the PPS population, consistent results were observed even after incorporating baseline microbial alpha diversity (Shannon index) into the Model 2 framework (adjusted relative risk 0.37; 95% CI 0.22-0.64; $P < 0.001$; Supplementary Table 5)."

Page 15, Line 312 in the revised manuscript:

" Additionally, despite statistical adjustment, the baseline divergence in oral microbiota between cohorts may still confound the interpretation of the intervention's independent effects."

Page 16 in the revised Supplementary information:

Supplementary Fig. 11. Salivary microbiota profiles stratified by OM status at baseline and overall between the *Limosilactobacillus reuteri* and placebo groups.

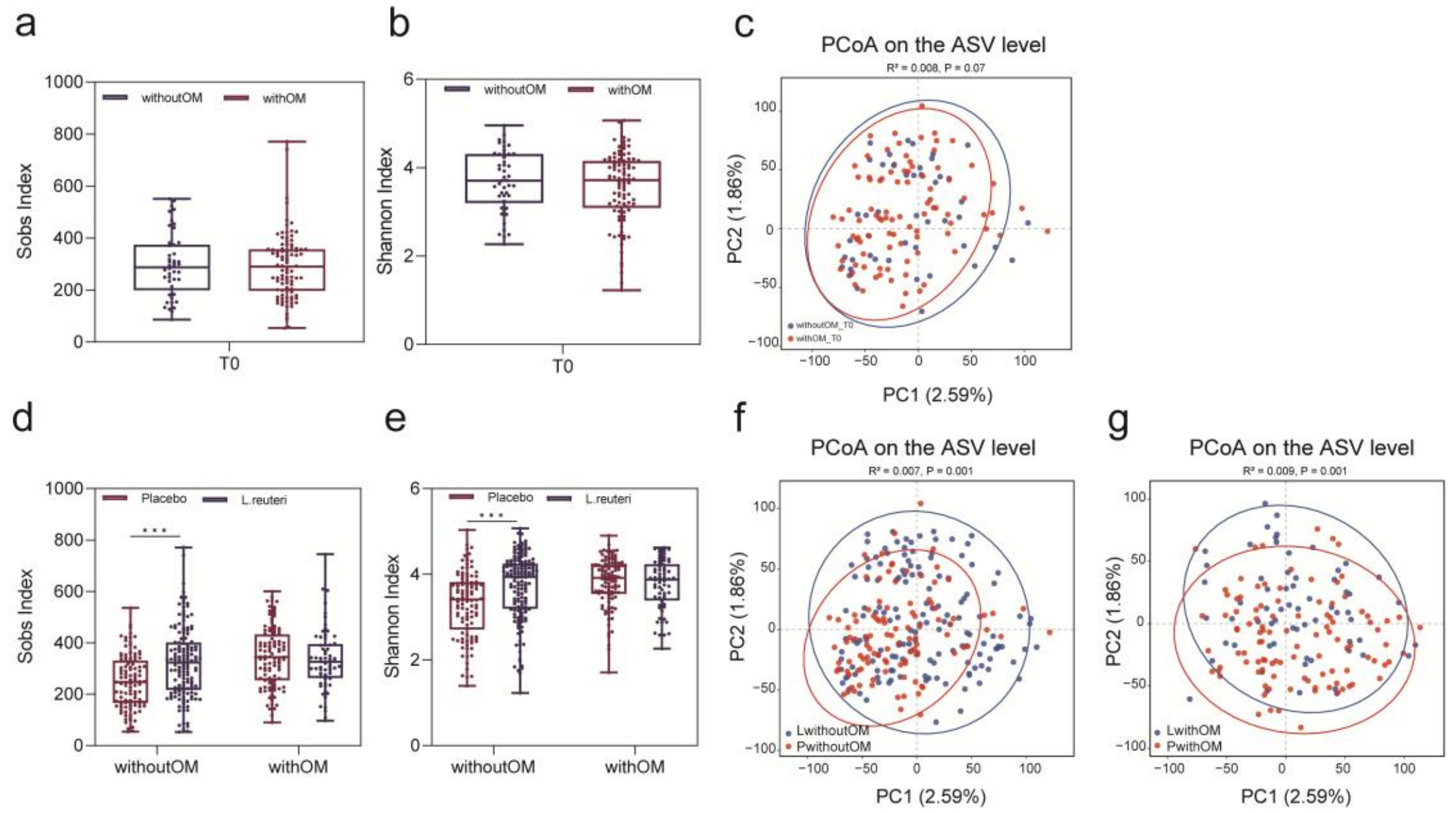

(**a-b**) Alpha diversity: Sobs and Shannon indices comparing salivary microbiota profiles stratified by oral mucositis (OM) status at baseline. (**c**) Beta diversity: Principal Coordinates Analysis (PCoA) plots based on Aitchison distance at the ASV level illustrating salivary microbiota profiles at T0, stratified by OM status. Statistical significance was assessed by PERMANOVA (R^2 and *P* values are indicated). (**d-e**) Alpha diversity: Sobs and Shannon indices comparing the *L. reuteri* and placebo groups, stratified by OM status. Boxplots depict the median, interquartile range, and range. (**f-g**) Beta diversity: PCoA plots based on Aitchison distance at the ASV level illustrating beta diversity differences between the *L. reuteri* and placebo groups, stratified by OM status. (**f**) Patients without OM; (**g**) Patients with OM. Abbreviations: OM, oral mucositis; SOM, severe oral mucositis; withOM, patients with oral mucositis; withoutOM, patients without oral mucositis; withSOM, patients with severe oral mucositis; withoutSOM, patients without severe oral mucositis; ASV, amplicon sequence variant; PCoA, principal coordinates analysis.

Comment 9: *There were minimal differences in the QoL outcomes between your experimental groups, with the exception of pain. Can you please comment on this. I would hypothesise that such a profound improvement in mucositis would translate into a positive QoL signal. In the absence of such signal, it is difficult to justify why this probiotic should be taken forward. Alternatively, you may like to consider flow on effects of controlling mucositis on clinical service provision. Is this something that can be extracted from clinical records? e.g. emergency presentations, admissions, length of*

stay, radiation dose reductions or modifications, insertion of a feeding tube, infection, supportive care medication use (e.g. not just opioids, but also antibiotics). Could you use this to model the economic benefit of your intervention?

Response to Comment 9:

We sincerely thank you for this insightful comment. Although no statistically significant differences were observed in other QoL dimensions, there was a favorable trend toward improved QoL in the *L. reuteri* group, particularly in domains related to oral function and daily activity. In addition, we extracted data on hospital admissions and length of stay from the electronic medical records to further explore the potential impact of *L. reuteri* on clinical service provision. The results showed that the *L. reuteri* group had lower hospitalization rates and a shorter mean length of stay compared with the placebo group. Furthermore, our data demonstrated that the *L. reuteri* group had significantly lower rates of parenteral nutrition use (23.9% vs 54.3%; relative risk = 0.44, 95% CI 0.28-0.70; $P < 0.001$) and analgesic use (7.0% vs 21.4%; relative risk = 0.33, 95% CI 0.13-0.87; $P = 0.03$) compared with the placebo group during radiotherapy. These findings indicate that *L. reuteri* supplementation effectively reduces the requirement for certain supportive care interventions, which holds certain clinical relevance for patients with head and neck cancer undergoing radiotherapy. We have supplemented the hospitalization rates and mean length of stay for both groups in the table below.

Accordingly, the specific revisions made in the manuscript are as follows:

Page 12, Line 246 in the revised manuscript:

"Beyond symptom control, the supplementation of *L. reuteri* correlated with a decreased utilization of parenteral nutrition and analgesics relative to placebo. This reduction in high-intensity supportive care implies potential economic benefits through optimized resource utilization."

Table for reviewers:

Hospitalization rates and mean length of stay in the *L. reuteri* and placebo groups. Rehospitalization rates were compared using the chi-square test. Mean length of stay was compared using the Wilcoxon rank-sum test.

Outcome	*L. reuteri* (n = 80)	Placebo (n = 80)	*P*
Rehospitalization, n (%)	8 (10.0)	14 (17.5)	0.251
Length of stay, days, mean ± SD	7.5 ± 2.6	8.6 ± 2.1	0.388

Comment 10: *You mention in your methods that both IIT and PP analyses were performed. In the results, it is unclear which has been used. Can you please report on both in your results, or make it explicitly clear what analysis was used.*

Response to Comment 10:

We sincerely thank you for raising this important point. The primary efficacy results presented were based on the ITT population in the main manuscript. In parallel, the corresponding analyses for the PPS have been conducted and were presented in full in Supplementary Table 5 for complete transparency. We have revised the Methods and Results section to explicitly state which analysis set is being reported in our manuscript.

Accordingly, the specific revisions made in the manuscript are as follows:

Page 20, Line 432 in the revised manuscript:

"Efficacy analyses were performed both on the Intention-To-Treat (ITT) set, which included all randomized patients, and on the Per-Protocol Set (PPS), defined as patients who completed the study. The primary analysis presented in the manuscript is based on the ITT population. The corresponding analysis for the PP population was provided in the Supplementary information."

Comment 11: *Why was the onset of (days) for RIOM not estimable in your intervention group (Table 2)?*

Response to Comment 11:

We sincerely thank you for raising this important point. The efficacy analyses presented in Table 2 were based on the ITT population. The median time to onset could not be reliably estimated in the *L.reuteri* group, because the incidence of SOM in this group was below 50% from the start of radiotherapy until the end of the study period. To further illustrate the between-group differences in efficacy, we performed an analysis restricted to participants who developed OM in the ITT population and PP population. The median time to onset of SOM was 28.0 days in the *L. reuteri* group and 21.0 days in the placebo group in the ITT set restricted to participants who developed OM, respectively (Supplementary Table 6).

Page 23 in the revised Supplementary information:

Supplementary Table 6. Efficacy results in patients with oral mucositis.

Analysis Set	Endpoint	*L.reuteri*	Placebo	Effect size (95%CI)	*P*
Participants in the ITT population who developed OM (N=108)	**Unadjusted primary analysis**				
	SOM duration, days, median	15.5	21.0	0.69 (0.55-0.85)	<.001
	SOM onset, days, median	28.0	21.0	0.40 (0.25-0.64)	<.001
	OM duration, days, median	38.0	46.0	0.78 (0.65-0.92)	.003
	OM onset, days, median	20.0	9.0	0.46 (0.31-0.68)	<.001
	**Adjusted model**				
	SOM duration, days, median	15.5	21.0	0.76 (0.61-0.93)	0.012
	SOM onset, days, median	28.0	21.0	0.36 (0.21-0.60)	<.001
	OM duration, days, median	38.0	46.0	0.77 (0.64-0.91)	0.004
	OM onset, days, median	20.0	9.0	0.37 (0.24-0.57)	<.001
Participants in the PP Set who developed OM (N=95)	**Unadjusted primary analysis**				
	SOM duration, days, median	18.0	25.0	0.70 (0.56-0.87)	.002
	SOM onset, days, median	28.0	20.0	0.29 (0.17-0.52)	<.001
	OM duration, days, median	39.0	49.0	0.79 (0.66-0.95)	.013
	OM onset, days, median	18.5	9.0	0.40 (0.26-0.61)	<.001
	**Adjusted model**				
	SOM duration, days, median	18.0	25.0	0.78 (0.63-0.97)	0.031
	SOM onset, days, median	28.0	20.0	0.25 (0.14-0.46)	<.001
	OM duration, days, median	39.0	49.0	0.78 (0.64-0.95)	0.015
	OM onset, days, median	18.5	9.0	0.33 (0.21-0.53)	<.001

Abbreviations: Unadjusted onset time comparisons used log-rank test (HR, 95% CI); duration: two-part model (MR, 95%CI). Adjusted model adjusted for tumor site, oral radiotherapy dose and chemotherapy. Cox regression was used for onset in adjusted models; two-part model for duration. *L. reuteri*, *Limosilactobacillus reuteri*; OM, oral mucositis; SOM, severe OM; MR: mean ratio; HR: hazard ratio; CI: confidence interval.

Comment 12: *I am extremely surprised at the very low rate of drop out. In my experience, this is a complex patient cohort with high needs. Can you please comment on why you had such low drop out. This is especially surprising in the placebo group who had no benefit. In my experience, when people are required to adhere to such a long intervention program (like this) and experience no benefit, the rate of drop out is high, or at least, the rate of adherence is low. Can you please comment on this.*

Response to Comment 12:

Thank you very much for this thoughtful comment. Our study was conducted at West China Hospital of Sichuan University, consistently ranked as the nation's second-best hospital in the China Hospital Rankings. As a leading national medical center, our institution enjoys exceptional patient trust and compliance rates.

The radiotherapy protocol required daily outpatient treatments with weekly follow-up visits, and our intervention did not impose an excessive time burden on patients. These high adherence rates align with findings from other top-tier Chinese medical institutions. For example, Professor Shen Lin's phase III randomized controlled trial at Peking University Cancer Hospital (published in *Journal of Clinical Oncology*, 2021) achieved an 89.6% enrollment rate (328/366), significantly exceeding typical Western trial participation rates of 30-50%.[11]

We attribute these variations to China's distinctive cultural milieu, where Confucian principles prioritizing collective well-being cultivate stronger physician-patient alliances and higher treatment adherence rates as opposed to more

individualistic societies. This cultural lens elucidates the outstanding compliance observed in our investigation.

Comment 13: *Following on from point 11 - there is no mention or discussion of adherence to the intervention. Was this recorded. Please include data on this. This is especially important as, in the context of mucositis and xerostomia, the feasibility of consuming sticky lozenges each day for 6-7 weeks seems challenging. Adherence should be considered in your ITT vs PP analyses. In line with this, I acknowledge that you reviewed Lactobacillus abundance as an indication of L. Reuteri colonisation, however feel this could have been achieved with greater precision. Could you perform targeted PCR for the probiotic strain in saliva samples to truly determine colonisation. This again may assist in more nuanced analyses, where efficacy could be strategies by the rate/completeness of colonisation.*

Response to Comment 13:

We sincerely appreciate your constructive and insightful comments. The point-by-point response is as follows:

(1) Adherence to the intervention: We have supplemented the revised manuscript with the methodology for adherence evaluation and the corresponding results. Specifically, treatment adherence was calculated as the ratio of the actual number of lozenges used to the prescribed number of lozenges during radiotherapy. The mean adherence rate was 94.3% in the *L. reuteri* group and 92.6% in the placebo group in the

ITT population, and 96.4% and 95.0% in the PP population, respectively.

(2) Targeted PCR for *L. reuteri* colonization: We fully agree that targeted PCR would provide a much more precise quantification of *L. reuteri* colonization. Given the insufficient residual microbial biomass in the preserved saliva samples, we performed a detailed longitudinal analysis of *L. reuteri* abundance to address your concern as thoroughly as possible. The results demonstrated that the relative abundance of *L. reuteri* was significantly higher in the intervention group at mid-radiotherapy and at the end of radiotherapy compared with the placebo group, which may indicate successful colonization of *L. reuteri*. However, we acknowledge that strain-specific PCR analysis may more accurately reflect the colonization of *L. reuteri*. We are committed to addressing this in future investigations. We have provided a figure showing the relative abundance of *L. reuteri* in both groups at each timepoint below.

Accordingly, the specific revisions made in the manuscript are as follows:

Page 18, Line 378 in the revised manuscript:

"Treatment adherence was calculated as the ratio of the actual number of lozenges used to the prescribed number of lozenges during radiotherapy."

Page 6, Line 113 in the revised manuscript:

"The mean adherence rate was 94.3% in the *L. reuteri* group and 92.6% in the placebo group in the ITT population, and 96.4% and 95.0% in the PP population, respectively."

Table for reviewers:

Adherence rate in the *L. reuteri* and placebo groups. Adherence rate was calculated as the ratio of the actual number of lozenges used to the prescribed number of lozenges during radiotherapy. Adherence rates were compared using the Wilcoxon rank-sum test.

Adherence rate (%), mean ± SD	*L. reuteri*	Placebo	*P*
Intention-to-Treat Set	94.3 ± 10.3	92.6 ± 11.5	0.063
Per Protocol Set	96.4 ± 3.57	95.0 ± 4.56	0.051

Figure for reviewers:

Longitudinal changes in relative abundance of *L. reuteri* at the species level. In both the placebo and *L. reuteri* groups, the relative abundance of *L. reuteri* initially increased and subsequently decreased across the three time points, which may reflect a degree of self-regulation by the oral microbiota. Notably, the *L. reuteri* group showed significantly higher abundance of *L. reuteri* compared with the placebo group at all post-intervention time points (Kruskal-Wallis test, *P* = 0.007), suggesting successful colonization by the administered probiotic. Abbreviations: T0, baseline; T1, mid-radiotherapy; T2, end-of-radiotherapy.

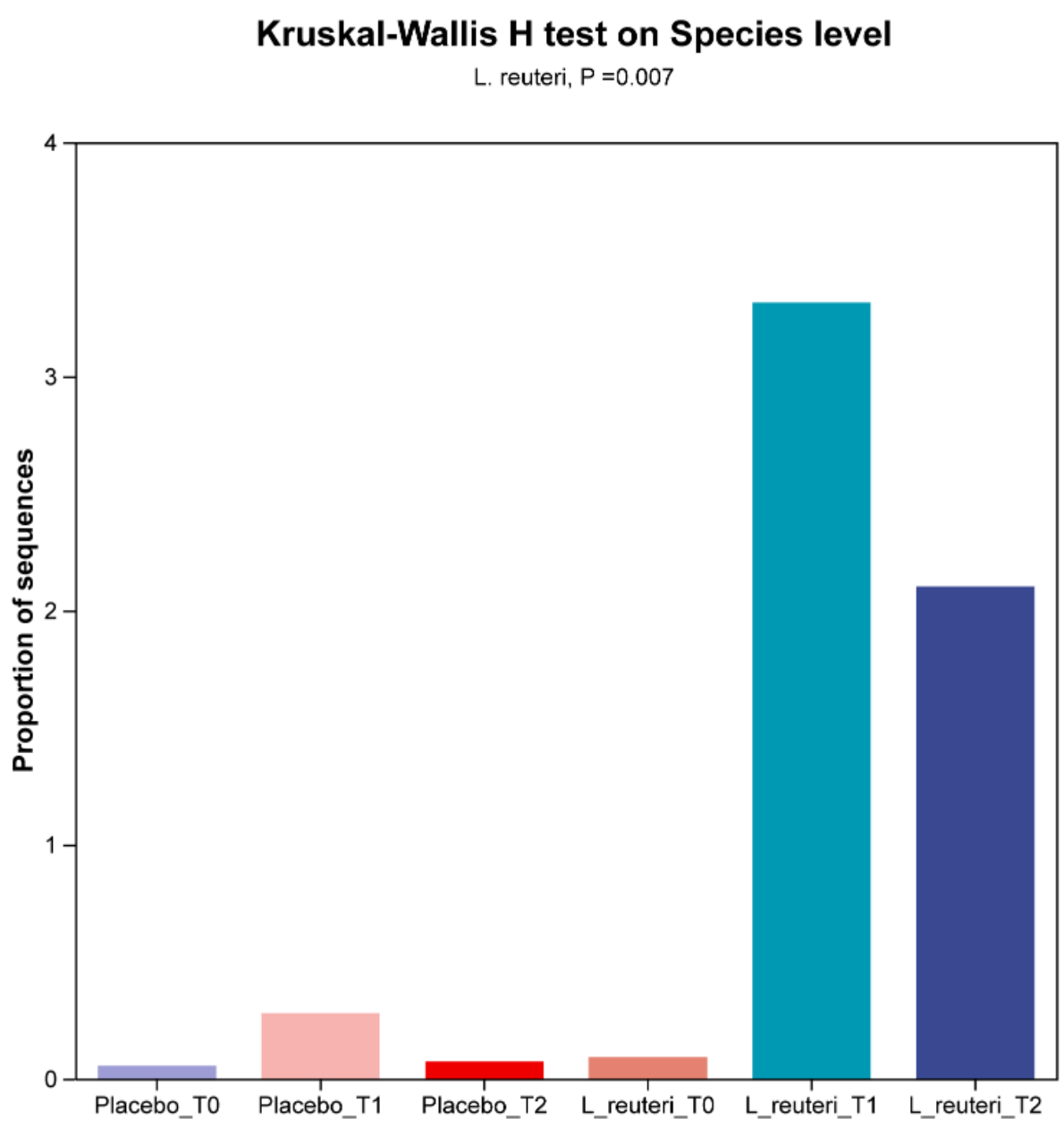

Comment 14: *The discussion would benefit from less repetition of results and more mechanistic thought / forward gazing. One key observation I have made from these data is that the efficacy signal is near identical to that you report in your JCO paper on Ssk12. There needs to be discussion on what this means mechanistically. To me, and as written in my JCO UTP paper (Wardill, Sonis, Bossi, 2024), there is a distinct possibility that the efficacy of both Ssk12 and LR are related not to the strain, but simply the sheer presence of a probiotic micro-organism that is capable of displacing or restricting pathogen blooms. Please comment on this, and relate your findings to your Ssk12 data more explicitly, particularly in terms of translating these promising clinical findings into a standard of care clinical intervention. Which one would you choose? Should you even choose one? Would there be greater efficacy if they were combined? Similarly - you could consider the concept of responder/non-responder, i.e. were there microbial attributes at baseline that dictate response?*

Response to Comment 14:

We truly thank you for this highly insightful and constructive comment. We fully agree with you that probiotic interventions may represent a broader ecological strategy centered on displacing or restricting pathogen blooms. Regarding the comparison between the two strains, we are unable to conclude which one is superior, as a head-to-head comparison of the two RCTs is not feasible. Each strain possesses its own distinct characteristics. However, in addition to the competitive exclusion and direct inhibition of pathogenic microorganisms exhibited by *SsK12*, *L. reuteri* also actively modulates

the host inflammatory microenvironment and maintains mucosal homeostasis.[1-4] We fully agree with you that combining the two strains might achieve greater efficacy, and we plan to conduct this research in future studies. Furthermore, we fully agree with you on the concept of responder/non-responder. Selecting specific strains for intervention based on baseline differences in patients' oral microbiota may enable more targeted interventions and achieve greater efficacy.

We have substantially revised the Discussion section to explicitly discuss the findings of *L. reuteri* in the present study and our previous clinical results with *Ssk12*, along with evidence from other probiotic studies.

Accordingly, the specific revisions made in the manuscript are as follows:

Page 12, Line 257 in the revised manuscript:

"Prior clinical trials have indicated that specific probiotic strains, including *Lactobacillus brevis CD2*, *Streptococcus salivarius K12* (*SsK12*), and probiotic combinations containing *Bifidobacterium longum*, *Lactobacillus lactis*, and *Enterococcus faecium* have demonstrated significant efficacy in reducing RIOM.[3,26,27] Collectively, these convergent beneficial effects across distinct strains raise important questions regarding the mechanistic basis of probiotic protection against RIOM. As proposed by Wardill et al., such conserved efficacy suggests that clinical benefit may not arise exclusively from strain-specific metabolic properties, but rather from the general ability of probiotic microorganisms to occupy ecological niches and competitively restrict pathogen colonization.[28] Notably, *L. reuteri* may extend beyond

these shared mechanisms by additionally modulating the host inflammatory microenvironment and preserving mucosal homeostasis, thereby reinforcing its protective role against RIOM.[29-31]"

Comment 15: *The preclinical model showed almost complete prevention of OM. This is extremely challenging to achieved and may indeed be cause for concern if it is impacting radiation toxicity so profoundly ... is it therefore influencing efficacy? I would recommend reviewing/including clinical data on treatment response, i.e. RECiST at 3 months post treatment to confirm your probiotic is not influencing radiation efficacy. Similarly, the level of protection you see in your preclinical model is much higher than what you see clinically. This should be discussed in light of the dose justification (both mice and men receiving the same dose of probiotic - thus relative to body size, the mice are getting a lot more microbes. Does this explain the higher efficacy seen in mice?).*

Response to Comment 15:

We sincerely thank you for these constructive comments.

Firstly, we have now added clinical tumor response data for our patient cohort at 3 months post-radiotherapy. As presented in the revised Results section, the objective response rates showed no statistically significant difference between the *L.reuteri* and placebo groups (Supplementary Table 10).

Secondly, the difference in the magnitude of OM protection observed between the preclinical model and the clinical cohort can be interpreted based on several interrelated

factors. As you pointed out, due to the difference in oral mucosal surface area between mice and humans, the relative probiotic dose administered per unit of mucosal surface area was substantially higher in mice than in patients. Furthermore, the total radiation dose delivered to mice was considerably lower than that received by human patients according to biologically effective dose. The highly controlled and standardized conditions of preclinical studies also contrast with the clinical setting, which is complicated by tumor heterogeneity, concurrent chemotherapy, and variable patient comorbidities. Collectively, these factors likely account for the stronger protective effect observed in the preclinical model. We have incorporated the relevant discussion into the Discussion section of the revised manuscript.

Accordingly, the specific revisions made in the manuscript are as follows:

Page 11, Line 230 in the revised manuscript:

"Among the enrolled patients, 51.2% (82/160) underwent surgery before radiotherapy, whereas 48.8% (78/160) received definitive radiotherapy and were therefore assessed for tumor response at 3 months after radiotherapy. Among patients who underwent surgery, distant metastasis occurred in one patient in the *L. reuteri* group and two patients in the placebo group, while one patient in each group experienced local recurrence during follow-up. According to RECIST v1.1 criteria, the complete response rates and partial response rates were 89.5 % (34/38) and 7.9% (3/38) in the *L. reuteri* group versus 90.0% (36/40) and 5.0% (2/40) in the placebo group among patients treated with definitive radiotherapy (Supplementary Table 10)."

Page 15, Line 310 in the revised manuscript:

"Third, the clinical efficacy was more modest than observed in murine models, likely reflecting translational discrepancies in relative probiotic dosing and biologically effective radiation doses."

Page 27 in the revised Supplementary information:

Supplementary Table 10. Tumor response in patients receiving definitive radiotherapy between *Limosilactobacillus reuteri* group and placebo group.

Response	*L. reuteri* (n=38)	Placebo (n=40)
Complete response, No. (%)	34 (89.5)	36 (90.0)
Partial response, No. (%)	3 (7.9)	2 (5.0)
Progressive disease, No. (%)	1 (2.6)	2 (5.0)

NOTE. Tumor response assessments were conducted at baseline and 3 months post-radiotherapy in patients with head and neck cancer receiving definitive radiotherapy. Radiographic evaluation included contrast-enhanced CT or MRI of the head and neck, contrast enhanced CT of the thorax, and abdominal ultrasound. Changes in tumor size were evaluated according to RECIST v1.1 criteria.

Comment 16: *What is the justification of stopping the animal model at day 8? What happens beyond day 8? Perhaps you haven't totally prevented OM, but instead delayed its onset?*

Response to Comment 16:

We sincerely thank you for raising this important point.

Firstly, the decision to terminate the animal at day 8 was based on animal welfare regulations and preliminary experimental data. The animal model used in our study is widely accepted and well-established, and in accordance with institutional ethical guidelines, mice were euthanized when body weight loss exceeded 20%.[14,15] Our

preliminary experimental data showed that mice subjected to 18 Gy oral cavity irradiation typically developed visible oral ulceration and reached the critical threshold of ⩾20% body weight loss by approximately day 8 post-irradiation. Accordingly, this time point was chosen as a humane and clinically relevant endpoint for evaluating the severity of OM. We have added a clarification regarding the endpoint choice in the Methods section of Supplementary information.

Secondly, we fully acknowledge your insightful comment that the intervention may have delayed the onset of OM rather than completely preventing it in the murine model. We have explicitly stated in the Results section of the fundamental experiments that the intervention may have delayed but not fully prevented OM onset. Importantly, our clinical data further demonstrated that *L. reuteri* supplementation not only delayed the onset of OM but also significantly reduced its overall incidence in the patient cohort, supporting a genuine protective effect beyond a simple delay.

Accordingly, the specific revisions made in the Supplementary information are as follows:

Page 2 in the revised Supplementary information:

"At the experimental endpoint (D8), all animals were euthanized due to ethical requirements following >20% body weight loss, and each group was further randomly divided into two subgroups for macroscopic (n=4 per subgroup) and histopathological (n=3 per subgroup) assessment."

Page 6 in the revised Supplementary information:

"It should be acknowledged that the mice were euthanized at this time point per animal ethics criteria, such that the intervention may have delayed but not fully prevented OM onset."

Comment 17: *The term dysbiosis is used throughout this manuscript and while I appreciate its convenience, it is increasingly scrutinized for its lack of specificity. Hence I strongly suggest amending your language throughout to conform with modern recommendations. https://pmc.ncbi.nlm.nih.gov/articles/PMC7017827/*

Response to Comment 17:

We sincerely thank you for this valuable suggestion. We have carefully read the recommended literature (PMC7017827) and systematically revised the manuscript to replace the term "dysbiosis" with more precise descriptors that reflect the actual microbial changes observed in our study.

Comment 18: *You may like to consider including this reference to support the role of the microbiome in OM - this was published by MASCC as an addendum to their pathogenesis framework. I transparently disclose that I am a co-author on this manuscript. https://link.springer.com/article/10.1007/s00520-024-08752-4.*

Response to Comment 18:

We sincerely thank you for this suggestion. We have now incorporated this citation in the revised Introduction section to strengthen the contextual background supporting

the rationale for probiotic intervention in RIOM.

Accordingly, the specific revisions made in the manuscript are as follows:

Page 4, Line 82 in the revised manuscript:

"Accumulating findings suggest that the radiotherapy-induced restructuring of the oral microbiota, specifically characterized by the depletion of commensal taxa and the concomitant enrichment of pathogenic taxa, contributes to the development and progression of RIOM, and that oral probiotics may mitigate mucosal injury by restoring microbial homeostasis.[3,7-9]"

[9] Stringer, A. M. et al. Updated perspectives on the contribution of the microbiome to the pathogenesis of mucositis using the MASCC/ISOO framework. Support Care Cancer 32, 558 (2024). https://doi.org:10.1007/s00520-024-08752-4.

Comment 19: *Some figures and tables would benefit from re-ordering of data, e.g, it feels awkward to have the intervention before the placebo, and much more natural to present placebo first. Similarly, in Figure 4e, starting with T2>>T0 (left to right) feels counterintuitive.*

Response to Comment 19:

Thank you very much for this important suggestion. We have carefully reviewed all figures and tables in the manuscript and have revised them accordingly.

Comment 20: *The rate of opioid use seems to be very low. In our experience, this patient*

group has rates of opioids use >90%. Please comment on this.

Response to Comment 20:

Thank you very much for this insightful comment. The lower rates of opioid use in our cohort can be primarily attributed to the characteristics of our Chinese patient population and cultural context, as explained below:

First, patients in China often hold excessive concerns about the potential for opioid addiction and related side effects, which may lead them to decline opioid prescriptions.[16,17]

Second, traditional cultural values emphasize endurance and stoicism rather than explicit expression of discomfort, especially among Chinese male patients, who tend to tolerate pain rather than actively seek analgesic treatment.[18,19]

Third, some patients in our cohort held religious or philosophical beliefs (e.g., Buddhism, Taoism) that encourage acceptance of suffering as part of life's journey, which may further reduce their willingness to seek pharmacological pain relief.[19,20]

Taken together, these cultural, social, and psychological factors unique to the Chinese population likely contributed to the low observed rate of opioid use in our study.

Comment 21: *Please provide more clarity on why N=7 mice/group were used, but then downstream analyses are only performed on N=3-4. How were the subgroups defined/selected?*

Response to Comment 21:

We sincerely thank you for raising this important point. In each group, 7 mice were used to observe the development of OM in both the *L.reuteri* and saline groups. Since macroscopic evaluation requires staining, it cannot be performed on the same mice used for histopathological analysis. Therefore, upon euthanasia at the experimental endpoint, 7 mice per group were randomly allocated to two distinct assessment modalities: macroscopic evaluation (n=4 per group) and histopathological analysis (n=3 per group), an approach that is consistent with previous studies.[15] We confirm that all subgroup assignments were performed randomly. We have supplemented a more detailed description of the randomization and allocation procedure in the Supplementary information.

Accordingly, the specific revisions made in the Supplementary information are as follows:

Page 2 in the revised Supplementary information:

"At the experimental endpoint (D8), all animals were euthanized due to ethical requirements following >20% body weight loss, and each group was further randomly divided into two subgroups for macroscopic (n=4 per subgroup) and histopathological (n=3 per subgroup) assessment."

Reviewer #4

Comment 1: *This is a high-significant, well-designed clinical trial that assessed the efficacy of Lactobacillus reuteri for prevention of radiation-induced oral mucositis (OM)*

in head and neck cancer patients. The trial was randomized and double-blinded in concordance with the CONSORT guidelines. The study found to an intervention with Lactobacillus reuteri containing lozenges twice a day to substantially and significantly reduce incidence, time-to-onset and duration as well as severity of OM, which is if replicated in other cohorts, can transform clinical management of head and neck cancer patients undergoing radiotherapy.

Response to Comment 1:

We sincerely thank you for the careful evaluation of our manuscript and for the constructive comments and valuable suggestions. Your expertise in the field of microbiology provided us with exceptionally professional guidance, and we greatly appreciate the time and effort you dedicated to our work. We have carefully addressed all points raised and revised the manuscript accordingly.

Comment 2: *The manuscript is well written and organized. The methods are sufficiently described in the main text and supplementary information. Patients' clinical characteristics and results of analysis of primary, secondary and exploratory outcomes are adequately and well presented, although it is recommended to present results from adjusted analysis in the main text and move unadjusted results to the supplementary.*

Response to Comment 2:

We sincerely thank you for the positive and constructive comments on our study. In accordance with your recommendations, we have moved the results from the

adjusted analyses into the main text.

Comment 3: *It is important to note that Lactobacillus reuteri has been reclassified as Limosilactobacillus reuteri, so the nomenclature should be updated in the manuscript accordingly. Moreover, a recent randomized clinical trial (PMID: 40887814) reported limited efficacy of L. reuteri in a similar clinical context. The study should be acknowledged in the introduction and discussed in comparison with the findings of the present study.*

Response to Comment 3:

We sincerely thank you for the careful review and valuable comments. We have globally updated the nomenclature in our manuscript. All instances of "*Lactobacillus reuteri*" have been revised to "*Limosilactobacillus reuteri*" across the main text, figures, tables, and Supplementary information to ensure compliance with the latest bacterial taxonomy.

Regarding the recent randomized clinical trial (PMID: 40887814), it is important to note that its subgroup analysis of patients treated with radiotherapy alone demonstrated that *L. reuteri* supplementation reduced OM severity scores - a finding that aligns closely with the results of our study. The absence of overall positive findings in that trial may be attributable to its small sample size (19 participants in the probiotic arm, 18 in the placebo arm; total n=37) and patient population heterogeneity. Notably, the majority of patients in that study received concurrent chemoradiotherapy (23/37,

62.2%), which differs from the patient population in our study (71/160, 44.4%). We have cited and discussed this study in detail in the Introduction and Discussion sections of the revised manuscript.

Accordingly, the specific revisions made in the manuscript are as follows:

Page 5, Line 97 in the revised manuscript:

"Despite promising preliminary findings, a recent randomized clinical trial reported controversial results, highlighting that its effectiveness in managing RIOM is not yet fully understood and requires further investigation.[23]"

Page 12, Line 249 in the revised manuscript:

"In contrast to our findings, a recent randomized clinical trial found no overall benefit of *L. reuteri* against OM, which may be explained by factors including its sample size and patient population heterogeneity.[23] Notably, subgroup analysis of patients receiving radiotherapy alone demonstrated reduced OM severity, which is consistent with our primary findings. These findings suggest that *L. reuteri* may represent a promising prophylactic strategy to improve supportive care in patients undergoing radiotherapy."

Comment 4: *While the trial is reported to have been registered under the identifier NCT06285591, no corresponding record can be found on ClinicalTrials.gov. This discrepancy should be clarified by the authors.*

Response to Comment 4:

We sincerely thank you for raising this important point. We would like to clarify that the trial was indeed successfully registered on ClinicalTrials.gov under the identifier NCT06285591. We have verified that the record is currently active and publicly accessible. The direct link to the trial registration is provided below for your verification: https://clinicaltrials.gov/study/NCT06285591.

Comment 5: *Substantial biological data is lost by restricting the analysis to the genus level. Using appropriate classification algorithm, e.g. blastn, and reference database (such as the Human Oral Microbiome Database, HOMD), ASVs generated from V3-4 sequencing can be reliably classified to the species level, which is more biologically meaningful for interpreting association with mucosal health and disease, as health and disease associated species frequently belong to the same genus.*

Response to Comment 5:

We sincerely thank you for this constructive suggestion. Following your recommendation, we have completely re-analyzed our microbiome data. Specifically, we used the blastn algorithm for taxonomic classification and adopted the HOMD as the primary reference database. Consequently, all ASVs have been re-classified and all downstream analyses, including α and β diversity, taxonomic composition plots, and differential abundance testing, have been updated to reflect species-level results throughout the revised manuscript.

Accordingly, the specific revisions made in the Supplementary information are as

follows:

Page 3 in the revised Supplementary information:

"Following sequencing, raw sequencing reads were processed to generate amplicon sequence variants (ASVs) using the DADA2 plugin in QIIME2.[6,7] ASV tables were then rarefied to 20,000 reads per sample to normalize sequencing depth prior to downstream analyses. Taxonomic assignment of ASVs was performed using BLASTn[7] (version 2.12.0) against the Human Oral Microbiome Database (HOMD, version 15.22), with a minimum identity threshold of 80% and query coverage of 90% to classify at the species level. All bioinformatics analyses were conducted on the Majorbio Cloud Platform (Majorbio, Shanghai, China; available at: www.majorbio.com)."

Comment 6: *The bioinformatic analysis pipeline used is suboptimal. In addition to the low taxonomic resolution, the methods used for beta diversity (UniFrac distances) and differential abundance analysis (LEfSe) are not compositional data aware (CDA). In addition, LEfSe does not appropriately account for multiple testing, as it does not report false discovery rate (FDR)-adjusted results. It is highly recommended to reanalyze the data using appropriate CDA methods such as Aitchison distances calculated from CLR transformed data for beta diversity and CLR transformed data with regression models adjusting for both confounders and FDR (such as MaAslin) for differential abundance analysis.*

Response to Comment 6:

We sincerely thank you for these highly insightful comments. We have comprehensively revised our statistical and bioinformatic methods. For β diversity analysis, we now employ Aitchison distances derived from CLR transformed data. For differential abundance analysis, we have replaced our previous approach with MaAsLin2, which incorporates appropriate confounder adjustments (including age, sex, BMI, smoking history, and alcohol consumption) and applies FDR correction to account for multiple testing. All corresponding results presented in the revised manuscript have been thoroughly updated to reflect these improved analytical methodologies.

Accordingly, the specific revisions made in the manuscript are as follows:

Page 19, Line 409 in the revised manuscript:

"Linear mixed-effects models were used to assess group differences in α diversity indices over time. β diversity was assessed using Aitchison distances calculated from centered log-ratio (CLR)-transformed taxon counts, with principal coordinate analysis (PCoA) applied to visualize microbial community structure. Statistical comparisons of β diversity were performed using PERMANOVA. For differential abundance analysis, the Kruskal-Wallis test was used among multiple groups, and the Wilcoxon rank-sum test was used for two-group comparisons. Multivariable association testing was conducted using MaAsLin2 with age, sex, BMI, smoking, and alcohol use as fixed effects and subject as a random effect. *P* values in all analyses were adjusted for multiple testing using the false discovery rate (FDR) method. Functional profiling of

microbial communities was performed using PICRUSt2."

Page 3 in the revised Supplementary information:

"Linear mixed-effects models were used to assess the differences in α diversity indices (Shannon and Sobs) between the *L. reuteri* and placebo groups over time. β diversity was assessed using Aitchison distances, calculated from centered log-ratio (CLR) transformed taxon counts to account for the compositional nature of relative abundance data. Principal coordinate analysis (PCoA) was applied to visualize shifts in microbial community structure and statistically compared using PERMANOVA. All analyses were conducted in R v4.4.0. Community heatmaps were generated at the species level using Z-score normalized mean abundance values (by row) for the top 30 taxa. Kruskal-Wallis rank sum tests were performed at the species level to identify differentially abundant taxa across groups, with unclassified taxa excluded prior to analysis. For two group comparisons, the Wilcoxon rank sum test was applied. To further account for potential confounding factors, we applied multivariable association testing using MaAsLin2, including age, sex, BMI, smoking, and alcohol use as fixed effects, and subject as a random intercept for repeated measurements. *P* values in both analyses were adjusted for multiple testing using the false discovery rate (FDR) method. Functional profiling of microbial communities was performed using PICRUSt2 (v2.2.0-b)."

Comment 7: *Data analysis lacks sufficient depth and integration with the clinical*

findings. For example, no comparisons of the microbiome were made between patients who developed and didn't develop OM/SOM, both overall and within each study arm. This is particularly important since beta-diversity results presented in the Supplementary information show a significant difference in baseline microbiomes between the two study arms, despite randomization and comparable baseline clinical characteristics. This raises concern that observed differences in clinical outcomes may be driven, at least in part, by underlying microbiome differences rather than the intervention itself. Accordingly, the microbiome may need to be included as a variable in the adjusted analysis. Furthermore, while the PCoA plots indicate significant microbial community composition between timepoints in each study arm, the specific microbial shifts at the genus and species levels are not described or correlated with outcomes.

Response to Comment 7:

We sincerely thank you for these insightful and constructive suggestions. We have conducted additional analyses and revised the manuscript accordingly, as detailed below:

1. Baseline microbiome imbalance: We included baseline Shannon index and other key clinical factors as covariates in the statistical models for the efficacy analysis of the PPS. Even after accounting for baseline Shannon index, *L. reuteri* supplementation remained a significant independent factor associated with a reduced incidence and duration of SOM (Supplementary Table 4). Furthermore, we have re-analyzed α and β

diversity at baseline stratified by patients' OM status. Both α diversity and β diversity showed no significant differences between patients who developed OM and those who did not at baseline (Supplementary Fig. 11 a-c).

2. Microbiome comparisons by OM status and study intervention: We have supplemented the manuscript with comprehensive microbiome comparisons stratified by OM status, both overall and between the two groups.

For between-group comparisons, regarding α diversity, the *L. reuteri* group exhibited significantly higher α diversity compared with the placebo group among patients who did not develop OM (Supplementary Fig. 11 d-e). With respect to β diversity, significant differences were observed between the *L. reuteri* and placebo groups when stratified by OM status (Supplementary Fig. 11 f-g).

For the overall cohort analysis, the results demonstrated that patients with ongoing OM exhibited significantly lower α diversity and distinct β diversity composition compared with those without OM (Supplementary Fig. 12 a-c), which is consistent with previous findings.[21] In addition, differential abundance analysis revealed enrichment of probiotic taxa including *L. reuteri* in patients without OM, whereas pathogenic taxa such as *Fusobacterium nucleatum* were enriched in those who developed OM (Supplementary Fig. 12 d-e).

These results suggest that microbial dysbiosis is an important factor influencing OM, and that *L. reuteri* may play a role in reversing this dysbiosis. We have integrated these findings into the revised manuscript and Supplementary information.

Accordingly, the specific revisions made in the manuscript and Supplementary information are as follows:

Page 10, Line 206 in the revised manuscript:

“In addition, we performed microbiome comparisons in the overall cohort stratified by OM status and between the *L. reuteri* and placebo groups; the results were consistent with the above findings (Supplementary Fig. 11-12).”

Page 16 in the revised Supplementary information:

Supplementary Fig. 11. Salivary microbiota profiles stratified by OM status at baseline and overall between the *Limosilactobacillus reuteri* and placebo groups.

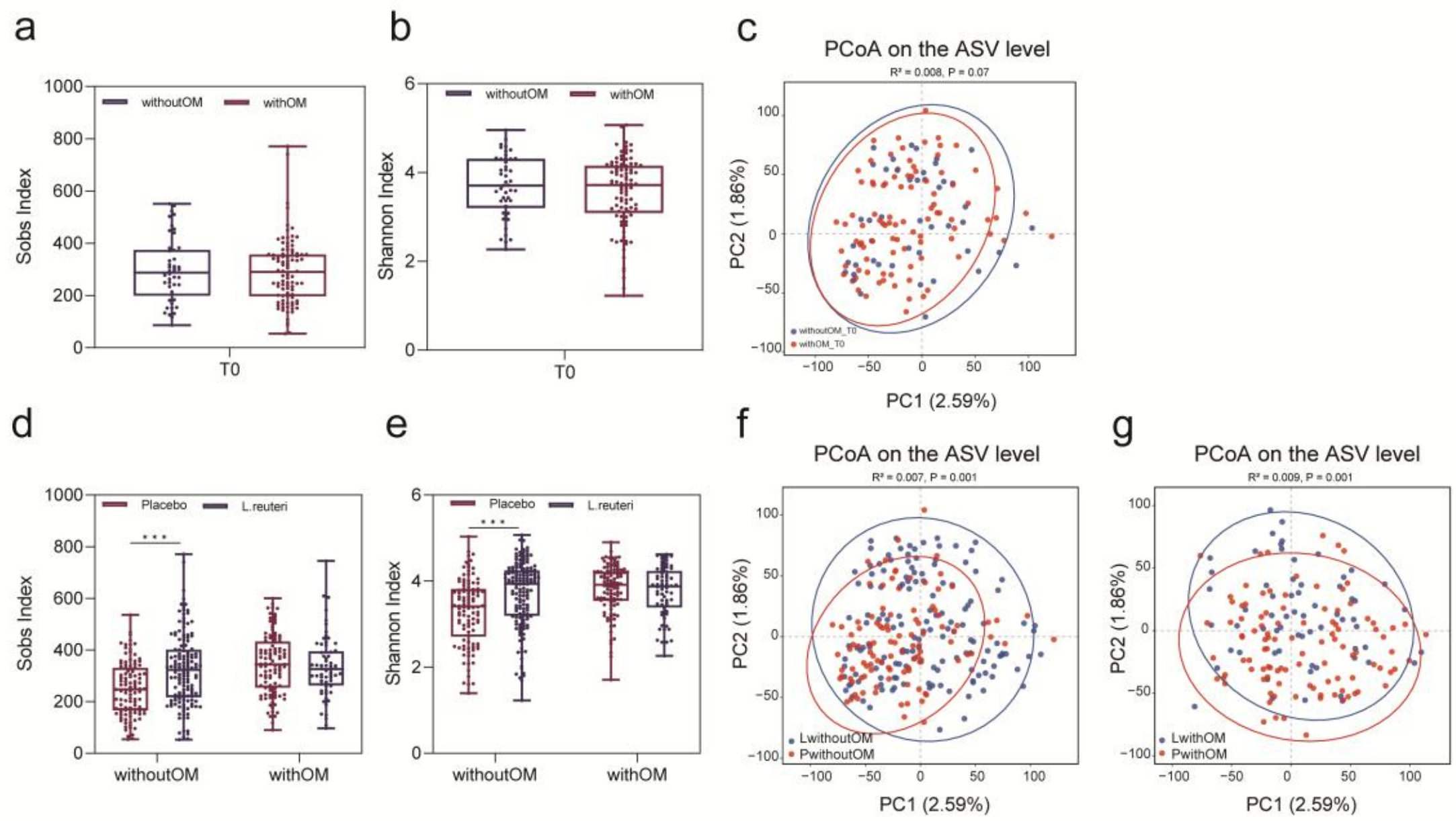

(**a-b**) Alpha diversity: Sobs and Shannon indices comparing salivary microbiota profiles stratified by oral mucositis (OM) status at baseline. (**c**) Beta diversity: Principal Coordinates Analysis (PCoA) plots based on Aitchison distance at the ASV level illustrating salivary microbiota profiles at T0, stratified by OM status. Statistical significance was assessed by PERMANOVA (R^2 and *P* values are indicated). (**d-e**) Alpha diversity: Sobs and Shannon indices comparing the *L. reuteri* and placebo groups, stratified by OM status. Boxplots depict the median, interquartile range, and range. (**f-g**) Beta diversity: PCoA plots based on Aitchison distance at the ASV level illustrating beta diversity differences between the *L. reuteri* and placebo groups, stratified by OM status. (**f**) Patients without OM; (**g**) Patients with OM. Abbreviations: OM, oral mucositis; SOM, severe oral mucositis; withOM, patients with oral mucositis;

withoutOM, patients without oral mucositis; withSOM, patients with severe oral mucositis; withoutSOM, patients without severe oral mucositis; ASV, amplicon sequence variant; PCoA, principal coordinates analysis.

Page 17 in the revised Supplementary information:

Supplementary Fig. 12. Salivary microbiota profiles stratified by OM status in the overall population.

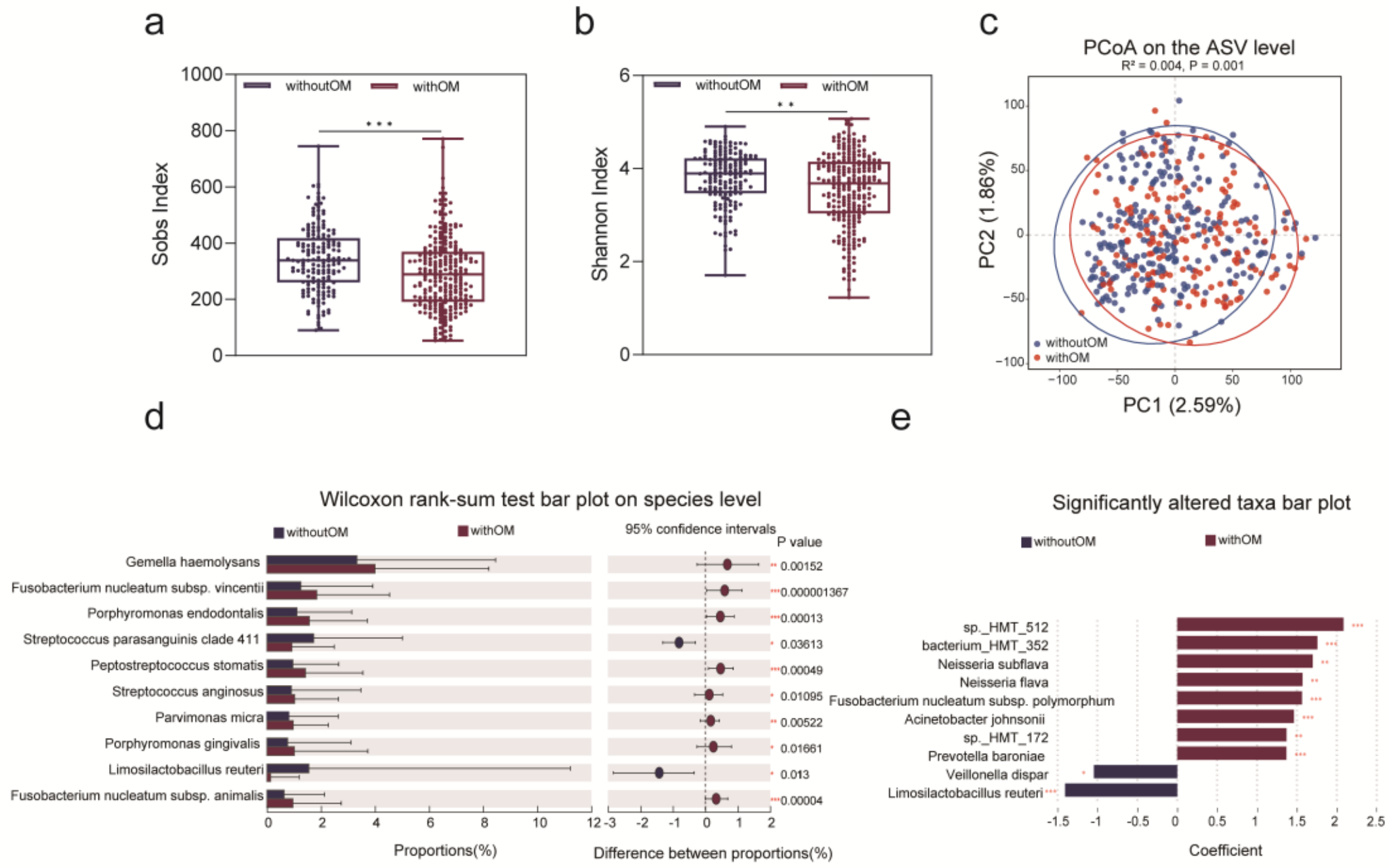

(**a-b**) Alpha diversity: Sobs and Shannon indices comparing patients with and without oral mucositis (OM). Boxplots depict the median, interquartile range, and range. (**c**) Beta diversity: Principal Coordinates Analysis (PCoA) plots based on Aitchison distance at the ASV level illustrating beta diversity between patients with and without OM. Statistical significance was assessed by PERMANOVA (R^2 and *P* values are indicated). (**d**) Differential taxon abundance: Mean relative abundances of the top differential taxa between patients with and without OM, as determined by the Kruskal-Wallis test. (**e**) Differential taxon abundance: Bacterial taxa significantly altered between patients with and without OM, identified by MaAsLin2 analysis. Abbreviations: OM, oral mucositis; SOM, severe oral mucositis; withOM, patients with oral mucositis; withoutOM, patients without oral mucositis; withSOM, patients with severe oral mucositis; withoutSOM, patients without severe oral mucositis; ASV, amplicon sequence variant; PCoA, principal coordinates analysis.

Comment 8: *Oral health status (caries, gingivitis, periodontitis, number of teeth, etc.) and baseline salivary rates not reported in Table 1.*

Response to Comment 8:

We sincerely thank you for raising this important point. We have now added the baseline salivary rates data to Table 1. The baseline salivary flow rates were comparable between the two groups, with a mean of 0.79 mL/min (SD = 0.41) in the *L. reuteri* group and 0.76 mL/min (SD = 0.33) in the placebo group ($P = 0.593$). Regarding oral health status, patients with severe periodontitis were excluded from our study per the eligibility criteria, and all participants underwent systematic oral examinations prior to enrollment to ensure relatively consistent baseline oral health conditions. Therefore, detailed baseline oral health parameters such as caries, gingivitis, and periodontitis were not specifically collected.

Page 31 in the revised manuscript:

TABLE 1 Baseline patient characteristics

Characteristic	No. (%) of Patients		
	L. reuteri (n = 80)	Placebo (n = 80)	All Patients (N = 160)
Sex, No. (%)			
Male	68 (85.0)	61 (76.2)	129 (80.6)
Female	12 (15.0)	19 (23.8)	31 (19.4)
Age, years, mean ± SD	56.3 ± 11.3	57.4 ± 12.4	56.8 ± 11.8
BMI, kg/m^2, mean ± SD	22.9 ± 3.6	22.6 ± 2.4	22.8 ± 3.0
ECOG PS, No. (%)			
0	54 (67.5)	53 (66.2)	107 (66.9)
1	24 (30.0)	25 (31.2)	49 (30.6)
2	2 (2.5)	2 (2.5)	4 (2.5)
Tumor pathologic types, No. (%)			
Squamous carcinoma	67 (83.8)	73 (91.2)	140 (87.5)
Others	13 (16.2)	7 (8.8)	20 (12.5)
Tumor site, No. (%)			
Nasopharynx	24 (30.0)	24 (30.0)	48 (30.0)
Oral cavity	22 (27.5)	22 (27.5)	44 (27.5)
Oropharynx	13 (16.2)	13 (16.2)	26 (16.2)
Hypopharynx	10 (12.5)	10 (12.5)	20 (12.5)
Larynx	8 (10.0)	7 (8.8)	15 (9.4)

Others [a]	3 (3.8)	4 (5.0)	7 (4.4)
Treatment type, No. (%)			
Definitive	38 (47.5)	40 (50.0)	78 (48.8)
Postoperative treatment	42 (52.5)	40 (50.0)	82 (51.2)
Concurrent chemotherapy, No. (%)			
Yes	37 (46.2)	34 (42.5)	71 (44.4)
No	43 (53.8)	46 (57.5)	89 (55.6)
TNM stage [b], No. (%)			
I-II	18 (22.5)	16 (20.0)	34 (21.2)
III	23 (28.7)	22 (27.5)	45 (28.1)
IVa	25 (31.2)	29 (36.2)	54 (33.8)
IVb	14 (17.5)	13 (16.2)	27 (16.9)
Tumor primary site, No. (%)			
T1	6 (7.5)	9 (11.2)	15 (9.4)
T2	26 (32.5)	26 (32.5)	52 (32.5)
T3	23 (28.7)	24 (30.0)	47 (29.4)
T4	25 (31.2)	21 (26.2)	46 (28.7)
Nodal involvement, No. (%)			
N0	28 (35.0)	25 (31.2)	53 (33.1)
N1	14 (17.5)	15 (18.8)	29 (18.1)
N2	26 (32.5)	24 (30.0)	50 (31.2)
N3	12 (15.0)	16 (20.0)	28 (17.5)
Tobacco use, No. (%)			
Yes	38 (47.5)	43 (53.8)	81 (50.6)
No	42 (52.5)	37 (46.2)	79 (49.4)
Alcohol use, No. (%)			
Yes	43 (53.8)	35 (43.8)	78 (48.8)
No	37 (46.2)	45 (56.2)	82 (51.2)
Oropharyngeal cancer HPV status, No. (%)			
Positive	5 (38.5)	3 (23.1)	8 (30.8)
Negative	8 (61.5)	10 (76.9)	18 (69.2)
Nasopharynx cancer EBV status, No. (%)			
Positive	24 (100.0)	24 (100.0)	48 (100.0)
Negative	0 (0.0)	0 (0.0)	0 (0.0)
Total radiotherapy dose, Gy, median (IQR)	66.0 (7.0)	66.0 (6.4)	66.0 (7.0)
Dose to oral cavity, Gy, median (IQR)	40.3 (13.9)	40.1 (10.9)	40.3 (13.3)
Baseline salivary rates, ml/min, mean ± SD	0.8 ± 0.4	0.8 ± 0.3	0.8 ± 0.4

Reviewer #5

Comment 1: *We appreciate your important work investigating a probiotic intervention for a debilitating side effect of radiotherapy. Please find below our major and specific concerns aimed at strengthening your manuscript.*

Response to Comment 1:

We sincerely thank you for the careful review of our manuscript and for the highly constructive comments, especially those concerning the statistical and methodological aspects of our study. These valuable suggestions have greatly improved the scientific rigor and overall quality of the manuscript. We have addressed each of the raised concerns point-by-point below and have revised the manuscript accordingly.

Comment 2: *The CONSORT diagram indicates that the Protocol and SAP were submitted as supplementary files. However, only the Protocol was found, and its statistical analysis section is quite vague. Please provide the SAP. The authors should provide a detailed description of all analytical methods used in the manuscript or Supplementary information, including but not limited to: how continuous and categorical data were described, what regression model was used for the primary outcome, and how relative risks (RR) were calculated.*

Response to Comment 2:

Thank you very much for highlighting this important point. We have now uploaded the formal SAP as a separate supplementary file. Furthermore, we have significantly expanded the "Statistical analysis" section in the revised Supplementary information to ensure full transparency and reproducibility. Accordingly, the specific revisions made in the Supplementary information are as follows:

Page 3 in the revised Supplementary information:

“Unadjusted primary analysis

The incidence of SOM was compared using the χ^2 test between the *L. reuteri* and placebo groups. The treatment effect was expressed as the relative risk (RR) with its 95% confidence interval (CI), calculated using the Katz logarithmic method. The time to the onset of SOM and OM was analyzed using the Kaplan-Meier method, with differences between groups compared via log-rank tests. Hazard ratios (HRs) and associated 95% CIs were estimated using a Cox proportional hazards model. The proportional hazards assumption was assessed using Schoenfeld residuals. Patients who did not develop SOM/OM during the study period were censored at the date of their last clinical assessment. The duration of SOM was calculated as the number of days from the first occurrence of SOM to the first instance of non-SOM, without subsequent SOM. Comparisons of duration were performed using two-part model (incorporating both the probability of occurrence and the conditional duration).

RR is defined as:

$$RR = \frac{p_1}{p_2} = \frac{a/(a+b)}{c/(c+d)}$$

Where p_1 and p_2 are the incidence proportions in the *L. reuteri* and placebo groups, respectively, and a, b, c, d are the cell counts from the 2×2 contingency table (with a and c representing the number of events in each group).

Adjusted multivariable models (Model 1)

To further evaluate the efficacy of *L. reuteri* while accounting for clinical heterogeneity, we employed multivariable regression models that adjusted for key

baseline covariates (Model 1): oral cavity radiation dose (continuous), primary tumor site (categorical), and receipt of concurrent chemotherapy (categorical).[9] The incidence and onset time of SOM/OM were analyzed using multivariable Cox regression, with the placebo group as the reference category. The duration of SOM/OM was analyzed using a two-part approach. The first part utilized multivariable logistic regression to model the probability of SOM/OM occurrence. In the second part, among patients who developed SOM/OM, the positive duration was modeled using a Gamma generalized linear model (GLM) with a log link. Adjusted marginal mean durations and between-group differences were derived by combining the predicted probabilities from the logistic component with the conditional mean estimates from the Gamma component. Results for the ITT population were presented in Table 2, while those for the PPS were detailed in Supplementary Table 5.

Adjusted multivariable models (Model 2)

To ensure the robustness of our findings, we conducted further adjusted analyses (Model 2). For the ITT population, Model 2 was expanded to include smoking and alcohol consumption history ("Yes" versus "No") as additional covariates in both the Cox and two-part models. For these binary variables, duration was expressed in the two-part models as the ratio of mean durations between exposure groups. For the PPS analysis, Model 2 further incorporated baseline microbial diversity (Shannon index), alongside smoking and alcohol history, to evaluate the intervention effect while controlling for baseline compositional differences in the microbiome. For the Shannon

diversity index in duration outcome analyses, effect estimates were reported as average marginal effects (AMEs) owing to its continuous nature, representing the mean change in duration per one-unit increase in the index, with all other covariates held constant. Results for the ITT population were presented in Table 2, while those for the PPS were detailed in Supplementary Table 5.

Subgroup analysis

We have added a forest plot to summarize subgroup analyses of the primary outcome (incidence of SOM) across key clinical subgroups, including tumor site, oral cavity radiotherapy dose, radiotherapy intent (definitive vs. adjuvant), use of concurrent chemotherapy, smoking history, and alcohol history. Risk ratios were estimated using modified Poisson regression with robust standard errors. For HPV status, due to the small sample size and potential for quasi-complete separation, Firth's penalized likelihood logistic regression was utilized to estimate the effect of HPV status on the primary outcome (incidence of SOM). Additionally, Fisher's exact test was used to compare the incidence of the endpoint between HPV-positive and HPV-negative groups.

To analyze longitudinal changes in questionnaire scores across follow-up time points, a mixed-effects model was employed. This model included fixed effects (treatment group, time, and group × time interaction) and random effects (subject), accounting for the correlation structure of repeated measurements. The model estimated predicted group means, mean differences, and corresponding P values.

Continuous variables following a normal distribution are presented as mean ± SD,

while those with a non-normal distribution are presented as median (IQR). Categorical variables were presented as frequencies and percentages. Continuous variables were analyzed using the T-test (parametric) or Wilcoxon rank-sum test (non-parametric), while categorical variables were compared with χ^2 or Fisher's exact tests, with statistical significance set at $P < .05$. Statistical analyses were performed using R software, version 4.4.0 (RStudio, PBC, Boston, MA, USA)."

Comment 3: *The Supplementary information mention the use of the Cox proportional hazards model to calculate HR and 95% CI without verifying the proportional hazards assumption. Please supplement the methods section with a description of how this assumption was evaluated.*

Response to Comment 3:

We sincerely thank you for this important methodological comment. We have tested the proportional hazards assumption for Cox regression models using the Schoenfeld residuals test. The results showed a violation of the proportional hazards assumption for both the time to onset of SOM and OM ($P < 0.05$). The reason for this finding may be the acute nature of SOM/OM, which typically occur during radiotherapy, and in the later follow-up, the event hazard became similarly low in both groups.

To address this, we performed a piecewise Cox regression analysis, using a cut-off of 36 days for SOM and 30 days for OM in the ITT population. The results showed that in the early stage (<36 days for SOM; <30 days for OM), the *L. reuteri* group had a

significantly lower hazard of developing the SOM/OM than the placebo group, whereas no significant difference was observed in the late stage. These results suggest that *L. reuteri* significantly delays the onset of SOM/OM. The results of the piecewise Cox regression analysis are presented in the table below.

Table for reviewers:

Results of the piecewise Cox regression analysis in the ITT population.

		HR (95% CI)	*P*
SOM onset	<36 days	0.37 (0.23-0.59)	<0.001
	⩾36 days	2.96 (0.34-25.44)	0.323
OM onset	<30 days	0.40 (0.27-0.58)	<0.001
	⩾30 days	1.00 (0.10-10.33)	1.000

Comment 4: *Duration endpoints are not handled appropriately. In the main analysis, patients who never developed SOM were assigned duration = 0 days, which can be seen in Supplementary statistical methods. This approach creates a heavily zero-inflated distribution, particularly in the intervention group where the incidence fell below 50%, rendering the reported median of 0.0 days a mathematical artifact rather than a true reflection of clinical recovery speed. Furthermore, treating "non-events" as "instantaneous events" within a Kaplan-Meier or Cox proportional hazards framework is statistically inappropriate as it violates the underlying assumptions of survival analysis. The Hurdle two-part model described in the Supplementary statistical methods (used for Supplementary Table 4) is suggested to be the main analysis.*

Response to Comment 4:

We sincerely thank you for raising this important point. We have revised the

statistical analysis method for the duration endpoint accordingly. Specifically, we now employ a two-part (Hurdle) model as the primary analytical approach for SOM/OM duration. The results showed that the duration of SOM/OM in the *L. reuteri* group remains significantly shorter than that in the placebo group, with the difference being statistically significant.

Accordingly, the specific revisions made in the manuscript and Supplementary information are as follows:

Page 3 in the revised Supplementary information:

"Unadjusted primary analysis

The incidence of SOM was compared using the chi-square test between the *L. reuteri* and placebo groups. The treatment effect was expressed as the relative risk (RR) with its 95% confidence interval (CI), calculated using the Katz logarithmic method. The time to the onset of SOM and OM was analyzed using the Kaplan-Meier method, with differences between groups compared via log-rank tests. Hazard ratios (HRs) and associated 95% CIs were estimated using a Cox proportional hazards model. The proportional hazards assumption was assessed using Schoenfeld residuals. Patients who did not develop SOM/OM during the study period were censored at the date of their last clinical assessment. The duration of SOM was calculated as the number of days from the first occurrence of SOM to the first instance of non-SOM, without subsequent SOM. Comparisons of duration were performed using two-part model (incorporating both the probability of occurrence and the conditional duration)."

Page 33, Line 699 in the revised manuscript:

TABLE 2 Efficacy results

Parameter	*L. reuteri* (n = 80)	Placebo (n = 80)	Effect size (95% CI)	*P*
Unadjusted primary analysis				
SOM incidence, %	37.5	61.3	0.61 (0.44-0.85)	.004
SOM onset, days, median	NE	16.0	0.43 (0.27-0.67)	<.001
SOM duration, days, median/mean (SD)	0.0/6.4 (9.6)	14.0/14.9 (14.5)	0.43 (0.21-0.66)	<.001
OM incidence, %	56.3	78.8	0.71 (0.57-0.89)	.004
OM onset, days, median	14.0	8.0	0.41 (0.28-0.60)	<.001
OM duration, days, median	14.0	35.0	0.56 (0.39-0.74)	.003
Model 1				
SOM incidence, %	37.5	61.3	0.44 (0.28-0.70)	<.001
SOM onset, days, median	NE	16.0	0.44 (0.28-0.70)	<.001
SOM duration, days, median/mean (SD)	0.0/6.4 (9.6)	14.0/14.9 (14.5)	0.46 (0.25-0.70)	.01
OM incidence, %	56.3	78.8	0.39 (0.26-0.58)	<.001
OM onset, days, median	14.0	8.0	0.39 (0.26-0.58)	<.001
OM duration, days, median	14.0	35.0	0.56 (0.40-0.74)	.004
Model 2				
SOM incidence, %	37.5	61.3	0.44 (0.28-0.69)	<.001
SOM onset, days, median	NE	16.0	0.44 (0.28-0.69)	<.001
SOM duration, days, median/mean (SD)	0.0/6.4 (9.6)	14.0/14.9 (14.5)	0.48 (0.26-0.71)	.02
OM incidence, %	56.3	78.8	0.36 (0.25-0.54)	<.001
OM onset, days, median	14.0	8.0	0.36 (0.25-0.54)	<.001
OM duration, days, median	14.0	35.0	0.57 (0.41-0.75)	.007

Abbreviations: Unadjusted incidence comparisons used χ^2 test (RR, 95% CI); onset time: log-rank test (HR, 95% CI); duration: two-part model (MR, 95%CI). Model 1 adjusted for tumor site, oral cavity radiotherapy dose and chemotherapy; Model 2 extended Model 1 by additionally adjusting for smoking history and alcohol consumption history. Cox regression was used for incidence/onset in adjusted models; two-part model for duration. *L. reuteri*, *Limosilactobacillus reuteri*; OM, oral mucositis; SOM, severe OM; SD, standard deviation; RR: relative risk; HR: hazard ratio; MR: mean ratio; CI: confidence interval; NE, not estimable.

Comment 5: *Line 355: The manuscript states that analyses were performed only on*

complete cases. It is recommended that the authors clearly state the hypothesized the missing data mechanism, evaluate it and consider imputation for sensitivity analysis where appropriate. Moreover, this description appears inconsistent with the Protocol, which mentions an imputation strategy for missing OM data. Please explain this discrepancy and report the missing data in the baseline table.

Response to Comment 5:

We sincerely thank you for this important comment. We would like to clarify how missing data were addressed for the different types of endpoints. For all endpoints, analyses were restricted to participants with complete data, and no imputation was performed. Specifically, for patients who withdrew from the intervention, we continued to collect data on OM-related endpoints to best reflect the clinical course, whereas no further data collection was performed for other endpoints. The Protocol mentioned an imputation strategy for missing OM data as a precautionary measure in anticipation of potential loss to follow-up or missed assessments. However, given the completeness of the data, this imputation plan was not used in the actual analysis. For the exploratory outcomes (e.g., quality of life, xerostomia, gustatory function), we conducted analyses on the PPS as prespecified in the Protocol, which included only participants who completed the intervention without major protocol deviations. Given that these outcomes were exploratory endpoints, imputation was not performed. We have provided a more detailed explanation of this in the revised manuscript.

Accordingly, the specific revisions made in the manuscript are as follows:

Page 20, Line 436 in the revised manuscript:

"For all end points, analyses were restricted to cases with complete data without imputation. Specifically, for patients who withdrew from the intervention, we continued to collect data on OM-related endpoints to best reflect the clinical course, whereas no further data collection was performed for other endpoints."

Comment 6: *Line 112 and Supplementary Table 4: In the adjusted analysis for the primary outcome, why does the title of S Table 4 state the ITT population, while the actual numbers for the treatment and control are 71 and 70 (which appear to be the PP set)? Similarly, Supplementary Table 5 shows the results for PP set but the first two column has identical results to Supplementary Table 4 that analyzed the ITT set. Please clarify.*

Response to Comment 6:

We sincerely apologize for this oversight and thank you for pointing out this discrepancy. The inconsistency was due to our negligence in failing to revise the first two columns of Table 4. The title, statistical interpretations of Table 4, and the entire content of Table 5 were correct. We have now carefully revised these tables to ensure full consistency and accuracy.

Accordingly, the specific revisions made in the manuscript and Supplementary information are as follows:

Page 33, Line 700 in the revised manuscript:

TABLE 2 Efficacy results

Parameter	*L. reuteri* (n = 80)	Placebo (n = 80)	Effect size (95% CI)	*P*
Unadjusted primary analysis				
SOM incidence, %	37.5	61.3	0.61 (0.44-0.85)	.004
SOM onset, days, median	NE	16.0	0.43 (0.27-0.67)	<.001
SOM duration, days, median/mean (SD)	0.0/6.4 (9.6)	14.0/14.9 (14.5)	0.43 (0.21-0.66)	<.001
OM incidence, %	56.3	78.8	0.71 (0.57-0.89)	.004
OM onset, days, median	14.0	8.0	0.41 (0.28-0.60)	<.001
OM duration, days, median	14.0	35.0	0.56 (0.39-0.74)	.003
Model 1				
SOM incidence, %	37.5	61.3	0.44 (0.28-0.70)	<.001
SOM onset, days, median	NE	16.0	0.44 (0.28-0.70)	<.001
SOM duration, days, median/mean (SD)	0.0/6.4 (9.6)	14.0/14.9 (14.5)	0.46 (0.25-0.70)	.01
OM incidence, %	56.3	78.8	0.39 (0.26-0.58)	<.001
OM onset, days, median	14.0	8.0	0.39 (0.26-0.58)	<.001
OM duration, days, median	14.0	35.0	0.56 (0.40-0.74)	.004
Model 2				
SOM incidence, %	37.5	61.3	0.44 (0.28-0.69)	<.001
SOM onset, days, median	NE	16.0	0.44 (0.28-0.69)	<.001
SOM duration, days, median/mean (SD)	0.0/6.4 (9.6)	14.0/14.9 (14.5)	0.48 (0.26-0.71)	.02
OM incidence, %	56.3	78.8	0.36 (0.25-0.54)	<.001
OM onset, days, median	14.0	8.0	0.36 (0.25-0.54)	<.001
OM duration, days, median	14.0	35.0	0.57 (0.41-0.75)	.007

Abbreviations: Unadjusted incidence comparisons used χ^2 test (RR, 95% CI); onset time: log-rank test (HR, 95% CI); duration: two-part model (MR, 95%CI). Model 1 adjusted for tumor site, oral cavity radiotherapy dose and chemotherapy; Model 2 extended Model 1 by additionally adjusting for smoking history and alcohol consumption history. Cox regression was used for incidence/onset in adjusted models; two-part model for duration. *L. reuteri*, *Limosilactobacillus reuteri*; OM, oral mucositis; SOM, severe OM; SD, standard deviation; RR: relative risk; HR: hazard ratio; MR: mean ratio; CI: confidence interval; NE, not estimable.

Page 22 in the revised Supplementary information:

Supplementary Table 5. Efficacy results in per-protocol set.

	L. reuteri (n=71)	Placebo (n=70)	Effect size (95% CI)	*P*

Unadjusted primary analysis				
SOM incidence, %	38.0	61.4	0.62 (0.44-0.88)	.009
SOM onset, days, median	28.0	20.0	0.42 (0.26-0.68)	<.001
SOM duration, days, median	0.0	15.5	0.44 (0.21-0.69)	.002
OM incidence, %	56.3	78.6	0.72 (0.56-0.91)	.008
OM onset, days, median	18.5	9.0	0.39 (0.26-0.59)	<.001
OM duration, days, median	14.0	35.0	0.57 (0.39-0.77)	.01
Radiotherapy treatment breaks ≥ five consecutive fractions, n (%)	1 (1.4)	1 (1.4)		
Model 1				
SOM incidence, %	38.0	61.4	0.44 (0.27-0.72)	<.001
SOM onset, days, median	28.0	20.0	0.44 (0.27-0.72)	<.001
SOM duration, days, median	0.0	15.5	0.49 (0.26-0.74)	.03
OM incidence, %	56.3	78.6	0.37 (0.24-0.56)	<.001
OM onset, days, median	18.5	9.0	0.37 (0.24-0.56)	<.001
OM duration, days, median	14.0	35.0	0.58 (0.40-0.77)	.02
Model 2				
SOM incidence, %	38.0	61.4	0.37 (0.22-0.64)	<.001
SOM onset, days, median	28.0	20.0	0.37 (0.22-0.64)	<.001
SOM duration, days, median	0.0	15.5	0.48 (0.25-0.74)	.06
OM incidence, %	56.3	78.6	0.24 (0.15-0.39)	<.001
OM onset, days, median	18.5	9.0	0.24 (0.15-0.39)	<.001
OM duration, days, median	14.0	35.0	0.57 (0.40-0.76)	.06

Abbreviations: Unadjusted incidence comparisons used χ^2 test (RR, 95% CI); onset time: log-rank test (HR, 95% CI); duration: two-part model (MR, 95% CI). Model 1 adjusted for tumor site, oral radiotherapy dose and chemotherapy; Model 2 extended Model 1 by additionally adjusting for smoking history, alcohol consumption history, and the Shannon index. Cox regression was used for incidence/onset in adjusted models; two-part model for duration. *L. reuteri, Limosilactobacillus reuteri*; OM, oral mucositis; SOM, severe OM; SD, standard deviation; RR: relative risk; HR: hazard ratio; MR: mean ratio; CI: confidence interval; NE, not estimable.

Comment 7: *Protocol Section 7.2: The authors state that OM was assessed twice per week during radiotherapy (at least 48 hours apart) and once per week for 8 weeks after radiotherapy. This raises concerns about true effect measurement:*

Potential underestimation of cumulative incidence if SOM occurs after the 8-week post-radiotherapy follow-up window and is treated as non-occurrence rather than right-

censoring.

Response to Comment 7:

Thank you very much for your valuable comment. We would like to clarify that SOM is typically an acute toxicity that occurs during radiotherapy, with the majority of cases occurring within the treatment period. In patients with head and neck cancer receiving conventional fractionated radiotherapy, SOM usually develops around 2-3 weeks after initiation, peaks during weeks 4-6, and gradually resolves within 2-4 weeks following completion of radiotherapy[22,23]. Published evidence and clinical guidelines consistently indicate that new-onset RIOM beyond the 8-week post-radiotherapy window is extremely rare. Accordingly, we selected an 8-week follow-up period after radiotherapy, which is consistent with previous prospective studies in this field.[24]

Comment 8: *Protocol Section 7.2: The authors state that OM was assessed twice per week during radiotherapy (at least 48 hours apart) and once per week for 8 weeks after radiotherapy. This raises concerns about true effect measurement:*

Bias in estimated onset and duration if OM episodes that both start and resolve between two scheduled visits are not captured, which may systematically shorten observed durations and exaggerate apparent treatment efficacy

Please clarify how interval censoring and the finite observation window were handled analytically.

Response to Comment 8:

We sincerely thank you for this insightful comment. We conducted follow-up visits twice weekly during radiotherapy and once weekly after radiotherapy to balance patient burden and adequate data capture. This schedule is consistent with the MASCC/ISOO guidelines[25], which recommend weekly or more frequent assessments for patients with head and neck cancer receiving (chemo)radiotherapy who are at high risk of severe mucositis, and is further supported by previously published prospective studies[24,26,27].

To ensure accurate measurement, we implemented multiple strategies: at each visit, patients were systematically asked whether their symptoms had worsened since the last assessment, enabling retrospective documentation of the onset time. Furthermore, all patients were encouraged to contact the blinded researchers immediately via WeChat if their oral symptoms deteriorated between visits, allowing for real-time evaluation. When possible, the onset date of SOM/OM was further verified by cross-referencing patients' recall during follow-up visits with the WeChat communication records. In addition, the World Health Organization grading scale, which combines objective mucosal findings with the patient's subjective assessment of oral intake, provides a more comprehensive and accurate reflection of the patient's true OM status.

In summary, the multiple measures described above were implemented to ensure accurate recording of the study endpoints in our research. We have provided further details in Supplementary information.

Accordingly, the specific revisions made in the Supplementary information are as follows:

Page 2 in the revised Supplementary information:

"At each scheduled visit, patients were systematically asked about any worsening of symptoms since the last assessment, so as to allow for retrospective documentation of the actual onset time. In addition, all patients were encouraged to contact the blinded investigators immediately via WeChat if their oral symptoms worsened between visits to facilitate real-time evaluation. The exact date of severe OM (SOM)/OM onset was further verified by cross-referencing patient recall during follow-up visits with the corresponding WeChat communication records where possible."

Comment 9: *Figure 4a and 4b present biodiversity and richness measured repeatedly across multiple time points. However, the statistical analysis described in Line 337 appears to rely on t-tests, which does not account for within-subject correlations. Please clarify whether methods appropriate for repeated measures (e.g., repeated measures ANOVA or mixed-effects models) were considered, and justify the choice of the current analytical approach.*

Response to Comment 9:

We sincerely thank you for raising this important point. We have re-analyzed the data using linear mixed-effects models with participant as a random intercept. We have updated the manuscript and the Methods section of Supplementary information.

Accordingly, the specific revisions made in the Supplementary information are as follows:

Page 3 in the revised Supplementary information:

"Linear mixed-effects models were used to assess the differences in α diversity indices (Shannon and Sobs) between the *L. reuteri* and placebo groups over time."

Comment 10: *Line 348: The description of the sample size calculation is unclear. It is not evident whether the assumed event rate in the intervention group was based on an absolute reduction of 40% (65%-25%) or a relative reduction of 25% from 65% (i.e., 48.75%). The authors are requested to clarify the exact assumptions used for the intervention group and to provide the detailed sample size calculation, including the formula and/or statistical code, in their response.*

Response to Comment 10:

We sincerely thank you for raising this important point. The intended event rate was an absolute reduction of 25 percentage points in the incidence of SOM, from an expected 65% in the placebo group to 40% in the *L. reuteri* group. We have now clarified this in the revised manuscript. The detailed steps and parameter settings are as follows:

To detect the specified difference between two independent proportions, a sample size calculation was performed using PASS 2021 (version 21.0.3). Assuming a two-sided significance level of α = 0.05, a statistical power of 80%, and equal group allocation, Fisher's exact test was used for the analysis. Based on these settings, a total of 71 patients per group was required. The detailed parameters and calculation setup

were illustrated in the figures below.

Accordingly, the specific revisions made in the manuscript are as follows:

Page 20, Line 424 in the revised manuscript:

"Based on previous studies and clinical practice, about 65% of head and neck cancer patients developed SOM during radiotherapy.[44] We anticipated that administration of *L. reuteri* would reduce SOM incidence by an absolute margin of 25% (from 65% to 40%). Accordingly, a sample size of 142 patients (71 per group) was required, calculated using PASS 2021 (version 21.0.3) with a two-sided α level of 0.05, 80% power, and 1:1 group allocation. After accounting for a 10% dropout rate and considerations for randomization balance, the final sample size was set at 160 patients (80 per group)."

Figure for reviewers: The process of calculating the sample size.

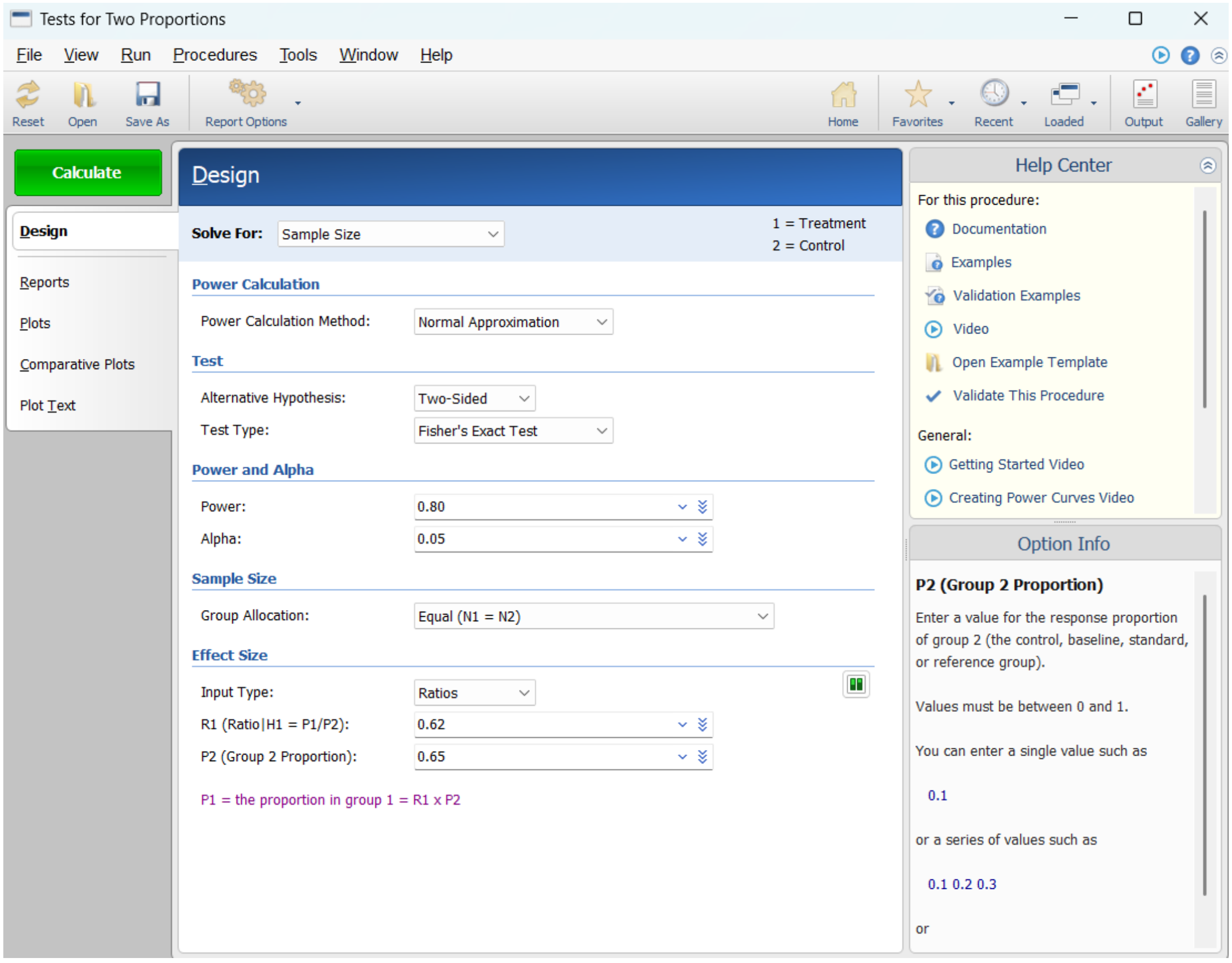

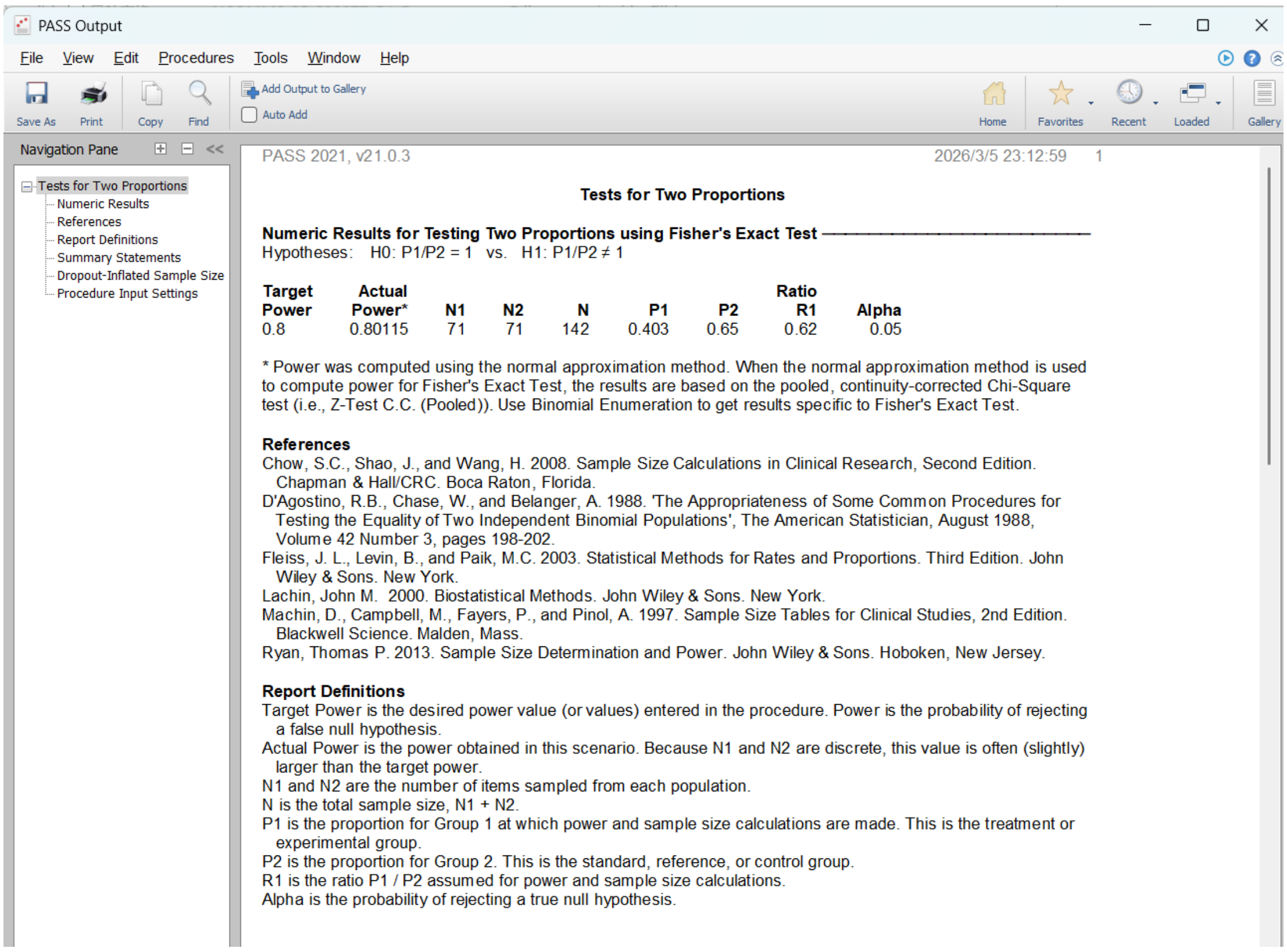

***Comment 11:** The protocol provided with the manuscript (version 1.0, dated 10*

November 2025) appears to have been finalized after the completion of the study (March 2024-January 2025). To ensure transparency and compliance with good clinical practice, the authors should submit the original protocol in place prior to study initiation, the final protocol version, and a detailed amendment history, including dates and justifications.

Response to Comment 11:

We sincerely thank you for raising this important point. The original protocol (version 1.0) was indeed finalized on 5 August 2023, prior to study initiation (March 2024). During manuscript preparation, we provided supplemental clarifications to the protocol. Specifically, we added detailed definitions of poor oral hygiene in the exclusion criteria and included a figure legend illustrating the follow-up schedule. Importantly, no changes were made to the study design, primary endpoint, sample size calculation, or statistical analysis plan. At that time, we mistakenly assumed that because these core methodological elements remained unchanged, the protocol version number could remain the same. Upon receiving your comment, we have now:

(1) Updated the protocol version to version 3.0 (dated 10 April 2026) to clearly reflect the supplemental clarifications.

(2) Prepared a detailed amendment history table documenting all changes have made, the corresponding dates, and justifications in the final protocol.

We have uploaded both versions of the protocol (original and final) along with the amendment history in the final protocol.

Comment 12: *Supplementary information: Why were other clinical parameters analyzed in the per-protocol (PP) population instead of the intention-to-treat (ITT) population (the primary analysis set)? Further clarification is needed.*

Response to Comment 12:

Thank you very much for this insightful question. For the primary and secondary endpoints, we strictly adhered to the ITT principle, as recommended by the CONSORT guidelines. For other clinical parameters, such as quality of life, oral and throat pain scores, these endpoints were exploratory in nature. Analyses of these exploratory endpoints were performed in the PP population, in which participants with major protocol deviations, missing data, or non-adherence during the intervention period were excluded. This analytical strategy was consistent with our study protocol.

Comment 13: *Since the authors mention in the Introduction that OM is a ubiquitous complication (with SOM occurring in 50%-70% of cases) and that MASCC/ISOO has proposed several interventions, why was the control group a placebo rather than an existing active treatment? Are there any recommended treatment measures in the current guidelines? It is suggested that the authors clarify in the Introduction the limitations of previous treatment options (if any).*

Response to Comment 13:

Thank you very much for your valuable suggestion. The MASCC/ISOO guidelines

recommend several interventions for OM; however, these recommendations are largely context-specific and lack universal applicability.[28] For instance, palifermin is the only agent approved by the FDA and EMA, but its indication is strictly limited to prophylactic use in patients with hematological malignancies undergoing hematopoietic stem cell transplantation.[29] Similarly, benzydamine mouthwash has Level I evidence for preventing OM, but only in head and neck cancer patients receiving moderate-dose radiotherapy (<50 Gy). Other commonly used agent, such as glutamine, antimicrobials, coating agents, and analgesics, are primarily supportive in nature and carry only Level II recommendations. This heterogeneity and the absence of a single, broadly accepted standard of care for OM support the need for placebo-controlled trials to establish efficacy. We have revised the Introduction section accordingly.

Regarding our study design, this was a Phase II trial aimed at exploring preliminary efficacy and safety. To ensure scientific rigor and minimize bias, the control group received a placebo in addition to standard oral care, which allowed for effective blinding of both investigators and participants, which is particularly important for the assessment of OM, consistent with most prior studies.[27,30,31] We agree that future Phase III studies should include active comparators, such as palifermin or other approved agents, to further validate these findings in head-to-head comparisons.

Accordingly, the specific revisions made in the manuscript are as follows:

Page 4, Line 68 in the revised manuscript:

"Currently, palifermin, a recombinant human keratinocyte growth factor, is the

only agent approved by the U.S. Food and Drug Administration (FDA) and the European Medicines Agency (EMA) for OM, but it is approved solely for OM prophylaxis in patients with hematological malignancies undergoing hematopoietic stem cell transplantation.[5] Among other interventions recommended by the Multinational Association of Supportive Care in Cancer/International Society of Oral Oncology (MASCC/ISOO), benzydamine mouthwash is supported by Level I evidence for preventing OM, but only in patients with head and neck cancer receiving moderate-dose radiotherapy (<50 Gy).[6] Other agents, including oral glutamine, antimicrobials, coating agents, anesthetics, and analgesics, are primarily used for symptomatic support and are limited to Level II recommendations.[6] Thus, the development of novel management strategies for radiation-induced oral mucositis (RIOM) remains paramount."

Comment 14: *Line 306: The authors mention that patients self-reported daily medication intake. Have authors considered how to verify the issue of adherence, such as regularly counting the pills in the medication boxes? Because overestimating adherence could lead to bias in the PP set.*

Response to Comment 14:

We sincerely thank you for this valuable suggestion. To accurately record study compliance, we implemented multiple measures. At each weekly follow-up visit, patients exchanged their used medication boxes for new ones, allowing the research

team to visually inspect and confirm the number of lozenges actually consumed. In addition, patients were asked to maintain daily medication diaries to record their lozenge intake. These approaches enabled us to track adherence more accurately. We have reported the adherence outcomes for both the ITT and PP populations in the main manuscript, and provided adherence data in the table below.

Accordingly, the specific revisions made in the manuscript are as follows:

Page 6, Line 113 in the revised manuscript:

"The mean adherence rate was 94.3% in the *L. reuteri* group and 92.6% in the placebo group in the ITT population, and 96.4% and 95.0% in the PP population, respectively."

Table for reviewers: Adherence rate in the *L. reuteri* and placebo groups. Adherence rate was calculated as the ratio of the actual number of lozenges used to the prescribed number of lozenges during radiotherapy. Adherence rates were compared using the Wilcoxon rank-sum test.

Adherence rate (%), mean ± SD	*L. reuteri*	Placebo	*P*
Intention-to-Treat Set	94.3 ± 10.3	92.6 ± 11.5	0.063
Per Protocol Set	96.4 ± 3.57	95.0 ± 4.56	0.051

Comment 15: *In Figure 1, the N for PP set of the placebo group should be 70 instead of 71 based on the flow table.*

Response to Comment 15:

We sincerely thank you for this comment. We have corrected the N value for the PP set of the placebo group in Figure 1 from 71 to 70, consistent with the participant

flow table.

Accordingly, the specific revisions made in the Figure 1 are as follows:

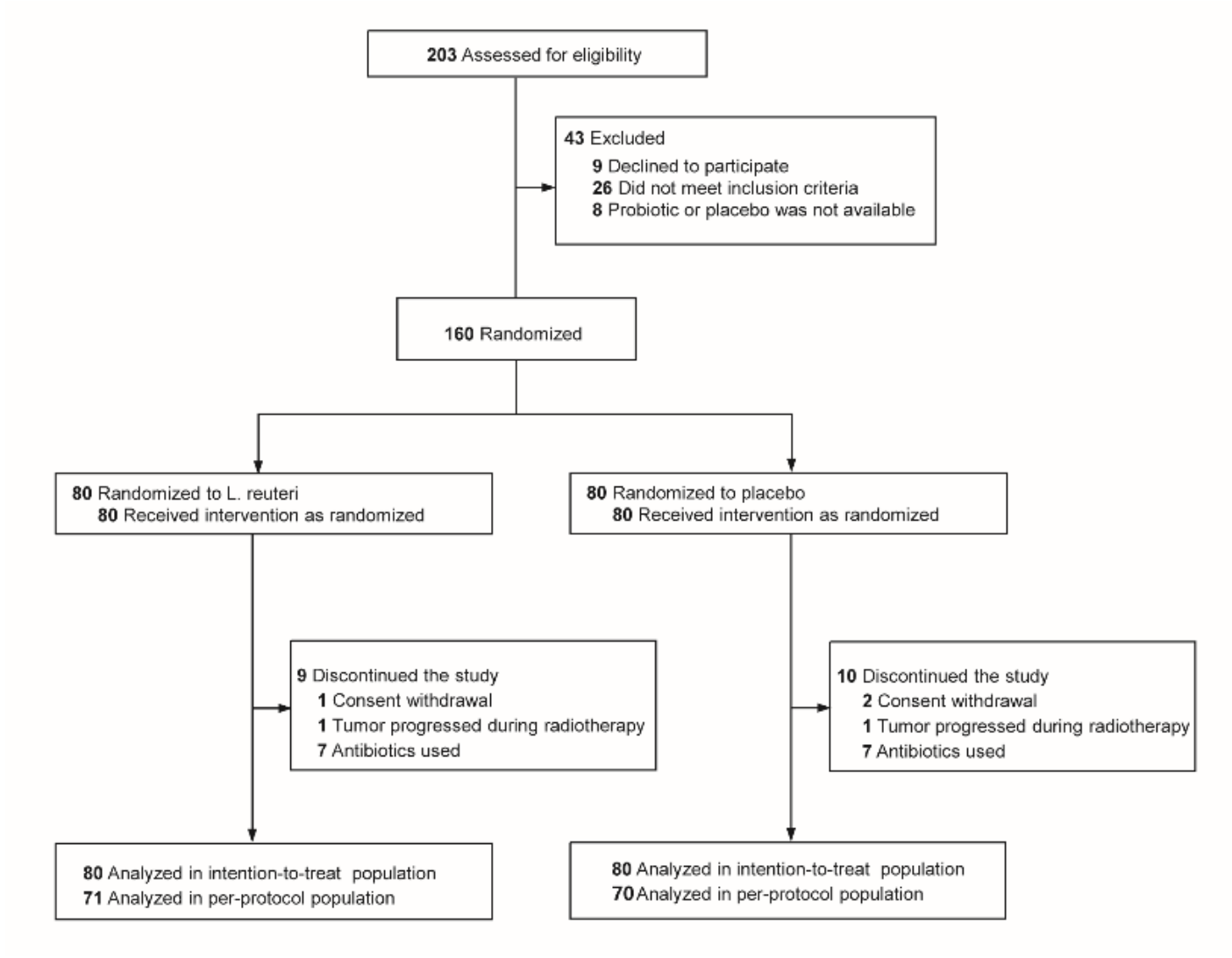

Comment 16: *Line 243: The authors state, "L. reuteri lozenges demonstrated significantly better patient acceptability, greater administration convenience, and more favorable cost-effectiveness profiles." As a placebo-controlled Phase II trial, direct comparison with other active treatments is not supported. The authors are advised to avoid extrapolating these findings beyond the study context.*

Response to Comment 16:

We sincerely thank you for this insightful comment. We fully agree that it is not appropriate to draw comparisons with other active treatments given that our study is a placebo-controlled Phase II trial. We have deleted the corresponding sentence from the revised manuscript.

Comment 17: *Figure 2 shows "No RT plan" and "Break time," but these labels are not*

interpretable without a clear legend embedded in the figure/caption. Also clarify explicitly why the swimmer plot uses n=45/80 vs 63/80 (appears to be restricted to participants who developed OM, but should be stated).

Response to Comment 17:

We sincerely thank you for these careful and helpful suggestions to improve the clarity of our manuscript. In response, we have now added a clear legend within the figure 2 to ensure that labels such as "No RT plan" and "Break time" are properly interpretable. Additionally, we have explicitly stated in the figure legend that the swimmer plot includes only participants who developed OM.

Accordingly, the specific revisions made in the figure legend are as follows:

Page 28, Line 648 in the revised manuscript:

"This figure displays only participants from both the *L. reuteri* group and the placebo group who developed OM. Each horizontal strip represents an individual patient. The top section indicates the timeline at or after radiotherapy, with a vertical grey line indicates the end of radiotherapy. The bottom strip highlights patients who experienced treatment breaks during radiotherapy (defined as ≥ five consecutive fractions). OM was assessed twice a week during radiotherapy and once a week for 8 weeks after radiotherapy. Red, WHO grade 4; yellow, WHO grade 3; purple, WHO grade 2; mauve, WHO grade 1; white, WHO grade 0; diagonal stripes, break time (Radiotherapy interruptions specifically attributable to SOM); X, no radiotherapy plan (The period at Week 7 with no scheduled radiotherapy, as patients had completed all

radiotherapy by Week 6); V, the visit for oral mucositis assessment; OM, oral mucositis; SOM, severe oral mucositis; *L. reuteri*, *Limosilactobacillus reuteri*."

Comment 18: *Supplementary Table 6 presents the same endpoints under two different N values (N=108 and N=95) without a clear definition of what differs between these sets. Please define inclusion criteria for each set.*

Response to Comment 18:

We sincerely thank you for raising this important point. We have now provided a clear definition of the inclusion criteria for each dataset in the revised Supplementary information. Specifically, N=108 represents solely those participants in the ITT population who developed OM, and N=95 represents solely those in the PP population who developed OM.

Accordingly, the specific revisions made in the Supplementary Table 6 are as follows:

Page 23 in the revised Supplementary information

Supplementary Table 6. Efficacy results in patients with oral mucositis.

Analysis Set	Endpoint	*L.reuteri*	Placebo	Effect size (95%CI)	*P*
Participants in the ITT population who developed OM (N=108)	**Unadjusted primary analysis**				
	SOM duration, days, median	15.5	21.0	0.69 (0.55-0.85)	<.001
	SOM onset, days, median	28.0	21.0	0.40 (0.25-0.64)	<.001
	OM duration, days, median	38.0	46.0	0.78 (0.65-0.92)	.003
	OM onset, days, median	20.0	9.0	0.46 (0.31-0.68)	<.001
	**Adjusted model**				
	SOM duration, days, median	15.5	21.0	0.76 (0.61-0.93)	0.012
	SOM onset, days, median	28.0	21.0	0.36 (0.21-0.60)	<.001
	OM duration, days, median	38.0	46.0	0.77 (0.64-0.91)	0.004
	OM onset, days, median	20.0	9.0	0.37 (0.24-0.57)	<.001
Participants in the PP Set who developed OM (N=95)	**Unadjusted primary analysis**				
	SOM duration, days, median	18.0	25.0	0.70 (0.56-0.87)	.002
	SOM onset, days, median	28.0	20.0	0.29 (0.17-0.52)	<.001
	OM duration, days, median	39.0	49.0	0.79 (0.66-0.95)	.013
	OM onset, days, median	18.5	9.0	0.40 (0.26-0.61)	<.001
	**Adjusted model**				
	SOM duration, days, median	18.0	25.0	0.78 (0.63-0.97)	0.031
	SOM onset, days, median	28.0	20.0	0.25 (0.14-0.46)	<.001
	OM duration, days, median	39.0	49.0	0.78 (0.64-0.95)	0.015
	OM onset, days, median	18.5	9.0	0.33 (0.21-0.53)	<.001

Abbreviations: Unadjusted onset time comparisons used log-rank test (HR, 95% CI); duration: two-part model (MR, 95%CI). Adjusted model adjusted for tumor site, oral radiotherapy dose and chemotherapy. Cox regression was used for onset in adjusted models; two-part model for duration. *L. reuteri*, *Limosilactobacillus reuteri*; OM, oral mucositis; SOM, severe OM; MR: mean ratio; HR: hazard ratio; CI: confidence interval.

Comment 19: *"Supplementary Table S1" should be "Supplementary Table 1" (Lines 314-315), and "Supplementary 1" appears to be an incomplete/incorrect reference (Line 329).*

Response to Comment 19:

We sincerely thank you for pointing out these formatting errors. The incomplete reference "Supplementary 1" in Line 329 refers to the study protocol. We have revised the incomplete reference "Supplementary 1" in Line 329 to "Protocol", and have corrected "Supplementary Table S1" to "Supplementary Table 1" in Lines 314-315.

Comment 20: *The process graph in the Protocol (page 10) incorrectly includes radiotherapy bars in the post-radiotherapy period and should be corrected.*

Response to Comment 20:

We sincerely thank you for your careful review. The error in the process graph on page 10 of the Protocol has been corrected in the revised version.

Accordingly, the specific revisions made in the process graph in the Protocol are as follows:

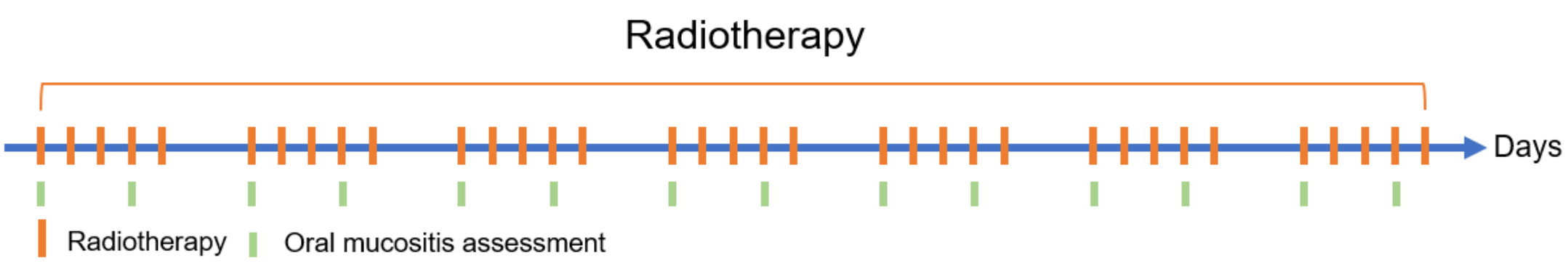

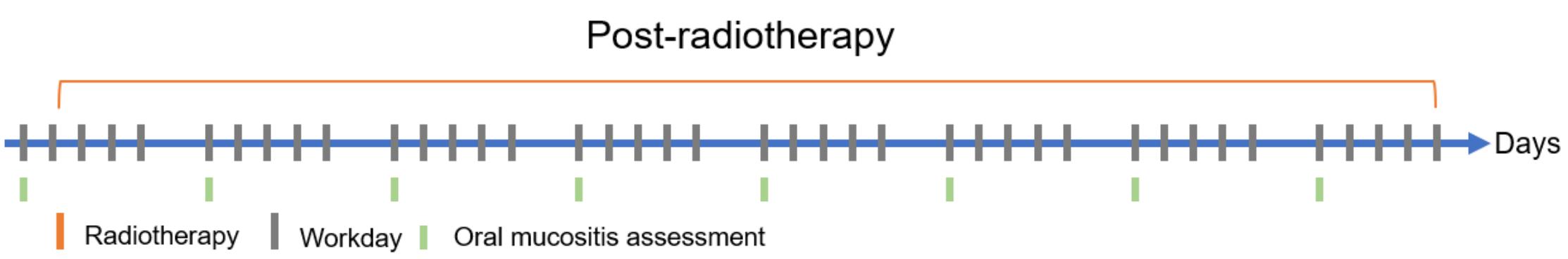

Reviewer #6

Comment: *I co-reviewed this manuscript with one of yous who provided the listed reports. This is part of the Nature Communications initiative to facilitate training in peer review and to provide appropriate recognition for Early Career Researchers who co-review manuscripts.*

Response to Comment:

We sincerely thank you for the careful and dedicated work in co-reviewing this manuscript. We greatly appreciate your valuable insights and constructive comments, which have been instrumental in improving the quality and rigor of our work.

We sincerely hope that the revised manuscript has addressed all editors' comments and now meets the standards of *Nature Communications*. We would like to express our sincere gratitude to the reviewers for their valuable time and constructive suggestions.

References:

1 Peng, X. *et al.* Streptococcus salivarius K12 Alleviates Oral Mucositis in Patients Undergoing Radiotherapy for Malignant Head and Neck Tumors: A Randomized Controlled Trial. *J Clin Oncol* **42**, 1426-1435 (2024). https://doi.org:10.1200/jco.23.00837

2 Luo, Z. *et al.* Limosilactobacillus reuteri in immunomodulation: molecular mechanisms and potential applications. *Front Immunol* **14**, 1228754 (2023). https://doi.org:10.3389/fimmu.2023.1228754

3 Wu, H. *et al.* Lactobacillus reuteri maintains intestinal epithelial regeneration and repairs damaged intestinal mucosa. *Gut Microbes* **11**, 997-1014 (2020). https://doi.org:10.1080/19490976.2020.1734423

4 Sharma, A. *et al.* Lactobacillus brevis CD2 lozenges reduce radiation- and chemotherapy-induced mucositis in patients with head and neck cancer: a randomized double-blind placebo-controlled study. *Eur J Cancer* **48**, 875-881 (2012). https://doi.org:10.1016/j.ejca.2011.06.010

5 Fan, X., Cui, H. & Liu, S. Summary of the best evidence for nutritional support programs in nasopharyngeal carcinoma patients undergoing radiotherapy. *Front Nutr* **11**, 1413117 (2024). https://doi.org:10.3389/fnut.2024.1413117

6 Arends, J. *et al.* ESPEN guidelines on nutrition in cancer patients. *Clin Nutr* **36**, 11-48 (2017). https://doi.org:10.1016/j.clnu.2016.07.015

7 Liu, F. *et al.* Trends, Characteristics, and Success Rates of Treatment for Severe Early Childhood Caries Under General Anesthesia: A Retrospective Study in Northwest China. *J Clin Pediatr Dent* **45**, 278-283 (2021). https://doi.org:10.17796/1053-4625-45.4.11

8 Cheng, L. *et al.* Expert consensus on dental caries management. *Int J Oral Sci* **14**, 17 (2022). https://doi.org:10.1038/s41368-022-00167-3

9 Bensadoun, R. J., Patton, L. L., Lalla, R. V. & Epstein, J. B. Oropharyngeal candidiasis in head and neck cancer patients treated with radiation: update 2011. *Support Care Cancer* **19**, 737-744 (2011). https://doi.org:10.1007/s00520-011-1154-4

10 Pinel, B., Cassou-Mounat, T. & Bensadoun, R. J. [Oropharyngeal candidiasis and radiotherapy]. *Cancer Radiother* **16**, 222-229 (2012). https://doi.org:10.1016/j.canrad.2011.11.004

11 Lu, Z. *et al.* Early Interdisciplinary Supportive Care in Patients With Previously Untreated Metastatic Esophagogastric Cancer: A Phase III Randomized Controlled Trial. *J Clin Oncol* **39**, 748-756 (2021). https://doi.org:10.1200/jco.20.01254

12 Navazesh, M. Methods for collecting saliva. *Ann N Y Acad Sci* **694**, 72-77 (1993). https://doi.org:10.1111/j.1749-6632.1993.tb18343.x

13 Villa, A., Connell, C. L. & Abati, S. Diagnosis and management of xerostomia and hyposalivation. *Ther Clin Risk Manag* **11**, 45-51 (2015). https://doi.org:10.2147/tcrm.S76282

14 Luo, J. *et al.* Smad7 Promotes Healing of Radiotherapy-Induced Oral Mucositis without Compromising Oral Cancer Therapy in a Xenograft Mouse Model. *Clin Cancer Res* **25**, 808-818 (2019). https://doi.org:10.1158/1078-0432.Ccr-18-1081

15 Han, G. *et al.* Preventive and therapeutic effects of Smad7 on radiation-induced oral

mucositis. *Nat Med* **19**, 421-428 (2013). https://doi.org:10.1038/nm.3118
16 Huang, Y. Current status of pain management in China: an overview. *Eur J Pain* **5 Suppl A**, 67-71 (2001). https://doi.org:10.1053/eujp.2001.0283
17 Xiao, H. *et al.* Pain Prevalence and Pain Management in a Chinese Hospital. *Med Sci Monit* **24**, 7809-7819 (2018). https://doi.org:10.12659/msm.912273
18 Xu, X., Luckett, T., Lovell, M. & Phillips, J. L. Cultural factors affecting Chinese migrants' perceptions and responses to cancer pain and its pharmacological management: A convergent mixed-method study. *Palliat Support Care* **21**, 688-696 (2023). https://doi.org:10.1017/s1478951522000360
19 Weissman, D. E., Gordon, D. & Bidar-Sielaff, S. Cultural aspects of pain management. *J Palliat Med* **7**, 715-716 (2004). https://doi.org:10.1089/jpm.2004.7.715
20 Chen, L. M., Miaskowski, C., Dodd, M. & Pantilat, S. Concepts within the Chinese culture that influence the cancer pain experience. *Cancer Nurs* **31**, 103-108 (2008). https://doi.org:10.1097/01.NCC.0000305702.07035.4d
21 Torozan, D. A. *et al.* Metagenomic Profiling of Oral Microbiome Dynamics During Chemoradiotherapy in Head and Neck Squamous Cell Carcinoma Patients. *Cancer Med* **14**, e70589 (2025). https://doi.org:10.1002/cam4.70589
22 Lalla, R. V. & Peterson, D. E. Oral mucositis. *Dent Clin North Am* **49**, 167-184, ix (2005). https://doi.org:10.1016/j.cden.2004.07.009
23 Sonis, S. T. A biological approach to mucositis. *J Support Oncol* **2**, 21-32; discussion 35-26 (2004).
24 Henke, M. *et al.* Palifermin decreases severe oral mucositis of patients undergoing postoperative radiochemotherapy for head and neck cancer: a randomized, placebo-controlled trial. *J Clin Oncol* **29**, 2815-2820 (2011). https://doi.org:10.1200/jco.2010.32.4103
25 Abdalla-Aslan, R. *et al.* Guidance on mucositis assessment from the MASCC Mucositis Study Group and ISOO: an international Delphi study. *EClinicalMedicine* **73**, 102675 (2024). https://doi.org:10.1016/j.eclinm.2024.102675
26 Liao, Z. *et al.* Bacterial Decolonization With Mupirocin Ointment for Acute Radiation Oral Mucositis Prevention: A Phase 3 Randomized Clinical Trial. *JAMA Oncol* **11**, 1141-1149 (2025). https://doi.org:10.1001/jamaoncol.2025.2361
27 Anderson, C. M. *et al.* Phase IIb, Randomized, Double-Blind Trial of GC4419 Versus Placebo to Reduce Severe Oral Mucositis Due to Concurrent Radiotherapy and Cisplatin For Head and Neck Cancer. *J Clin Oncol* **37**, 3256-3265 (2019). https://doi.org:10.1200/jco.19.01507
28 Elad, S. *et al.* MASCC/ISOO clinical practice guidelines for the management of mucositis secondary to cancer therapy. *Cancer* **126**, 4423-4431 (2020). https://doi.org:10.1002/cncr.33100
29 Sonis, S. T. Treatment for Oral Mucositis-Current Options and an Update of Small Molecules Under Development. *Curr Treat Options Oncol* **22**, 25 (2021). https://doi.org:10.1007/s11864-021-00823-6
30 Jiang, C. *et al.* A randomized, double-blind, placebo-controlled trial of probiotics to reduce

the severity of oral mucositis induced by chemoradiotherapy for patients with nasopharyngeal carcinoma. *Cancer* **125**, 1081-1090 (2019). https://doi.org:10.1002/cncr.31907

31 Le, Q. T. *et al.* Palifermin reduces severe mucositis in definitive chemoradiotherapy of locally advanced head and neck cancer: a randomized, placebo-controlled study. *J Clin Oncol* **29**, 2808-2814 (2011). https://doi.org:10.1200/jco.2010.32.4095

We sincerely appreciate the continued efforts of the reviewers in evaluating our manuscript. In response to the reviewers' comments, we have undertaken corresponding revisions to the manuscript. In brief, we have performed more in-depth analyses of the oral microbiota and updated statistical analyses where appropriate.

We believe that all reviewer comments have been addressed in the revised manuscript and in this response. We hope that the revisions now meet the standards for publication in ***Nature Communications***. All changes have been tracked using the "Track Changes" function in the manuscript. Below, we provide point-by-point responses to each comment. Please also find attached a detailed, point-by-point response to all reviewer comments and a marked version of the revised manuscript.

Point-by-point response to the reviewer comments on the manuscript "***Limosilactobacillus reuteri*** **for Radiation-Induced Oral Mucositis in Patients with Head and Neck Cancer: A Randomized Clinical Trial**".

Reviewer #3

Comment 1: *Dear Peng and colleagues - thank you for our thorough and considered rebuttal. It was great to see almost all of the requested edits implemented, and where necessary, clear justification for any concerns raised in R1. Thank you for your efforts.*

Response to Comment 1:

Thank you very much for your positive and encouraging comments. Your insightful suggestions have helped us greatly improve our manuscript. We truly appreciate your time and effort.

Comment 2: *When describing your study relative to the Ssk12 study (2023), I would make it explicitly clear that these were independent studies. When setting up the rationale as aiming to compare the efficacy of different strains, it may be misinterpreted as a head-to-head comparison. Please make it clear that all cohorts, including the placebo, were independent in the two studies to avoid confusion.*

Response to Comment 2:

We sincerely apologize for our incorrect use of the term "head-to-head comparison"

when describing the choice between *SsK12* and *L. reuteri*. We fully agree with you that the present trial is an independent study separate from our previously published *Ssk12* study. All participant cohorts in these two trials are entirely independent. When discussing the choice between *SsK12* and *L. reuteri*, each strain possesses its own distinct characteristics. Specifically, in addition to the competitive exclusion and direct inhibition of pathogenic microorganisms exhibited by *SsK12*, *L. reuteri* also actively modulates the host inflammatory microenvironment and maintains mucosal homeostasis.[1-4] We fully agree with you that combining the two strains might achieve greater efficacy, and we plan to conduct this research in future studies.

Comment 3: *The baseline microbiome differences between the placebo and intervention are nicely described now, thank you. Can you please clarify that you tested how you tested the association between baseline microbiome composition and future OM. What time point did you define mucositis (or was it just highest grade?). In this statement, is sounds like you looked at baseline microbiome and stratified by baseline OM severity. "Expanded analysis of oral microbiota at baseline: We performed additional analyses to explore whether baseline oral microbiota composition differed according to OM status. The results showed that no significant differences in α diversity or β diversity between patients with OM and patients without OM at baseline (Supplementary Fig. 11a-c).*

Response to Comment 3:

We sincerely thank you for this important and insightful comment. For the association analysis between baseline oral microbiome and future OM development: we compared baseline α diversity and β diversity between patients who developed OM during radiotherapy and those who never developed OM in the whole study period. This analysis was conducted to investigate whether baseline oral microbiome composition influences subsequent OM development. Regarding the definition of OM status for grouping: patients were classified as with OM if oral mucositis occurred at any time during the study, and as without OM if oral mucositis never developed throughout the study.

We sincerely apologize for the misunderstanding caused by inaccurate terminology usage, and we have now revised relevant descriptions and figure legends in the revised manuscript and Supplementary Information. We have also additionally performed differential abundance analysis of baseline taxa between samples from patients who subsequently developed OM and those who remained OM-free, to further identify candidate taxa predictive of subsequent OM development.

Accordingly, the specific revisions made in the Supplementary Information are as follows:

Page 16 in the revised Supplementary Information:

Supplementary Fig. 11. Salivary microbiota profiles stratified by subsequent OM development using baseline samples in the overall cohort.

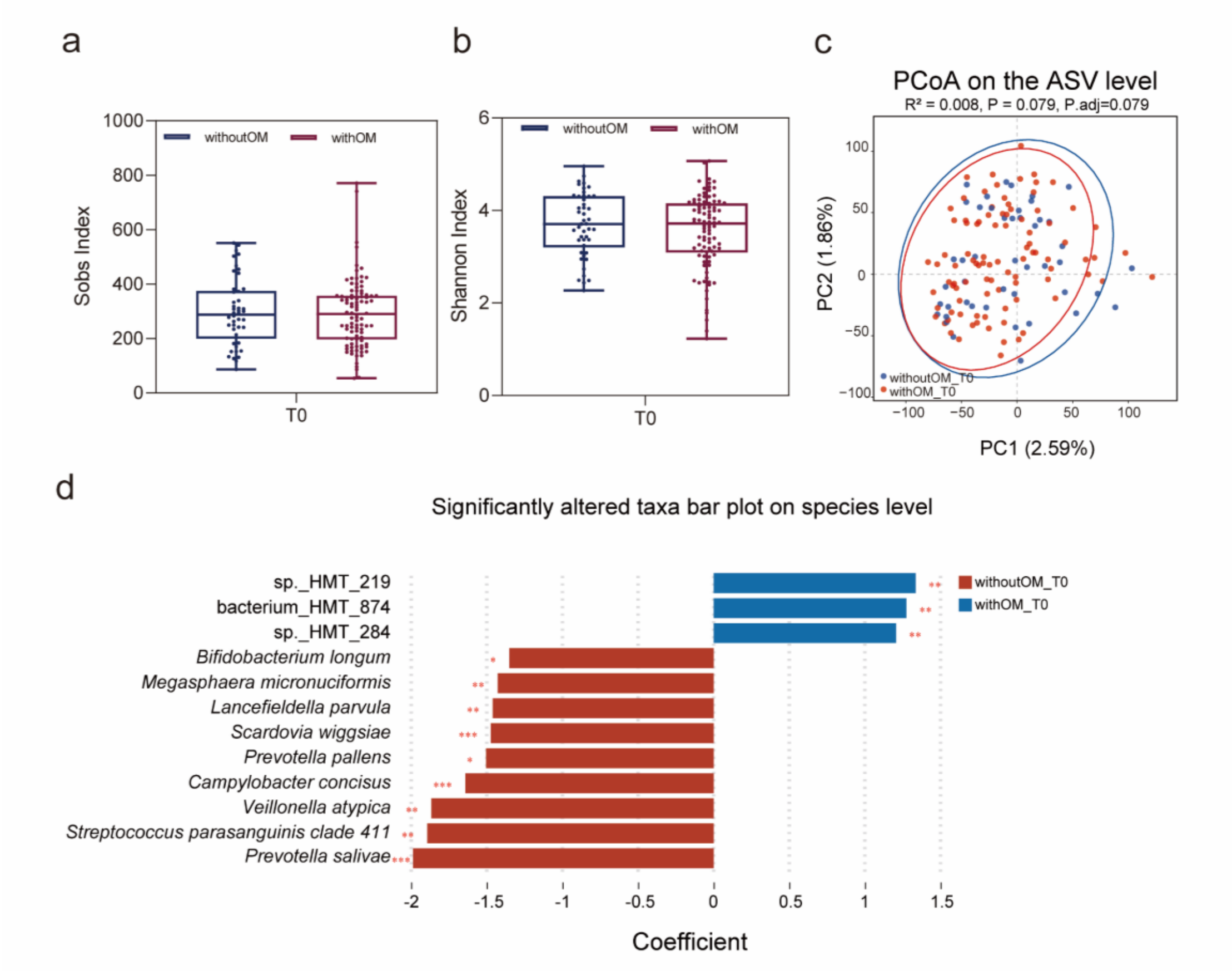

(**a-b**) α diversity: Sobs and Shannon indices comparing salivary microbiota profiles stratified by subsequently OM status at baseline. (**c**) β diversity: PCoA plots based on Aitchison distance at the ASV level illustrating salivary microbiota profiles at baseline, stratified by subsequently OM status. Statistical significance was assessed by PERMANOVA (R^2 and P values are indicated), with P values adjusted using the FDR correction. (**d**) Significantly differentially abundant bacterial taxa between baseline samples from patients with subsequent OM and those remaining OM-free, identified by MaAsLin2. P values were adjusted for multiple comparisons: * $P < 0.05$, ** $P < 0.01$, *** $P < 0.001$. Abbreviations: OM, oral mucositis; T0, baseline; withOM, patients with oral mucositis; withoutOM, patients without oral mucositis; withOM_T0, baseline samples from patients who subsequently developed OM; withoutOM_T0, baseline samples from patients who remained OM-free; ASV, amplicon sequence variant; PCoA, principal coordinates analysis; FDR, false discovery rate.

Comment 4: *It was fascinating to learn of the nuances of the Chinese culture with respect to stoicism, pain, adherence etc. I note that none of this went into the discussion of the manuscript. While I appreciate it doesn't require lengthy discussion, it may be of use to some readers who are familiar with these values.*

Response to Comment 4:

Thank you for this insightful comment. We have incorporated a discussion of this sociocultural nuance in the manuscript, noting that traditional stoic attitudes toward pain and greater symptom tolerance among Chinese individuals may contribute to lower analgesic uptake, helping contextualize our findings for international readers.

Accordingly, the specific revisions made in the manuscript are as follows:

Page 13, Line 258 in the revised manuscript:

"Notably, the relatively low analgesic use in our cohort may be partly attributed to traditional stoic attitudes toward pain among Chinese patients.[26-28]"

Reviewer #4

Comment 1: *The reviewers have made a commendable effort to address the concerns raised during the first round of review; however, the revisions are still not satisfactory:.*

Response to Comment 1:

We sincerely apologize that our first round of revision failed to fully meet your expectations. We have further refined the bioinformatic analysis and downstream microbiome data analysis in the second round of revision. Our point-by-point responses are provided below.

Comment 2: *While the authors performed species-level taxonomy assignment of the ASVs using blastn as recommended, the percent identity threshold applied (80%) is very low. For species-level classification, at least 98% is commonly used. Often higher*

coverage than 90% is also required for more reliable assignments. With the parameters employed, the species level calls are not reliable. It is also not clear how blastn was run and how ties were handled.

Response to Comment 2:

We sincerely thank you for this important methodological comment. We have revised the bioinformatic parameters for species-level classification by setting the percent identity threshold to 98% and the coverage threshold to 90% to ensure more reliable species-level assignments. Additionally, we have supplemented detailed methodological descriptions of the blastn running procedures and tie-handling strategies in the revised Supplementary Information to clarify the analytical workflow.

Accordingly, the specific revisions made in the Supplementary Information are as follows:

Page 3 in the revised Supplementary Information:

"Taxonomic assignment of ASVs was performed using BLASTn7 (version 2.12.0) against the Human Oral Microbiome Database (HOMD, version 15.22), with a minimum identity threshold of 98% and query coverage of 90% to classify at the species level. A blast-best-hit approach was adopted for annotation, with tie-breaking criteria applied in the following priority order: (1) smallest E-value (most significant), (2) highest percent identity if E-values were equal, (3) highest query coverage if both E-value and identity were equal, and (4) if still tied, the entry with the highest taxonomic rank and the most complete annotation information was selected."

Comment 3: *Since CLR-transformation is now applied to the data, there is no need to rarefy the ASV tables since this results in loss of data.*

Response to Comment 3:

Thank you very much for this valuable suggestion in refining our analytical pipeline. We have revised the bioinformatics analysis pipeline by removing the rarefaction step of ASV tables for β diversity and differential abundance analysis. We also have updated the Methods section accordingly and confirm that this change does not alter the main conclusions of our study but improves the robustness and compositional coherence of the results.

Accordingly, the specific revisions made in the Supplementary Information are as follows:

Page 3 in the revised Supplementary Information:

"The ASV table was rarefied to an even depth of 26,080 reads per sample for α diversity analysis. The original non-rarefied ASV table was used for β diversity analysis (Aitchison distance based on centered log-ratio transformation), Kruskal-Wallis comparative tests, and multivariate association analysis using MaAsLin2."

Comment 4: *The authors have switched to compositional data aware methods as recommended (CLR transformation, Aitchison distances, and MaAslin2, etc.) but they need to specify the threshold an method used for FDR and ensure that PERMANOVA results are also adjusted for pairwise comparisons where applicable.*

Response to Comment 4:

We sincerely thank you for this insightful comment. We have explicitly stated the multiple comparison correction method and threshold in the revised manuscript and Supplementary Information. Specifically, all differential analyses were performed using the Benjamini-Hochberg procedure to control the FDR, with a significance threshold set at adjusted $P < 0.05$. For the PERMANOVA analyses, we have incorporated FDR correction for pairwise P values and reported both raw and adjusted P values in the revised Supplementary Information.

Accordingly, the specific revisions made in the manuscript and Supplementary Information are as follows:

Page 21, Line 436 in the revised manuscript:

"P values from all analyses were adjusted for multiple testing using the Benjamini-Hochberg false discovery rate (FDR) method, with statistical significance defined as adjusted $P < 0.05$."

Page 3 in the revised Supplementary Information:

"P values from all analyses were adjusted for multiple testing using the Benjamini-Hochberg false discovery rate (FDR) method, with statistical significance defined as adjusted $P < 0.05$."

Page 14 in the revised Supplementary Information:

Supplementary Fig. 9. The comparison of principal coordinate analysis.

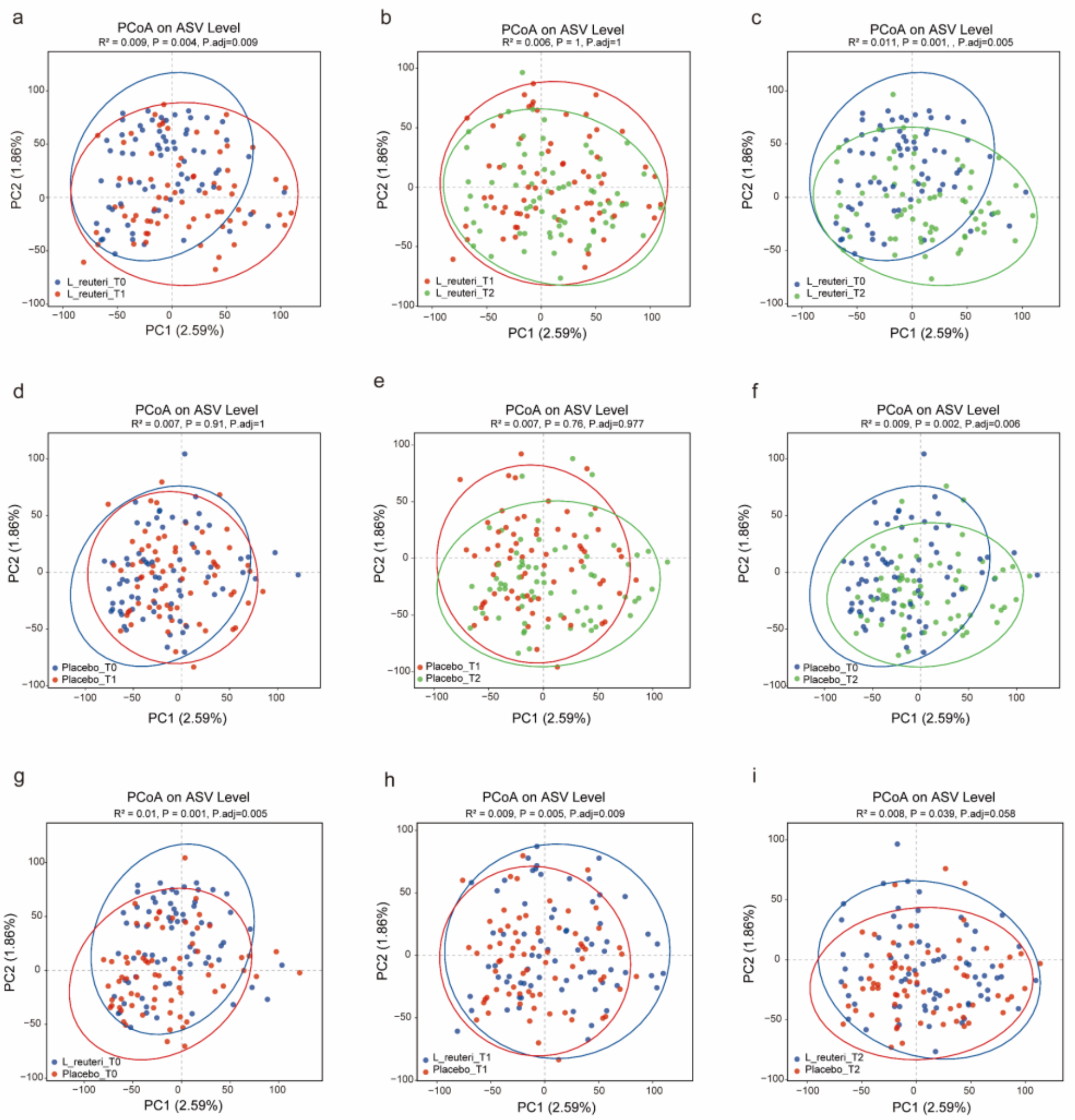

a-c, β diversity of oral microbiota in the *L. reuteri* group at baseline (T0) vs mid-radiotherapy (T1), T1 vs end of radiotherapy (T2), and T0 vs T2; **d-f,** β diversity of oral microbiota in the placebo group at T0 vs T1, T1 vs T2, and T0 vs T2; **g-i,** β diversity of oral microbiota comparing *L. reuteri* and placebo groups at T0, T1, and T2. All β diversity analyses were performed using principal coordinates analysis (PCoA) based on Aitchison distances, with statistical significance tested by permutational multivariate analysis of variance (PERMANOVA) and corrected for multiple comparisons using the false discovery rate (FDR) method.

Comment 5: *This remains a major concern. A more thoughtful and focused analysis is needed to demonstrate how baseline microbiome composition may have influenced the study outcomes and how it was modulated by the intervention. Here are specific comments:*

Supplementary Figure 9g shows a significant difference in the microbiome between the intervention and placebo groups at baseline. Differential abundance analysis should be performed, and the identified DA species (not just the Shannon index) should be included in the efficacy analysis to assess whether they confounded the effect of the intervention.

Response to Comment 5:

We sincerely thank you for this constructive comment. In response, we incorporated into our adjusted statistical model a representative baseline differentially abundant microbial species (*Actinomyces graevenitzii*) that was selected for having the largest effect size and well-established biological relevance to OM.[5,6] After adjustment for the baseline abundance of *Actinomyces graevenitzii*, the effect of the *L. reuteri* supplementation on OM incidence remained statistically significant (RR=0.71, 95%CI: 0.57-0.88, *P*=0.002), whereas the abundance of *Actinomyces graevenitzii* showed no significant association with OM incidence (RR=1.00, 95%CI: 0.99-1.00, *P*=0.38).

Furthermore, we performed additional sensitivity analysis to evaluate the robustness of our findings. First, we adjusted the multivariable model for the first principal component (PC1) of baseline β diversity, along with other clinical covariates including tumor site, concurrent chemotherapy, oral radiation dose, smoking history, alcohol use, and the Shannon index. Second, we established a model only containing the intervention and the officially named differentially abundant species (Supplementary Fig. 15a), with all other clinical covariates excluded. Both analyses

supported that *L. reuteri* supplementation may serve as a protective factor against OM. The results of the sensitivity analysis are presented in the table below.

Accordingly, the specific revisions made in the Supplementary Information are as follows:

Page 4 in the revised Supplementary Information:

"For the PPS analysis, Model 2 further incorporated the baseline Shannon index and the absolute abundance of *Actinomyces graevenitzii*, alongside smoking and alcohol history, to evaluate the intervention effect while controlling for baseline compositional differences in the microbiome."

Table for reviewers: Sensitivity analysis adjusting for the first principal component (PC1) of baseline β diversity and other clinical covariates (tumor site, concurrent chemotherapy, oral radiation dose, smoking history, alcohol consumption, Shannon index). Risk ratio (RR), hazard ratio (HR), and marginal effect estimates are presented for OM incidence, onset, and duration, respectively. For OM duration, the mean ratio (MR) is reported for the binary intervention group and the average marginal effect (AME) is reported for the continuous variable (PC1).

	OM incidence		OM onset		OM duration	
	RR (95%CI)	*P*	HR (95%CI)	*P*	MR/AME (95%CI)	*P*
Group	0.694 (0.560-0.859)	<0.001	0.233 (0.142-0.383)	<0.001	0.559 (0.388-0.751)	0.045
PC1	1.000 (0.997-1.004)	0.908	0.998 (0.991-1.005)	0.577	-0.072 (-0.186, 0.043)	0.252

Table for reviewers: Sensitivity analysis adjusting for the intervention group and the abundance of differentially abundant microbial species. RR, HR, and marginal effect estimates are presented for OM incidence, onset, and duration, respectively. For

OM duration, the MR is reported for the binary intervention variable, while the AME is reported for continuous microbial abundance variables.

	OM incidence		OM onset		OM duration	
	RR (95%CI)	*P*	HR (95%CI)	*P*	MR/AME (95%CI)	*P*
Group	0.633 (0.476-0.841)	0.002	0.299 (0.181-0.495)	<0.001	0.487 (0.305-0.688)	0.007
Actinomyces graevenitzii	1.000 (1.000-1.000)	0.656	1.000 (1.000-1.001)	0.494	0.007 (-0.004, 0.018)	0.234
Oribacterium sinus	1.001 (1.000-1.003)	0.037	1.002 (0.999-1.005)	0.250	0.064 (0.004-0.123)	0.219
Fusobacterium periodonticum	1.000 (1.000-1.000)	0.074	1.000 (1.000-1.001)	0.204	0.004 (-0.004, 0.013)	0.660
Prevotella pallens	0.999 (0.997-1.001)	0.189	0.998 (0.995-1.001)	0.248	-0.048 (-0.113, 0.016)	0.290
Neisseria subflava	1.000 (1.000-1.000)	0.001	1.000 (1.000-1.000)	0.075	0.005 (-2.2e-4, 0.010)	0.250
Lancefieldella parvula	1.000 (0.999-1.001)	0.849	1.000 (0.998-1.002)	0.786	0.010 (-0.018, 0.038)	0.338
Johnsonella ignava	1.005 (1.001-1.008)	0.006	1.019 (1.004-1.034)	0.010	0.504 (-0.248, 1.255)	0.343

Comment 6: *A more important question is whether baseline (T0) microbiome predicts development of OM/SOM overall (i.e., independent of the intervention) and within each study arm. The first part is addressed in Supplementary Figure 11a–c, but although the beta diversity analysis is only marginally significant (0.07), DA analysis should be carried out to identify taxa (species or genera) that could potentially predict OM/SOM. The authors attempted to perform stratified analysis by intervention in Supplementary Figure 11d–g, but used data from all time points (likely misunderstanding what was meant by "overall"), which is not appropriate. As in a–c, the stratified analysis should be limited to T0 samples and accompanied by DA analysis. As these analyses are based on baseline data, they are predictive in nature and should be framed as such.*

Response to Comment 6:

We sincerely thank you for this insightful and constructive comment. We have now

revised the analyses, limiting all stratified microbiome analyses to baseline (T0) samples and supplementing these with differential abundance testing to identify predictive microbial taxa in the revised Supplementary Fig. 11. The results showed that, compared with participants who subsequently developed OM, those remaining OM-free during radiotherapy had significantly lower baseline abundances of known oral pathobionts, such as *Campylobacter concisus* and *Scardovia wiggsiae*.[7,8] These findings indicate that baseline carriage of these species may help predict an increased risk of OM in patients with head and neck cancer undergoing radiotherapy.

Accordingly, the specific revisions made in the Supplementary Information are as follows:

Page 16 in the revised Supplementary Information:

Supplementary Fig. 11. Salivary microbiota profiles stratified by subsequent OM development using baseline samples in the overall cohort.

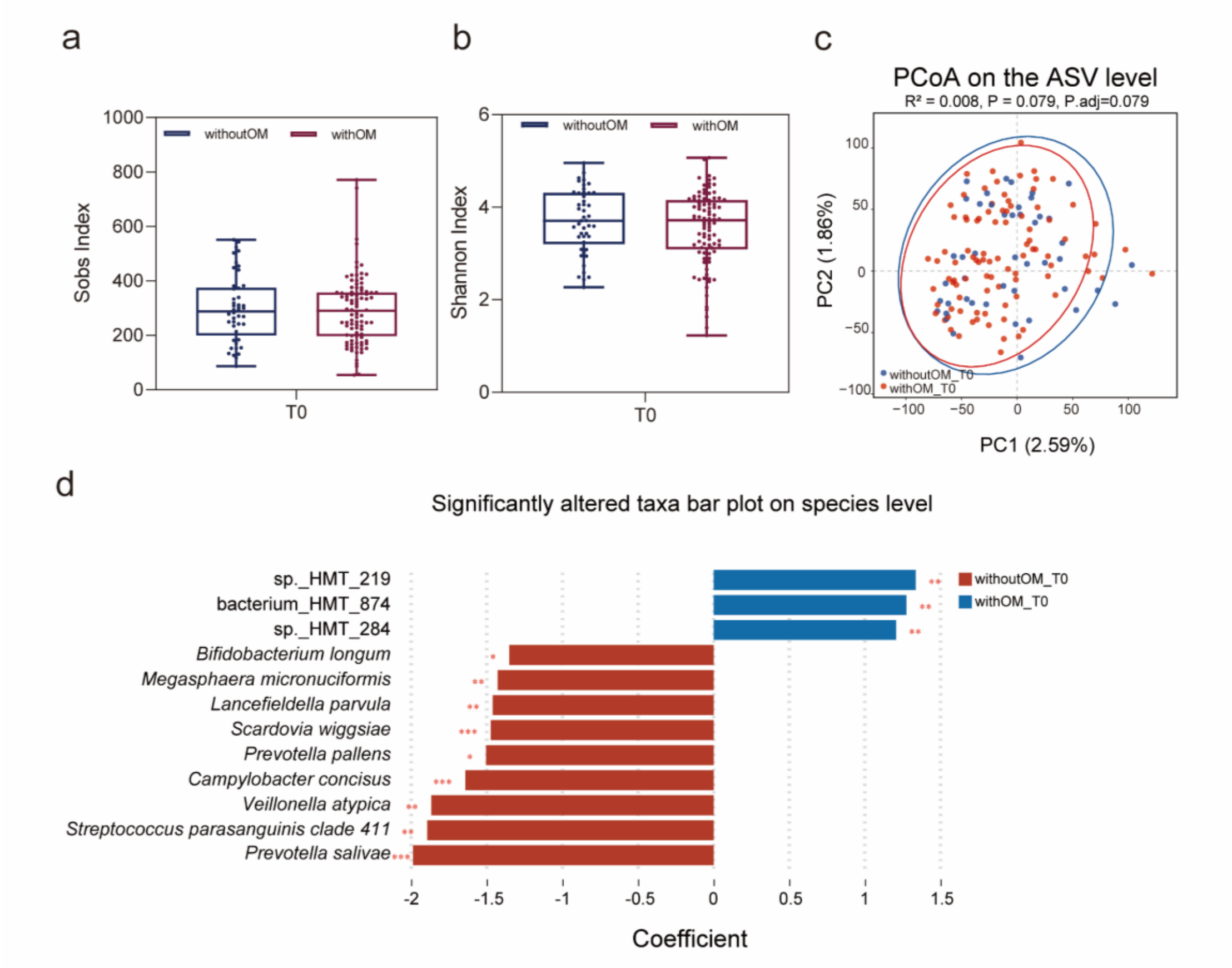

(**a-b**) α diversity: Sobs and Shannon indices comparing salivary microbiota profiles stratified by subsequently OM status at baseline. (**c**) β diversity: PCoA plots based on Aitchison distance at the ASV level illustrating salivary microbiota profiles at baseline, stratified by subsequently OM status. Statistical significance was assessed by PERMANOVA (R^2 and *P* values are indicated), with *P* values adjusted using the FDR correction. (d) Significantly differentially abundant bacterial taxa between baseline samples from patients with subsequent OM and those remaining OM-free, identified by MaAsLin2. *P* values were adjusted for multiple comparisons: * $P < 0.05$, ** $P < 0.01$, *** $P < 0.001$. Abbreviations: OM, oral mucositis; T0, baseline; withOM, patients with oral mucositis; withoutOM, patients without oral mucositis; withOM_T0, baseline samples from patients who subsequently developed OM; withoutOM_T0, baseline samples from patients who remained OM-free; ASV, amplicon sequence variant; PCoA, principal coordinates analysis; FDR, false discovery rate.

Comment 7: *Accordingly, the data presented in Supplementary Figure 12 are not meaningful, since all time points were included. Instead, the authors should assess the microbiome associated with development of OM/SOM at T1 and, separately, T2, both overall (i.e., independent of the intervention) and stratified by intervention. This may provide important insight into why the intervention was effective only in a subset of patients (e.g., probiotic colonization occurring only in a subset). These analyses are*

associative rather than predictive and should be clearly interpreted in that context.

Response to Comment 7:

We sincerely thank you for this constructive and valuable comment. We have supplemented relevant analyses to evaluate oral microbiome profiles linked to OM occurrence at mid-radiotherapy (T1) and end-radiotherapy (T2), covering overall population and intervention-stratified subgroups. The results showed that participants who did not develop OM exhibited higher relative abundances of typical probiotics such as *L. reuteri*, *Limosilactobacillus fermentum*, and *Streptococcus salivarius* compared with those who developed OM at the end of radiotherapy (Supplementary Fig. 12f). When stratified by intervention allocation, among patients who developed OM, the placebo group showed a higher relative abundance of pro-inflammatory taxa such as *Prevotella dentalis* and *Fusobacterium nucleatum* subsp. *animalis* at the end of radiotherapy (Fig. 4j). Importantly, among patients who developed OM, *L. reuteri* supplementation appeared to be associated with shifts in the oral microbial landscape relative to placebo. Specifically, the *L. reuteri* group was characterized by higher abundances of beneficial commensals, including *Rothia mucilaginosa* and *Streptococcus sanguinis* at the end of radiotherapy (Fig. 4j). Furthermore, we performed within-group comparisons according to OM status (Supplementary Fig. 14), which demonstrated that in the *L. reuteri* group, participants without OM had higher abundances of beneficial commensals, including *L. reuteri* and *Streptococcus salivarius*, compared with those who developed OM at the end of radiotherapy (Supplementary

Fig. 14q). All the above findings were interpreted as associative outcomes as suggested.

Accordingly, the specific revisions made in the Supplementary Information are as follows:

Page 17-20 in the revised Supplementary Information:

Supplementary Fig. 12. Salivary microbiota profiles stratified by OM status at mid-radiotherapy and radiotherapy completion in the overall cohort.

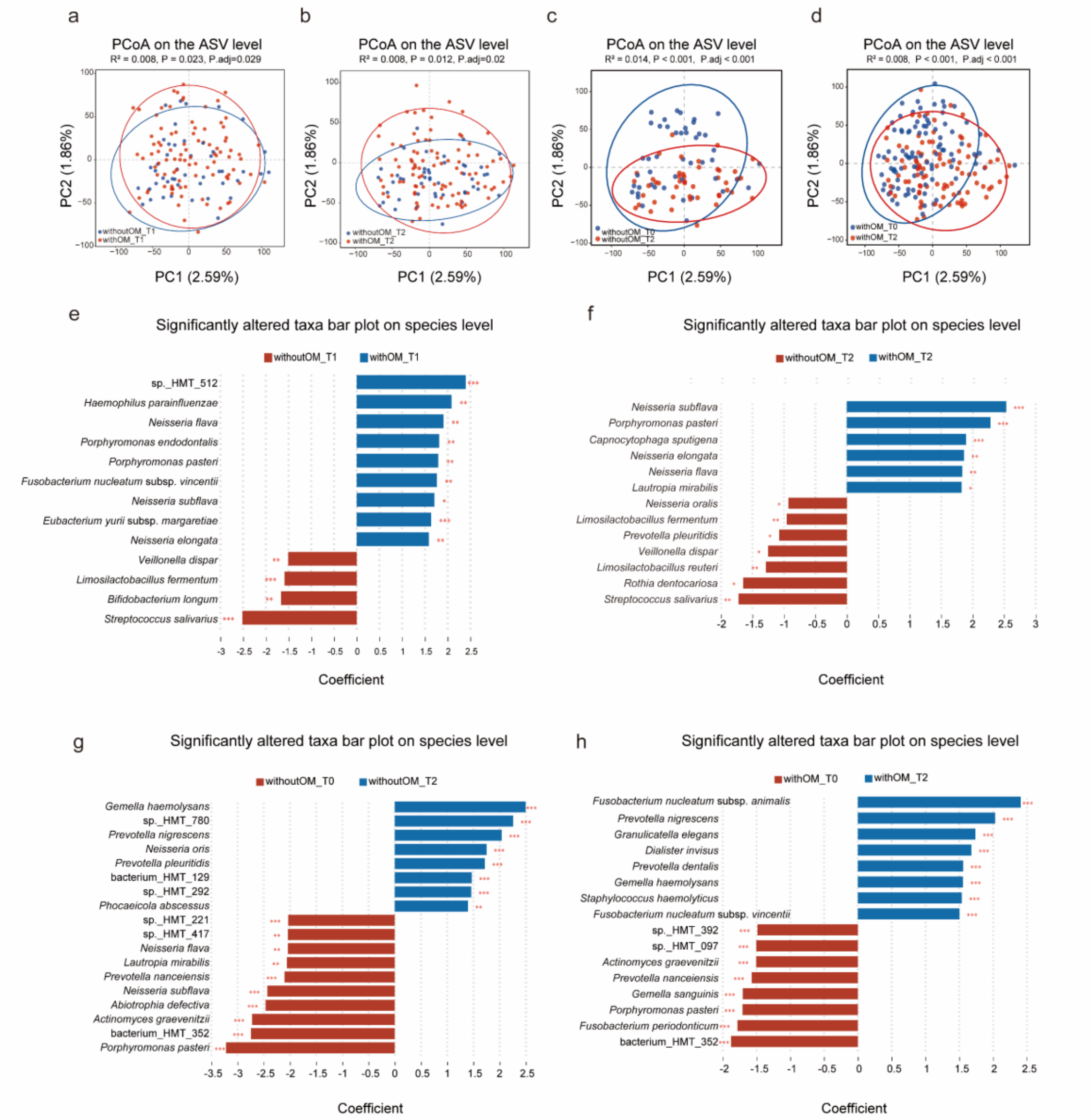

(**a-d**) PCoA plots based on Aitchison distances showing salivary microbial β diversity. Comparisons were presented between patients with and without OM at mid-radiotherapy (**a**) and radiotherapy completion (**b**), and longitudinal changes from baseline to radiotherapy completion within the non-OM (**c**) and OM (**d**) groups. R^2 and *P* values were calculated using PERMANOVA, with *P* values adjusted using the FDR correction. (**e-h**) Differentially abundant taxa at the species level identified by MaAsLin2 analysis. Bar plots illustrate significant differences between OM and non-OM groups at T1 (**e**) and T2 (**f**), and shifts from T0 to T2 within the non-OM (**g**) and OM (**h**) groups. *P* values were adjusted for multiple comparisons using the FDR correction: * $P < 0.05$, ** $P < 0.01$, *** $P < 0.001$. Abbreviations: OM, oral mucositis; T0, baseline; T1, mid-radiotherapy; T2, end-radiotherapy; withOM_T0, baseline samples from patients who subsequently developed OM; withoutOM_T0, baseline samples from patients who remained OM-free; withOM_T1, patients with oral mucositis at mid-radiotherapy; withoutOM_T1, patients without oral mucositis at mid-radiotherapy; withOM_T2, patients with oral mucositis at end-radiotherapy; withoutOM_T2, patients without oral mucositis at end-radiotherapy; ASV, amplicon sequence variant; PCoA, principal coordinates analysis.

Supplementary Fig. 13. Comparisons of salivary microbiota between the *Limosilactobacillus reuteri* and placebo groups stratified by OM status at assessed timepoints.

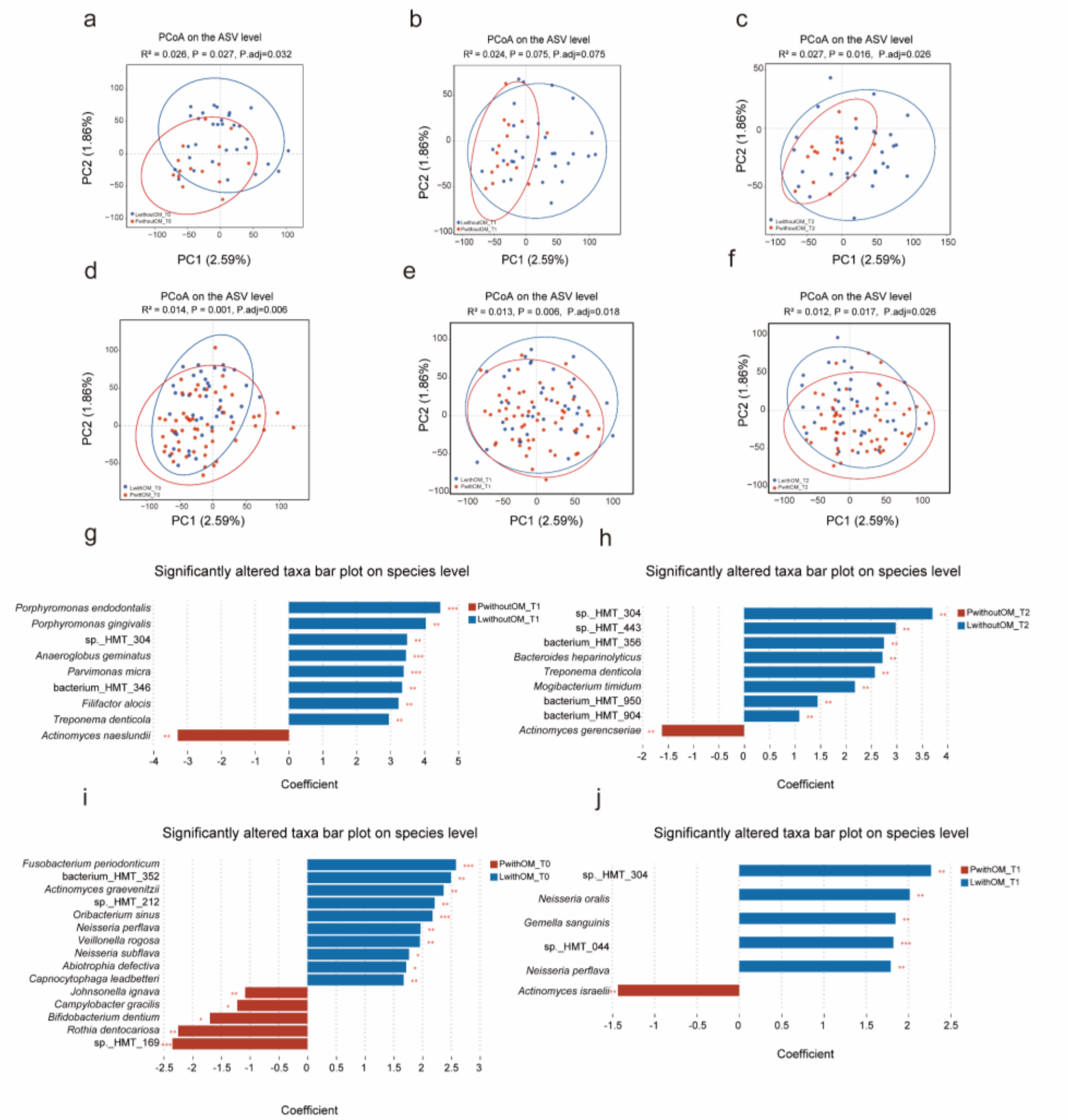

(**a-f**) PCoA plots based on Aitchison distances showing β diversity between the *L. reuteri* and placebo groups. Comparisons were presented for patients without OM at T0 (**a**), T1 (**b**), and T2 (**c**), and for patients with OM at T0 (**d**), T1 (**e**), and T2 (**f**). *P* values were determined by PERMANOVA, with *P* values adjusted using the FDR correction. (**g-j**) Differentially abundant taxa at the species level between the *L. reuteri* and placebo groups identified by MaAsLin2. Bar plots show significant differences in the non-OM group at T1 (**g**) and T2 (**h**), and in the OM group at T0 (**i**) and T1 (**j**). No significant differential taxa were identified at T0 in non-OM patients; T2-associated taxa in OM patients were presented in Fig. 4. *P* values were adjusted for multiple comparisons using the FDR correction: * $P < 0.05$, ** $P < 0.01$, *** $P < 0.001$. Abbreviations: OM, oral mucositis; T0, baseline; T1, mid-radiotherapy; T2, end-radiotherapy; LwithOM_T0, baseline samples from patients in the *L. reuteri* group who subsequently developed OM; LwithoutOM_T0, baseline samples from patients in the *L. reuteri* group who remained OM-free; LwithOM_T1, patients with OM in the *L. reuteri* group at mid-radiotherapy; LwithoutOM_T1, patients without OM in the *L. reuteri* group at mid-radiotherapy; LwithOM_T2, patients with OM in the *L. reuteri* group at radiotherapy completion; LwithoutOM_T2, patients without OM in the *L. reuteri* group at radiotherapy completion; PwithOM_T0, baseline samples from patients in the placebo group who subsequently developed OM; PwithoutOM_T0, baseline samples from patients in the placebo group who remained OM-free; PwithOM_T1, patients with OM in the placebo group at mid-radiotherapy; PwithoutOM_T1, patients without OM in the placebo group at mid-radiotherapy; PwithOM_T2, patients with OM in the placebo group at radiotherapy completion; PwithoutOM_T2, patients without OM in the placebo group at radiotherapy completion; ASV, amplicon sequence variant; PCoA, principal coordinates analysis; *L.reuteri, Limosilactobacillus reuteri*.

Supplementary Fig. 14. Comparative salivary microbiota dynamics stratified by OM status within the placebo and *Limosilactobacillus reuteri* groups.

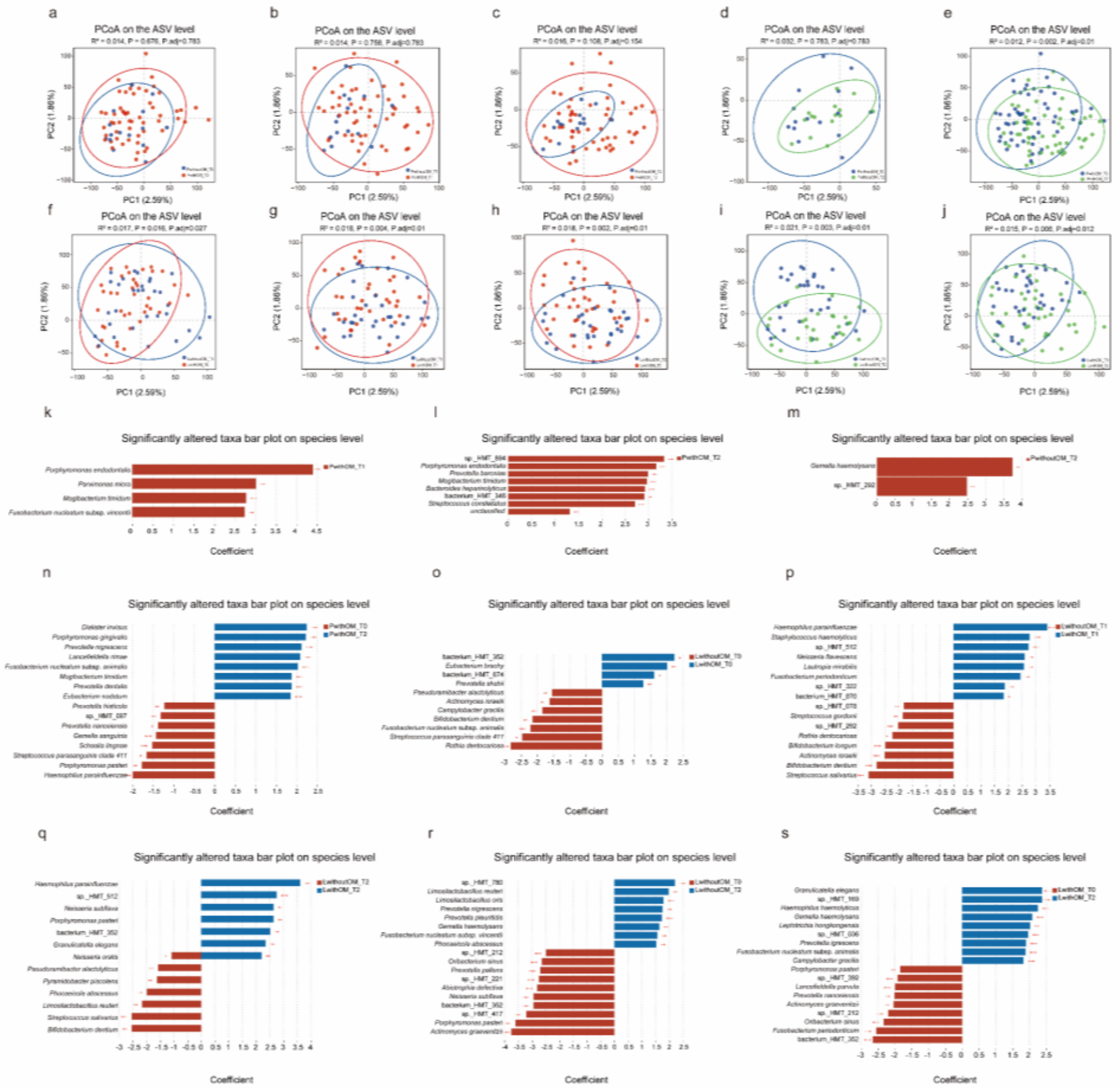

(**a-j**) PCoA plots based on Aitchison distances illustrating salivary microbial β diversity. Panels (**a–e**) represent the placebo group: comparisons between patients with and without OM at T0 (**a**), T1 (**b**), and T2 (**c**), alongside longitudinal shifts from T0 to T2 within the non-OM (**d**) and OM (**e**) subgroups. Panels (**f-j**) represent the *L. reuteri* group: comparisons by OM status at T0 (**f**), T1 (**g**), and T2 (**h**), and longitudinal shifts from T0 to T2 for the non-OM (**i**) and OM (**j**) subgroups. Statistical significance was assessed by PERMANOVA, with *P* values adjusted using the FDR correction. (**k-s**) Differentially abundant taxa at the species level identified by MaAsLin2. For the placebo group (**k-n**): significant differences between patients with and without OM at T1 (**k**) and T2 (**l**), and shifts from T0 to T2 for the non-OM (**m**) and OM (**n**) subgroups. No significant differences were observed at T0. For the *L. reuteri* group (**o–s**): significant differences by OM status at T0 (**o**), T1 (**p**), and T2 (**q**), and shifts from T0 to T2 for the non-OM (**r**) and OM (**s**) subgroups. *P* values are adjusted for multiple comparisons: * $P < 0.05$, ** $P < 0.01$, *** $P < 0.001$. Abbreviations: OM, oral mucositis; T0, baseline; T1, mid-radiotherapy; T2, end-radiotherapy; LwithOM_T0, baseline samples from patients in the *L. reuteri* group who subsequently developed OM; LwithoutOM_T0, baseline samples from patients in the *L. reuteri* group who remained OM-free; LwithOM_T1, patients with OM in the *L. reuteri* group at mid-radiotherapy; LwithoutOM_T1, patients without OM in the *L. reuteri* group at mid-radiotherapy; LwithOM_T2, patients with OM in the *L. reuteri* group at radiotherapy completion; LwithoutOM_T2, patients without OM in the *L. reuteri* group at radiotherapy completion; PwithOM_T0, baseline samples from patients in the placebo group who subsequently developed OM; PwithoutOM_T0, baseline samples from patients in the placebo group who remained OM-free; PwithOM_T1, patients with OM in the placebo group at mid-radiotherapy; PwithoutOM_T1, patients without OM in the placebo group at mid-radiotherapy; PwithOM_T2, patients with OM in the placebo group at radiotherapy completion; PwithoutOM_T2, patients without OM in the placebo group at radiotherapy completion; ASV, amplicon sequence variant; PCoA, principal coordinates analysis; *L.reuteri, Limosilactobacillus reuteri*.

Supplementary Fig. 15. Taxonomic and predicted functional shifts between the *Limosilactobacillus reuteri* and placebo groups.

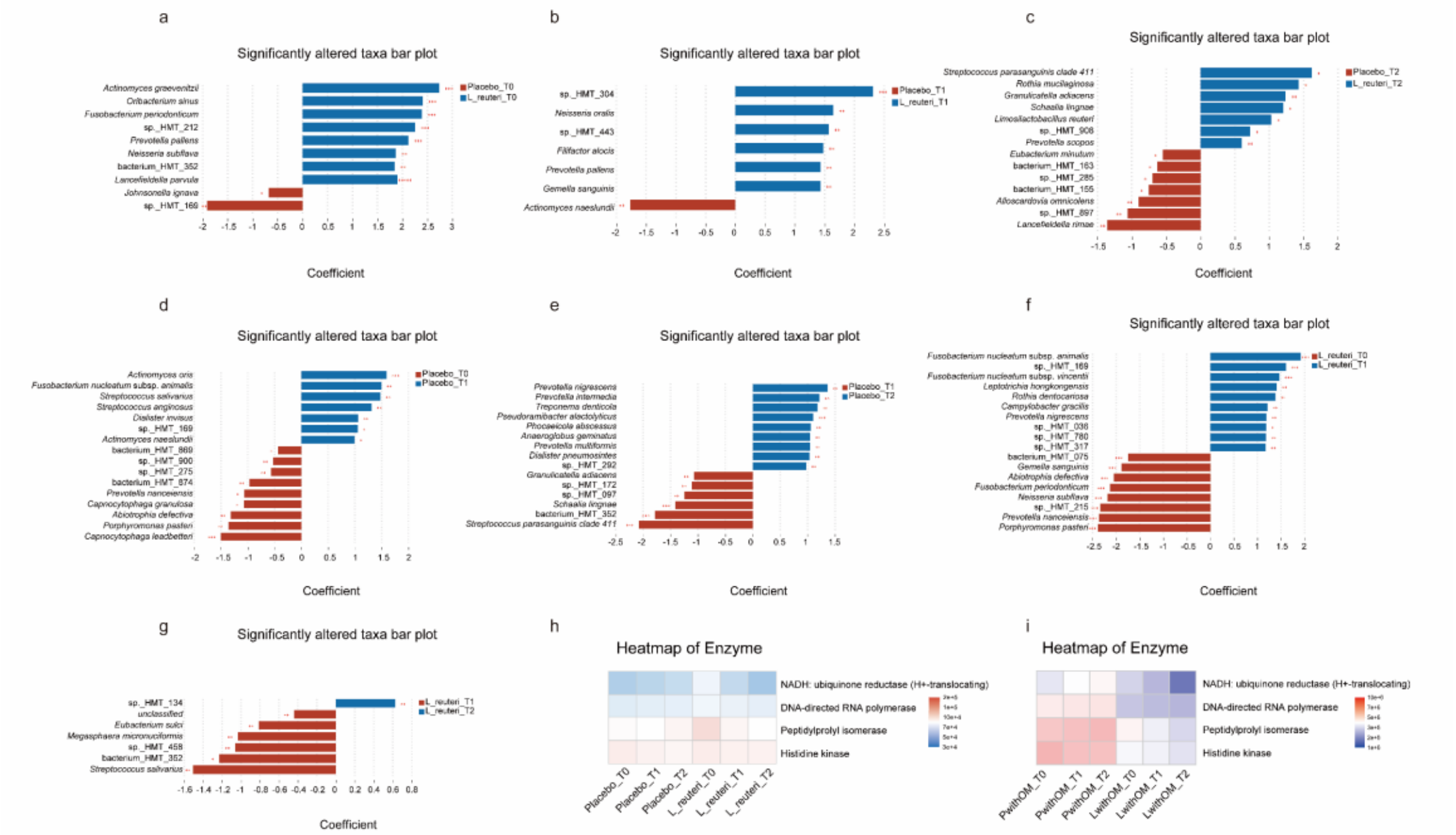

(**a-c**) Differentially abundant species between the *L. reuteri* and placebo groups at T0 (**a**), T1 (**b**), and T2 (**c**) identified by MaAsLin2 analysis. (**d-g**) Longitudinal shifts in microbial composition within the placebo group (T1 vs. T0, **d**; T1 vs. T2, **e**) and the *L. reuteri* group (T1 vs. T0, **f**; T1 vs. T2, **g**). (**h-i**) Heatmaps showing predicted functional profiles (enzymes) based on PICRUSt2 across timepoints in the overall cohort (**h**) and stratified by patients who developed OM (**i**). *P* values were adjusted for multiple comparisons: * $P < 0.05$, ** $P < 0.01$, *** $P < 0.001$. ASV: amplicon sequence variant; PC, principal component; PCoA, principal coordinates analysis; Placebo_T0, prior to radiotherapy with a placebo; Placebo_T1, the mid-radiotherapy with a placebo; Placebo_T2, the end of radiotherapy with a placebo; *L. reuteri*, *Limosilactobacillus reuteri*; *L. reuteri*_T0, prior to radiotherapy with the probiotic *Limosilactobacillus reuteri*; *L. reuteri*_T1, the mid-radiotherapy with the probiotic *Limosilactobacillus reuteri*; *L. reuteri*_T2, the end of radiotherapy with the probiotic *Limosilactobacillus reuteri*.

Comment 8: *Figure 4: I think this should be used to carefully present microbiome data most relevant to the study objective and outcomes, i.e. 1) Effect of the intervention on the microbiome (e.g. Longitudinal changes T0 T1 T02 including DA analysis results); and 2) Microbiome differences/changes in relation to OM/SOM (both baseline and follow-up). Perhaps, supplementary figures 11 and 12 after the revisions mentioned above. Multivariate analysis (e.g. MaAslin2) of T0, and separately T2, data including both clinical outcome and intervention as covariates would be very informative.*

Response to Comment 8:

We sincerely thank you for these constructive and insightful suggestions, which have significantly guided the restructuring of our microbiome data presentation to better align with the study's objectives. As suggested, we have completely reorganized Fig. 4 to focus on: 1) the longitudinal effect of the *L. reuteri* intervention; and 2) the microbial changes associated with OM development.

(1) Longitudinal intervention effects (Fig. 4a-h): We now present the longitudinal changes in α diversity and β diversity for both the placebo and *L. reuteri* groups from baseline through the end of radiotherapy (Fig. 4a-d). Furthermore, we performed differential abundance analysis to track specific taxa over time. We found that the placebo group exhibited a marked enrichment of opportunistic pathogens associated with oral inflammation at the end of radiotherapy, such as *Fusobacterium nucleatum* subsp. *animalis* and *Prevotella nigrescens* (Fig. 4e, g). In contrast, *L. reuteri* supplementation appeared to stabilize the microbial ecosystem by suppressing these pathogens and promoting commensal taxa like *Gemella haemolysans* (Fig. 4f, h).

(2) Microbiome signatures related to OM (Fig. 4i-j): We performed additional differential species analysis on the overall population stratified by OM status. The results showed that at baseline, participants who subsequently remained OM-free had significantly lower abundances of pathobionts such as *Campylobacter concisus* and *Scardovia wiggsiae* compared with those who developed OM (Fig. 4i).

(3) Interaction between intervention and OM status (Fig. 4j): Among patients who

developed OM, *L. reuteri* supplementation appeared to be associated with a distinct microbial landscape compared to the placebo group at the end of radiotherapy. Specifically, the *L. reuteri* group showed higher abundances of beneficial commensals (e.g., *Rothia mucilaginosa*, *Streptococcus sanguinis*) and lower abundances of potent pathogens (e.g., *Prevotella dentalis*) at the end of radiotherapy.

These findings, now more clearly visualized in revised Figure 4, suggest that *L. reuteri* may modulate the oral microbiota to attenuate pathogen blooms and sustain the growth of commensals, thereby preserving a more balanced microbial community even upon clinical OM onset.

Accordingly, the specific revisions made in the manuscript are as follows:

Page 10, Line 200 in the revised manuscript:

"Differential species analysis revealed that the placebo group exhibited higher abundances of several opportunistic pathogens associated with oral inflammation at the end of radiotherapy, including *Fusobacterium nucleatum* subsp. *animalis* and *Prevotella nigrescens* (Fig. 4e, g). In contrast, *L. reuteri* supplementation appeared to stabilize the microbial ecosystem by suppressing the overgrowth of these periodontal pathogens and promoting a community dominated by commensal taxa such as *Gemella haemolysans* (Fig. 4f, h). Notably, compared with participants who subsequently developed OM, those remaining OM-free during radiotherapy had significantly lower baseline abundances of known oral pathobionts, such as *Campylobacter concisus* and *Scardovia wiggsiae* (Fig. 4i). Importantly, among patients who developed OM, *L.*

reuteri supplementation appeared to alter the microbial landscape compared to the placebo. Specifically, the *L. reuteri* group was characterized by higher abundances of beneficial commensals like *Rothia mucilaginosa* and *Streptococcus sanguinis*, while being associated with lower abundances of potent pathogens such as *Prevotella dentalis* and *Fusobacterium nucleatum* subsp. *animalis* (Fig. 4j). These findings suggest that *L. reuteri* may promote oral microbial homeostasis by constraining the expansion of radiotherapy-induced pathogens and sustaining a resilient commensal community, even in the context of clinical OM. In addition, we performed microbiome comparisons in the overall cohort stratified by OM status and between the *L. reuteri* and placebo groups; the results were consistent with the above findings (Supplementary Fig. 11-14)."

Page 14, Line 285 in the revised manuscript:

"We performed longitudinal profiling of the oral microbiota during radiotherapy to investigate the impact of *L. reuteri* supplementation on RIOM. Our findings indicate that *L. reuteri* supplementation may contribute to the stabilization of the oral microbiota during radiotherapy by preserving commensal species, inhibiting pathogenic bacteria, and enriching specific metabolic pathways. Specifically, the higher abundances of oral health-associated taxa including *Rothia mucilaginosa* and *Streptococcus sanguinis* among patients who developed OM within the *L. reuteri* group at the end of radiotherapy indicate that *L. reuteri* supplementation may be associated with reduced radiation-induced perturbation of the oral microbial community.[35,36] In contrast, patients with OM receiving placebo exhibited higher abundances of *Prevotella dentalis*

and *Fusobacterium nucleatum*, taxa that are associated with dental caries, periodontal inflammation, and opportunistic pathogenicity in the oral cavity.[37-40] Their enrichment may further aggravate radiation-induced oral tissue injury."

Fig. 4. Impact of *Limosilactobacillus reuteri* administration on the salivary microbiota during radiotherapy.

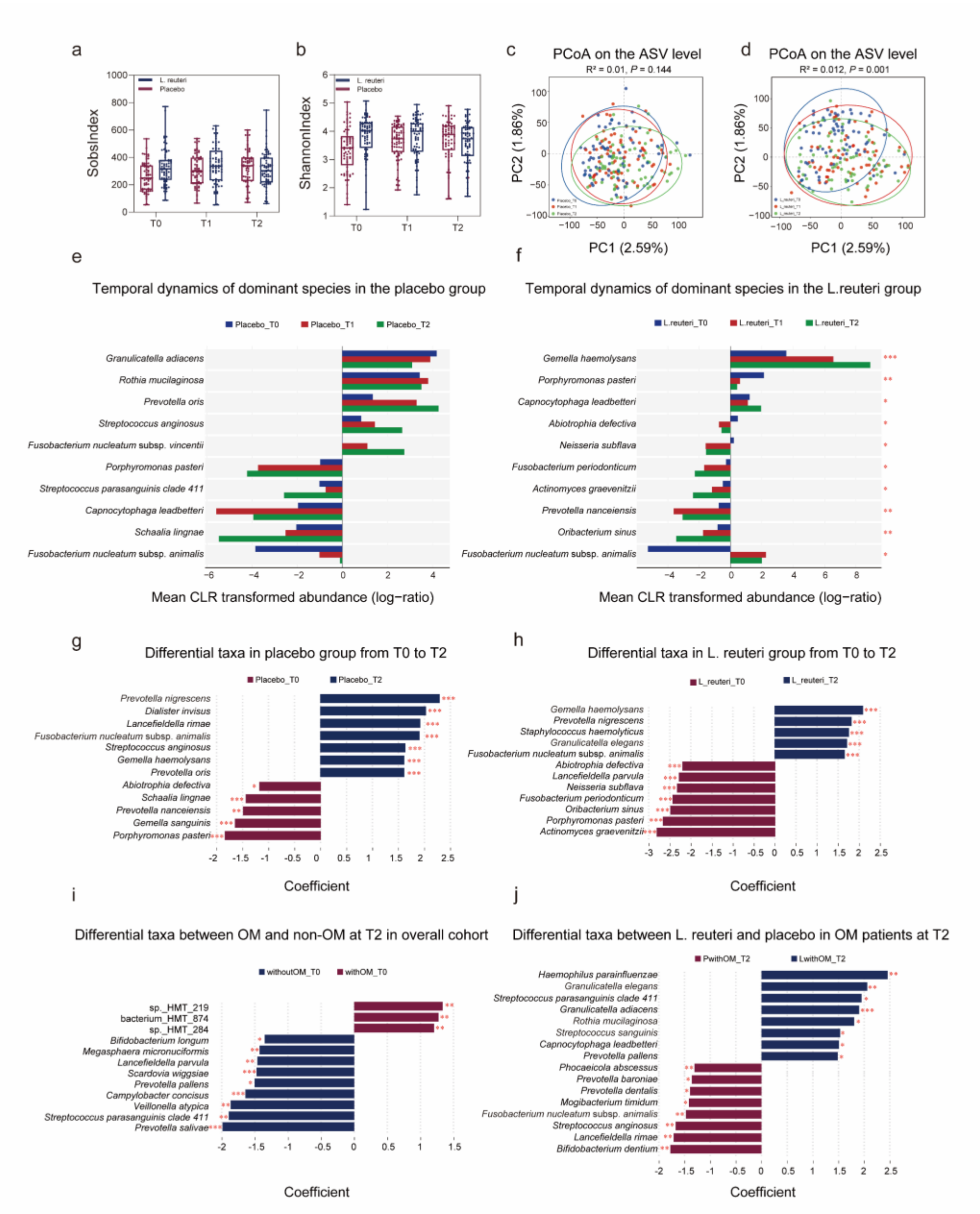

a-b The α diversity of the oral microbiota was evaluated using the Sobs and Shannon indices. For box-and-whisker plots, boxes represent the interquartile range (IQR) with the median indicated by a horizontal line, and whiskers extend to the minimum and maximum observed values. **c-d** β diversity was assessed using Aitchison distances calculated from centered log-ratio (CLR) transformed taxon counts, visualized via principal coordinate analysis (PCoA) plots, and statistically validated by PERMANOVA.

e-f Temporal dynamics of dominant taxa at the species level in the placebo (**e**) and *L. reuteri* (**f**) groups. Adjusted *P* values were calculated using the Kruskal-Wallis H test with Benjamini-Hochberg FDR correction for multiple testing. **g-j** Differentially abundant taxa identified by MaAsLin2 analysis. Bar plots show shifts from baseline (T0) to radiotherapy completion (T2) within the placebo (**g**) and *L. reuteri* (**h**) groups; differences between patients subsequently with and without OM in the overall cohort at T0 (**i**); and the impact of *L. reuteri* versus placebo on the microbiota of patients with OM at T2 (**j**). *P* values were adjusted for multiple comparisons using the FDR correction: **P* < .05, ***P* < .01, ****P* < .001. ASV: amplicon sequence variant; PC, principal component; PCoA, principal coordinates analysis; Placebo_T0, prior to radiotherapy with a placebo; Placebo_T1, the mid-radiotherapy with a placebo; Placebo_T2, the end of radiotherapy with a placebo; *L. reuteri*, *Limosilactobacillus reuteri*; *L. reuteri*_T0, prior to radiotherapy with the probiotic *Limosilactobacillus reuteri*; *L. reuteri*_T1, the mid-radiotherapy with the probiotic *Limosilactobacillus reuteri*; *L. reuteri*_T2, the end of radiotherapy with the probiotic *Limosilactobacillus reuteri;* withoutOM_T0, baseline samples from patients who subsequently remained OM-free; withOM_T0, baseline samples from patients who subsequently developed OM; PwithOM_T2, patients with oral mucositis in the placebo group at the end of radiotherapy; LwithOM_T2, patients with oral mucositis in the *Limosilactobacillus reuteri* group at the end of radiotherapy.

Reviewer #5

Comment 1: *We thank the authors for their detailed point-by-point responses and revisions. Several concerns from the first round have been adequately addressed. However, a number of methodological issues remain unresolved or insufficiently responded to, as outlined below.*

Response to Comment 1:

We sincerely apologize that our first-round revisions have not fully addressed your concerns. We have further revised the manuscript according to your valuable suggestions, with detailed point-by-point responses provided below.

Comment 2: *In response to our previous comment, the authors confirmed that the proportional hazards assumption was violated for both SOM and OM onset (Schoenfeld residuals test, P < 0.05), and conducted a piecewise Cox regression as a sensitivity analysis. However, the results of this piecewise Cox analysis were presented only in a "Table for reviewers" and have not been incorporated into the manuscript or*

supplementary materials. Given that the proportional hazards assumption has been formally shown to be violated, the overall hazard ratios reported in Table 2 for onset endpoints may not be statistically valid as standalone effect estimates. The authors are requested to include the piecewise Cox regression results in the supplementary materials and to add appropriate discussion of this limitation in the manuscript.

Response to Comment 2:

We sincerely thank you for the insightful suggestions. We have added the results of piecewise Cox regression analysis to the Supplementary Information and included corresponding discussion regarding this limitation in the revised manuscript.

Accordingly, the specific revisions made in the manuscript and Supplementary Information are as follows:

Page 8, Line 153 in the revised manuscript:

"Notably, although the proportional hazards assumption was violated for the onset endpoints, which may be attributed to the acute characteristics of OM, piecewise Cox regression revealed that the effect was still significant during the initial segment (Supplementary Table 6)."

Page 16, Line 330 in the revised manuscript:

"Furthermore, the proportional hazards assumption was violated for onset endpoints; thus, the corresponding overall hazard ratios may not be statistically valid as standalone effect estimates."

Page 34, the footnote of Table 2 in the revised manuscript:

"As the proportional hazards assumption was violated for onset endpoints, the results of the piecewise Cox regression are presented in Supplementary Table 6."

Page 23 in the revised Supplementary Information:

Supplementary Table 6. Results of the piecewise Cox regression analysis for SOM/OM onset in the intention-to-treat population.

		HR (95% CI)	*P*
SOM onset	<36 days	0.37 (0.23-0.59)	<0.001
	⩾36 days	2.96 (0.34-25.44)	0.323
OM onset	<30 days	0.40 (0.27-0.58)	<0.001
	⩾30 days	1.00 (0.10-10.33)	1.000

Abbreviations: OM, oral mucositis; SOM, severe OM.

Comment 3: *In response to our previous comment, the authors adopted the two-part (Hurdle) model as the primary analytical approach for duration endpoints. However, the revised Table 2 continues to report the median SOM duration as 0.0 days for the L. reuteri group alongside the mean ratio from the two-part model. Given that this median value was previously acknowledged to be a mathematical artifact of the zero-inflated distribution rather than a clinically meaningful estimate, its continued presence in the table risks misleading readers. The authors are requested to either remove the median duration from Table 2 and throughout the paper, or add a clear explanatory footnote.*

Response to Comment 3:

Thank you very much for your careful and insightful comments. We have removed all median values for duration endpoints from Table 2 and throughout the manuscript and Supplementary Information to avoid any potential misinterpretation.

Comment 4: *The title of Supplementary Table 4 refers to results "with efficacy in Model 3." However, Model 3 is not defined anywhere in the manuscript, the supplementary methods, or the SAP. Only Model 1 and Model 2 are formally described. The authors are requested to either formally define Model 3 or correct the table title, and to confirm that the term "Model 3" has been removed from all parts of the manuscript.*

Response to Comment 4:

We greatly appreciate your careful review and this important comment. We have revised the title and footnote of Supplementary Table 4 by replacing "Model 3" with "Model 2", and thoroughly checked the full manuscript and Supplementary Information to ensure that the term "Model 3" has been entirely removed throughout.

Comment 5: *In the main text (Lines 131–135), the authors describe the adjusted effect estimates from Model 1 and Model 2 as "adjusted relative risk," whereas the footnote of Table 2 explicitly states that "Cox regression was used for incidence/onset in adjusted models." Cox proportional hazards regression yields a hazard ratio (HR), not a relative risk (RR). These two effect measures are conceptually and numerically distinct and are not interchangeable, and mislabeling HR as RR represents a fundamental statistical terminology error that directly contradicts the table footnote.*

Furthermore, in Table 2, the effect size for SOM/OM incidence switches from RR in the unadjusted analysis to HR in the adjusted models (Model 1 and Model 2), yet both are

presented in the same "Effect size" column without per-row labeling. This is potentially misleading, as readers may interpret the change in magnitude (e.g., RR 0.61 to HR 0.44) as solely reflecting the effect of covariate adjustment, when it also reflects a change in the effect measure itself.

The authors should use the modified Poisson regression as used in subgroup analysis if the modified RR is the desired output, or clearly state in the manuscript that HR and RR are not comparable.

Response to Comment 5:

We sincerely appreciate your careful and critical comments on statistical terminology and effect-size reporting. We have re-analyzed the adjusted incidence outcomes using modified Poisson regression throughout the manuscript, replaced all incorrectly labeled HR with adjusted RR, revised Table 2 accordingly with a clear footnote, and corrected relevant descriptions in the main text to eliminate confusion between HR and RR.

Accordingly, the specific revisions made in the manuscript are as follows:

Page 34 in the revised manuscript:

TABLE 2 Efficacy results

Parameter	*L. reuteri* (n = 80)	Placebo (n = 80)	Effect size (95% CI)	*P*
Unadjusted primary analysis				
SOM incidence, %	37.5	61.3	0.61 (0.44-0.85)	.004
SOM onset, days, median	NE	16.0	0.43 (0.27-0.67)	<.001
SOM duration, days, mean (SD)	6.4 (9.6)	14.9 (14.5)	0.43 (0.21-0.66)	<.001
OM incidence, %	56.3	78.8	0.71 (0.57-0.89)	.004

OM onset, days, median	14.0	8.0	0.41 (0.28-0.60)	<.001
OM duration, days, mean (SD)	19.9 (21.7)	35.8 (24.5)	0.56 (0.39-0.74)	.003
Model 1				
SOM incidence, %	37.5	61.3	0.62 (0.45-0.84)	.002
SOM onset, days, median	NE	16.0	0.44 (0.28-0.70)	<.001
SOM duration, days, mean (SD)	6.4 (9.6)	14.9 (14.5)	0.46 (0.25-0.70)	.01
OM incidence, %	56.3	78.8	0.72 (0.59-0.88)	.001
OM onset, days, median	14.0	8.0	0.39 (0.26-0.58)	<.001
OM duration, days, mean (SD)	19.9 (21.7)	35.8 (24.5)	0.56 (0.40-0.74)	.004
Model 2				
SOM incidence, %	37.5	61.3	0.62 (0.46-0.85)	.003
SOM onset, days, median	NE	16.0	0.44 (0.28-0.69)	<.001
SOM duration, days, mean (SD)	6.4 (9.6)	14.9 (14.5)	0.48 (0.26-0.71)	.02
OM incidence, %	56.3	78.8	0.72 (0.59-0.88)	.001
OM onset, days, median	14.0	8.0	0.36 (0.25-0.54)	<.001
OM duration, days, mean (SD)	19.9 (21.7)	35.8 (24.5)	0.57 (0.41-0.75)	.007

Note: Unadjusted incidence comparisons used the χ^2 test (RR, 95% CI); onset time used the log-rank test (HR, 95% CI); duration used a two-part model (MR, 95% CI). Model 1 adjusted for tumor site, oral radiotherapy dose, and chemotherapy. Model 2 extended Model 1 by additionally adjusting for smoking history and alcohol use history. Modified Poisson regression was used for incidence in adjusted models (aRR, 95% CI); Cox regression was used for onset in adjusted models (aHR, 95% CI); a two-part model was used for duration (aMR, 95% CI). As the proportional hazards assumption was violated for onset endpoints, the results of the piecewise Cox regression are presented in Supplementary Figure 6. Abbreviations. *L. reuteri*, *Limosilactobacillus reuteri*; OM, oral mucositis; SOM, severe OM; SD, standard deviation; RR: relative risk; HR: hazard ratio; MR: mean ratio; aRR: adjusted relative risk; aHR: adjusted hazard ratio; aMR: adjusted mean ratio; CI: confidence interval; NE, not estimable.

Comment 6: *The manuscript states (Line 440) that "Bonferroni correction for multiple comparisons was applied for secondary outcome analyses." However, the corrected significance threshold is not reported anywhere in the paper. The authors are requested to explicitly report this threshold in the manuscript so that readers can independently evaluate the statistical significance of each secondary endpoint.*

Response to Comment 6:

Thank you very much for this insightful comment. We have now explicitly reported the Bonferroni-corrected significance threshold in the Methods section. Specifically, as five secondary outcomes were analyzed, the corrected alpha level was set at 0.05/5 = 0.01.

Accordingly, the specific revisions made in the manuscript are as follows:

Page 22, Line 459 in the revised manuscript:

"Bonferroni correction was applied for secondary outcome analyses, with a corrected significance threshold of $P < 0.01$ (0.05/5)."

Reviewer #6

Comment: *I co-reviewed this manuscript with one of the reviewers who provided the listed reports. This is part of the Nature Communications initiative to facilitate training in peer review and to provide appropriate recognition for Early Career Researchers who co-review manuscripts.*

Response to Comment:

We sincerely thank you for the careful and dedicated work in co-reviewing this manuscript. We greatly appreciate your valuable insights and constructive comments, which have been instrumental in improving the quality and rigor of our work.

We sincerely hope that the revised manuscript has addressed all reviewers' comments and now meets the standards of *Nature Communications*. We would like to express our sincere gratitude to the reviewers for their valuable time and constructive suggestions.

References:

1. Peng, X., *et al.* Streptococcus salivarius K12 Alleviates Oral Mucositis in Patients Undergoing Radiotherapy for Malignant Head and Neck Tumors: A Randomized Controlled Trial. *J Clin Oncol* **42**, 1426-1435 (2024).
2. Luo, Z., *et al.* Limosilactobacillus reuteri in immunomodulation: molecular mechanisms and potential applications. *Front Immunol* **14**, 1228754 (2023).
3. Wu, H., *et al.* Lactobacillus reuteri maintains intestinal epithelial regeneration and repairs damaged intestinal mucosa. *Gut Microbes* **11**, 997-1014 (2020).
4. Sharma, A., *et al.* Lactobacillus brevis CD2 lozenges reduce radiation- and chemotherapy-induced mucositis in patients with head and neck cancer: a randomized double-blind placebo-controlled study. *Eur J Cancer* **48**, 875-881 (2012).
5. Könönen, E. Polymicrobial infections with specific Actinomyces and related organisms, using the current taxonomy. *J Oral Microbiol* **16**, 2354148 (2024).
6. Hall, V. Actinomyces--gathering evidence of human colonization and infection. *Anaerobe* **14**, 1-7 (2008).
7. Tanner, A.C., Dzink, J.L., Ebersole, J.L. & Socransky, S.S. Wolinella recta, campylobacter concisus, bacteroides gracilis, and Eikenella corrodens from periodontal lesions. *J Periodontal Res* **22**, 327-330 (1987).
8. Majumdar, B., Patil, S., Testarelli, L. & Nardo, D.D. Emerging role of Scardovia wiggsiae as an oral pathogen in early childhood caries: a scoping review. *Journal of Clinical Pediatric Dentistry* **50**, 1-8 (2026).

Response to Reviewers

Manuscript Title: *Limosilactobacillus reuteri* for Radiation-Induced Oral Mucositis in Patients with Head and Neck Cancer: A Randomized Clinical Trial

Dear Reviewers,

We would like to express our sincere gratitude for your insightful and constructive comments on our manuscript. Your feedback has been invaluable in helping us improve the quality and rigor of our work. In response to your comments, we have undertaken corresponding revisions to the manuscript. In brief, we have revised the taxonomic nomenclature, reorganized Figure 4 with additional panels, corrected the directionality of associations, applied more stringent Bonferroni corrections across all endpoints, and refined the statistical terminology. We believe that all your comments have been addressed in the revised manuscript and in this response. All changes have been tracked using the "Track Changes" function in the manuscript. Below, we provide point-by-point responses to each comment.

Reviewer #4

Comment 1: *Thank you to the authors for the substantial revision. A few more comments:*

Response to Comment 1:

Thank you again for your constructive comments on our manuscript. Your insightful suggestions have been extremely helpful in guiding our revisions. We have carefully addressed each of the remaining comments and revised the manuscript accordingly. Point-by-point responses have been provided below.

Comment 2: *For unnamed taxa ending with sp. HMT XXX, the genus name should be included throughout (text and figures), e.g. Pseudoleptotrichia sp. HMT-219.*

Response to Comment 2:

We sincerely thank you for this suggestion. We have now carefully revised the entire manuscript and all figures to ensure that for every unnamed taxon ending with "sp. HMT XXX", the genus name is consistently included.

Comment 3: *Figure 4i: the description above the panel should be corrected (T0 instead of T2). Logically, panel j should be used to present differences between OM and without OM at T2. Additional two panels should be used to show differences between Placebo and L. reuteri in OM and separately without OM groups. This will account of all major findings that need to present in the main manuscript.*

Response to Comment 3:

Thank you very much for this valuable suggestion. We have corrected the heading above panel i from "T2" to "T0". We have also revised Figure 4 as suggested: panel j now presents the comparison between OM and without OM at T2. Additionally, we have added two new panels (now panels k and l) to separately display the placebo vs. *L. reuteri* comparisons in the OM group and the non-OM group at T2.

Accordingly, the specific revisions made in Figure 4 are as follows:

Fig. 4. Comparison of salivary microbiota composition between treatment groups stratified by oral mucositis status during radiotherapy.

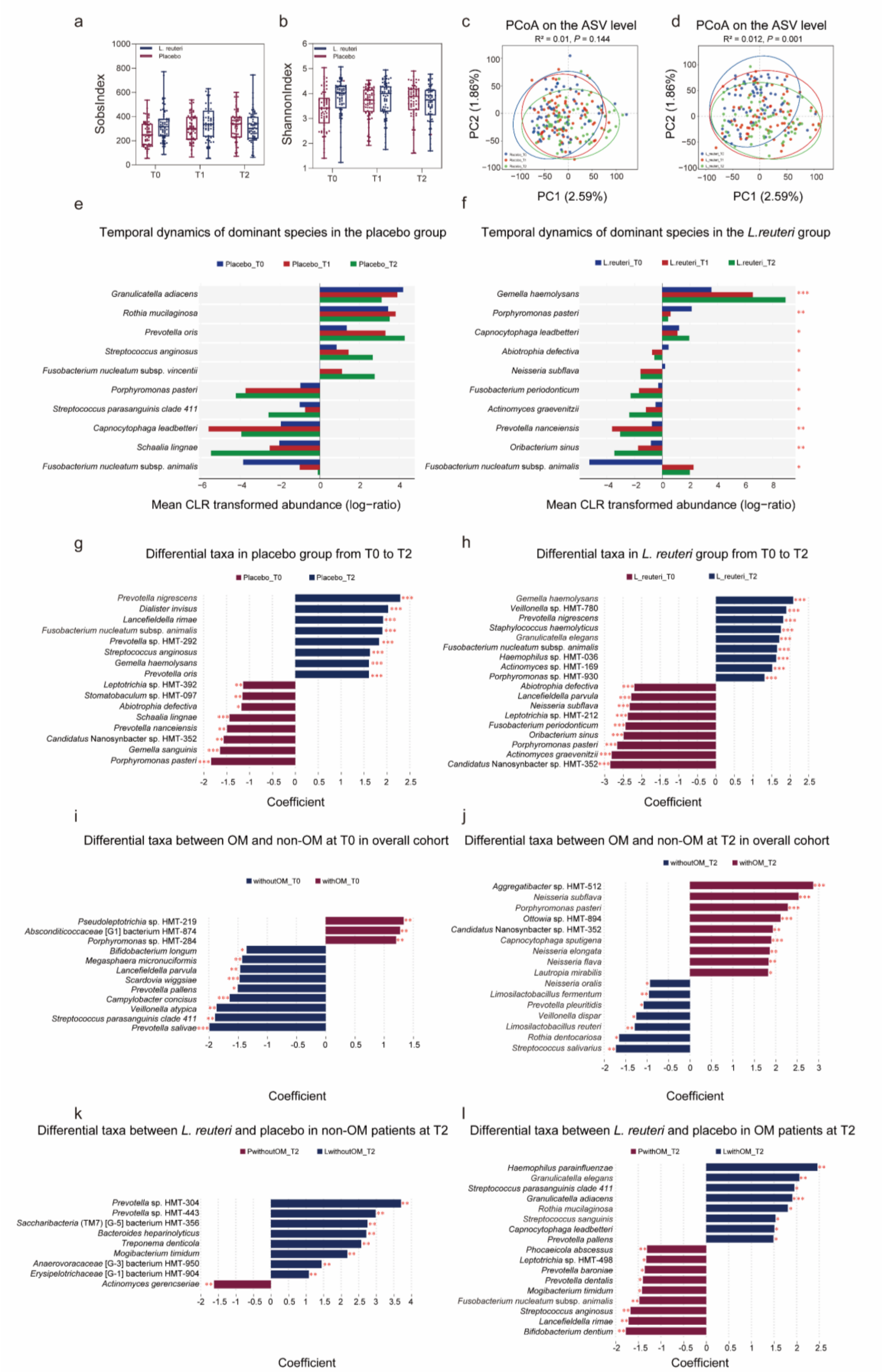

a-b The α diversity of the oral microbiota was evaluated using the Sobs and Shannon indices. For box-and-whisker plots, boxes represent the interquartile range (IQR) with the median indicated by a horizontal line, and whiskers extend to the minimum and maximum observed values. **c-d** β diversity was assessed using Aitchison distances

calculated from centered log-ratio (CLR) transformed taxon counts, visualized via principal coordinate analysis (PCoA) plots, and statistically validated by PERMANOVA. **e-f** Temporal dynamics of dominant taxa at the species level in the placebo (**e**) and *L. reuteri* (**f**) groups. Adjusted *P* values were calculated using the Kruskal-Wallis H test with Benjamini-Hochberg FDR correction for multiple testing. **g-l** Differentially abundant taxa identified by MaAsLin2 analysis. Bar plots show shifts from baseline (T0) to radiotherapy completion (T2) within the placebo (g) and *L. reuteri* (h) groups; differences between patients with and without OM at T0 (i) and T2 (j); and the impact of *L. reuteri* versus placebo on the microbiota of non-OM (**k**) and OM patients (**l**) at T2. Positive and negative coefficients indicate enrichment in the comparison and reference groups, respectively. *P* values were adjusted for multiple comparisons using the FDR correction: **P* < .05, ***P* < .01, ****P* < .001. ASV: amplicon sequence variant; PC, principal component; PCoA, principal coordinates analysis; Placebo_T0, prior to radiotherapy with a placebo; Placebo_T1, the mid-radiotherapy with a placebo; Placebo_T2, the end of radiotherapy with a placebo; *L. reuteri*, *Limosilactobacillus reuteri*; *L. reuteri*_T0, prior to radiotherapy with the probiotic *Limosilactobacillus reuteri*; *L. reuteri*_T1, the mid-radiotherapy with the probiotic *Limosilactobacillus reuteri*; *L. reuteri*_T2, the end of radiotherapy with the probiotic *Limosilactobacillus reuteri*; withoutOM_T0, baseline samples from patients who subsequently remained OM-free; withOM_T0, baseline samples from patients who subsequently developed OM; withoutOM_T2, patients without oral mucositis at the end of radiotherapy; withOM_T2, patients with oral mucositis at the end of radiotherapy; PwithoutOM_T2, patients without oral mucositis in the placebo group at the end of radiotherapy; LwithoutOM_T2, patients without oral mucositis in the *Limosilactobacillus reuteri* group at the end of radiotherapy. PwithOM_T2, patients with oral mucositis in the placebo group at the end of radiotherapy; LwithOM_T2, patients with oral mucositis in the *Limosilactobacillus reuteri* group at the end of radiotherapy.

Comment 4: *Be very careful with interpertation of the results. For example several pathobionts increased overtime in both groups not just the placebo groups, e.g. F. nucleatum and Prevotella nigrescens (compare panels g and h, Figure 4. Also, note that the taxa with -ve coefficient means association with the reference (control) group. In this case, taxa such as Scardovia wiggsiae and C. concisus at T0 are actually associated with decreased risk of OM not the other way around. Please check the directionality of associations and revise accordingly--this is the most important part of the microbial findings.*

Response to Comment 4:

We sincerely thank you for this critical and insightful comment. We have removed most interpretive statements from the Results section and thoroughly revised the microbiome-related content to ensure accurate and objective reporting of the findings. Specifically, we have updated the text to acknowledge that several pathobionts (e.g.,

Fusobacterium nucleatum and *Prevotella nigrescens*) increased significantly in both the placebo and *L. reuteri* groups following radiotherapy (Fig. 4g, h), rather than suggesting this was exclusive to the placebo group. We have corrected the directionality of *Scardovia wiggsiae* and *Campylobacter concisus* and have also revised the interpretation of coefficients throughout the manuscript to ensure accuracy and consistency with our statistical models.

Accordingly, the specific revisions made in the manuscript are as follows:

Page 10, Line 194 in the revised manuscript:

"Analysis of α diversity showed that both the Sobs and Shannon indices remained stable across the assessed time points in the *L. reuteri* group (**Fig. 4a-b**). For β diversity, principal coordinates analysis demonstrated specific shifts in community structure in the *L. reuteri* group during radiotherapy ($P = 0.001$), while no such change was shown in the placebo group (**Fig. 4c-d; Supplementary Fig. 9**). Longitudinal differential abundance analysis revealed that radiotherapy induced substantial shifts in the oral microbiome regardless of the intervention. Specifically, several opportunistic pathobionts, including *Fusobacterium nucleatum* subsp. *animalis* and *Prevotella nigrescens*, increased from T0 to T2 in both the placebo and *L. reuteri* groups **(Fig. 4g, h).** We subsequently evaluated the overall cohort to identify microbial signatures associated with OM clinical status. At T0, participants who remained OM-free throughout the study exhibited higher relative abundances of specific taxa including *Veillonella atypica* and *Streptococcus parasanguinis* clade 411 (**Fig. 4i**). By T2, the

microbial profiles of patients without OM were characterized by higher abundances of commensals (e.g., *Limosilactobacillus reuteri* and *Rothia dentocariosa*), while patients with OM showed higher abundances of *Porphyromonas pasteri* and *Capnocytophaga sputigena* (**Fig. 4j**). To further explore the associations of the probiotic intervention, we stratified the T2 cohort by OM status and compared the two treatment arms. In OM-free patients, specific taxonomic variations were observed in the *L.reuteri* group relative to the placebo group (**Fig. 4k**). Notably, in OM patients, the placebo group showed higher abundance of pathobionts (e.g., *Prevotella dentalis* and *Fusobacterium nucleatum* subsp. *animalis*), whereas the *L. reuteri* group showed higher abundance of oral commensals such as *Rothia mucilaginosa* and *Streptococcus sanguinis* despite inflammation (**Fig. 4l**)."

Reviewer #5

Comment 1: *We thank the authors for their detailed point-by-point responses and revisions. Several concerns from the previous round have been adequately addressed. However, a number of methodological issues remain unresolved or insufficiently responded to, as outlined below.*

Response to Comment 1:

We sincerely apologize that our previous revisions have not fully addressed your concerns. We have further revised the manuscript according to your valuable suggestions, with detailed point-by-point responses provided below.

Comment 2: *Following Reviewer 5's Comment 6, the authors now report a Bonferroni-corrected significance threshold of P < 0.01 (0.05/5) for secondary outcome analyses. However, restricting Bonferroni correction to the five secondary endpoints while testing the primary endpoint at an uncorrected alpha = 0.05 is only valid under a pre-specified hierarchical (gatekeeping) testing procedure, in which formal testing of secondary endpoints is conditional on the primary endpoint reaching significance. Neither the Methods section nor the SAP describes such a hierarchical structure. In the absence of a pre-specified gatekeeping procedure, all formally tested endpoints belong to a single multiplicity family, and Bonferroni correction should be applied across all six endpoints (corrected threshold ≈ 0.0083). The authors should either explicitly describe and justify a pre-specified hierarchical testing procedure in the Methods, or apply Bonferroni correction across all six endpoints.*

Response to Comment 2:

We sincerely thank you for this crucial methodological comment. We now apply a single Bonferroni correction across all six formally tested endpoints, giving a corrected two-sided significance threshold of $\alpha = 0.05/6 \approx 0.0083$. We have revised the Methods section accordingly to specify the Bonferroni-corrected significance threshold applied across all six endpoints.

Accordingly, the specific revisions made in the manuscript and Supplementary information are as follows:

Page 21, Line 454 in the revised manuscript:

"Bonferroni correction was applied for primary and secondary outcome analyses, with a corrected significance threshold of $P < 0.0083$ (0.05/6)."

Page 4 in the revised Supplementary information:

"For primary and secondary endpoints, the threshold for statistical significance was set to $P < 0.0083$ following Bonferroni correction."

Comment 3: *The Table 2 footnote states that unadjusted incidence comparisons used "the χ^2 test (RR, 95% CI)." This is imprecise: the χ^2 test yields only a P value and does not itself produce a risk ratio or confidence interval. The RR and its 95% CI must be computed separately. The footnote should clarify the method used to obtain the unadjusted RR and CI as described in the Supplementary information.*

Response to Comment 3:

We sincerely thank you for your careful review and for pointing out this terminological imprecision. In our actual statistical analysis script, the RR was calculated as the ratio of incidence proportions between the two groups, and its 95% CI was computed using the normal approximation method on the log scale (the Katz logarithmic method). We have revised the footnote in Table 2 to accurately describe the statistical methods used.

Accordingly, the specific revisions made in the footnote of Table 2 are as follows:

"Unadjusted incidence comparisons used the χ^2 test (RR and its 95% CI were computed separately using the Katz logarithmic method);"

We sincerely hope the revised manuscript has fully addressed all your comments and now meets the standards of *Nature Communications*. We would like to express our deep gratitude to you for your valuable time and constructive suggestions.
